# Marine Cytotoxin Santacruzamate A Derivatives as Potent HDAC1-3 Inhibitors and Their Synergistic Anti-Leukemia Effects with Venetoclax

**DOI:** 10.3390/md22060250

**Published:** 2024-05-28

**Authors:** Wanting Hao, Leyan Wang, Tongqiang Xu, Geng Jia, Yuqi Jiang, Chong Qin, Xiaoyang Li

**Affiliations:** Key Laboratory of Marine Drugs, Chinese Ministry of Education, School of Medicine and Pharmacy, Ocean University of China, Qingdao 266003, China; haowanting@stu.ouc.edu.cn (W.H.); 21230813049@stu.ouc.edu.cn (L.W.); xutongqiang@ouc.edu.cn (T.X.); jiageng163@163.com (G.J.); jiangyuqi@ouc.edu.cn (Y.J.); qc@ouc.edu.cn (C.Q.)

**Keywords:** Santacruzamate A, class I HDAC inhibitors, Venetoclax, combination therapy, AML cell apoptosis

## Abstract

Acute myeloid leukemia (AML) is a hematologic malignancy characterized by infiltration of the blood and bone marrow, exhibiting a low remission rate and high recurrence rate. Current research has demonstrated that class I HDAC inhibitors can downregulate anti-apoptotic proteins, leading to apoptosis of AML cells. In the present investigation, we conducted structural modifications of marine cytotoxin Santacruzamate A (SCA), a compound known for its inhibitory activity towards HDACs, resulting in the development of a novel series of potent class I HDACs hydrazide inhibitors. Representative hydrazide-based compound **25c** exhibited concentration-dependent induction of apoptosis in AML cells as a single agent. Moreover, **25c** exhibited a synergistic anti-AML effect when combined with Venetoclax, a clinical Bcl-2 inhibitor employed in AML therapy. This combination resulted in a more pronounced downregulation of anti-apoptotic proteins Mcl-1 and Bcl-xL, along with a significant upregulation of the pro-apoptotic protein cleaved-caspase3 and the DNA double-strand break biomarker γ-H2AX compared to monotherapy. These results highlighted the potential of **25c** as a promising lead compound for AML treatment, particularly when used in combination with Venetoclax.

## 1. Introduction

Acute myeloid leukemia (AML) is a heterogeneous blood disease caused by genetic mutations that give myeloid blast cells a selective growth advantage and cause them to fail to differentiate into normal blood cells. This disease is one of the most common types in the adult leukemia family, and it predominantly affects the elderly. Among the therapeutic drugs of AML, the most common standard chemotherapy combination regimen is Cytarabine/Daunorubicin (3 + 7), which involves three days of oral administration of medications such as Mitoxantrone or Idarubicin, followed by seven days of intravenous infusion of maintenance chemotherapy, and it is often used in patients < 60 years who are in good health [1]. Molecularly targeted drugs such as Bcl-2 inhibitors, FLT3 inhibitors and IDH inhibitors also show promising clinical efficacy [2,3]. Pre-clinical and clinical studies indicate that Bcl-2 inhibitors (e.g., Venetoclax) can successfully restart the unregulated apoptosis process of malignant cells with high safety, and with good synergy when combined with drugs targeting multiple molecular pathways [4], thus showing great research potential.

Tumorigenesis factors of hematological tumor diseases are often characterized by genetic aberrations that disrupt normal cellular functions, and the pathogenesis of AML is closely related to epigenetic perturbations [4,5,6]. Histone deacetylases (HDACs), a group of epigenetic anti-cancer targets, have garnered significant attention in recent years [7]. So far, a total of 18 isoforms have been found in humans, and they can be divided into two categories according to different catalytic sites: Zn^2+^-dependent proteins and NAD^+^-dependent proteins. The former consists of 11 subtypes, which are divided into three classes: class I (HDAC1, 2, 3, 8), class II (HDAC4, 5, 6, 7, 9, 10) and class IV (HDAC11). The latter consists of seven subtypes, such as class III (sirtuin 1-7) [8]. Among those, class I isoforms HDAC1-3 play a key role in regulating the acetylation levels of histones, thus modifying the chromatin structure and its accessibility for gene transcription, which is more significant in solid tumors and hematological malignancies [5].

Recent studies reveal that class I HDACs are generally overexpressed in AML cells. Depletion of HDAC1/2 promotes AML cell apoptosis, and the loss of HDAC3 can inhibit cell proliferation and promote cell differentiation of AML cells [9]. Previous studies have shown that class I HDAC inhibitor (HDACI) monotherapy downregulates anti-apoptotic proteins, leading to the activation of pro-caspase3 and triggering apoptosis in AML cells [10]. Our previous study indicated that class I HDACIs could induce G1/S cell cycle arrest leading to cell death by a p53-dependent pathway [11]. These findings demonstrate the potential of class I HDACI monotherapy in the treatment of AML. A number of class I HDACIs are currently in development, such as Entinostat [12] and Chidamide [13]. It has been found that class I HDACIs can also promote the efficacy of other drugs and have a strong effect in reversing drug resistance. Chen et al. found that the downregulation of Mcl-1 and Bcl-xL by Chidamide fully activated the pro-apoptotic gene Bim to induce apoptosis. And when combined with Venetoclax, Chidamide might have a synergistic effect and overcome the effect of drug resistance for Venetoclax in AML cells [14]. Moreover, Chidamide was used in the treatment of three R/R AML patients who failed to respond to the combination regimen of Venetoclax and Azacitidine, successfully achieving complete remission after relapse and greatly extending the life cycle of the patients [15]. Therefore, it is very necessary to develop novel class I HDACIs and explore the combination of multiple drugs.

The ocean is regarded as an important treasure house of natural medicine. Santacruzamate A (SCA), a cytotoxin isolated from Panamanian Pacific tuft cyanobacteria, was found in previous studies to have potential inhibitory activity against HDAC2 [16]. According to the virtual docking model of SCA and HDAC2 constructed by Gromek et al., the chemical structure of SCA corresponded with the pharmacophore model of traditional HDAC inhibitors, which is composed of the Cap, Linker and zinc binding group (ZBG) [17]. The group interacting with the protein surface is named “Cap”, the one binding Zn^2+^ at the bottom of the catalytic site is called “ZBG” and the group connecting these two parts in the middle hydrophobic region is termed “Linker” [11]. To a certain extent, the selectivity and potency of HDAC inhibitors are significantly dependent on the structural characteristics of their ZBGs [18]. At present, the ZBGs used in class I HDACIs mainly include hydroxamic acids, phenylamides and hydrazides. The docking results indicated that SCA formed monodentate chelation with zinc ions of HDAC2 [17]. In a previous study, we found that hydrazides as ZBG exhibited a higher titer, higher class I HDAC (1 and 3) selectivity and better pharmacokinetics than hydroxamic acids and phenylamides [11,19,20]. It is hypothesized that modifying the ZBG to form bidentate chelates may increase the HDAC inhibitory activity of SCA. Consequently, this study aimed to use the marine-derived compound SCA as a hit compound, fix ZBG as a hydrazide structure and modify Cap and Linker to obtain a new series of class I HDACIs.

## 2. Results

### 2.1. Chemistry

**Scheme 1:**
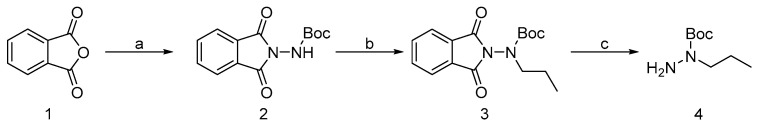
Preparation of hydrazide substituent compound **4**. Reagents and conditions: (a) tert-butyl hydrazinecarboxylate, toluene, 83% yield; (b) 1-bromopropane, TBAB and K_2_CO_3_, acetonitrile, 90% yield; (c) monomethylhydrazine, ethanol, 85% yield.

Isobenzofuran-1,3-dione (**1**) was reacted as a hydrazide acylation agent to obtain product **2**. Alkylation with bromopropane of **2** gave **3**, which was followed by dephthaloylation with methylhydrazine to give the desired compound **4** (Scheme 1).

**Scheme 2:**
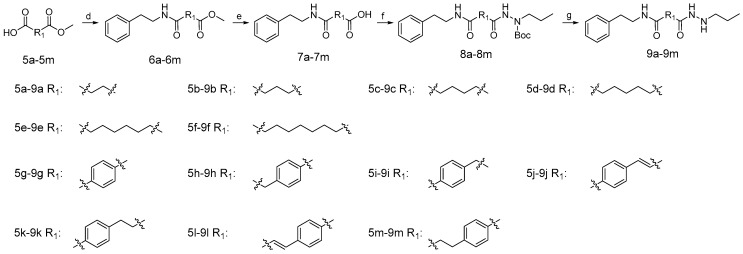
Synthesis of compounds **9a**–**9m**. Reagents and conditions: (d) 2-phenylethan-1-amine, HATU, DIPEA, DMF, 55% yield; (e) 1.5 N NaOH aqueous, MeOH; 1 N HCl aqueous, pH = 5, 85% yield; (f) **4**, HATU and DIPEA, DMF, 67% yield. (g) TFA, DCM; saturated 1 N NaOH aqueous, pH = 9, 76% yield.

Phenethylamine was condensed with the corresponding dicarboxylic acid monomethyl esters (**5a**–**5m**) to give **6a**–**6m**, which hydrolyzed under alkaline conditions to give **7a**–**7m**. The compounds **7a**–**7m** were coupled with **4** by HATU-mediated amide formation to afford **8a**–**8m**, which were finally treated with trifluoroacetic acid to yield the end products **9a**–**9m** (Scheme 2).

**Scheme 3:**
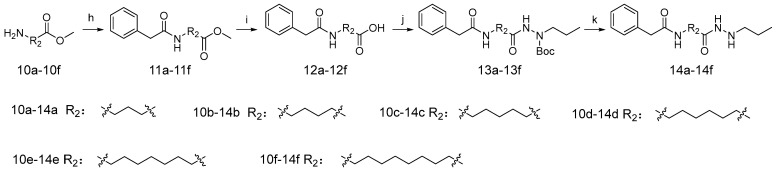
Synthesis of the compounds **14a**–**14f**. Reagents and conditions: (h) 2-phenylacetyl chloride, DIPEA, THF, 75% yield; (i) 1.5 N NaOH aqueous, MeOH; 1 N HCl aqueous, pH = 5, 85% yield; (j) **4**, HATU and DIPEA, DMF, 67% yield; (k) TFA, DCM; saturated 1 N NaOH aqueous, pH = 9, 76% yield.

Compounds **10a**–**10f** reacted with 2-phenylacetyl chloride and afforded the amides **11a**–**11f**, which were hydrolyzed under alkaline conditions to give **12a**–**12f**. The compounds **12a**–**12f** were coupled with **4** by HATU-mediated amide formation that afforded **13a**–**13f**, which were finally treated with trifluoroacetic acid to yield the end products **14a**–**14f** (Scheme 3).

**Scheme 4:**
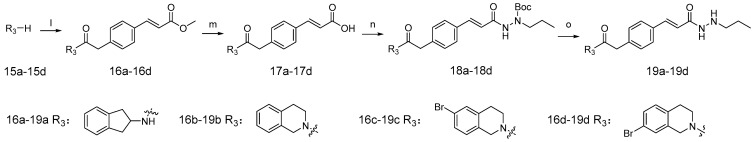
Synthesis of compounds **19a**–**19d**. Reagents and conditions: (l) 2-(4-(3-methoxy-3-oxoprop-1-en-1-yl)phenyl)acetic acid, HATU, DIPEA, DMF, 65% yield; (m) 1.5 N NaOH aqueous, MeOH; 1 N HCl aqueous, pH = 5, 85% yield; (n) **4**, HATU and DIPEA, DMF, 67% yield; (o) TFA, DCM; saturated 1 N NaOH aqueous, pH = 9, 76% yield.

2-(4-(3-methoxy-3-oxoprop-1-en-1-yl)phenyl)acetic acid was condensed with the corresponding monomethyl arylamines (**15a**–**15d**) to give **16a**–**16d**, which were hydrolyzed under alkaline conditions to give **17a**–**17d**. The compounds **17a**–**17d** were coupled with **4** by HATU-mediated amide formation that afforded **18a**–**18d**, which were finally treated with trifluoroacetic acid to yield the end products **19a**–**19d** (Scheme 4).

**Scheme 5:**
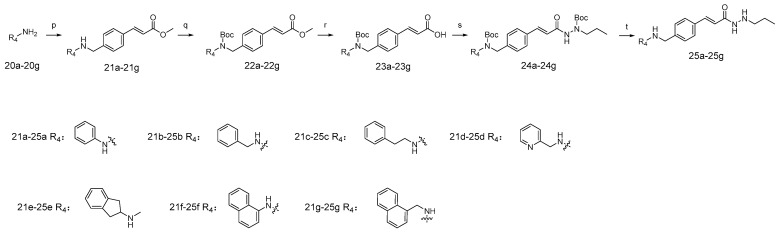
Synthesis of compounds **25a**–**25g**. Reagents and conditions: (p) methyl 3-(4-(bromomethyl)phenyl)acrylate, K_2_CO_3_, DMF, 45% yield; (q) (Boc)_2_O, TEA, DCM, 70% yield; (r) 1.5 N NaOH aqueous, MeOH; 1 N HCl aqueous, pH = 5, 85% yield; (s) **4**, HATU and DIPEA, DMF, 67% yield; (t) TFA, DCM; saturated 1 N NaOH aqueous, pH = 9, 76% yield.

Reactions of methyl 3-(4-(bromomethyl)phenyl)acrylate and the corresponding monomethyl arylamines (**20a**–**20g**) were performed to obtain the products **21a**–**21g**. Boc-protection of **21a**–**21g** gave **22a**–**22g**, which were hydrolyzed under alkaline conditions to give **23a**–**23g**. The compounds **23a**–**23g** were coupled with **4** by HATU-mediated amide formation that afforded **24a**–**24g**, which were finally treated with trifluoroacetic acid to yield the end products **25a**–**25g** (Scheme 5).

**Scheme 6:**
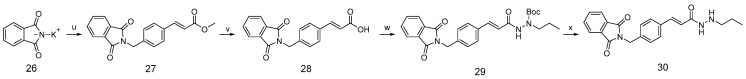
Synthesis of compound **30**. Reagents and conditions: (u) methyl 3-(4-(bromomethyl)phenyl)acrylate, K_2_CO_3_, DMF, 70% yield; (v) 1.5 N NaOH aqueous, MeOH; 1 N HCl aqueous, pH = 5, 85% yield; (w) **4**, HATU and DIPEA, DMF, 55% yield; (x) TFA, DCM; saturated 1 N NaOH aqueous, pH = 9, 80% yield.

Reaction of methyl 3-(4-(bromomethyl)phenyl)acrylate and potassium phthalimide (**26**) was performed to obtain the product **27**, which hydrolyzed under alkaline conditions to give **28**. Compound **28** was coupled with **4** by HATU-mediated amide formation that afforded **29**, which was finally treated with trifluoroacetic acid to yield the end product **30** (Scheme 6).

### 2.2. Compound Design

**Figure 1:**
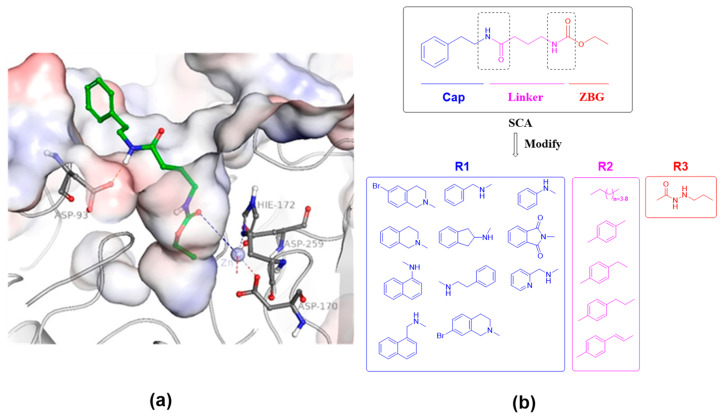
Basis of compound design. (**a**) Docking pose of SCA (green) in the binding site of HDAC3 (PDB: 4A69); (**b**) chemical structures of the designed analogs of SCA.

As reported in the original document, SCA has good inhibitory activity against class I HDACs, especially HDAC2, with an IC_50_ value of 0.112 nM [16]. However, currently designed and synthesized SCA derivatives did not significantly enhance the inhibitory activity and selectivity of HDACs, and they regrettably showed proliferation inhibition ability at the micromolar level in a variety of cancer cell lines [17,21]. Gromek et al. developed a docking model of HDAC2 and SCA and discovered that the modification of the carbamate structure of SCA might potentially enhance its HDAC inhibitory activity [16]. Specifically, the presence of a terminal oxygen in SCA limited its ability to form optimal chelation interactions, hindering activity promotion. Additionally, the planar structure of the terminal urethane extension might impact the binding conformation. Furthermore, SCA might not form hydrogen bonds with key residues like Asp104, His146 and His145 in the model [17]. Therefore, the substitution of the end ZBG of SCA is crucial for the initial optimization of the compounds. As mentioned earlier, considering the hydrazide group has shown higher activity and selectivity against HDAC1/3, we conducted a docking of SCA and HDAC3 to further verify the above conclusions (Figure 1). The results were consistent, so we decided to replace the ZBG of the SCA with a hydrazide group. Our group previously explored the structure–activity relationships (SARs) of N-substituted hydrazide-based HDACIs and found that three-carbon linker chain monosubstitution hydrazide showed the best selectivity and inhibitory effect [11]. In this study, we designed compounds with N-propyl acetyl hydrazine. Since the modification of Cap and Linker groups could potentially improve the inhibitory activity and selectivity, our initial approach tried to compare the effect of the amide order in Cap and Linker on activity and then optimize the Cap groups and Linker groups (Figure 1). These newly designed compounds were expected to have the following improvements: (1) increased selectivity for class I HDACs and (2) enhanced activity against class I HDACs and anti-leukemia activity compared to the parent molecule SCA.

The initial step involves an examination of the impact of the sequence of amide bonds connecting the Cap to the Linker and the optimal Linker length, which is analyzed through two distinct scenarios. Compound **9b** is achieved by directly modifying the ethyl carbamate of SCA with N-propyl acetyl hydrazine. Subsequent variations in the length of the Linker alkyl chain, while keeping the Cap and ZBG constant, result in the synthesis of compounds **9a** and **9c**–**9f** (Table 1). Furthermore, altering the sequence of amide bonds within the Cap region, while maintaining the alkyl chain length, leads to compound **14b**, while adjusting the length of the Linker alkyl chain results in compounds **14a** and **14c**–**14f** (Table 1). Due to the pronounced enhancement effect of the hydrazide group on the activity of HDAC1-3, especially HDAC1 and HDAC3, recombinant HDAC1/3 enzymes were utilized in the screening of the newly designed compounds for initial enzymatic inhibitory activity. The initial screening concentrations were 100 nM and 10 nM, respectively. In terms of the hydrazide sequence, phenethylamine-capped compounds **9a**–**9f** generally exhibited superior enzymatic activity compared to phenacetyl-capped compounds (**14a**–**14f**). The overall enzymatic inhibition activity of both series of compounds increased initially with the lengthening of the Linker, followed by a decrease. Notably, compounds **9e** and **14e** displayed the highest activity when the Linker was an alkyl chain with eight carbons. In addition, in our enzyme inhibitory activity determination, SCA showed no activity in the corresponding system, which was inconsistent with the original literature reports [16]. However, some literature reports later confirmed that the inhibitory activity of synthetic SCA was relatively weak [17,22].

Subsequently, given the high flexibility of the Linker of the octyldiacyl group, we endeavored to create compounds **9g**–**9m** through a rigid modification of **9e**, incorporating benzene rings and double bonds as structural constraints. In this series, the inhibitory activity of **9j**–**9l**, modified by an isoelectronic body, ware nearly double that of **9e**, with an inhibition rate toward HDAC3 exceeding 50% at 10 nM. However, the impact of double bonds and sequence on enzyme activity was deemed insignificant, as demonstrated by **9j** and **9k**. Based on the retention of the benzene ring and double bond in compound **9k**, the original amide bond was substituted with an amino group to obtain compound **25c**. Remarkably, the activity of compound **25c** surpassed that of the previously described compounds, with an inhibition rate exceeding 95% toward HDAC3 at a concentration of 10 nM. This finding underscored the essential role of the amino group in enzyme inhibition. Consequently, the structural framework of compound **25c** was retained while the Cap group was modified (Table 2).

Initially, compound **25a**, featuring an aniline group as the Cap group, exhibited inferior enzyme inhibition activity compared to compounds **25b** and **25c**, which respectively featured benzylamine and phenylethylamine groups as Cap groups. Additionally, it was observed that excessively elongating the Cap group led to undesired flexible transformations, underscoring the importance of maintaining an optimal Cap group length. In order to investigate the impact of a larger Cap group volume, the phenyl groups in compounds **25a** and **25b** were substituted with naphthalene groups to yield compounds **25f** and **25g**, respectively. However, the inhibitory activity of the latter was approximately half as potent as that of the former, indicating that a smaller Cap group volume was more favorable for the inhibitory activity against HDAC1/3. Furthermore, the substitution of the benzene ring in compound **25b** with a pyridine ring to obtain **25d** that yielded a less favorable outcome, suggesting that the presence of a nitrogen-containing heterocyclic ring might not enhance the compound’s activity. The synthesis of compounds **25e** and **30** through cyclization of the phenylethylamine group of **25c** resulted in lesser variance in activity for the former compound, while the latter activity had significantly reduced. Furthermore, a comparison of compounds **19a**–**19d**, obtained by altering the Cap group of **9k**, reiterated that a bulky Cap group was not ideal for enzyme binding. These findings hold significant implications for the strategic development of hydrazine HDACIs (Table 3).

Based on the preliminary screening results mentioned above, we comprehensively selected 10 compounds (**9j**, **9k**, **9l**, **25a**, **25b**, **25c**, **25d**, **25e**, **25g** and **30**) to determine their IC_50_s toward HDAC1-3 (Table 4). The results were largely as anticipated and consistent with the findings from the initial screening. Intuitively, **9j**, **25b**, **25c** and **25e** showed relatively higher HDAC1-3 activity. Finally, compounds **25b**, **25c**, **25e** and **25g** were chosen for subsequent cell-based activity screening.

### 2.3. Cell Proliferation Inhibition

We first examined the anti-proliferative activity of **25b**, **25c**, **25e** and **25g** against four AML-associated cell lines (MV4-11, THP-1, HL60 and Kasumi-1 cells). The cell viability after compound treatment was evaluated by resorufin intensity. The class I HDACs selective inhibitor Entinostat was chosen as a positive control. Table 5 summarized the IC_50_ of the selected compounds in four cell lines. The selected compounds could inhibit the growth of the above four kinds of cells. Among the tested compounds, **25c** displayed the best anti-proliferative activity, with IC_50_ values of 337.6–480.3 nM. However, no inhibitory activity was detected in the synthesized SCA (Table 5).

### 2.4. HDAC Isoform Selectivity of ***25c***

**Figure 2:**
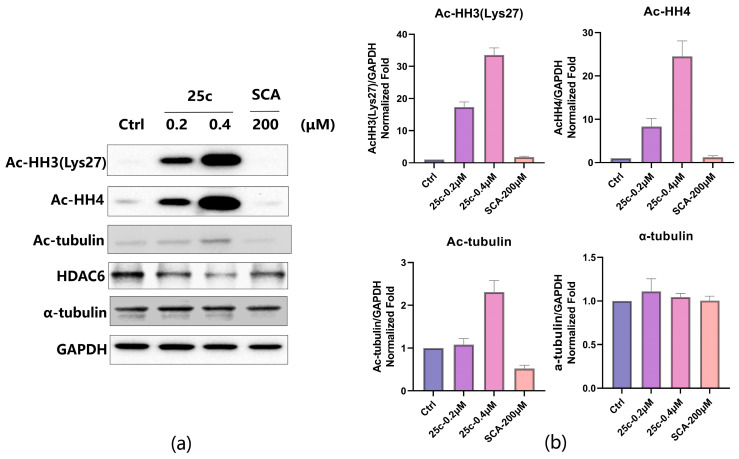
Western blot analysis of the enzyme substrates that interact with **25c** in MV4-11 cells. (**a**) Western blot analysis of AcHH3(Lys27), AcHH4, Ac-tubulin, HDAC6 and α-tubulin in MV4-11 cells after treatment of compound **25c** and SCA at the concentrations of 0.2, 0.4 and 200 μM for 24 h; (**b**) The expression levels of AcHH3(Lys27), AcHH4, Ac-tubulin and α-tubulin were quantified. Data are shown as mean ± SEM from three independent experiments.

Enzymatic and cell-based assays indicate that **25c** was the most potent HDACI among all of the compounds we designed. Thus, we chose **25c** for further biological study. To assess the HDAC isoforms selectivity of **25c**, we determined its IC_50_ value for other HDAC subtypes (HDAC4, 5, 6, 7, 8, 9 and 11) (Table 6). As there were no appropriate substrates for HDAC10, the inhibitory activity toward this isotype was not determined. The inhibitory activity of compound **25c** for HDAC4, 5, 6, 7, 9 and 11 exceeded 10,000 nM, and its inhibitory activity for HDAC8 was 4109 nM, so **25c** was a class I HDAC selective inhibitor. We also conducted Western blot analysis to validate the selectivity of **25c**. The results indicated that compound **25c** significantly elevated the level of acetylated histone H3 (AcHH3) and acetylated histone H4 (AcHH4), aligning with the inhibitory effects on HDAC1, 2 and 3. Surprisingly, the level of acetyl-tubulin increased even though **25c** did not inhibit HDAC6, a protein responsible for the deacetylation of tubulin. Our hypothesis conjectured that the upregulation of Ac-tubulin, a substrate of HDAC6, could be attributed to the transcriptional inhibition of HDAC6 (Figure 2).

### 2.5. ***25c*** and Venetoclax Synergistically Inhibit Cell Proliferation

**Figure 3:**
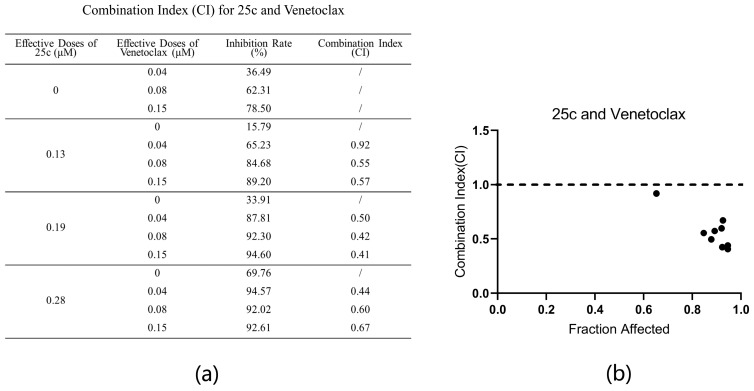
Combination index (CI) values for **25c** and Venetoclax after treatment for 72 h. (**a**) Inhibition rate and CI for the combination of **25c** and Venetoclax after treatment for 72 h. (**b**) Fraction affected and CI for **25c** and Venetoclax after treatment for 72 h. Data were analyzed using CompuSyn Software. CIs of <1, =1 and >1 indicate synergism, additive effect and antagonism, respectively.

Venetoclax is a drug used to treat specific types of leukemia and lymphoma, known as a Bcl-2 inhibitor. Bcl-2 is a protein that plays a key role in regulating apoptosis. In some types of leukemia and lymphoma, cancer cells evade the natural mechanism of apoptosis by overexpressing Bcl-2, leading to the oversurvival of cancer cells [23]. During Venetoclax treatment, acquired drug resistance is a common phenomenon, and the compensatory high expression of Mcl-1 may be one of the causes of drug resistance in AML cells [24]. According to previous reports, both class I HDAC inhibitors and Venetoclax could induce apoptosis and perform a synergistic effect [24]. As the IC_50_ of **25c** measured in the above experiments was 340.1 ± 82.5 nM in the MV4-11 cell line, showing the more obvious effect among the four cell lines, we selected the MV4-11 cell line for the combination cell assay and analyzed the data according to the Chou–Talalay method [25]. The results indicated that the inhibitory effect of the combination of **25c** with Venetoclax was higher than the inhibitory effect of the single agents. When the concentration of **25c** was higher than 0.13 μM, the CI values of Venetoclax were all less than 1, indicating that the two drugs had a strong synergistic effect (Figure 3).

### 2.6. Venetoclax and ***25c*** Synergistically Promote Apoptosis in AML Cell

**Figure 4:**
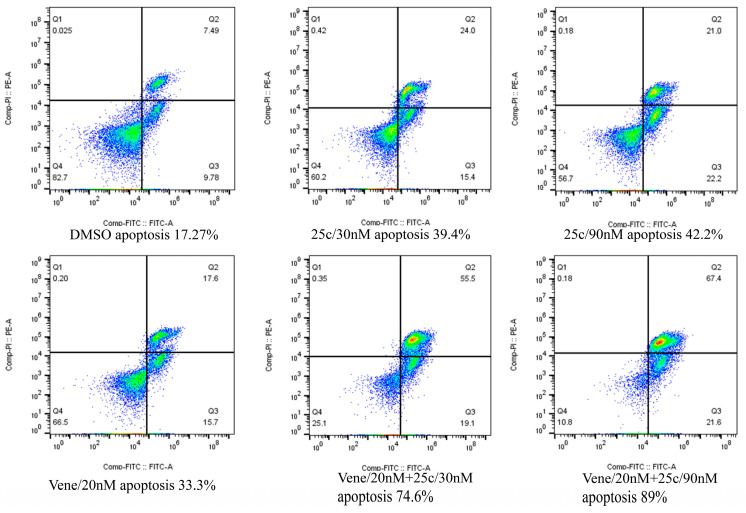
Apoptosis analysis of **25c** and Venetoclax in MV4-11 for 24 h.

According to previous studies and our experiments, both **25c** and Venetoclax could induce apoptosis. In order to further explore the synergistic effect of combination drugs, we performed flow cytometry to detect apoptosis of single and combination drugs by analyzing Annexin V levels and propidium iodide permeability. MV4-11 cells were exposed to **25c** and Venetoclax individually and in combination for a duration of 24 h. The results indicated a notable decrease in the apoptosis of MV4-11 cells when treated with the combined treatment compared to either drug alone. The observed rise in apoptosis rate was attributed to the synergistic effect of the drug combination, rather than the sum effect of the individual drugs (Figure 4).

**Figure 5:**
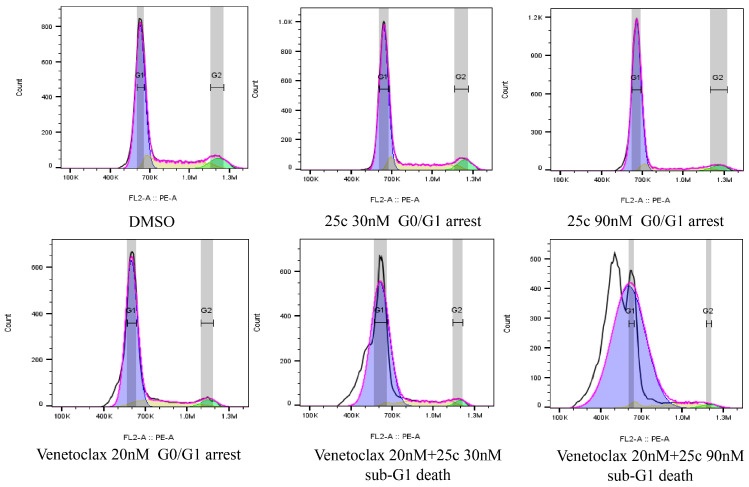
Cell cycle analysis of **25c** and Venetoclax in MV4-11 cells for 24 h.

Next, we exposed the MV4-11 cell line to the same drug concentrations and time as the apoptosis experiment and then measured the cell cycle distribution using flow cytometry. Numerous studies have confirmed that HDACIs induce cell cycle arrest in the G0/G1 phase in vitro [26]. As can be seen from Figure 5, the proportion of the S phase and G2/M phase decreased in cells treated with **25c**, while the proportion of the G0/G1 phase increased. The results showed that **25c** could block MV4-11 cells in the G0/G1 phase and exert the effect of cell cycle arrest and cell proliferation inhibition. However, the combination of the two drugs could induce sub-G1 cell death and apoptosis significantly (Figure 5).

### 2.7. Effects of ***25c*** and Venetoclax on Transcriptomes in Multiple Genes and Signaling Pathways in MV4-11 Cells

**Figure 6:**
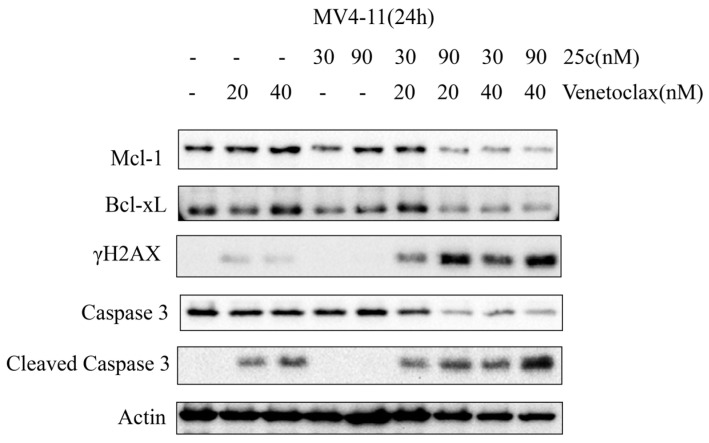
Changes in Bcl-2 family apoptotic regulatory proteins and apoptotic pathway proteins detected by Western blot.

No substantial alterations were noted in the level of cleaved-caspase3 at concentrations of 30 nM and 90 nM in the **25c** monotherapy group, but the level in the Venetoclax monotherapy group was slightly higher; however, a heightened level of cleaved-caspase3 was observed in the combined treatment group which was higher than any single drug effect, suggesting that the combination led to an increased apoptotic effect. γ-H2AX protein is recognized as a specific biomarker following DNA double-strand breaks, thereby suggesting an escalation in DNA damage subsequent to drug combination. The levels of γ-H2AX protein in the two monotherapy groups surpassed those of the control group, leading to enhanced efficacy in the combined treatment group. Furthermore, the expression of anti-apoptotic proteins Mcl-1 and Bcl-xL in the Bcl-2 protein family was significantly reduced in the combination treatment cohort, which may be involved in apoptosis induction and cell resistance mechanisms (Figure 6).

### 2.8. Study on Metabolic Stability of ***25c*** In Vitro

**Figure 7:**
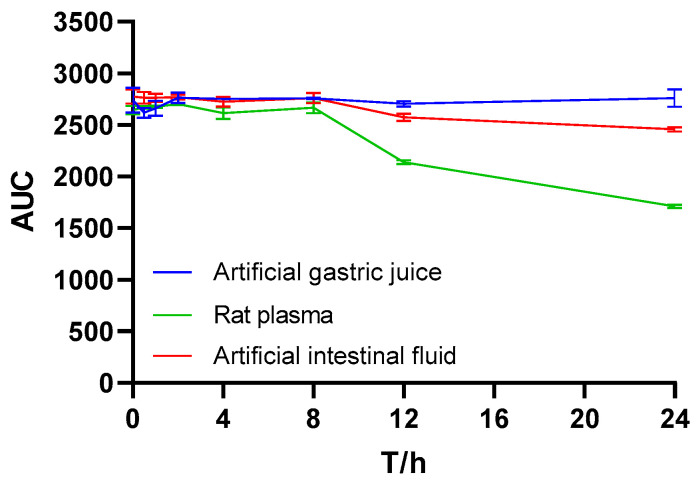
Stability of compound **25c** in rat plasma, artificial gastroenteric fluid and artificial intestinal fluid within 24 h.

After verifying the in vitro antitumor activity of **25c** and its synergistic effect in combination with Venetoclax, we conducted in vitro stability studies. **25c** remained relatively stable in artificial gastric juice, and degradation occurred in artificial intestinal juice and rat plasma, beginning after about 8 h, with degradation of 12.52% and 35.60%, respectively (Figure 7).

## 3. Discussion

In this study, we modified the structure of SCA through the substitution of the original ZBG with hydrazide group and modifications to the Cap and Linker components. The designed and synthesized compounds exhibited significantly improved HDAC inhibitory activity, with compound **25c** demonstrating the highest potency in both HDAC enzyme inhibition and cell-based antitumor assays. Compound **25c** was identified as a class I HDAC selective inhibitor, displaying IC_50_ values of 28.1 nM, 134.9 nM and 2.4 nM against HDAC1, 2 and 3, respectively. Furthermore, the IC_50_ value of **25c** against four AML cell lines ranged from 337.6 to 480.3 nM, indicating its potential anti-AML activity in vitro. Flow cytometry and Western blot analysis revealed that **25c** facilitated the cleavage of caspase 3, resulting in apoptosis and inhibition of cell proliferation in MV4-11 cells.

Additionally, the co-administration of Venetoclax demonstrated a synergistic enhancement of anti-proliferative activity in AML cell lines, as evidenced by the calculated CI value at specific concentrations. When used in combination with Venetoclax, **25c** demonstrated potentially and synergistically enhanced cytotoxicity. Additionally, Figure 4 and Figure 5 clearly show an increase in the sub-G1 cell population, indicating that the combined treatment of the two drugs synergistically induced apoptosis, particularly promoting late apoptosis in the MV4-11 cell line. Furthermore, the expression levels of γH2AX, Mcl-1 and Bcl-xL decreased following the combination of **25c** and Venetoclax. The former was associated with DNA damage, while the latter was linked to the inhibition of apoptosis; however, the specific underlying mechanism remained unclear.

Furthermore, prior research has indicated that elevated levels of Mcl-1/Bcl-xL may serve as a contributing factor to drug resistance to Venetoclax. It has been observed that **25c** has the potential to counteract this resistance, thus implying that **25c** could potentially mitigate the issue of drug resistance to Venetoclax.

## 4. Materials and Methods

### 4.1. General Chemistry

All chemical solvents and reagents were purchased from chemical vendors, such as Bidepharm, Aladdin, Macklin, Energy and Leyan, and used as received without further purification unless otherwise noted. ^1^H NMR and ^13^C NMR data were collected in DMSO-*d*_6_/CDCl_3_ using a JMTC-400 MHz (Japan Superconductor Technology, Inc, Takatsukdai, Japan) instrument or an Agilent One Probe-500 MHz spectrometer (Agilent Technologies UK Ltd, Oxford, England) with TMS as an internal standard. Chemical shifts (*δ*) and coupling constants (*J*) were reported in parts per million (ppm) and hertz (Hz), respectively. Mass spectral data were collected on a Waters Acquity QDa mass detector (Waters, Singapore and Ireland). All target compounds were >95% pure by HPLC analysis, performed on an Agilent 1260 Infinity II HPLC instrument (Agilent Technologies, Waldbronn, Germany) using an Agilent 5 TC-C18 (2) C18 column (5 μm, 4.6 mm × 250 mm) using an equivalent method of potassium phosphate buffer/acetonitrile.

#### Procedure for Preparation of Target Compounds

Tert-butyl (1,3-dioxoisoindolin-2-yl)carbamate (**2**). Isobenzofuran-1,3-dione (**1**, 60 g, 462 mmol) and tert-butyl hydrazinecarboxylate (65 g, 440 mmol) were dissolved in toluene (50 mL) at 115 °C for 36 h. After the reaction was finished, the toluene was removed under vacuum. The solid was dispersed in petroleum ether/AcOEt (10:1) solution, stirred for 1 h, and then filtered to obtain a white solid (112 g, 83% yield). ^1^H NMR (400 MHz, CDCl_3_) *δ* 7.91–7.87 (m, 2H), 7.79–7.75 (m, 2H), 6.73 (s, 1H), 1.49 (s, 9H).

Tert-butyl (1,3-dioxoisoindolin-2-yl)(propyl)carbamate (**3**). Compound **2** (10.0 g, 38.15 mmol) was dissolved in acetonitrile (30 mL) at 80 °C, and to this solution was added K_2_CO_3_ (15.8 g, 114.46 mmol) and tetrabutylammonium bromide (3.7 g, 11.45 mmol) for 0.5 h. Then, 1-bromopropane (5.6 g, 45.79 mmol) was added. The reaction mixture was allowed to stir at 80 °C overnight and was monitored by TLC. After the reaction finished, the residue was washed with brine and dried over anhydrous Na_2_SO_4_. The volatiles were removed under vacuum to yield a pale yellow oil (8.8 g, 90% yield). ^1^H NMR (400 MHz, CDCl_3_) *δ* 7.87–7.81 (m, 2H), 7.78–7.70 (m, 2H), 3.59–3.54 (m, 2H), 1.59–1.50 (m, 2H), 1.47 (s, 4H), 1.27 (s, 5H), 0.91 (t, *J* = 7.4 Hz, 3H).

Tert-butyl 1-propylhydrazine-1-carboxylate (**4**). Compound **3** (7.4 g, 24 mmol) was dissolved in ethanol (30 mL), and the reaction mixture was cooled to −10 °C, and to this solution was added monomethylhydrazine (5.6 g, 48 mmol) to reflux 8 h. After the reaction was finished, the ethanol was removed under vacuum to obtain a crude product which was purified via flash column chromatography to give compound **4**, a white oil (3.8 g, 85% yield). ^1^H NMR (400 MHz, CDCl_3_) *δ* 3.95 (s, 2H), 3.32 (t, *J* = 7.2 Hz, 2H), 1.62–1.53 (m, 2H), 1.46 (s, 9H), 0.87 (t, *J* = 7.4 Hz, 3H).

Methyl 4-oxo-4-(phenethylamino)butanoate (**6a**). 4-Methoxy-4-oxobutanoic acid (**5a**, 250 mg, 1.89 mmol) and HATU (1077 mg, 2.84 mmol) were dissolved in anhydrous DMF (2 mL) under an ice bath followed by the addition of DIPEA (738 mg, 5.67 mmol). The mixture was stirred at 0 °C for 30 min. 2-Phenylethan-1-amine (275 mg, 2.27 mmol) was added dropwise. After that, the reaction mixture was stirred at room temperature (r.t.) for 6 h, and the reaction was monitored by TLC (CH_2_Cl_2_/MeOH 25:1). Then, the reaction solution was diluted with AcOEt, and the mixture was washed with 1 N HCl, saturated NaHCO_3_, and brine three times. The organic layer was dried over anhydrous Na_2_SO_4_ and evaporated under vacuum. The crude product was purified by chromatographic using petroleum ether/AcOEt (1:1) as mobile phase to obtain **6a** as a pale yellow solid (238 mg, 48% yield). ^1^H NMR (400 MHz, CDCl_3_) *δ* 7.37 (t, *J* = 7.0 Hz, 2H), 7.33–7.28 (m, 1H), 7.25 (d, *J* = 7.7 Hz, 2H), 5.69 (s, 1H), 3.73 (s, 3H), 3.60–3.55 (m, 2H), 2.87 (t, *J* = 6.1 Hz, 2H), 2.71 (t, *J* = 5.9 Hz, 2H), 2.48 (t, *J* = 5.9 Hz, 2H).

Methyl 5-oxo-5-(phenethylamino)pentanoate (**6b**). Using the synthetic method of compound **6a**, 5-methoxy-5-oxopentanoic acid (**5b**) and 2-phenylethan-1-amine gave **6b** as a pale yellow solid (48% yield). ^1^H NMR (400 MHz, CDCl_3_) *δ* 7.37 (t, *J* = 6.9 Hz, 2H), 7.31 (t, *J* = 6.3 Hz, 1H), 7.25 (d, *J* = 7.7 Hz, 2H), 5.60 (s, 1H), 3.72 (s, 3H), 3.61–3.56 (m, 2H), 2.88 (t, *J* = 6.0 Hz, 2H), 2.40 (t, *J* = 7.1 Hz, 2H), 2.24 (t, *J* = 7.4 Hz, 2H), 2.02–1.95 (m, 2H).

Methyl 6-oxo-6-(phenethylamino)hexanoate (**6c**). Using the synthetic method of compound **6a**, 6-methoxy-6-oxohexanoic acid (**5c**) and 2-phenylethan-1-amine gave **6c** as a pale yellow solid (50% yield). ^1^H NMR (400 MHz, CDCl_3_) *δ* 7.29 (t, *J* = 7.5 Hz, 2H), 7.22 (d, *J* = 6.7 Hz, 1H), 7.18 (d, *J* = 8.0 Hz, 2H), 5.53 (s, 1H), 3.65 (s, 3H), 3.53–3.48 (m, 2H), 2.80 (t, *J* = 7.0 Hz, 2H), 2.34–2.25 (m, 2H), 2.18–2.07 (m, 2H), 1.61 (t, *J* = 3.9 Hz, 4H).

Methyl 7-oxo-7-(phenethylamino)heptanoate (**6d**). Using the synthetic method of compound **6a**, 7-methoxy-7-oxoheptanoic acid (**5d**) and 2-phenylethan-1-amine gave **6d** as a pale yellow solid (52% yield). The resulting crude product was directly used in the next step of the reaction.

Methyl 8-oxo-8-(phenethylamino)octanoate (**6e**). Using the synthetic method of compound **6a**, 8-methoxy-8-oxooctanoic acid (**5e**) and 2-phenylethan-1-amine gave **6e** as a pale yellow solid (45% yield). ^1^H NMR (400 MHz, CDCl_3_) *δ* 7.26 (t, *J* = 7.5 Hz, 2H), 7.22–7.17 (m, 1H), 7.14 (d, *J* = 7.5 Hz, 2H), 5.42 (s, 1H), 3.61 (s, 3H), 3.49–3.44 (m, 2H), 2.76 (t, *J* = 6.9 Hz, 2H), 2.24 (t, *J* = 7.4 Hz, 2H), 2.06 (t, *J* = 7.5 Hz, 2H), 1.57–1.52 (m, 4H), 1.31–1.22 (m, 4H).

Methyl 9-oxo-9-(phenethylamino)nonanoate (**6f**). Using the synthetic method of compound **6a**, 9-methoxy-9-oxononanoic acid (**5f**) and 2-phenylethan-1-amine gave **6f** as a pale yellow solid (20% yield). ^1^H NMR (400 MHz, CDCl_3_) *δ* 7.32–7.27 (m, 2H), 7.24–7.15 (m, 3H), 3.65 (s, 3H), 3.54–3.47 (m, 2H), 2.81 (d, *J* = 6.9 Hz, 2H), 2.28 (t, *J* = 7.5 Hz, 2H), 2.13–2.07 (m, 2H), 1.62–1.53 (m, 4H), 1.32–1.25 (m, 6H).

Methyl 4-(phenethylcarbamoyl)benzoate (**6g**). Using the synthetic method of compound **6a**, 4-(methoxycarbonyl)benzoic acid (**5g**) and 2-phenylethan-1-amine gave **6g** as a pale yellow solid (41% yield). ^1^H NMR (400 MHz, DMSO-*d*_6_) *δ* 8.76 (t, *J* = 5.9 Hz, 1H), 8.03 (d, *J* = 8.0 Hz, 2H), 7.93 (d, *J* = 8.0 Hz, 2H), 7.30 (t, *J* = 7.4 Hz, 2H), 7.27–7.16 (m, 3H), 3.88 (s, 3H), 3.53–3.47 (m, 2H), 2.86 (t, *J* = 7.5 Hz, 2H).

Methyl 4-(2-oxo-2-(phenethylamino)ethyl)benzoate (**6h**). Using the synthetic method of compound **6a**, 2-(4-(methoxycarbonyl)phenyl)acetic acid (**5h**) and 2-phenylethan-1-amine gave **6h** as a pale yellow solid (60% yield). ESI-MS *m*/*z*: 319.87 [M + Na]^+^.

Methyl 2-(4-(phenethylcarbamoyl)phenyl)acetate (**6i**). Using the synthetic method of compound **6a**, 4-(2-methoxy-2-oxoethyl)benzoic acid (**5i**) and 2-phenylethan-1-amine gave **6i** as a pale yellow solid (55% yield). The resulting crude product was directly used in the next step of the reaction.

Methyl-3-(4-(phenethylcarbamoyl)phenyl)acrylate (**6j**). Using the synthetic method of compound **6a**, 4-(3-methoxy-3-oxoprop-1-en-1-yl)benzoic acid (**5j**) and 2-phenylethan-1-amine gave **6j** as a pale yellow solid (60% yield). ^1^H NMR (400 MHz, DMSO-*d*_6_) *δ* 8.64 (t, *J* = 5.6 Hz, 1H), 7.86–7.80 (m, 4H), 7.69 (d, *J* = 16.1 Hz, 1H), 7.30 (t, *J* = 7.2 Hz, 2H), 7.26–7.17 (m, 3H), 6.74 (d, *J* = 16.1 Hz, 1H), 3.74 (s, 3H), 3.52–3.46 (m, 2H), 2.85 (t, *J* = 7.4 Hz, 2H). ESI-MS *m*/*z*: 311.74 [M + H]^+^.

Methyl 3-(4-(phenethylcarbamoyl)phenyl)propanoate (**6k**). Using the synthetic method of compound **6a**, 4-(3-methoxy-3-oxopropyl)benzoic acid (**5k**) and 2-phenylethan-1-amine gave **6k** as a pale yellow solid (60% yield). ^1^H NMR (400 MHz, CDCl_3_) *δ* 7.38–7.34 (m, 2H), 7.34 (d, *J* = 1.5 Hz, 2H), 7.32–7.28 (m, 2H), 7.26 (s, 1H), 7.23 (d, *J* = 6.7 Hz, 2H), 3.56 (s, 3H), 3.45–3.41 (m, 2H), 3.18 (d, *J* = 5.6 Hz, 2H), 2.80 (s, 2H). ESI-MS *m*/*z*: 333.86 [M + Na]^+^.

Methyl-4-(3-oxo-3-(phenethylamino)prop-1-en-1-yl)benzoate (**6l**). Using the synthetic method of compound **6a**, 3-(4-(methoxycarbonyl)phenyl)acrylic acid (**5l**) and 2-phenylethan-1-amine gave **6l** as a pale yellow solid (63% yield). ESI-MS *m*/*z*: 331.87 [M + Na]^+^.

Methyl 4-(3-oxo-3-(phenethylamino)propyl)benzoate (**6m**). Using the synthetic method of compound **6a**, 3-(4-(methoxycarbonyl)phenyl)propanoic acid (**5m**) and 2-phenylethan-1-amine gave **6m** as a pale yellow solid (42% yield). The resulting crude product was directly used in the next step of the reaction.

4-Oxo-4-(phenethylamino)butanoic acid (**7a**). Compound **6a** (225 mg, 1 mmol) was dissolved in 4 mL of methanol, and to this solution was added 1.5 mL of a 1 mol/L NaOH aqueous solution. The mixture was stirred for 8 h, and the reaction was monitored by TLC (DCM/methanol 25:1). The methanol was evaporated under vacuum and acidified by 1 N HCl, and then the solid was extracted, filtered, and washed many times with water; the product **7a** was obtained after drying, a white solid (195 mg, 90% yield). ^1^H NMR (400 MHz, DMSO-*d*_6_) *δ* 12.06 (s, 1H), 7.93 (t, *J* = 5.4 Hz, 1H), 7.31–7.26 (m, 2H), 7.23–7.16 (m, 3H), 3.27–3.22 (m, 2H), 2.72–2.66 (m, 2H), 2.44–2.37 (m, 2H), 2.29 (t, *J* = 7.3 Hz, 2H).

5-Oxo-5-(phenethylamino)pentanoic acid (**7b**). Using the synthetic method of compound **7a**, compound **6b** gave **7b** as a white solid (80% yield). ^1^H NMR (400 MHz, DMSO-*d*_6_) *δ* 11.98 (s, 1H), 7.84 (t, *J* = 5.7 Hz, 1H), 7.29–7.18 (m, 2H), 7.18–7.09 (m, 3H), 3.27–3.17 (m, 2H), 2.66 (t, *J* = 7.4 Hz, 2H), 2.14 (t, *J* = 7.4 Hz, 2H), 2.03 (t, *J* = 7.4 Hz, 2H), 1.69–1.61 (m, 2H).

6-Oxo-6-(phenethylamino)hexanoic acid (**7c**). Using the synthetic method of compound **7a**, compound **6c** gave **7c** as a white solid (82% yield). ESI-MS *m*/*z*: 249.78 [M + H]^+^.

7-Oxo-7-(phenethylamino)heptanoic acid (**7d**). Using the synthetic method of compound **7a**, compound **6d** gave **7d** as a white solid (81% yield). The resulting crude product was directly used for the next reaction.

8-Oxo-8-(phenethylamino)octanoic acid (**7e**). Using the synthetic method of compound **7a**, compound **6e** gave **7e** as a white solid (87% yield). ^1^H NMR (400 MHz, DMSO-*d*_6_) *δ* 11.92 (s, 1H), 7.79 (t, *J* = 5.7 Hz, 1H), 7.26–7.23 (m, 2H), 7.19–7.11 (m, 3H), 3.24–3.19 (m, 2H), 2.66 (t, *J* = 7.4 Hz, 2H), 2.14 (t, *J* = 7.4 Hz, 2H), 1.98 (t, *J* = 7.4 Hz, 2H), 1.47–1.37 (m, 4H), 1.23–1.12 (m, 4H). ESI-MS *m*/*z*: 299.83 [M + H]^+^.

9-Oxo-9-(phenethylamino)nonanoic acid (**7f**). Using the synthetic method of compound **7a**, compound **6f** gave **7f** as a white solid (87% yield). ^1^H NMR (400 MHz, DMSO-*d*_6_) *δ* 7.79 (t, *J* = 5.6 Hz, 1H), 7.29–7.21 (m, 2H), 7.17–7.13 (m, 3H), 3.26–3.17 (m, 2H), 2.65 (t, *J* = 7.4 Hz, 2H), 2.14 (t, *J* = 7.4 Hz, 2H), 1.98 (t, *J* = 7.4 Hz, 2H), 1.49–1.35 (m, 4H), 1.22–1.13 (m, 6H).

4-(Phenethylcarbamoyl)benzoic acid (**7g**). Using the synthetic method of compound **7a**, compound **6g** gave **7g** as a white solid (85% yield). ^1^H NMR (400 MHz, DMSO-*d*_6_) 13.12 (s, 1H), *δ* 8.69 (t, *J* = 5.6 Hz, 1H), 8.02–7.93 (m, 2H), 7.90–7.83 (m, 2H), 7.32–7.12 (m, 5H), 3.51–3.42 (m, 2H), 2.86–2.78 (m, 2H).

4-(2-Oxo-2-(phenethylamino)ethyl)benzoic acid (**7h**). Using the synthetic method of compound **7a**, compound **6h** gave **7h** as a white solid (85% yield). ^1^H NMR (400 MHz, DMSO-*d*_6_) *δ* 8.12 (t, *J* = 5.6 Hz, 1H), 7.82 (d, *J* = 8.3 Hz, 2H), 7.31–7.26 (m, 2H), 7.26–7.20 (m, 2H), 7.17–7.11 (m, 3H), 3.27–3.22 (m, 2H), 2.67 (t, *J* = 7.3 Hz, 2H).

2-(4-(Phenethylcarbamoyl)phenyl)acetic acid (**7i**). Using the synthetic method of compound **7a**, compound **6i** gave **7i** as a white solid (75% yield). The resulting crude product was directly used for the next reaction.

3-(4-(Phenethylcarbamoyl)phenyl)acrylic acid (**7j**). Using the synthetic method of compound **7a**, compound **6j** gave **7j** as a white solid (91% yield). ^1^H NMR (400 MHz, DMSO-*d*_6_) *δ* 8.60 (t, *J* = 5.7 Hz, 1H), 7.81 (d, *J* = 8.4 Hz, 2H), 7.73 (d, *J* = 8.3 Hz, 2H), 7.58 (d, *J* = 16.0 Hz, 1H), 7.30–7.12 (m, 5H), 6.59 (d, *J* = 16.1 Hz, 1H), 3.50–3.40 (m, 2H), 2.81 (t, *J* = 7.4 Hz, 2H). ESI-MS *m*/*z*: 317.85 [M + Na]^+^.

3-(4-(Phenethylcarbamoyl)phenyl)propanoic acid (**7k**). Using the synthetic method of compound **7a**, compound **6k** gave **7k** as a white solid (81% yield). ^1^H NMR (400 MHz, DMSO-*d*_6_) *δ* 8.45 (t, *J* = 5.6 Hz, 1H), 7.70 (d, *J* = 8.0 Hz, 2H), 7.27–7.24 (m, 4H), 7.21–7.14 (m, 3H), 3.50–3.39 (m, 2H), 2.84–2.78 (m, 4H), 2.52 (t, *J* = 7.6 Hz, 2H).

4-(3-Oxo-3-(phenethylamino)prop-1-en-1-yl)benzoic acid (**7l**). Using the synthetic method of compound **7a**, compound **6l** gave **7l** as a white solid (80% yield). ^1^H NMR (400 MHz, DMSO-*d*_6_) *δ* 8.24 (t, *J* = 5.7 Hz, 1H), 7.92 (d, *J* = 8.3 Hz, 2H), 7.62 (d, *J* = 8.3 Hz, 2H), 7.42 (d, *J* = 15.8 Hz, 1H), 7.31–7.23 (m, 2H), 7.23–7.12 (m, 3H), 6.69 (d, *J* = 15.8 Hz, 1H), 3.41–3.39 (m, 2H), 2.75 (t, *J* = 7.3 Hz, 2H).

4-(3-Oxo-3-(phenethylamino)propyl)benzoic acid (**7m**). Using the synthetic method of compound **7a**, compound **6m** gave **7m** as a white solid (80% yield). ^1^H NMR (400 MHz, DMSO-*d*_6_) *δ* 7.91 (t, *J* = 5.7 Hz, 1H), 7.84 (d, *J* = 8.2 Hz, 2H), 7.32–7.28 (m, 2H), 7.28–7.24 (m, 2H), 7.21–7.16 (m, 1H), 7.15–7.10 (m, 2H), 3.26–3.21 (m, 2H), 2.86 (t, *J* = 7.6 Hz, 2H), 2.65 (t, *J* = 7.3 Hz, 2H), 2.38 (t, *J* = 7.6 Hz, 2H).

Tert-butyl-2-(4-oxo-4-(phenethylamino)butanoyl)-1-propylhydrazine-1-carboxylate (**8a**). Compound **7a** (190 mg, 0.86 mmol) and HATU (490 mg, 1.29 mmol) were dissolved in anhydrous DMF (2 mL) under an ice bath followed by the addition of DIPEA (334 mg, 2.58 mmol). The mixture was stirred at 0 °C for 30 min. Compound **4** (180 mg, 1.03 mmol), was added dropwise; after that, the reaction mixture was stirred at r.t. for 6 h, and the reaction was monitored by TLC (CH_2_Cl_2_/MeOH 25:1). Then, the reaction solution was diluted with AcOEt, and the mixture was washed with 1 N HCl, saturated NaHCO_3_, and brine three times. The organic layer was dried over anhydrous Na_2_SO_4_ and evaporated under vacuum. The crude product was purified by chromatographic using DCM/methanol (1:1) as mobile phase to obtain **8a** as a white solid (185 mg. 56% yield). ^1^H NMR (400 MHz, DMSO-*d*_6_) *δ* 9.89 (s, 1H), 7.95 (t, *J* = 5.5 Hz, 1H), 7.31–7.25 (m, 2H), 7.19 (d, *J* = 5.3 Hz, 3H), 4.34 (t, *J* = 5.1 Hz, 2H), 3.47–3.42 (m, 2H), 3.24 (d, *J* = 4.7 Hz, 2H), 2.71–2.66 (m, 2H), 1.35 (s, 9H), 1.07–1.03 (m, 4H), 0.82 (t, *J* = 7.2 Hz, 3H).

Tert-butyl-2-(6-oxo-6-(phenethylamino)hexanoyl)-1-propylhydrazine-1-carboxylate (**8b**). Using the synthetic method of **8a**, compound **7b** and compound **4** gave **8b** as a white solid (69% yield). ^1^H NMR (400 MHz, CDCl_3_) *δ* 7.34–7.26 (m, 2H), 7.25–7.15 (m, 3H), 3.61 (s, 2H), 3.28 (s, 2H), 2.87 (s, 2H), 2.21–2.15 (m, 2H), 1.96–1.91 (m, 2H), 1.77 (s, 4H), 1.46 (s, 9H), 0.87 (t, *J* = 7.4 Hz, 3H).

Tert-butyl-2-(6-oxo-6-(phenethylamino)hexanoyl)-1-propylhydrazine-1-carboxylate (**8c**). Using the synthetic method of **8a**, compound **7c** and compound **4** gave **8c** as a white solid (75% yield). ^1^H NMR (400 MHz, DMSO-*d*_6_) *δ* 9.81 (s, 1H), 7.81 (t, *J* = 5.9 Hz, 1H), 7.25 (t, *J* = 7.5 Hz, 2H), 7.16 (d, *J* = 7.1 Hz, 3H), 3.24–3.19 (m, 4H), 2.65 (t, *J* = 7.5 Hz, 2H), 2.01 (d, *J* = 6.5 Hz, 4H), 1.44 (s, 6H), 1.33 (m, 9H), 0.79 (t, *J* = 7.5 Hz, 3H).

Tert-butyl-2-(7-oxo-7-(phenethylamino)heptanoyl)-1-propylhydrazine-1-carboxylate (**8d**). Using the synthetic method of **8a**, compound **7d** and compound **4** gave **8d** as a white solid (60% yield). ^1^H NMR (400 MHz, CDCl_3_) *δ* 7.27 (d, *J* = 7.9 Hz, 2H), 7.21 (d, *J* = 7.0 Hz, 1H), 7.16 (d, *J* = 7.5 Hz, 2H), 3.51–3.46 (m, 2H), 3.39 (t, *J* = 7.3 Hz, 2H), 2.78 (t, *J* = 6.9 Hz, 2H), 2.16–2.12 (m, 4H), 1.65–1.57 (m, 4H), 1.55–1.48 (m, 2H), 1.42 (d, *J* = 7.4 Hz, 8H), 1.33 (t, *J* = 7.8 Hz, 2H), 0.87 (t, *J* = 7.4Hz, 3H).

Tert-butyl-2-(8-oxo-8-(phenethylamino)octanoyl)-1-propylhydrazine-1-carboxylate (**8e**). Using the synthetic method of **8a**, compound **7e** and compound **4** gave **8e** as a white solid (47% yield). ^1^H NMR (400 MHz, CDCl_3_) *δ* 7.40–7.35 (m, 2H), 7.32–7.28 (m, 1H), 7.25 (d, *J* = 7.6 Hz, 2H), 3.60–3.55 (m, 2H), 3.49 (t, *J* = 7.3 Hz, 2H), 2.88 (t, *J* = 7.1 Hz, 2H), 2.24–2.15 (m, 4H), 1.75–1.58 (m, 6H), 1.54 (s, 9H), 1.41–1.35 (m, 4H), 0.96 (t, *J* = 7.2 Hz, 3H).

Tert-butyl-2-(9-oxo-9-(phenethylamino)nonanoyl)-1-propylhydrazine-1-carboxylate (**8f**). Using the synthetic method of **8a**, compound **7f** and compound **4** gave **8f** as a white solid (53% yield). ^1^H NMR (400 MHz, CDCl_3_) *δ* 7.71 (s, 1H), 7.29–7.25 (m, 2H), 7.22–7.12 (m, 3H), 5.72 (s, 1H), 3.52–3.44 (m, 2H), 3.43–3.37 (m, 2H), 2.77 (d, *J* = 7.1 Hz, 2H), 2.14 (t, *J* = 7.6 Hz, 2H), 2.08 (t, *J* = 7.6 Hz, 2H), 1.66–1.49 (m, 6H), 1.41 (s, 9H), 1.26 (d, *J* = 5.5 Hz, 6H), 0.86 (t, *J* = 7.4 Hz, 3H).

Tert-butyl-2-(4-(phenethylcarbamoyl)benzoyl)-1-propylhydrazine-1-carboxylate (**8g**). Using the synthetic method of **8a**, compound **7g** and compound **4** gave **8g** as a white solid (49% yield). ^1^H NMR (400 MHz, DMSO-*d*_6_) *δ* 10.61 (s, 1H), 8.70 (s, 1H), 7.90–7.86 (m, 4H), 7.30 (t, *J* = 7.6 Hz, 2H), 7.25–7.18 (m, 3H), 3.52–3.47 (m, 2H), 3.39 (s, 2H), 2.86 (t, *J* = 7.5 Hz, 2H), 1.53 (s, 2H), 1.39 (s, 9H), 0.89 (t, *J* = 7.3 Hz, 3H).

Tert-butyl-2-(4-(2-oxo-2-(phenethylamino)ethyl)benzoyl)-1-propylhydrazine-1-carboxylate (**8h**). Using the synthetic method of **8a**, compound **7h** and compound **4** gave **8h** as a white solid (53% yield). The resulting crude product was directly used for the next reaction.

Tert-butyl-2-(2-(4-(phenethylcarbamoyl)phenyl)acetyl)-1-propylhydrazine-1-carboxylate (**8i**). Using the synthetic method of **8a**, compound **7i** and compound **4** gave **8i** as a white solid (76% yield). ^1^H NMR (400 MHz, DMSO-*d*_6_) *δ* 7.75 (d, *J* = 7.9 Hz, 1H), 7.65 (d, *J* = 8.3 Hz, 1H), 7.37–7.19 (m, 8H), 3.47–3.42 (m, 4H), 2.85–2.83 (m, 2H),2.69 (d, *J* = 1.1 Hz, 2H), 1.53–1.43 (m, 2H), 1.41–1.34 (s, 9H), 1.05 (t, *J* = 7.0 Hz, 3H).

Tert-butyl-2-(3-(4-(phenethylcarbamoyl)phenyl)acryloyl)-1-propylhydrazine-1-carboxylate (**8j**). Using the synthetic method of **8a**, compound **7j** and compound **4** gave **8j** as a white solid (75% yield). The resulting crude product was directly used in the next step of the reaction.

Tert-butyl-2-(3-(4-(phenethylcarbamoyl)phenyl)propanoyl)-1-propylhydrazine-1-carboxylate (**8k**). Using the synthetic method of **8a**, compound **7k** and compound **4** gave **8k** as a white solid (70% yield). ^1^H NMR (400 MHz, DMSO-*d*_6_) *δ* 9.90 (s, 1H), 8.47 (t, *J* = 5.1 Hz, 1H), 7.74 (d, *J* = 8.3 Hz, 2H), 7.30–7.28 (m, 2H), 7.27 (s, 2H), 7.24 (d, *J* = 1.7 Hz, 1H), 7.22–7.17 (m, 2H), 3.50–3.44 (m, 2H), 3.19 (s, 2H), 2.90–2.82 (m, 4H), 2.40 (t, *J* = 7.6 Hz, 2H), 1.39 (s, 2H), 1.32 (s, 9H), 0.78 (t, *J* = 7.4 Hz, 3H).

Tert-butyl-2-(4-(3-oxo-3-(phenethylamino)prop-1-en-1-yl)benzoyl)-1-propylhydrazine-1-carboxylate (**8l**). Using the synthetic method of **8a**, compound **7l** and compound **4** gave **8l** as a white solid (49% yield). ^1^H NMR (400 MHz, DMSO-*d*_6_) *δ* 10.56 (s, 1H), 8.25 (t, *J* = 5.5 Hz, 1H), 7.84 (d, *J* = 8.3 Hz, 1H), 7.66 (d, *J* = 8.4 Hz, 2H), 7.45 (d, *J* = 15.8 Hz, 1H), 7.29 (d, *J* = 7.5 Hz, 2H), 7.26–7.20 (m, 3H), 6.71 (d, *J* = 15.8 Hz, 1H), 4.34 (t, *J* = 5.1 Hz, 4H), 2.80 (d, *J* = 7.4 Hz, 2H), 1.51 (d, *J* = 7.4 Hz, 2H), 1.33 (s, 9H), 0.89 (t, *J* = 8.0 Hz, 3H).

Tert-butyl-2-(4-(3-oxo-3-(phenethylamino)propyl)benzoyl)-1-propylhydrazine-1-carboxylate (**8m**). Using the synthetic method of **8a**, compound **7m** and compound **4** gave **8m** as a white solid (43% yield). ^1^H NMR (400 MHz, DMSO-*d*_6_) *δ* 10.40 (s, 1H), 7.89–7.84 (m, 1H), 7.70 (d, *J* = 9.5 Hz, 1H), 7.27–7.21 (m, 4H), 7.15 (d, *J* = 7.4 Hz, 1H), 7.10 (d, *J* = 7.4 Hz, 2H), 4.31 (t, *J* = 5.1 Hz, 2H), 3.23–3.18 (m, 2H), 2.82 (t, *J* = 7.4 Hz, 3H), 2.62 (t, *J* = 7.3 Hz, 2H), 2.34 (t, *J* = 7.6 Hz, 2H), 1.50–1.43 (m, 2H), 1.34 (s, 9H), 0.84 (t, *J* = 7.9 Hz, 3H).

4-Oxo-N-phenethyl-4-(2-propylhydrazineyl)butanamide (**9a**). Compound **8a** (170 mg, 0.97 mmol) was dissolved in 4 mL of a mixed solution of DCM and TFA (2:1); the solution was stirred at r.t. for 2 h. The reaction was monitored by TLC. After completion, the volatiles were removed under vacuum. The pH was adjusted to 9 with 1 N NaOH aqueous. The mixture was extracted with DCM, the combined DCM layer was dried over anhydrous Na_2_SO_4_. The solvent was removed in vacuum and the residue was purified via flash column chromatography to give compound **9a**. The resulting residues were purified by chromatography using DCM/methanol (15:1) as mobile phase to obtain **9a** as a white solid (139 mg, 82% yield). ^1^H NMR (400 MHz, DMSO-*d*_6_) *δ* 9.23 (s, 1H), 7.92 (t, *J* = 5.6 Hz, 1H), 7.28 (t, *J* = 7.5 Hz, 2H), 7.19 (d, *J* = 7.1 Hz, 3H), 4.74 (s, 1H), 3.26–3.21 (m, 2H), 2.68 (t, *J* = 7.5 Hz, 2H), 2.60 (t, *J* = 7.2 Hz, 2H), 2.28 (d, *J* = 6.5 Hz, 2H), 2.23 (d, *J* = 6.4 Hz, 2H), 1.42–1.32 (m, 2H), 0.85 (t, *J* = 7.4 Hz, 3H). ^13^C NMR (126 MHz, DMSO-*d*_6_) *δ* 171.36, 170.30, 137.08, 129.35, 128.60, 126.70, 53.47, 42.91, 39.01, 33.91, 29.50, 29.13, 29.07, 28.93, 26.77, 25.65, 21.21, 12.05. HRMS (AP-ESI) *m*/*z*: calcd for C_15_H_23_N_3_O_2_ [M + Na]^+^, 300.16825; found, 300.16800. HPLC analysis of compound **9a** (purity: 100.0%).

5-Oxo-N-phenethyl-5-(2-propylhydrazineyl)pentanamide (**9b**). Using the synthetic method of **9a**, compound **8b** gave **9b** as a white solid (36% yield). ^1^H NMR (400 MHz, DMSO-*d*_6_) *δ* 9.21 (s, 1H), 7.87 (s, 1H), 7.28 (t, *J* = 6.8 Hz, 2H), 7.19 (d, *J* = 7.1 Hz, 3H), 4.76 (s, 1H), 3.28–3.22 (m, 2H), 2.69 (t, *J* = 7.3 Hz, 2H), 2.61 (t, *J* = 7.1 Hz, 2H), 2.05–1.97 (m, 4H), 1.75–1.65 (m, 2H), 1.42–1.33 (m, 2H), 0.86 (t, *J* = 6.6 Hz, 3H). ^13^C NMR (126 MHz, DMSO-*d*_6_) δ 171.99, 139.99, 129.07, 128.74, 126.49, 53.50, 40.58, 35.67, 35.19, 33.41, 21.98, 21.22, 12.05. HRMS (AP-ESI) *m*/*z*: calcd for C_16_H_25_N_3_O_2_ [M + Na]^+^, 314.18390 found, 314.18372. HPLC analysis of compound **9b** (purity: 99.80%).

6-Oxo-N-phenethyl-6-(2-propylhydrazineyl)hexanamide (**9c**). Using the synthetic method of **9a**, compound **8c** gave **9c** as a white solid (42% yield). ^1^H NMR (400 MHz, CDCl_3_) *δ* 7.41 (s, 1H), 7.33–7.27 (m, 2H), 7.25–7.21 (m, 1H), 7.21–7.15 (m, 2H), 5.69 (s, 1H), 3.55–3.48 (m, 2H), 2.83–2.73 (m, 4H), 2.17–2.12 (m, 4H), 1.64–1.60 (m, 4H), 1.51–1.44 (m, 2H), 0.93 (t, *J* = 7.4 Hz, 3H). ^13^C NMR (126 MHz, DMSO-*d*_6_) *δ* 172.28, 171.21, 129.07, 128.74, 126.48, 53.48, 40.56, 35.66 (d, *J* = 5.0 Hz), 33.81, 25.40 (d, *J* = 2.6 Hz), 21.21, 12.05. HRMS (AP-ESI) *m*/*z*: calcd for C_17_H_27_N_3_O_2_ [M + H]^+^, 306.21760; found, 306.21771. HPLC analysis of compound **9c** (purity: 100.0%).

7-Oxo-N-phenethyl-7-(2-propylhydrazineyl)heptanamide (**9d**). Using the synthetic method of **9a**, compound **8d** gave **9d** as a white solid (55% yield). ^1^H NMR (400 MHz, CDCl_3_) *δ* 7.31 (t, *J* = 7.4 Hz, 2H), 7.25–7.18 (m, 3H), 5.54 (s, 1H), 3.53–3.48 (m, 2H), 2.83–2.75 (m, 4H), 2.12 (t, *J* = 7.6 Hz, 4H), 1.67–1.59 (m, 4H), 1.53–1.43 (m, 2H), 1.35–1.27 (m, 2H), 0.92 (t, *J* = 7.4 Hz, 3H). ^13^C NMR (126 MHz, DMSO-*d*_6_) *δ* 172.38, 171.31, 139.99, 129.06, 128.73, 126.47, 53.49, 40.55, 35.73, 35.69, 33.84, 28.65, 25.48, 21.21, 12.04. HRMS (AP-ESI) *m*/*z*: calcd for C_18_H_29_N_3_O_2_ [M + H]^+^, 320.23325; found, 320.23337. HPLC analysis of compound **9d** (purity: 96.65%).

8-Oxo-N-phenethyl-8-(2-propylhydrazineyl)octanamide (**9e**). Using the synthetic method of **9a**, compound **8e** gave **9d** as a white solid (65% yield). ^1^H NMR (400 MHz, CDCl_3_) *δ* 7.29 (d, *J* = 7.5 Hz, 1H), 7.21 (d, *J* = 7.2 Hz, 1H), 7.16 (d, *J* = 7.4 Hz, 3H), 5.52 (s, 1H), 3.51–3.46 (m, 2H), 2.80–2.73 (m, 4H), 2.08 (t, *J* = 7.5 Hz, 4H), 1.63–1.54 (m, 4H), 1.48–1.41 (m, 2H), 1.33–1.24 (m, 4H), 0.90 (t, *J* = 7.4 Hz, 3H). ^13^C NMR (126 MHz, DMSO-*d*_6_) *δ* 172.43, 171.36, 139.99, 129.08, 128.71, 126.47, 53.46, 40.52, 35.83, 35.66, 33.92, 28.82, 25.68, 25.59, 21.21, 12.06. HRMS (AP-ESI) *m*/*z*: calcd for C_19_H_31_N_3_O_2_ [M + H]^+^, 334.24890; found, 334.24878. HPLC analysis of compound **9e** (purity: 98.71%).

9-Oxo-N-phenethyl-9-(2-propylhydrazineyl)nonanamide (**9f**). Using the synthetic method of **9a**, compound **8f** gave **9f** as a white solid (53% yield). ^1^H NMR (400 MHz, DMSO-*d*_6_) *δ* 9.20 (s, 1H), 7.82 (t, *J* = 5.8 Hz, 1H), 7.31–7.24 (m, 2H), 7.22–7.15 (m, 3H), 4.77 (s, 1H), 3.28–3.21 (m, 2H), 2.69 (t, *J* = 7.3 Hz, 2H), 2.60 (t, *J* = 7.1 Hz, 2H), 2.05–1.94 (m, 4H), 1.50–1.42 (m, 4H), 1.38–1.32 (m, 2H), 1.21 (d, *J* = 5.9 Hz, 6H), 0.85 (t, *J* = 7.5 Hz, 3H). ^13^C NMR (126 MHz, DMSO-*d*_6_) *δ* 172.44, 171.36, 139.99, 129.06, 128.71, 126.47, 53.48, 40.50, 35.84, 35.67, 33.91, 28.98, 28.95, 28.89, 25.70, 25.64, 21.21, 12.04. HRMS (AP-ESI) *m*/*z*: calcd for C_20_H_33_N_3_O_2_ [M + H]^+^, 348.26455; found, 348.26443. HPLC analysis of compound **9f** (purity: 99.64%).

N-Phenethyl-4-(2-propylhydrazine-1-carbonyl)benzamide (**9g**). Using the synthetic method of **9a**, compound **8g** gave **9g** as a white solid (73% yield). ^1^H NMR (400 MHz, DMSO-*d*_6_) *δ* 10.13 (d, *J* = 5.3 Hz, 1H), 8.72 (t, *J* = 5.5 Hz, 1H), 7.88 (s, 4H), 7.29 (t, *J* = 7.4 Hz, 2H), 7.26–7.16 (m, 3H), 5.12 (d, *J* = 6.6 Hz, 1H), 3.49 (d, *J* = 7.5 Hz, 2H), 2.85 (t, *J* = 7.5 Hz, 2H), 2.75 (d, *J* = 6.4 Hz, 2H), 1.51–1.42 (m, 2H), 0.91 (t, *J* = 7.4 Hz, 3H). ^13^C NMR (126 MHz, DMSO-*d*_6_) *δ* 165.87, 164.98, 139.96, 137.21, 135.81, 129.11, 128.79, 127.61, 127.47, 126.54, 53.47, 41.38, 35.47, 21.29, 12.11. HRMS (AP-ESI) *m*/*z*: calcd for C_19_H_23_N_3_O_2_ [M + H]^+^, 326.18630; found, 326.18600. HPLC analysis of compound **9g** (purity: 99.52%).

N-Phenethyl-2-(4-(2-propylhydrazine-1-carbonyl)phenyl)acetamide (**9h**). Using the synthetic method of **9a**, compound **8h** gave **9h** as a white solid (71% yield). ^1^H NMR (400 MHz, DMSO-*d*_6_) *δ* 9.54 (s, 1H), 8.52 (t, *J* = 5.5 Hz, 1H), 7.74 (d, *J* = 8.3 Hz, 2H), 7.33–7.27 (m, 4H), 7.25–7.17 (m, 3H), 4.85 (s, 1H), 3.50–3.43 (m, 2H), 3.40 (s, 2H), 2.83 (t, *J* = 7.4 Hz, 2H), 2.61 (t, *J* = 7.1 Hz, 2H), 1.42–1.32 (m, 2H), 0.85 (t, *J* = 7.4 Hz, 3H). ^13^C NMR (126 MHz, DMSO-*d*_6_) *δ* 168.98, 166.51, 139.97, 139.85, 133.19, 129.21, 129.12, 128.80, 127.53, 126.55, 41.31, 35.53, 21.14. HRMS (AP-ESI) *m*/*z*: calcd for C_20_H_25_N_3_O_2_ [M + H]^+^, 340.20195; found, 340.20212. HPLC analysis of compound **9h** (purity: 100.0%).

4-(2-Oxo-2-(2-propylhydrazineyl)ethyl)-N-phenethylbenzamide (**9i**). Using the synthetic method of **9a**, compound **8i** gave **9i** as a white solid (60% yield). ^1^H NMR (400 MHz, DMSO-*d*_6_) *δ* 11.77 (s, 1H), 8.59 (t, *J* = 5.6 Hz, 1H), 7.79 (d, *J* = 7.9 Hz, 2H), 7.38 (d, *J* = 8.2 Hz, 2H), 7.29 (t, *J* = 7.4 Hz, 2H), 7.26–7.16 (m, 3H), 3.66 (s, 2H), 3.50–3.45 (m, 2H), 3.00 (d, *J* = 15.6 Hz, 2H), 2.84 (t, *J* = 7.4 Hz, 2H), 1.65–1.55 (m, 2H), 0.89 (t, *J* = 7.5 Hz, 3H). ^13^C NMR (126 MHz, DMSO-*d*_6_) *δ* 169.29, 166.28, 140.00, 138.18, 133.65, 129.50, 129.13, 128.80, 127.71, 126.55, 51.63, 41.32, 39.83, 35.56, 17.96, 11.27. HRMS (AP-ESI) *m*/*z*: calcd for C_20_H_25_N_3_O_2_ [M + H]^+^, 340.20195; found, 340.20184. HPLC analysis of compound **9i** (purity: 100.0%).

4-(3-Oxo-3-(2-propylhydrazineyl)prop-1-en-1-yl)-N-phenethylbenzamide (**9j**). Using the synthetic method of **9a**, compound **8j** gave **9j** as a white solid (61% yield). ^1^H NMR (400 MHz, DMSO-*d*_6_) *δ* 9.65 (s, 1H), 8.61 (t, *J* = 5.5 Hz, 1H), 7.84 (d, *J* = 8.2 Hz, 2H), 7.63 (d, *J* = 8.3 Hz, 2H), 7.48 (d, *J* = 15.9 Hz, 1H), 7.30 (t, *J* = 7.5 Hz, 2H), 7.26–7.19 (m, 3H), 6.62 (d, *J* = 15.9 Hz, 1H), 5.06 (s, 1H), 3.53–3.44 (m, 2H), 2.85 (t, *J* = 7.4 Hz, 2H), 2.72–2.67 (m, 2H), 1.48–1.39 (m, 2H), 0.89 (t, *J* = 7.4 Hz, 3H). ^13^C NMR (126 MHz, DMSO-*d*_6_) *δ* 166.00, 163.96, 139.95, 137.97, 137.87, 135.46, 129.12, 128.81, 128.18, 127.79, 126.56, 122.27, 53.48, 35.52, 21.23, 12.02. HRMS (AP-ESI) *m*/*z*: calcd for C_21_H_25_N_3_O_2_ [M + H]^+^, 352.20195; found, 352.20187. HPLC analysis of compound **9j** (purity: 97.40%).

4-(3-Oxo-3-(2-propylhydrazineyl)propyl)-N-phenethylbenzamide (**9k**). Using the synthetic method of **9a**, compound **8k** gave **9k** as a white solid (61% yield). ^1^H NMR (400 MHz, DMSO-*d*_6_) *δ* 9.23 (s, 1H), 8.47 (t, *J* = 5.6 Hz, 1H), 7.73 (d, *J* = 1.7 Hz, 1H), 7.72 (d, *J* = 1.9 Hz, 1H), 7.31–7.17 (m, 7H), 4.78 (s, 1H), 3.50–3.42 (m, 2H), 2.89–2.79 (m, 4H), 2.55 (t, *J* = 7.1 Hz, 2H), 2.33 (t, *J* = 7.6 Hz, 2H), 1.35–1.26 (m, 2H), 0.82 (t, *J* = 7.4 Hz, 3H). ^13^C NMR (126 MHz, DMSO-*d*_6_) *δ* 165.12, 139.89, 138.04, 137.94, 134.05, 129.10, 128.81, 128.09, 127.82, 126.57, 124.32, 53.50, 40.83, 35.54, 21.30, 12.13. HRMS (AP-ESI) *m*/*z*: calcd for C_21_H_27_N_3_O_2_ [M − H]^+^, 352.20195; found, 352.20206. HPLC analysis of compound **9k** (purity: 100%).

N-Phenethyl-3-(4-(2-propylhydrazine-1-carbonyl)phenyl)acrylamide (**9l**). Using the synthetic method of **9a**, compound **8l** gave **9l** as a white solid (47% yield). ^1^H NMR (400 MHz, DMSO-*d*_6_) *δ* 10.07 (s, 1H), 8.26 (t, *J* = 5.7 Hz, 1H), 7.84 (d, *J* = 8.1 Hz, 2H), 7.62 (d, *J* = 8.0 Hz, 2H), 7.44 (d, *J* = 15.8 Hz, 1H), 7.30 (t, *J* = 7.5 Hz, 2H), 7.24–7.18 (m, 3H), 6.70 (d, *J* = 15.8 Hz, 1H), 5.13 (s, 1H), 3.43 (t, *J* = 7.3 Hz, 2H), 2.80–2.73 (m, 4H), 1.51–1.42 (m, 2H), 0.91 (t, *J* = 7.4 Hz, 3H). ^13^C NMR (126 MHz, DMSO-*d*_6_) *δ* 165.10, 139.87, 138.03, 134.08, 129.10, 128.81, 128.07, 127.84, 126.59, 124.23, 53.50, 40.82, 35.55, 21.30, 12.13. HRMS (AP-ESI) *m*/*z*: calcd for C_21_H_25_N_3_O_2_ [M + H]^+^, 352.20195; found, 352.20200. HPLC analysis of compound **9l** (purity: 98.12%).

N-Phenethyl-3-(4-(2-propylhydrazine-1-carbonyl)phenyl)propanamide (**9m**). Using the synthetic method of **9a**, compound **8m** gave **9m** as a white solid (42% yield). ^1^H NMR (400 MHz, DMSO-*d*_6_) *δ* 9.96 (s, 1H), 7.93 (t, *J* = 5.7 Hz, 1H), 7.76–7.69 (m, 2H), 7.25 (d, *J* = 7.9 Hz, 4H), 7.21–7.15 (m, 1H), 7.13 (d, *J* = 7.0 Hz, 2H), 5.14 (s, 1H), 3.28–3.18 (m, 2H), 2.83 (t, *J* = 7.6 Hz, 2H), 2.73 (t, *J* = 7.1 Hz, 2H), 2.65 (t, *J* = 7.3 Hz, 2H), 2.36 (t, *J* = 7.6 Hz, 2H), 1.50–1.41 (m, 2H), 0.90 (t, *J* = 7.4 Hz, 3H). ^13^C NMR (126 MHz, DMSO-*d*_6_) *δ* 171.45, 165.60, 145.30, 139.95, 131.32, 129.07, 128.73, 128.63, 127.50, 126.48, 53.56, 40.63, 37.01, 35.62, 31.30, 21.30, 12.14. HRMS (AP-ESI) *m*/*z*: calcd for C_21_H_25_N_3_O_2_ [M + H]^+^, 354.21760; found, 354.21738. HPLC analysis of compound **9m** (purity: 99.81%).

Methyl 4-(2-phenylacetamido)butanoate (**11a**). Methyl 4-aminobutanoate (**10a**, 250 mg, 1.62 mmol) was dissolved in DMF (2 mL) under an ice bath followed by the addition of TEA (654 mg, 6.47 mmol). The mixture was stirred at 0 °C for 30 min. 2-Phenylacetyl chloride (497 mg, 4.24 mmol) was added dropwise. After that, the reaction was stirred at r.t. for 6 h, and the reaction was monitored by TLC (CH_2_Cl_2_/MeOH 25:1). Then, DMF solution was washed with 1 N HCl, saturated NaHCO_3_, and brine three times, dried over anhydrous Na_2_SO_4_, and evaporated under vacuum. The crude product was purified by chromatography using petroleum ether/AcOEt (1:1) as mobile phase to obtain **11a** as a white solid (200 mg, yield 45%). ^1^H NMR (400 MHz, DMSO-*d*_6_) *δ* 8.05 (t, *J* = 5.7 Hz, 1H), 7.35–7.26 (m, 2H), 7.26–7.18 (m, 3H), 3.58 (s, 3H), 3.38 (s, 2H), 3.10–3.00 (m, 2H), 2.30 (t, *J* = 7.5 Hz, 2H), 1.68–1.61 (m, 2H). ESI-MS *m*/*z*: 257.79 [M + Na]^+^.

Methyl 5-(2-phenylacetamido)pentanoate (**11b**). Using the synthetic method of **11a**, methyl 5-aminopentanoate (**10b**) and 2-phenylacetyl chloride gave **11b** as a pale yellow solid (70% yield). ^1^H NMR (400 MHz, CDCl_3_) *δ* 7.34 (t, *J* = 7.6 Hz, 2H), 7.28 (d, *J* = 7.9 Hz, 1H), 7.23 (s, 2H), 5.47 (s, 1H), 3.63 (s, 3H), 3.55 (s, 2H), 3.22–3.17 (m, 2H), 2.27 (t, *J* = 7.0 Hz, 2H), 1.59–1.52 (m, 2H), 1.48–1.42 (m, 2H).

Methyl 6-(2-phenylacetamido)hexanoate (**11c**). Using the synthetic method of **11a**, methyl 6-aminohexanoate (**10c**) and 2-phenylacetyl chloride gave **11c** as a pale yellow solid (65% yield). ^1^H NMR (400 MHz, DMSO-*d*_6_) *δ* 7.96 (t, *J* = 5.7 Hz, 1H), 7.26–7.15 (m, 5H), 3.54 (s, 3H), 3.34 (s, 2H), 3.00–2.96 (m, 2H), 2.23 (t, *J* = 7.5 Hz, 2H), 1.51–1.43 (m, 2H), 1.38–1.31 (m, 2H), 1.24–1.16 (m, 2H).

Methyl 7-(2-phenylacetamido)heptanoate (**11d**). Using the synthetic method of **11a**, methyl 7-aminoheptanoate (**10d**) and 2-phenylacetyl chloride gave **11d** as a pale yellow solid (58% yield). ^1^H NMR (400 MHz, CDCl_3_) *δ* 7.36–7.32 (m, 2H), 7.30–7.26 (m, 1H), 7.24 (s, 1H), 7.23 (d, *J* = 1.9 Hz, 1H), 5.36 (s, 1H), 3.64 (d, *J* = 0.6 Hz, 3H), 3.55 (s, 2H), 3.20–3.15 (m, 2H), 2.25 (t, *J* = 7.5 Hz, 2H), 1.60–1.52 (m, 2H), 1.43–1.36 (m, 2H), 1.30–1.19 (m, 4H).

Methyl 8-(2-phenylacetamido)octanoate (**11e**). Using the synthetic method of **11a**, methyl 8-aminooctanoate (**10e**) and 2-phenylacetyl chloride gave **11e** as a pale yellow solid (60% yield). ^1^H NMR (400 MHz, CDCl_3_) *δ* 7.36 (t, *J* = 6.9 Hz, 2H), 7.31 (s, 1H), 7.25 (d, *J* = 7.6 Hz, 2H), 5.33 (s, 1H), 3.66 (d, *J* = 2.0 Hz, 3H), 3.56 (d, *J* = 2.0 Hz, 2H), 3.21–3.16 (m, 2H), 2.28 (t, *J* = 6.5 Hz, 2H), 1.62–1.55 (m, 2H), 1.44–1.36 (m, 2H), 1.28–1.19 (m, 6H).

Methyl 9-(2-phenylacetamido)nonanoate (**11f**). Using the synthetic method of **11a**, methyl 9-aminononanoate (**10f**) and 2-phenylacetyl chloride gave **11f** as a pale yellow solid (43% yield). ^1^H NMR (400 MHz, CDCl_3_) *δ* 7.38–7.34 (m, 2H), 7.32–7.28 (m, 3H), 3.66 (s, 3H), 3.57 (s, 2H), 3.21–3.15 (m, 2H), 2.29 (t, *J* = 7.5 Hz, 2H), 1.63–1.56 (m, 2H), 1.43–1.36 (m, 2H), 1.30–1.23 (m, 8H). ESI–MS *m*/*z*: 328.87 [M + Na]^+^.

4-(2-Phenylacetamido)butanoic acid (**12a**). Using the synthetic method of compound **7a**, compound **11a** gave **12a** as a white solid (75% yield). ^1^H NMR (400 MHz, DMSO-*d*_6_) *δ* 12.01 (s, 1H), 8.01 (t, *J* = 5.6 Hz, 1H), 7.31–7.23 (m, 2H), 7.23–7.14 (m, 3H), 3.35 (s, 2H), 3.08–2.97 (m, 2H), 2.17 (t, *J* = 7.4 Hz, 2H), 1.62–1.54 (m, 2H).

5-(2-Phenylacetamido)pentanoic acid (**12b**). Using the synthetic method of compound **7a**, compound **11b** gave **12b** as a white solid (72% yield). ^1^H NMR (400 MHz, DMF-*d*7) *δ* 8.02 (t, *J* = 5.7 Hz, 1H), 7.31–7.18 (m, 5H), 3.38 (s, 2H), 3.05–3.00 (m, 2H), 2.19 (t, *J* = 7.2 Hz, 2H), 1.54–1.44 (m, 2H), 1.43–1.34 (m, 2H).

6-(2-Phenylacetamido)hexanoic acid (**12c**). Using the synthetic method of compound **7a**, compound **11c** gave **12c** as a white solid (69% yield). The resulting crude product was directly used for the next reaction. ESI-MS *m*/*z*: 249.77 [M + H]^+^.

7-(2-Phenylacetamido)heptanoic acid (**12d**). Using the synthetic method of compound **7a**, compound **11d** gave **12d** as a white solid (82% yield). ^1^H NMR (400 MHz, DMSO-*d*_6_) *δ* 7.95 (t, *J* = 5.8 Hz, 1H), 7.29–7.12 (m, 5H), 3.34 (s, 2H), 3.00–2.96 (m, 2H), 2.10–2.14 (m, 2H), 1.46–1.39 (m, 2H), 1.36–1.30 (m, 2H), 1.22–1.18 (m, 4H).

8-(2-Phenylacetamido)octanoic acid (**12e**). Using the synthetic method of compound **7a**, compound **11e** gave **12e** as a white solid (67% yield). ^1^H NMR (400 MHz, DMSO-*d*_6_) *δ* 11.92 (s, 1H), 7.95 (t, *J* = 5.6 Hz, 1H), 7.27–7.15 (m, 5H), 3.34 (s, 2H), 3.00–2.96 (m, 2H), 2.14 (t, *J* = 7.3 Hz, 2H), 1.49–1.39 (m, 2H), 1.36–1.30 (m, 2H).

9-(2-Phenylacetamido)nonanoic acid (**12f**). Using the synthetic method of compound **7a**, compound **11f** gave **12f** as a white solid (77% yield). The resulting crude product was directly used for the next reaction.

Tert-butyl-2-(4-(2-phenylacetamido)butanoyl)-1-propylhydrazine-1-carboxylate (**13a**). Using the synthetic method of **8a**, compound **12a** and compound **4** gave **13a** as a yellow oil (77% yield). ^1^H NMR (400 MHz, DMSO-*d*_6_) *δ* 9.88 (s, 1H), 8.05 (s, 1H), 7.22–7.18 (m, 5H), 3.38 (s, 2H), 3.07–3.03 (m, 2H), 2.05 (d, *J* = 7.3 Hz, 2H), 1.67–1.60 (m, 2H), 1.33 (s, 9H), 1.23 (s, 2H), 0.83 (t, *J* = 7.4 Hz, 3H).

Tert-butyl-2-(5-(2-phenylacetamido)pentanoyl)-1-propylhydrazine-1-carboxylate (**13b**). Using the synthetic method of **8a**, compound **12b** and compound **4** gave **13b** as a yellow oil (70% yield). ^1^H NMR (400 MHz, DMSO-*d*_6_) *δ* 9.84 (s, 1H), 8.01 (t, *J* = 5.6 Hz, 1H), 7.32–7.24 (m, 3H), 7.24–7.16 (m, 2H), 3.38 (s, 2H), 3.24 (d, *J* = 10.4 Hz, 2H), 3.05–3.00 (m, 2H), 2.06 (t, *J* = 7.2 Hz, 2H), 1.54–1.48 (m, 2H), 1.45 (d, *J* = 13.8 Hz, 2H), 1.34 (s, 9H), 1.32–1.22 (m, 2H), 0.83 (t, *J* = 7.4 Hz, 3H).

Tert-butyl-(6-(2-phenylacetamido)hexanoyl)-1-propylhydrazine-1-carboxylate (**13c**). Using the synthetic method of **8a**, compound **12c** and compound **4** gave **13c** as a yellow oil (58% yield). The resulting crude product was directly used for the next reaction.

Tert-butyl-2-(7-(2-phenylacetamido)heptanoyl)-1-propylhydrazine-1-carboxylate (**13d**). Using the synthetic method of **8a**, compound **12d** and compound **4** gave **13d** as a yellow oil (43% yield). The resulting crude product was directly used for the next reaction.

Tert-butyl-2-(8-(2-phenylacetamido)octanoyl)-1-propylhydrazine-1-carboxylate (**13e**). Using the synthetic method of **8a**, compound **12e** and compound **4** gave **13e** as a yellow oil (40% yield). ^1^H NMR (400 MHz, CDCl_3_) *δ* 7.39–7.33 (m, 3H), 7.30 (d, *J* = 7.1 Hz, 1H), 5.40 (s, 1H), 3.56 (s, 2H), 3.43 (t, *J* = 7.3 Hz, 2H), 3.22–3.17 (m, 2H), 2.16 (t, *J* = 7.7 Hz, 2H), 1.66–1.61 (m, 2H), 1.57–1.51 (m, 2H), 1.41 (s, 9H), 1.33–1.18 (m, 8H), 0.89 (t, *J* = 7.2 Hz, 3H).

Tert-butyl-2-(9-(2-phenylacetamido)nonanoyl)-1-propylhydrazine-1-carboxylate (**13f**). Using the synthetic method of **8a**, compound **12f** and compound **4** gave **13f** as a yellow oil (39% yield). The resulting crude product was directly used for the next reaction.

N-(4-Oxo-4-(2-propylhydrazineyl)butyl)-2-phenylacetamide (**14a**). Using the synthetic method of **9a**, compound **13a** gave **14a** as a white solid (75% yield). ^1^H NMR (400 MHz, CDCl_3_) *δ* 7.37–7.29 (m, 3H), 7.24 (s, 2H), 5.81 (s, 1H), 3.56 (s, 2H), 3.27–3.22 (m, 2H), 2.76 (t, *J* = 7.1 Hz, 2H), 2.10 (t, *J* = 6.9 Hz, 2H), 1.78–1.72 (m, 2H), 1.44–1.42 (m, 2H), 0.93 (t, *J* = 7.3 Hz, 3H). ^13^C NMR (126 MHz, DMSO-*d*_6_) *δ* 170.46, 136.96, 129.39, 128.64, 126.74, 53.45, 42.86, 38.73, 31.50, 25.85, 21.21, 12.05. HRMS (AP-ESI) *m*/*z*: calcd for C_15_H_23_N_3_O_2_ [M + H]^+^, 278.18630; found, 278.18652. HPLC analysis of compound **14a** (purity: 100.0%).

N-(5-Oxo-5-(2-propylhydrazineyl)pentyl)-2-phenylacetamide (**14b**). Using the synthetic method of **9a**, compound **13b** gave **14b** as a white solid (60% yield). ^1^H NMR (400 MHz, DMSO-*d*_6_) *δ* 9.21 (s, 1H), 8.01 (t, *J* = 5.7 Hz, 1H), 7.33–7.16 (m, 5H), 4.77 (s, 1H), 3.38 (s, 2H), 3.04–3.00 (m, 2H), 2.60 (t, *J* = 7.2 Hz, 2H), 2.00 (t, *J* = 7.3 Hz, 2H), 1.52–1.44 (m, 2H), 1.40–1.33 (m, 4H), 0.86 (t, *J* = 6.7 Hz, 3H). ^13^C NMR (126 MHz, DMSO-*d*_6_) *δ* 129.37, 128.62, 126.71, 53.48, 42.86, 38.82, 33.58, 29.11, 23.19, 21.21, 12.06. HRMS (AP-ESI) *m*/*z*: calcd for C_16_H_23_N_3_O_2_ [M + H]^+^, 292.20195; found, 292.20203. HPLC analysis of compound **14b** (purity: 100.0%).

N-(6-Oxo-6-(2-propylhydrazineyl)hexyl)-2-phenylacetamide (**14c**). Using the synthetic method of **9a**, compound **13c** gave **14c** as a white solid (80% yield). ^1^H NMR (400 MHz, CDCl_3_) *δ* 7.37–7.28 (m, 3H), 7.20 (d, *J* = 31.6 Hz, 2H), 5.51 (d, *J* = 7.7 Hz, 1H), 3.55 (s, 2H), 3.21–3.16 (m, 2H), 2.76 (t, *J* = 7.3 Hz, 2H), 2.09 (t, *J* = 7.4 Hz, 2H), 1.64–1.57 (m, 2H), 1.51–1.40 (m, 4H), 1.30–1.22 (m, 2H), 0.93 (t, *J* = 7.4 Hz, 3H). ^13^C NMR (126 MHz, DMSO-*d*_6_) *δ* 171.35, 170.31, 137.04, 129.37, 128.62, 126.71, 53.22, 38.95, 33.77, 29.26, 26.41, 25.29, 20.74, 11.93. HRMS (AP-ESI) *m*/*z*: calcd for C_18_H_29_N_3_O_2_ [M + Na]^+^, 328.1995; found, 328.1999. HPLC analysis of compound **14c** (purity: 100.0%).

N-(7-Oxo-7-(2-propylhydrazineyl)heptyl)-2-phenylacetamide (**14d**). Using the synthetic method of **9a**, compound **13d** gave **14d** as a white solid (85% yield). ^1^H NMR (400 MHz, CDCl_3_) *δ* 7.38–7.32 (m, 2H), 7.31–7.28 (m, 1H), 7.26–7.22 (m, 2H), 7.19 (s, 1H), 5.45 (s, 1H), 3.55 (s, 2H), 3.21–3.15 (m, 2H), 2.77 (t, *J* = 7.3 Hz, 2H), 2.08 (t, *J* = 7.5 Hz, 2H), 1.62–1.55 (m, 2H), 1.51–1.44 (m, 2H), 1.43–1.37 (m, 2H), 1.34–1.16 (m, 4H), 0.92 (t, *J* = 7.4 Hz, 3H). ^13^C NMR (126 MHz, DMSO-*d*_6_) *δ* 171.34, 170.30, 137.08, 129.35, 128.60, 126.70, 53.47, 42.90, 38.99, 33.89, 29.42, 28.70, 26.54, 25.62, 21.21, 12.05. HRMS (AP-ESI) *m*/*z*: calcd for C_18_H_29_N_3_O_2_ [M + H]^+^, 320.23325; found, 320.23349. HPLC analysis of compound **14d** (purity: 100.0%).

N-(8-Oxo-8-(2-propylhydrazineyl)octyl)-2-phenylacetamide (**14e**). Using the synthetic method of **9a**, compound **13e** gave **14e** as a white solid (70% yield). ^1^H NMR (400 MHz, DMSO-*d*_6_) *δ* 9.20 (s, 1H), 7.98 (t, *J* = 5.7 Hz, 1H), 7.33–7.16 (m, 5H), 4.77 (s, 1H), 3.37 (s, 2H), 3.04–2.99 (m, 2H), 2.60 (t, *J* = 7.1 Hz, 2H), 1.99 (t, *J* = 7.3 Hz, 2H), 1.50–1.43 (m, 2H), 1.40–1.33 (m, 4H), 1.26–1.18 (m, 6H), 0.86 (t, *J* = 7.4 Hz, 3H). ^13^C NMR (126 MHz, DMSO-*d*_6_) *δ* 171.41, 170.31, 137.08, 129.34, 128.60, 126.70, 53.34, 42.91, 39.00, 33.84, 29.49, 28.92, 28.85, 26.71, 25.54, 20.96, 11.98. HRMS (AP-ESI) *m*/*z*: calcd for C_19_H_31_N_3_O_2_ [M + H]^+^, 334.24890; found, 334.24899. HPLC analysis of compound **14e** (purity: 100.0%).

N-(9-Oxo-9-(2-propylhydrazineyl)nonyl)-2-phenylacetamide (**14f**). Using the synthetic method of **9a**, compound **13f** gave **14f** as a white solid (70% yield). ^1^H NMR (400 MHz, DMSO-*d*_6_) *δ* 9.20 (s, 1H), 7.98 (t, *J* = 5.5 Hz, 1H), 7.32–7.17 (m, 5H), 4.77 (s, 1H), 3.37 (s, 2H), 3.04–2.99 (m, 2H), 2.60 (t, *J* = 7.1 Hz, 2H), 1.99 (t, *J* = 7.4 Hz, 2H), 1.50–1.43 (m, 2H), 1.42–1.33 (m, 4H), 1.21 (s, 8H), 0.85 (t, *J* = 7.4 Hz, 3H). ^13^C NMR (126 MHz, DMSO-*d*_6_) *δ* 171.36, 170.30, 137.08, 129.35, 128.60, 126.70, 53.47, 42.91, 39.01, 33.91, 29.50, 29.13, 29.07, 28.93, 26.77, 25.65, 21.21, 12.05. HRMS (AP-ESI) *m*/*z*: calcd for C_20_H_33_N_3_O_2_ [M + H]^+^, 348.26455; found, 348.26477. HPLC analysis of compound **14f** (purity: 100.0%).

Methyl-3-(4-((2,3-dihydro-1H-inden-2-yl)carbamoyl)phenyl)acrylate (**16a**). Using the synthetic method of compound **6a**, 2,3-dihydro-1H-inden-2-amine (**15a**) and 4-(3-methoxy-3-oxoprop-1-en-1-yl)benzoic acid gave **16a** as a pale yellow oil (55% yield). ^1^H NMR (400 MHz, DMSO-*d*_6_) *δ* 8.75 (d, *J* = 7.1 Hz, 1H), 7.90 (d, *J* = 8.1 Hz, 2H), 7.81 (d, *J* = 8.1 Hz, 2H), 7.69 (d, *J* = 16.1 Hz, 1H), 7.24–7.22 (m, 2H), 7.17–7.14 (m, 2H), 6.75 (d, *J* = 16.0 Hz, 1H), 4.73–4.67 (m, 1H), 3.73 (s, 3H), 3.26 (d, *J* = 7.8 Hz, 1H), 3.22 (d, *J* = 7.9 Hz, 1H), 2.99–2.93 (m, 2H).

Methyl-3-(4-(1,2,3,4-tetrahydroisoquinoline-2-carbonyl)phenyl)acrylate (**16b**). Using the synthetic method of compound **6a**, 1,2,3,4-tetrahydroisoquinoline (**15b**) and 4-(3-methoxy-3-oxoprop-1-en-1-yl)benzoic acid gave **16b** as a pale yellow oil (48% yield). ^1^H NMR (400 MHz, CDCl_3_) *δ* 7.70 (d, *J* = 16.0 Hz, 1H), 7.58 (d, *J* = 8.1 Hz, 2H), 7.47 (d, *J* = 7.9 Hz, 2H), 7.21–7.16 (m, 4H), 6.48 (d, *J* = 16.0 Hz, 1H), 4.88 (s, 1H), 4.57 (s, 1H), 3.99 (s, 1H), 3.81 (s, 3H), 3.63 (s, 1H), 2.91 (d, *J* = 29.2 Hz, 2H).

Methyl-3-(4-(6-bromo-1,2,3,4-tetrahydroisoquinoline-2-carbonyl)phenyl)acrylate (**16c**). Using the synthetic method of compound **6a**, 6-bromo-1,2,3,4-tetrahydroisoquinoline (**15c**) and 4-(3-methoxy-3-oxoprop-1-en-1-yl)benzoic acid gave **16c** as a pale yellow oil (45% yield). ^1^H NMR (400 MHz, CDCl_3_) *δ* 7.60 (d, *J* = 16.0 Hz, 1H), 7.50 (d, *J* = 8.0 Hz, 2H), 7.37 (d, *J* = 7.8 Hz, 2H), 7.23 (s, 2H), 6.97 (s, 1H), 6.40 (d, *J* = 16.1 Hz, 1H), 4.58 (d, *J* = 116.4 Hz, 2H), 3.73 (s, 3H), 3.70 (d, *J* = 116.4 Hz, 2H), 2.87–2.74 (m, 2H).

Methyl-3-(4-(7-bromo-1,2,3,4-tetrahydroisoquinoline-2-carbonyl)phenyl)acrylate (**16d**). Using the synthetic method of compound **6a**, 7-bromo-1,2,3,4-tetrahydroisoquinoline (**15d**) and 4-(3-methoxy-3-oxoprop-1-en-1-yl)benzoic acid gave **16d** as a pale yellow oil (46% yield). ^1^H NMR (400 MHz, CDCl_3_) *δ* 7.69 (d, *J* = 16.1 Hz, 1H), 7.57 (d, *J* = 8.2 Hz, 2H), 7.45 (d, *J* = 7.8 Hz, 2H), 7.38–7.23 (m, 2H), 7.02 (d, *J* = 8.4 Hz, 1H), 6.48 (d, *J* = 16.0 Hz, 1H), 4.84 (s, 1H), 4.54 (s, 1H), 3.96 (s, 1H), 3.81 (s, 3H), 3.62 (s, 1H), 2.90 (d, *J* = 31.6 Hz, 2H).

3-(4-((2,3-dihydro-1H-inden-2-yl)carbamoyl)phenyl)acrylic acid (**17a**). Using the synthetic method of compound **7a**, compound **16a** gave **17a** as a white solid (65% yield). ^1^H NMR (400 MHz, DMSO-*d*_6_) *δ* 12.63 (s, 1H), 8.71 (d, *J* = 6.9 Hz, 1H), 7.87 (d, *J* = 8.3 Hz, 2H), 7.73 (d, *J* = 8.2 Hz, 1H), 7.58 (d, *J* = 16.0 Hz, 1H), 7.21–7.10 (m, 4H), 6.59 (d, *J* = 16.0 Hz, 1H), 4.72–4.63 (m, 1H), 3.24–3.18 (m, 2H), 2.96–2.91 (m, 2H).

3-(4-(1,2,3,4-tetrahydroisoquinoline-2-carbonyl)phenyl)acrylic acid (**17b**). Using the synthetic method of compound **7a**, compound **16b** gave **17b** as a white solid (50% yield). ^1^H NMR (400 MHz, DMSO-*d*_6_) *δ* 12.50 (s, 1H), 7.74 (d, *J* = 7.9 Hz, 3H), 7.60 (d, *J* = 16.0 Hz, 1H), 7.46 (s, 2H), 7.21 (s, 1H), 7.16 (d, *J* = 9.8 Hz, 3H), 6.57 (d, *J* = 16.0 Hz, 1H), 4.73 (s, 1H), 3.81 (s, 1H), 2.82 (s, 2H). ESI-MS *m*/*z*: 307.87 [M + H]^+^.

3-(4-(6-bromo-1,2,3,4-tetrahydroisoquinoline-2-carbonyl)phenyl)acrylic acid (**17c**). Using the synthetic method of compound **7a**, compound **16c** gave **17c** as a white solid (43% yield). ^1^H NMR (400 MHz, DMSO-*d*_6_) *δ* 7.72 (d, *J* = 8.2 Hz, 2H), 7.58–7.53 (m, 1H), 7.43 (d, *J* = 7.7 Hz, 2H), 7.38 (s, 2H), 7.21 (s, 1H), 6.58 (d, *J* = 16.0 Hz, 1H), 4.68 (s, 1H), 4.49 (s, 1H), 3.78 (s, 1H), 3.50 (s, 1H), 2.82 (s, 2H).

3-(4-(7-bromo-1,2,3,4-tetrahydroisoquinoline-2-carbonyl)phenyl)acrylic acid (**17d**). Using the synthetic method of compound **7a**, compound **16d** gave **17d** as a white solid (38% yield). ^1^H NMR (400 MHz, DMSO-*d*_6_) *δ* 7.74 (d, *J* = 8.0 Hz, 2H), 7.60 (d, *J* = 16.0 Hz, 1H), 7.44 (d, *J* = 6.5 Hz, 2H), 7.38 (s, 2H), 7.21 (s, 1H), 6.57 (d, *J* = 16.0 Hz, 1H), 4.69 (s, 1H), 4.49 (s, 1H), 3.78 (s, 1H), 3.50 (s, 1H), 2.83 (s, 2H). ESI-MS *m*/*z*: 385.75 [M + H]^+^.

Tert-butyl-2-(3-(4-((2,3-dihydro-1H-inden-2-yl)carbamoyl)phenyl)acryloyl)-1-propylhydrazine-1-carboxylate (**18a**). Using the synthetic method of **8a**, compound **17a** and compound **4** gave **18a** as a pale yellow solid (51% yield).

Tert-butyl-1-propyl-2-(3-(4-(1,2,3,4-tetrahydroisoquinoline-2-carbonyl)phenyl)acryloyl)hydrazine-1-carboxylate (**18b**). Using the synthetic method of **8a**, compound **17b** and compound **4** gave **18b** as a pale yellow solid (44% yield). ^1^H NMR (400 MHz, DMSO-*d*_6_) *δ* 10.21 (s, 1H), 7.87–7.76 (m, 1H), 7.69 (d, *J* = 8.2 Hz, 2H), 7.59 (d, *J* = 15.9 Hz, 1H), 7.50 (d, *J* = 7.5 Hz, 2H), 7.18 (s, 3H), 6.66 (d, *J* = 15.9 Hz, 1H), 3.36 (d, *J* = 4.7 Hz, 2H), 2.86 (s, 2H), 2.69 (s, 2H), 1.50 (t, *J* = 7.3 Hz, 2H), 1.37 (s, 9H), 1.18 (d, *J* = 7.1 Hz, 2H), 0.87 (t, *J* = 7.4 Hz, 3H).

Tert-butyl-2-(3-(4-(6-bromo-1,2,3,4-tetrahydroisoquinoline-2-carbonyl)phenyl)acryloyl)-1-propylhydrazine-1-carboxylate (**18c**). Using the synthetic method of **8a**, compound **17c** and compound **4** gave **18c** as a pale yellow solid (35% yield). ^1^H NMR (400 MHz, DMSO-*d*_6_) *δ* 10.18 (s, 1H), 7.65 (d, *J* = 8.1 Hz, 2H), 7.56 (d, *J* = 15.9 Hz, 1H), 7.46 (d, *J* = 7.7 Hz, 2H), 7.38 (s, 2H), 7.23 (s, 1H), 6.63 (d, *J* = 15.9 Hz, 1H), 4.69 (s, 1H), 4.50 (s, 1H), 3.79 (s, 1H), 3.51 (s, 1H), 3.32 (t, *J* = 7.7 Hz, 2H), 2.83 (s, 2H), 1.48–1.34 (m, 2H), 1.34 (s, 9H), 0.83 (t, *J* = 7.4 Hz, 3H).

Tert-butyl-2-(3-(4-(7-bromo-1,2,3,4-tetrahydroisoquinoline-2-carbonyl)phenyl)acryloyl)-1-propylhydrazine-1-carboxylate (**18d**). Using the synthetic method of **8a**, compound **17d** and compound **4** gave **18d** as a pale yellow solid (34% yield). ^1^H NMR (400 MHz, CDCl_3_) *δ* 7.58 (d, *J* = 7.7 Hz, 1H), 7.44 (d, *J* = 24.9 Hz, 5H), 7.32 (s, 2H), 7.07 (s, 1H), 6.45 (d, *J* = 15.7 Hz, 1H), 4.81 (s, 1H), 4.52 (s, 1H), 3.95 (s, 1H), 3.62 (s, 1H), 3.52 (t, *J* = 7.2 Hz, 2H), 2.86 (s, 2H), 1.64–1.48 (m, 2H), 1.48 (s, 9H), 0.92 (t, *J* = 7.4 Hz, 3H).

N-(2,3-Dihydro-1H-inden-2-yl)-4-(3-oxo-3-(2-propylhydrazineyl)prop-1-en-1-yl)benzamide (**19a**). Using the synthetic method of **9a**, compound **18a** gave **19a** as a white solid (43% yield). ^1^H NMR (400 MHz, DMSO-*d*_6_) *δ* 9.65 (s, 1H), 8.71 (d, *J* = 7.0 Hz, 1H), 7.89 (d, *J* = 8.2 Hz, 2H), 7.63 (d, *J* = 8.3 Hz, 2H), 7.47 (d, *J* = 15.8 Hz, 1H), 7.24–7.21 (m, 2H), 7.18–7.11 (m, 2H), 6.62 (d, *J* = 15.8 Hz, 1H), 5.08 (s, 1H), 4.75–4.66 (m, 1H), 3.27–3.21 (m, 2H), 2.99–2.93 (m, 2H), 2.69 (t, *J* = 7.2 Hz, 2H), 1.48–1.39 (m, 2H), 0.88 (t, *J* = 7.4 Hz, 3H). ^13^C NMR (126 MHz, DMSO-*d*_6_) *δ* 166.13, 163.94, 141.72, 137.97, 137.89, 135.38, 128.41, 127.70, 126.87, 124.91, 122.28, 53.48, 51.08, 39.30, 21.22, 12.03. HRMS (AP-ESI) *m*/*z*: calcd for C_22_H_26_O_2_N_3_ [M + H]^+^, 364.20195; found, 364.20175. HPLC analysis of compound **19a** (purity: 95.70%).

N′-Propyl-3-(4-(1,2,3,4-tetrahydroisoquinoline-2-carbonyl)phenyl)acrylohydrazide (**19b**). Using the synthetic method of **9a**, compound **18b** gave **19b** as a white solid (38% yield). ^1^H NMR (400 MHz, DMSO-*d*_6_) *δ* 9.72 (s, 1H), 7.64 (d, *J* = 8.3 Hz, 2H), 7.53–7.46 (m, 3H), 7.31–7.08 (m, 4H), 6.64 (d, *J* = 15.9 Hz, 1H), 4.76 (s, 1H), 4.57 (s, 1H), 3.84 (s, 1H), 3.56 (s, 1H), 2.85 (s, 2H), 2.70 (t, *J* = 7.1 Hz, 2H), 1.49–1.39 (m, 2H), 0.89 (t, *J* = 7.4 Hz, 3H). ^13^C NMR (126 MHz, DMSO-*d*_6_) *δ* 164.01, 138.03, 127.97, 121.98, 53.51, 21.25, 12.03. HRMS (AP-ESI) *m*/*z*: calcd for C_22_H_25_N_3_O_2_ [M + H]^+^, 364.20195; found, 364.20215. HPLC analysis of compound **19b** (purity: 98.25%).

3-(4-(6-Bromo-1,2,3,4-tetrahydroisoquinoline-2-carbonyl)phenyl)-N′-propylacrylohydrazide (**19c**). Using the synthetic method of **9a**, compound **18c** gave **19c** as a pale yellow solid (40% yield). ^1^H NMR (400 MHz, DMSO-*d*_6_) *δ* 9.68 (s, 1H), 7.64 (d, *J* = 8.0 Hz, 2H), 7.54–7.44 (m, 3H), 7.42 (d, *J* = 1.9 Hz, 2H), 7.25 (s, 1H), 6.61 (d, *J* = 15.9 Hz, 1H), 5.10 (s, 1H), 4.72 (s, 1H), 4.54 (s, 1H), 3.82 (s, 1H), 3.54 (s, 1H), 2.86 (s, 2H), 2.70 (t, *J* = 7.1 Hz, 2H), 1.48–1.39 (m, 2H), 0.89 (t, *J* = 7.4 Hz, 3H). ^13^C NMR (126 MHz, DMSO-*d*_6_) *δ* 164.00, 138.06, 132.97, 128.75, 127.98, 53.50, 12.03. HRMS (AP-ESI) *m*/*z*: calcd for C_22_H_25_O_2_N_3_Br [M + H]^+^, 442.11247; found, 442.11209. HPLC analysis of compound **19c** (purity: 99.27%).

3-(4-(7-Bromo-1,2,3,4-tetrahydroisoquinoline-2-carbonyl)phenyl)-N′-propylacrylohydrazide (**19d**). Using the synthetic method of **9a**, compound **18d** gave **19d** as a pale yellow solid (39% yield). ^1^H NMR (400 MHz, DMSO-*d*_6_) *δ* 10.86 (s, 1H), 7.72 (d, *J* = 7.9 Hz, 2H), 7.65 (d, *J* = 15.9 Hz, 1H), 7.51 (d, *J* = 7.8 Hz, 2H), 7.42 (d, *J* = 1.9 Hz, 2H), 7.25 (s, 1H), 6.68 (d, *J* = 15.9 Hz, 1H), 4.73 (s, 1H), 4.53 (s, 1H), 3.82 (s, 1H), 3.53 (s, 1H), 2.97 (t, *J* = 7.6 Hz, 2H), 2.87 (d, *J* = 9.6 Hz, 2H), 1.61–1.52 (m, 2H), 0.92 (t, *J* = 7.4 Hz, 3H). ^13^C NMR (126 MHz, DMSO-*d*_6_) *δ* 164.00, 138.06, 132.97, 128.75, 53.50. HRMS (AP-ESI) *m*/*z*: calcd for C_22_H_25_O_2_N_3_Br [M + H]^+^, 442.11247; found, 442.11215. HPLC analysis of compound **19d** (purity: 99.12%).

Methyl-3-(4-((phenylamino)methyl)phenyl)acrylate (**21a**). Aniline (**20a**, 81 mg, 0.755 mmol) was dissolved in DMF (1.5 mL) followed by the addition of K_2_CO_3_ (104 mg, 0.755 mmol); the mixed solution was refluxed at 80 °C for 0.5 h. Methyl-3-(4-(bromomethyl)phenyl)acrylate (150 mg, 0.378 mmol) was added dropwise during the refluxing at 80 °C and stirred for 6 h, and the reaction was monitored by TLC (CH_2_Cl_2_/MeOH 15:1). Then, the reaction solution was diluted with AcOEt, and the mixture was washed with 1 N HCl, saturated NaHCO_3_, and brine three times. The organic layer was dried over anhydrous Na_2_SO_4_ and evaporated under vacuum. The crude product was purified by chromatography using DCM/methanol (50:1) as mobile phase to obtain **21a** as a white solid (134mg, yield 65%). ^1^H NMR (400 MHz, CDCl_3_) *δ* 7.69 (d, *J* = 16.0 Hz, 1H), 7.53–7.47 (m, 2H), 7.43–7.36 (m, 2H), 7.22–7.14 (m, 2H), 6.75–6.71 (m, 1H), 6.64–6.60 (m, 2H), 6.43 (d, *J* = 16.0 Hz, 1H), 4.37 (s, 2H), 3.81 (s, 3H).

Methyl-3-(4-((benzylamino)methyl)phenyl)acrylate (**21b**). Using the synthetic method of **21a**, phenylmethanamine (**20b**) and methyl-3-(4-(bromomethyl)phenyl)acrylate gave **21b** as a pale yellow solid (42% yield). ^1^H NMR (400 MHz, DMSO-*d*_6_) *δ* 7.67 (d, *J* = 2.3 Hz, 1H), 7.64 (d, *J* = 10.3 Hz, 2H), 7.39 (d, *J* = 8.0 Hz, 2H), 7.36–7.32 (m, 3H), 7.31 (d, *J* = 1.0 Hz, 1H), 7.25–7.19 (m, 1H), 6.61 (d, *J* = 16.1 Hz, 1H), 3.72 (s, 3H), 3.69 (s, 2H), 3.66 (s, 2H).

Methyl-3-(4-((phenethylamino)methyl)phenyl)acrylate (**21c**). Using the synthetic method of **21a**, 2-phenylethan-1-amine (**20c**) and methyl-3-(4-(bromomethyl)phenyl)acrylate gave **21c** as a pale yellow solid (42% yield). ^1^H NMR (400 MHz, DMSO-*d*_6_) *δ* 7.67–7.61 (m, 3H), 7.35 (d, *J* = 8.2 Hz, 2H), 7.29–7.23 (m, 2H), 7.21–7.13 (m, 3H), 6.59 (d, *J* = 16.1 Hz, 1H), 3.74 (s, 2H), 3.72 (s, 3H), 2.71 (d, *J* = 2.2 Hz, 4H).

Methyl-3-(4-(((pyridin-2-ylmethyl)amino)methyl)phenyl)acrylate (**21d**). Using the synthetic method of **21a**, pyridin-2-ylmethanamine (**20d**) and methyl-3-(4-(bromomethyl)phenyl)acrylate gave **21d** as a pale yellow solid (42% yield). ^1^H NMR (400 MHz, DMSO-*d*_6_) *δ* 8.46 (s, 1H), 7.74–7.69 (m, 1H), 7.67–7.58 (m, 3H), 7.42 (d, *J* = 8.2 Hz, 1H), 7.36 (d, *J* = 8.1 Hz, 2H), 7.20 (s, 1H), 6.58 (d, *J* = 16.1 Hz, 1H), 3.73 (s, 2H), 3.71 (s, 2H), 3.68 (s, 3H). ESI-MS *m*/*z*: 282.91 [M + H]^+^.

Methyl 3-(4-(((2,3-dihydro-1H-inden-2-yl)amino)methyl)phenyl)acrylate (**21e**). Using the synthetic method of **21a**, 2,3-dihydro-1H-inden-2-amine (**20e**) and methyl-3-(4-(bromomethyl)phenyl)acrylate gave **21e** as a pale yellow solid (56% yield). ^1^H NMR (400 MHz, DMSO-*d*_6_) *δ* 7.71–7.58 (m, 3H), 7.40 (d, *J* = 8.0 Hz, 2H), 7.17–7.14 (m, 2H), 7.12–7.05 (m, 2H), 6.61 (d, *J* = 16.1 Hz, 1H), 3.77 (s, 2H), 3.72 (s, 3H), 3.51–3.44 (m, 1H), 3.06–3.00 (m, 2H), 2.73–2.67 (m, 2H).

Methyl-3-(4-((naphthalen-1-ylamino)methyl)phenyl)acrylate (**21f**). Using the synthetic method of **21a**, naphthalen-1-amine (**20f**) and methyl-3-(4-(bromomethyl)phenyl)acrylate gave **21f** as a pale yellow solid (45% yield). ^1^H NMR (400 MHz, DMSO-*d*_6_) *δ* 8.29–8.21 (m, 1H), 7.78–7.72 (m, 1H), 7.65 (d, *J* = 7.6 Hz, 2H), 7.48–7.39 (m, 4H), 7.15 (t, *J* = 7.8 Hz, 1H), 7.07 (d, *J* = 8.1 Hz, 1H), 7.00 (t, *J* = 6.0 Hz, 1H), 6.58 (d, *J* = 16.1 Hz, 1H), 6.33 (d, *J* = 7.6 Hz, 1H), 4.53 (d, *J* = 5.9 Hz, 2H), 3.71 (s, 3H). ESI-MS *m*/*z*: 317.90 [M + H]^+^.

Methyl-3-(4-(((naphthalen-1-ylmethyl)amino)methyl)phenyl)acrylate (**21g**). Using the synthetic method of **21a**, naphthalen-1-ylmethanamine (**20g**) and methyl-3-(4-(bromomethyl)phenyl)acrylate gave **21g** as a pale yellow solid (47% yield). ^1^H NMR (400 MHz, DMSO-*d*_6_) *δ* 8.18–8.10 (m, 1H), 7.93–7.89 (m, 1H), 7.81 (d, *J* = 8.1 Hz, 1H), 7.69–7.64 (m, 3H), 7.54–7.49 (m, 3H), 7.47–7.42 (m, 3H), 6.62 (d, *J* = 16.0 Hz, 1H), 4.12 (s, 2H), 3.82 (s, 2H), 3.72 (s, 3H). ESI-MS *m*/*z*: 331.81 [M + H]^+^.

Methyl-3-(4-(((tert-butoxycarbonyl)(phenyl)amino)methyl)phenyl)acrylate (**22a**). Compound **21a** (120 mg, 0.18 mmol) was dissolved in 2.5 mL of DCM, which was added to 147 mg of (Boc)_2_O and 188 μL of TEA. The mixture was stirred overnight, and DCM was condensed under vacuum to obtain **22a** as a yellow solid (90% yield). ^1^H NMR (400 MHz, DMSO-*d*_6_) *δ* 7.69–7.64 (m, 2H), 7.62 (d, *J* = 16.1 Hz, 1H), 7.32–7.26 (m, 2H), 7.24 (d, *J* = 8.0 Hz, 2H), 7.22–7.18 (m, 2H), 7.17–7.12 (m, 1H), 6.60 (d, *J* = 16.0 Hz, 1H), 4.86 (s, 2H), 3.71 (s, 3H), 1.37 (s, 9H). ESI-MS *m*/*z*: 369.18 [M + H]^+^.

Methyl-3-(4-((benzyl(tert-butoxycarbonyl)amino)methyl)phenyl)acrylate (**22b**). Using the synthetic method of **22a**, compound **21b** gave **22b** as a white solid (92% yield). The resulting crude product was directly used for the next reaction.

Methyl-3-(4-(((tert-butoxycarbonyl)(phenethyl)amino)methyl)phenyl)acrylate (**22c**). Using the synthetic method of **22a**, compound **21c** gave **22c** as a white solid (95% yield). ESI-MS *m*/*z*: 396.17 [M + H]^+^.

Methyl-3-(4-(((tert-butoxycarbonyl)(pyridin-2-ylmethyl)amino)methyl)phenyl)acrylate (**22d**). Using the synthetic method of **22a**, compound **21d** gave **22d** as a white solid (95% yield). ^1^H NMR (400 MHz, CDCl_3_) *δ* 8.51 (d, *J* = 4.8 Hz, 1H), 7.69–7.61 (m, 2H), 7.46 (d, *J* = 8.1 Hz, 2H), 7.28 (d, *J* = 11.5 Hz, 2H), 7.23 (s, 1H), 7.17–7.14 (m, 2H), 6.41 (d, *J* = 16.0 Hz, 1H), 4.55 (s, 2H), 4.46 (s, 2H), 3.80 (s, 3H), 1.26 (s, 9H).

Methyl)-3-(4-(((tert-butoxycarbonyl)(2,3-dihydro-1H-inden-2-yl)amino)methyl)phenyl)acrylate (**22e**). Using the synthetic method of **22a**, compound **21e** gave **22e** as a white solid (93% yield). ^1^H NMR (400 MHz, DMSO-*d*_6_) *δ* 7.70–7.63 (m, 3H), 7.25 (d, *J* = 8.0 Hz, 2H), 7.16–7.08 (m, 4H), 6.62 (d, *J* = 16.0 Hz, 1H), 4.47 (s, 2H), 3.72 (s, 3H), 2.97 (d, *J* = 8.4 Hz, 4H), 1.33 (s, 9H).

Methyl-3-(4-(((tert-butoxycarbonyl)(naphthalen-1-yl)amino)methyl)phenyl)acrylate (**22f**). Using the synthetic method of **22a**, compound **21f** gave **22f** as a white solid (94% yield). The resulting crude product was directly used for the next reaction.

Methyl-3-(4-(((tert-butoxycarbonyl)(naphthalen-1-ylmethyl)amino)methyl)phenyl)acry late (**22g**). Using the synthetic method of **22a**, compound **21g** gave **22g** as a white solid (95% yield). ^1^H NMR (400 MHz, DMSO-*d*_6_) *δ* 8.10 (s, 1H), 7.98–7.93 (m, 1H), 7.86 (d, *J* = 8.2 Hz, 1H), 7.69–7.60 (m, 3H), 7.56–7.53 (m, 2H), 7.51–7.44 (m, 1H), 7.30 (s, 1H), 7.22 (d, *J* = 6.8 Hz, 2H), 6.61 (d, *J* = 16.0 Hz, 1H), 4.88 (s, 2H), 4.34 (s, 2H), 3.72 (s, 3H), 1.42 (s, 9H).

3-(4-(((Tert-butoxycarbonyl)(phenyl)amino)methyl)phenyl)acrylic acid (**23a**). Using the synthetic method of compound **7a**, compound **22a** gave **23a** as a white solid (65% yield). ^1^H NMR (400 MHz, DMSO-*d*_6_) *δ* 7.66–7.55 (m, 3H), 7.51 (d, *J* = 16.0 Hz, 1H), 7.28–7.23 (m, 2H), 7.22–7.15 (m, 4H), 7.14–7.09 (m, 1H), 6.44 (d, *J* = 16.0 Hz, 1H), 4.82 (s, 2H), 1.33 (s, 9H). ESI-MS *m*/*z*: 367.85 [M + H]^+^.

3-(4-((Benzyl(tert-butoxycarbonyl)amino)methyl)phenyl)acrylic acid (**23b**). Using the synthetic method of compound **7a**, compound **22b** gave **23b** as a white solid (71% yield). ^1^H NMR (400 MHz, DMSO-*d*_6_) *δ* 12.31 (s, 1H), 7.62 (d, *J* = 8.2 Hz, 2H), 7.54 (d, *J* = 16.0 Hz, 1H), 7.33–7.29 (m, 2H), 7.25–7.17 (m, 5H), 6.47 (d, *J* = 16.0 Hz, 1H), 4.35 (s, 2H), 4.29 (s, 2H), 1.35 (s, 9H).

3-(4-((Phenethylamino)methyl)phenyl)acrylic acid (**23c**). Using the synthetic method of compound **7a**, compound **22c** gave **23c** as a white solid (70% yield). ^1^H NMR (400 MHz, DMSO-*d*_6_) *δ* 7.53 (d, *J* = 8.1 Hz, 2H), 7.34 (d, *J* = 15.9 Hz, 1H), 7.25–7.11 (m, 7H), 6.45 (d, *J* = 16.0 Hz, 1H), 4.33 (s, 2H), 3.28 (s, 2H), 2.70 (t, *J* = 7.1 Hz, 2H), 1.32 (s, 9H).

3-(4-(((Tert-butoxycarbonyl)(pyridin-2-ylmethyl)amino)methyl)phenyl)acrylicacid (**23d**). Using the synthetic method of compound **7a**, compound **22d** gave **23d** as a white solid (72% yield). ^1^H NMR (400 MHz, DMSO-*d*_6_) *δ* 8.55 (d, *J* = 4.1 Hz, 1H), 7.92 (t, *J* = 7.8 Hz, 1H), 7.62 (d, *J* = 8.2 Hz, 2H), 7.54 (d, *J* = 16.0 Hz, 1H), 7.44–7.23 (m, 4H), 6.48 (d, *J* = 16.0 Hz, 1H), 4.51 (s, 2H), 4.45 (s, 2H), 1.26 (s, 9H).

3-(4-(((Tert-butoxycarbonyl)(2,3-dihydro-1H-inden-2-yl)amino)methyl)phenyl)acrylicacid (**23e**). Using the synthetic method of compound **7a**, compound **22e** gave **23e** as a white solid (63% yield). ^1^H NMR (400 MHz, DMSO-*d*_6_) *δ* 7.51 (d, *J* = 7.9 Hz, 2H), 7.30 (d, *J* = 15.9 Hz, 1H), 7.17 (d, *J* = 7.8 Hz, 2H), 7.11–7.09 (m, 2H), 7.08–7.04 (m, 2H), 6.45 (d, *J* = 15.9 Hz, 1H), 4.66 (s, 1H), 4.41 (s, 2H), 2.93 (d, *J* = 8.5 Hz, 4H), 1.29 (s, 9H).

3-(4-(((Tert-butoxycarbonyl)(naphthalen-1-yl)amino)methyl)phenyl)acrylic acid (**23f**). Using the synthetic method of compound **7a**, compound **22f** gave **23f** as a white solid (59% yield). The resulting crude product was directly used for the next reaction.

3-(4-(((Tert-butoxycarbonyl)(naphthalen-1-ylmethyl)amino)methyl)phenyl)acrylic acid (**23g**). Using the synthetic method of compound **7a**, compound **22g** gave **23g** as a white solid (65% yield). ^1^H NMR (400 MHz, DMSO-*d*_6_) *δ* 8.07 (s, 1H), 7.94–7.89 (m, 1H), 7.83 (d, *J* = 8.2 Hz, 1H), 7.65–7.53 (m, 3H), 7.52–7.48 (m, 2H), 7.47–7.42 (m, 1H), 7.26 (s, 1H), 7.18 (d, *J* = 6.6 Hz, 2H), 6.52 (dd, *J* = 44.7, 16.0 Hz, 1H), 4.85 (s, 2H), 4.28 (s, 2H), 1.37 (s, 9H). ESI-MS *m*/*z*: 439.90 [M + Na]^+^.

Tert-butyl-2-(3-(4-(((tert-butoxycarbonyl)(phenyl)amino)methyl)phenyl)acryloyl)-1-pr-opylhydrazine-1-carboxylate (**24a**). Using the synthetic method of **8a**, compound **23a** and compound **4** gave **24a** as a pale yellow solid (41% yield). ^1^H NMR (400 MHz, DMSO-*d*_6_) *δ* 10.11 (s, 1H), 7.51 (d, *J* = 8.0 Hz, 2H), 7.46 (d, *J* = 15.6 Hz, 1H), 7.26 (t, *J* = 7.7 Hz, 2H), 7.22 (d, *J* = 7.9 Hz, 2H), 7.17 (d, *J* = 7.9 Hz, 2H), 7.14–7.09 (m, 1H), 6.51 (d, *J* = 15.9 Hz, 1H), 4.82 (s, 2H), 1.46–1.41 (m, 4H), 1.34 (s, 18H), 0.82 (t, *J* = 7.3 Hz, 3H).

Tert-butyl-2-(3-(4-((benzyl(tert-butoxycarbonyl)amino)methyl)phenyl)acryloyl)-1-propylhydrazine-1-carboxylate (**24b**). Using the synthetic method of **8a**, compound **23b** and compound **4** gave **24b** as a pale yellow solid (42% yield). ^1^H NMR (400 MHz, DMSO-*d*_6_) *δ* 10.15 (s, 1H), 7.59–7.54 (m, 2H), 7.34 (t, *J* = 6.9 Hz, 2H), 7.29–7.19 (m, 6H), 6.61–6.55 (m, 1H), 2.89 (d, *J* = 6.0 Hz, 2H), 2.73 (d, *J* = 6.0 Hz, 2H), 1.52–1.46 (m, 6.8 Hz, 4H), 1.39–1.38 (m, 18H), 0.86 (t, *J* = 7.1 Hz, 3H).

Tert-butyl-2-(3-(4-((phenethylamino)methyl)phenyl)acryloyl)-1-propylhydrazine-1-car-boxylate (**24c**). Using the synthetic method of **8a**, compound **23c** and compound **4** gave **24c** as a pale yellow solid (60% yield). ^1^H NMR (400 MHz, CDCl_3_) *δ* 7.66 (d, *J* = 15.5 Hz, 1H), 7.46 (d, *J* = 8.2 Hz, 2H), 7.29–7.26 (m, 2H), 7.25 (d, *J* = 1.4 Hz, 1H), 7.19 (t, *J* = 7.4 Hz, 4H), 6.39 (d, *J* = 15.6 Hz, 1H), 4.37 (s, 2H), 3.51 (t, *J* = 7.2 Hz, 2H), 3.35–3.29 (m, 2H), 1.63–1.56 (m, 4H), 1.47 (s, 18H), 0.91 (s, 3H).

Tert-butyl-2-(3-(4-(((tert-butoxycarbonyl)(pyridin-2-ylmethyl)amino)methyl)phenyl)ac-ryloyl)-1-propylhydrazine-1-carboxylate (**24d**). Using the synthetic method of **8a**, compound **23d** and compound **4** gave **24d** as a pale yellow solid (61% yield). ^1^H NMR (400 MHz, DMSO-*d*_6_) *δ* 10.15 (s, 1H), 8.51 (d, *J* = 4.4 Hz, 1H), 8.02–7.87 (m, 2H), 7.79–7.74 (m, 1H), 7.53 (d, *J* = 23.1 Hz, 2H), 7.29–7.25 (m, 3H), 6.57 (d, *J* = 15.8 Hz, 1H), 4.50 (d, *J* = 18.3 Hz, 2H), 4.43 (d, *J* = 19.9 Hz, 2H), 3.20 (t, *J* = 7.0 Hz, 2H), 1.52–1.45 (m, 2H), 1.43 (s, 9H), 1.40 (s, 9H), 0.86 (t, *J* = 7.4 Hz, 3H).

Tert-butyl-2-(3-(4-(((tert-butoxycarbonyl)(2,3-dihydro-1H-inden-2-yl)amino)methyl)ph-enyl)acryloyl)-1-propylhydrazine-1-carboxylate (**24e**). Using the synthetic method of **8a**, compound **23e** and compound **4** gave **24e** as a pale yellow solid (60% yield). The resulting crude product was directly used in the next step of the reaction.

Tert-butyl-2-(3-(4-(((tert-butoxycarbonyl)(naphthalen-1-yl)amino)methyl)phenyl)acryloyl)-1-propylhydrazine-1-carboxylate (**24f**). Using the synthetic method of **8a**, compound **23f** and compound **4** gave **24f** as a pale yellow solid (58% yield). ESI-MS *m*/*z*: 459.87 [M + H − 100]^+^.

Tert-butyl-2-(3-(4-(((tert-butoxycarbonyl)(naphthalen-1-ylmethyl)amino)methyl)phen-yl)acryloyl)-1-propylhydrazine-1-carboxylate (**24g**). Using the synthetic method of **8a**, compound **23g** and compound **4** gave **24g** as a pale yellow solid (60% yield). ^1^H NMR (400 MHz, DMSO-*d*_6_) *δ* 10.14 (s, 1H), 8.08 (s, 1H), 7.97–7.91 (m, 1H), 7.87 (d, *J* = 8.2 Hz, 1H), 7.56–7.52 (m, 4H), 7.52–7.45 (m, 2H), 7.32 (s, 1H), 7.23 (d, *J* = 7.2 Hz, 2H), 6.57 (d, *J* = 15.8 Hz, 1H), 4.89 (s, 2H), 4.32 (s, 2H), 1.41 (s, 18H), 0.86 (t, *J* = 7.4 Hz, 3H).

3-(4-((Phenylamino)methyl)phenyl)-N′-propylacrylohydrazide (**25a**). Using the synthetic method of **9a**, compound **24a** gave **25a** as a white solid (58% yield). ^1^H NMR (400 MHz, DMSO-*d*_6_) *δ* 11.28 (s, 1H), 7.64 (s, 1H), 7.59 (s, 2H), 7.42 (d, *J* = 7.9 Hz, 2H), 7.07–7.01 (m, 2H), 6.65–6.42 (m, 4H), 4.31 (s, 2H), 3.06 (s, 2H), 1.61 (s, 2H), 0.93 (t, *J* = 6.3 Hz, 3H). ^13^C NMR (126 MHz, DMSO-*d*_6_) *δ* 148.46, 143.46, 132.91, 129.34, 128.63, 128.29, 116.75, 113.11, 11.24. HRMS (AP-ESI) *m*/*z*: calcd for C_19_H_24_ON_3_ [M + H]^+^, 310.19107; found, 310.19139. HPLC analysis of compound **25a** (purity: 99.77%).

3-(4-((Benzylamino)methyl)phenyl)-N′-propylacrylohydrazide (**25b**). Using the synthetic method of **9a**, compound **24b** gave **25b** as a white solid (58% yield). ^1^H NMR (400 MHz, DMSO-*d*_6_) *δ* 10.87 (s, 1H), 9.43 (s, 2H), 7.69 (d, *J* = 8.0 Hz, 2H), 7.60 (d, *J* = 15.9 Hz, 1H), 7.54 (d, *J* = 7.9 Hz, 2H), 7.51–7.48 (m, 2H), 7.46–7.42 (m, 3H), 6.70 (d, *J* = 15.9 Hz, 1H), 4.21 (s, 4H), 2.94 (t, *J* = 7.4 Hz, 2H), 1.60–1.51 (m, 2H), 0.91 (t, *J* = 7.4 Hz, 3H). ^13^C NMR (126 MHz, DMSO-*d*_6_) *δ* 140.93, 135.31, 134.12, 131.06, 130.45, 129.46, 129.15, 128.53, 119.50, 52.27, 50.52, 50.10, 18.90, 11.43. HRMS (AP-ESI) *m*/*z*: calcd for C_20_H_26_ON_3_ [M + H]^+^, 324.20679; found, 324.20704. HPLC analysis of compound **25b** (purity: 100.0%).

3-(4-((Phenethylamino)methyl)phenyl)-N′-propylacrylohydrazide (**25c**). Using the synthetic method of **9a**, compound **24c** gave **25c** as a white solid (65% yield). ^1^H NMR (400 MHz, DMSO-*d*_6_) *δ* 10.87 (s, 1H), 9.15 (s, 2H), 7.70 (d, *J* = 8.0 Hz, 2H), 7.64–7.52 (m, 3H), 7.34 (t, *J* = 7.2 Hz, 2H), 7.28–7.24 (m, 3H), 6.70 (d, *J* = 15.9 Hz, 1H), 4.23 (t, *J* = 5.4 Hz, 2H), 3.16 (s, 2H), 2.95 (t, *J* = 8.7 Hz, 4H), 1.60–1.51 (m, 2H), 0.91 (t, *J* = 7.4 Hz, 3H). ^13^C NMR (126 MHz, DMSO-*d*_6_) δ 164.07, 138.22, 137.77, 135.82, 130.79, 129.12, 129.08, 128.17, 127.23, 121.51, 53.50, 50.40, 48.32, 32.34, 21.23, 12.02. HRMS (AP-ESI) *m*/*z*: calcd for C_21_H_28_ON_3_ [M + H]^+^, 338.22269; found, 338.22235. HPLC analysis of compound **25c** (purity: 100.0%).

N′-Propyl-3-(4-(((pyridin-2-ylmethyl)amino)methyl)phenyl)acrylohydrazide (**25d**). Using the synthetic method of **9a**, compound **24d** gave **25d** as a white solid (80% yield). ^1^H NMR (400 MHz, DMSO-*d*_6_) *δ* 9.61 (s, 1H), 8.51 (d, *J* = 3.7 Hz, 1H), 7.81–7.76 (m, 1H), 7.52 (d, *J* = 8.2 Hz, 2H), 7.48 (s, 1H), 7.46 (d, *J* = 3.9 Hz, 1H), 7.44–7.38 (m, 3H), 7.30–7.26 (m, 1H), 6.53 (d, *J* = 15.9 Hz, 1H), 3.85 (s, 2H), 3.81 (s, 2H), 2.69 (t, *J* = 7.1 Hz, 2H), 1.47–1.38 (m, 2H), 0.88 (t, *J* = 7.4 Hz, 3H). ^13^C NMR (126 MHz, DMSO-*d*_6_) *δ* 164.13, 152.33, 149.49, 138.63, 137.80, 133.40, 131.16, 128.19, 124.10, 123.76, 121.29, 53.30, 50.23, 50.15, 20.83, 11.89. HRMS (AP-ESI) *m*/*z*: calcd for C_19_H_25_ON_4_ [M + H]^+^, 325.20229; found, 325.20184. HPLC analysis of compound **25d** (purity: 95.11%).

3-(4-(((2,3-Dihydro-1H-inden-2-yl)amino)methyl)phenyl)-N′-propylacrylohydrazide (**25e**). Using the synthetic method of **9a**, compound **24e** gave **25e** as a white solid (80% yield). ^1^H NMR (400 MHz, DMSO-*d*_6_) *δ* 10.82 (s, 1H), 9.46–9.26 (m, 2H), 7.70 (d, *J* = 8.2 Hz, 2H), 7.64–7.55 (m, 3H), 7.23–7.19 (m, 2H), 7.24–7.18 (m, 2H), 6.70 (d, *J* = 15.9 Hz, 1H), 4.11–4.02 (m, 1H), 3.37–3.31 (m, 2H), 3.18–2.92 (m, 2H), 2.94 (t, *J* = 7.5 Hz, 2H), 1.60–1.51 (m, 2H), 0.91 (t, *J* = 7.4 Hz, 3H). ^13^C NMR (126 MHz, DMSO-*d*_6_) *δ* 139.83, 135.57, 134.06, 131.02, 128.44, 127.50, 124.99, 120.28, 57.65, 52.72, 49.11, 36.17, 19.75, 11.65. HRMS (AP-ESI) *m*/*z*: calcd for C_23_H_26_ON_3_ [M + H]^+^, 350.22269; found, 350.22260. HPLC analysis of compound **25e** (purity: 97.02%).

3-(4-((Naphthalen-1-ylamino)methyl)phenyl)-N′-propylacrylohydrazide (**25f**). Using the synthetic method of **9a**, compound **24f** gave **25f** as a white solid (80% yield). ^1^H NMR (400 MHz, DMSO-*d*_6_) *δ* 9.58 (d, *J* = 6.1 Hz, 1H), 8.29–8.20 (m, 1H), 7.78–7.71 (m, 1H), 7.50 (d, *J* = 8.0 Hz, 2H), 7.47–7.38 (m, 5H), 7.16 (t, *J* = 7.8 Hz, 1H), 7.07 (d, *J* = 8.1 Hz, 1H), 7.01 (t, *J* = 6.0 Hz, 1H), 6.49 (d, *J* = 15.8 Hz, 1H), 6.33 (d, *J* = 7.6 Hz, 1H), 5.04 (s, 1H), 4.52 (d, *J* = 5.8 Hz, 2H), 2.70–2.65 (m, 2H), 1.46–1.37 (m, 2H), 0.88 (t, *J* = 7.4 Hz, 3H). ^13^C NMR (126 MHz, DMSO-*d*_6_) *δ* 164.33, 144.00, 142.34, 138.80, 134.50, 133.77, 128.46, 127.99, 127.85, 127.11, 126.09, 124.54, 123.49, 122.04, 115.96, 104.08, 53.53, 46.65, 21.23, 12.02. HRMS (AP-ESI) *m*/*z*: calcd for C_23_H_26_ON_3_ [M + H]^+^, 360.20704; found, 360.20673. HPLC analysis of compound **25f** (purity: 100.0%).

3-(4-(((Naphthalen-1-ylmethyl)amino)methyl)phenyl)-N′-propylacrylohydrazide (**25g**). Using the synthetic method of **9a**, compound **24g** gave **25g** as a white solid (85% yield). ^1^H NMR (400 MHz, DMSO-*d*_6_) *δ* 9.65 (s, 1H), 8.16–8.11 (m, 1H), 7.93–7.89 (m, 1H), 7.81 (d, *J* = 8.1 Hz, 1H), 7.53–7.48 (m, 5H), 7.47–7.41 (m, 4H), 6.55 (d, *J* = 15.8 Hz, 1H), 5.04 (s, 1H), 4.12 (s, 2H), 3.81 (s, 2H), 2.69 (t, *J* = 7.1 Hz, 2H), 1.48–1.38 (m, 2H), 0.88 (t, *J* = 7.4 Hz, 3H). ^13^C NMR (126 MHz, DMSO-*d*_6_) *δ* 164.35, 138.80, 133.98, 133.78, 131.90, 129.23, 128.84, 127.92, 127.85, 126.57, 126.37, 126.10, 125.83, 124.44, 120.22, 53.55, 52.65, 50.04, 21.25, 12.03. HRMS (AP-ESI) *m*/*z*: calcd for C_24_H_28_ON_3_ [M + H]^+^, 374.22269; found, 374.22229. HPLC analysis of compound **25g** (purity: 100.0%).

Methyl-3-(4-((1,3-dioxoisoindolin-2-yl)methyl)phenyl)acrylate (**27**). Using the synthetic method of **21a**, isoindoline-1,3-dione (**26**) and methyl-3-(4-(bromomethyl)phenyl)acrylate gave **27** as a white solid (77% yield). ^1^H NMR (400 MHz, DMSO-*d*_6_) *δ* 7.89–7.79 (m, 4H), 7.59 (d, *J* = 16.1 Hz, 1H), 7.55–7.48 (m, 2H), 7.31 (d, *J* = 8.0 Hz, 2H), 6.57 (d, *J* = 16.0 Hz, 1H), 4.76 (s, 2H), 3.67 (s, 3H). ESI-MS *m*/*z*: 321.88 [M + H]^+^.

3-(4-((1,3-Dioxoisoindolin-2-yl)methyl)phenyl)acrylic acid (**28**). Using the synthetic method of compound **7a**, compound **27** gave **28** as a white solid (80% yield). ^1^H NMR (400 MHz, DMSO-*d*_6_) *δ* 8.85 (t, *J* = 6.0 Hz, 1H), 7.75–7.73 (m, 1H), 7.60 (d, *J* = 8.2 Hz, 2H), 7.58–7.52 (m, 2H), 7.50–7.46 (m, 1H), 7.44–7.42 (m, 1H), 7.39 (d, *J* = 8.1 Hz, 2H), 6.47 (d, *J* = 16.0 Hz, 1H), 4.42 (d, *J* = 6.0 Hz, 2H).

Tert-butyl-2-(3-(4-((1,3-dioxoisoindolin-2-yl)methyl)phenyl)acryloyl)-1-propylhydrazine-1-carboxylate (**29**). Using the synthetic method of **8a**, compound **28** and compound **4** gave **29** as a pale yellow solid (58% yield). The resulting crude product was directly used for the next reaction.

3-(4-((1,3-Dioxoisoindolin-2-yl)methyl)phenyl)-N′-propylacrylohydrazide (**30**). Using the synthetic method of **9a**, compound **29** gave **30** as a white solid (85% yield). ^1^H NMR (400 MHz, DMSO-*d*_6_) *δ* 11.38 (s, 1H), 7.96–7.84 (m, 4H), 7.63 (t, *J* = 8.1 Hz, 3H), 7.38 (d, *J* = 7.9 Hz, 2H), 6.63 (d, *J* = 15.8 Hz, 1H), 4.80 (s, 2H), 3.07 (t, *J* = 7.8 Hz, 2H), 1.66–1.58 (m, 2H), 0.91 (t, *J* = 7.4 Hz, 3H). ^13^C NMR (126 MHz, DMSO-*d*_6_) *δ* 164.49, 142.06, 139.42, 135.09, 133.68, 132.02, 128.77, 128.40, 123.74, 117.89, 52.04, 41.07, 38.69, 18.38, 11.28. HRMS (AP-ESI) *m*/*z*: calcd for C_21_H_22_O_3_N_3_ [M + H]^+^, 364.16557; found, 364.16516. HPLC analysis of compound **30** (purity: 99.08%).

### 4.2. Cell Culture

The AML cell lines MV4-11, THP-1, KASUMI-1 and HL-60 were purchased from Procell Life Science & Technology Co., Ltd. (Wuhan, China). THP-1 and KASUMI-1 were cultured in RPMI-1640 complete medium containing 10% fetal bovine serum and 1% penicillin and streptomycin. MV4-11 was cultured in IMDM complete medium containing 10% fetal bovine serum and 1% penicillin and streptomycin. HL-60 was cultured in IMDM complete medium containing 20% fetal bovine serum and 1% penicillin and streptomycin. All cell lines were cultured in an incubator with humidity set at 95%, temperature at 37 °C and 5% CO_2_.

#### 4.2.1. In Vitro HDAC Inhibition Fluorescence Assay

All of the HDAC enzymes were purchased from BPS Bioscience. In vitro HDAC inhibition assays were conducted as previously described. In brief, 20 μL of recombinant HDAC enzyme solution (HDAC1-9, 11) was mixed with various concentrations of the tested compound (20 μL). The mixture was incubated at 37 °C for 1 h, and then, 10 μL of fluorogenic substrate (Boc-Lys (acetyl)-AMC for HDAC1, 2, 3 and 6; Boc-Lys (trifluoroacetyl)AMC for HDAC4, 5, 7, 8, 9 and 11) was added to achieve a final concentration of 50 μM. After incubation at 37 °C for 2 h, the catalysis was stopped by the addition of 10 μL of developer containing 30 mg/mL trypsin and 6 μM trichostatin A (TSA). Then, 30 min later, the fluorescence intensity was measured using a microplate reader at excitation and emission wavelengths of 360 and 460 nm, respectively. The inhibition ratios were calculated from the fluorescence intensity readings of tested wells relative to those of control wells, and the IC_50_ curves and values were determined by GraphPad Prism 6.0, using the “log(inhibitor) vs. normalized response-variable slope” function.

#### 4.2.2. Cell Viability Assay

MV4-11, THP-1, KASUMI-1 and HL-60 cells were plated in 96-well transparent plates (10,000 to 15,000 cells/100 μL) and after incubation overnight, cells were treated with gradient-diluted inhibitors with several concentrations. After the cells were incubated with inhibitors for 72 h, 1 mg/mL of Resazurin (Sigma-Aldrich, Saint Louis, MO, USA) was added (working concentration was 0.1 mg/mL). After incubation at 37 °C for about 3 h, the fluorescence intensity of resorcinol was measured at 560 nm/590 nm (excitation/emission) with a Spectra Max i3 Microplate Reader, and the cell viability was determined. The IC_50_ curve was drawn using GraphPad Prism 9.5 software.

#### 4.2.3. Microdilution Checkerboard Assay

The inhibition effect of **25c** combined with Venetoclax on the proliferation of MV4-11 cells was determined by microdilution checkerboard assay. CompuSyn software was used to calculate the combined action index of the two compounds, and the cell inhibition rates of the two compounds acting alone and the combined action were input to calculate the combination index (CI) to evaluate the synergistic effect of the drugs. A CI value less than 1 indicates a synergistic effect, a CI value equal to 1 indicates an additive effect and a CI value greater than 1 indicates an antagonistic effect.

#### 4.2.4. Cell Cycle and Apoptosis Study

MV4-11 cells were plated into six-well plates at a density of 2.5 × 10^6^ per well. After incubation overnight, cells were treated with different concentrations of **25c** and Venetoclax alone or in combination for 24 h. Then, cells were harvested, washed once with 1 mL PBS and fixed with 75% pre-cooled ethanol at 4 °C overnight. The next day, fixed cells were washed twice with PBS, and incubated with DNase-free Rnase A and propidium iodide using a Cell Cycle Detection Kit (Absin, Shanghai, China). The cell cycle distribution was analyzed by flow cytometry (BD Accuri C6, Becton Dickinson, NJ, USA). Data were analyzed by FlowJo for Windows V10.8.1 software.

MV4-11 cells were inoculated in a 12-well plate at a density of 1 × 10^6^ cells per well. After incubation overnight, cells were treated with different concentrations of **25c** and Venetoclax alone or in combination. After 24 h, cells were collected, washed once with 1 mL PBS, re-suspended with 300 μL 1× binding buffer, and filtered through a cell filter to prepare a single-cell suspension. The cells were stained with AnnexinV-FITC 5 μL and propidium iodide (Absin, China) 5 μL and incubated at room temperature for 15 min, and apoptosis was analyzed by BD Accuri C6 Plus flow cytometry (BD Accuri C6, Becton Dickinson, USA). Data were analyzed by FlowJo for Windows V10.8.1 software.

#### 4.2.5. Western Blot Analysis

MV4-11 cells were inoculated into 12-well plates at a density of 800,000 cells/mL. After incubation overnight, each well was treated with different concentrations of **25c** and Venetoclax alone or in combination. After treatment for 24 h, the cells were collected and washed with pre-cooled PBS. A protease inhibitor (cocktail) and RIPA buffer were added for cell lysis. The decomposed solution was centrifuged at 12,000 rpm for 15 min at 4 °C, and the extracted protein was located in the supernatant. The proteins in each sample were denatured by heating at 100 °C in loading buffer (5×) for 10 min. The same amount of proteins in each sample was separated by SDS-PAGE and then electrotransferred to a PVDF membrane (Merck Millipore, Billerica, MA, USA) and enclosed in 5% skim milk powder for 1 h. The primary antibody diluted with an immune response booster solution was incubated in a shaking bed at 4 °C overnight, and the horseradish (HRP)-conjugated second antibody diluted with 5% skim milk powder was incubated at room temperature for 1 h on the second day. After the PVDF membrane was washed with TBST, the target protein bands could be detected with chemiluminescent reagents (Millipore, Billerica, MA, USA) and visualized in the Tanon 5200 + fluorescence imager.

The primary antibodies Caspase 3 (including Cleaved Caspase-3) (sc-56053), Mcl-1 (94296S), Bcl-xL (ab98143), γ-H2AX (9718S), AcHH3 (Lys27) (8173S), AcHH4 (sc-377520), HH3 (ab1791), Ac-tubulin (sc-23950), HDAC6 (sc-28386), α-tubulin (sc-23948), GAPDH (sc-59540) and β-actin (8H10D10) were purchased from Abcam, Cell Signaling Technology and Santa Cruz Biotechnology.

### 4.3. Molecular Docking against HDAC3

Docking sites were defined using Schrodinger, and the simulations used a rigid receptor and triangle matcher methodology for ligand site placement with Schrodinger scoring. One hundred fifteen poses were calculated per binding site. Structural files included PDB 4A69 for HDAC3.

### 4.4. In Vitro Stability of ***25c***

To evaluate the stability of compound **25c** in vitro, 15 μL 6 μg/mL reserve solution of compound **25c** was incubated with 135 μL rat plasma, rat liver homogenate, artificial intestinal oil and artificial gastric fluid at 37 °C for different time points: 0 h, 0.5 h, 1 h, 2 h, 4 h, 8 h, 12 h and 24 h. Equal samples were collected, and 600 μL acetonitrile was added to quench enzyme reactions. The reaction mixture was swirled for 30 s and then centrifuged at 15,000 rpm at 4 °C for 15 min. The supernatant of each sample was analyzed directly by HPLC (1260 Infinity II, Agilent Technologies, Waldbronn, Germany).

## Figures and Tables

**Table 1:** Structure of compounds (**9a**–**9f**, **14a**–**14f**) and their inhibition rate against HDAC1/3.

Compound No.	Structure	Inhibition Rate of HDAC1 (%) ^α^		Inhibition Rate of HDAC3 (%) ^α^	
		100 nM	10 nM	100 nM	10 nM
**9a**	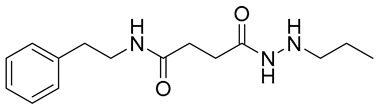	0.3 ± 1.7	2.2 ± 2.3	11.1 ± 0.5	−0.6 ± 1.9
**9b**	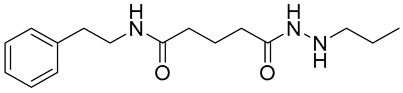	3.1 ± 1.1	−9.2 ± 1.4	53.7 ± 0.0	11.50 ± 0.7
**9c**	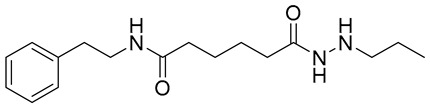	27.5 ± 0.6	−6.00± 1.2	61.9 ± 0.7	18.2 ± 2.9
**9d**	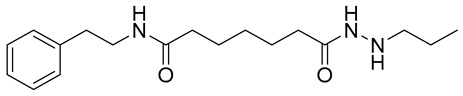	51.96 ± 2.86	5.58 ± 2.39	76.82 ± 0.47	21.2 ± 1.5
**9e**	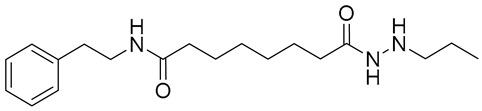	65.6 ± 0.1	12.04 ± 0.9	85.5 ± 0.6	38.8 ± 3.0
**9f**	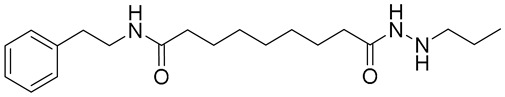	57.1 ± 0.7	15.9 ± 1.6	79.9 ± 0.8	19.6 ± 8.0
**14a**	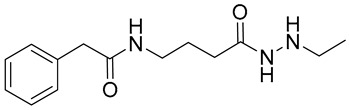	2.3 ± 2.4	−7.0 ± 2.6	18.2 ± 3.3	2.4 ± 2.9
**14b**	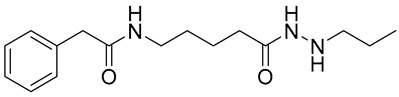	7.7 ± 2.1	−9.04 ± 0.1	45.2 ± 1.1	16.6 ± 3.2
**14c**	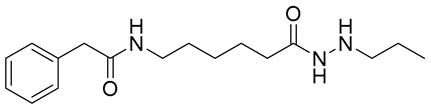	14.4 ± 2.4	−6.8 ± 2.1	54.4 ± 0.7	16.1 ± 3.3
**14d**	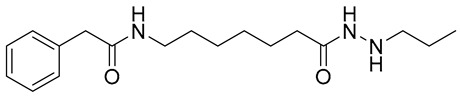	43.2 ± 0.1	1.9 ± 3.7	76.8 ± 0.4	21.2 ± 1.5
**14e**	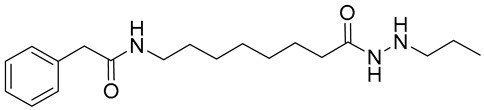	51.6 ± 0.9	8.8 ± 3.7	71.5 ± 0.7	23.1 ± 0.1
**14f**	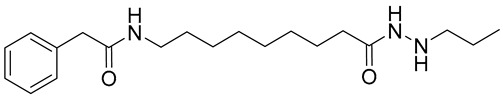	32.7 ± 1.2	9.8 ± 0.4	48.8 ± 1.1	3.2 ± 10.6
**SCA**	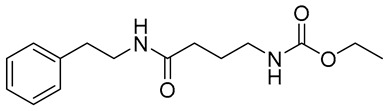	/	/	/	/
**Entinostat**	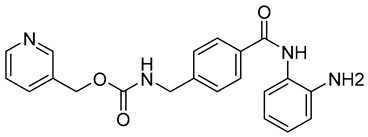	37.6 ± 2.6	3.8 ± 1.1	77.9 ± 1.6	12.5 ± 1.0

^α^ Inhibition rate values are the mean of three independent experiments ± the standard error of the mean (SEM).

**Table 2:** Structure of compounds (**9g**–**9m**, **25c**) and their inhibition rate against HDAC1/3.

Compound No.	Structure	Inhibition Rate of HDAC1 (%) ^α^		Inhibition Rate of HDAC3 (%) ^α^	
		100 nM	10 nM	100 nM	10 nM
**9g**	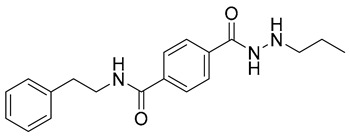	30.1 ± 2.1	23.5 ± 2.0	50.6 ± 5.4	11.2 ± 8.0
**9h**	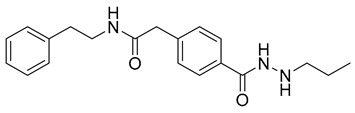	10.1 ± 0.7	5.61 ± 1.6	−5.9 ± 0.6	−4.61 ± 13.6
**9i**	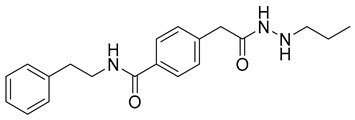	16.4 ± 1.2	12.5 ± 0.2	−3.1 ± 3.4	−0.8 ± 4.2
**9j**	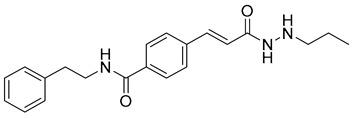	76.7 ± 0.3	32.8 ± 0.1	89.4 ± 0.3	64.5 ± 0.1
**9k**	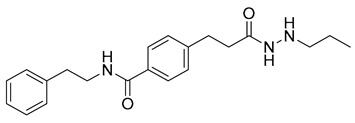	67.9 ± 1.4	23.1 ± 2.1	85.8 ± 0.5	58.6 ± 4.6
**9l**	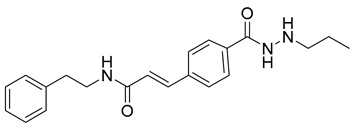	74.5 ± 0.3	36.5 ± 2.1	87.2 ± 2.6	78.1 ± 0.1
**9m**	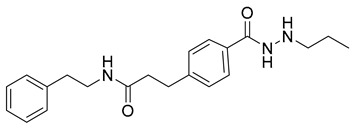	67.3 ± 2.8	21.2 ± 0.9	80.7 ± 0.7	27.2 ± 2.7
**25c**	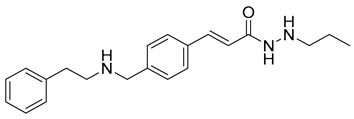	83.6 ± 0.4	31.13 ± 2.09	95.1 ± 0.1	96.1 ± 0.3
**SCA**	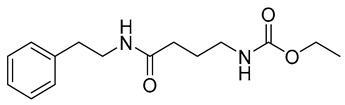	/	/	/	/
**Entinostat**	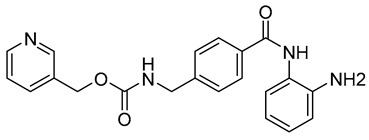	37.6 ± 2.6	3.8 ± 1.1	77.9 ± 1.6	12.5 ± 1.0

^α^ Inhibition rate values are the mean of three independent experiments ± SEM.

**Table 3:** Structure of compounds (**25a**–**25g**) and their inhibition rate against HDAC1/3.

Compound No.	Structure	Inhibition Rate of HDAC1 (%) ^α^		Inhibition Rate of HDAC3 (%) ^α^	
		100 nM	10 nM	100 nM	10 nM
**25a**	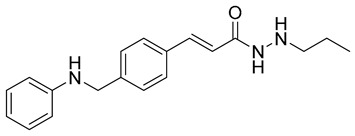	60.7 ± 0.7	14.2 ± 1.5	80.4 ± 0.7	49.2 ± 0.9
**25b**	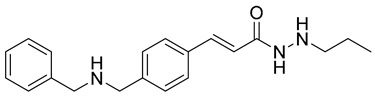	77.0 ± 0.3	21.8 ± 1.6	97.9 ± 0.2	94.8 ± 0.1
**25c**	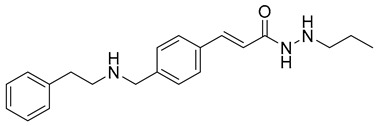	83.6 ± 0.4	31.1 ± 2.1	95.1 ± 0.1	96.1 ± 0.3
**25d**	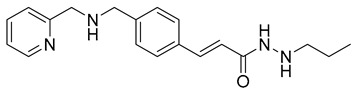	61.7 ± 1.1	9.3 ± 2.3	86.9 ± 0.1	62.5 ± 0.4
**25f**	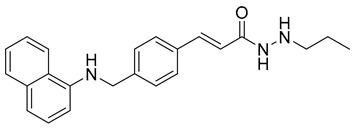	71.2 ± 0.9	31.3 ± 0.0	60.7 ± 0.5	25.7 ± 1.3
**25g**	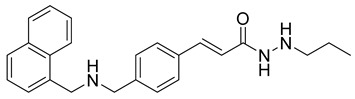	76.5 ± 5.0	33.9 ± 0.0	71.1 ± 1.6	45.9 ± 0.0
**25e**	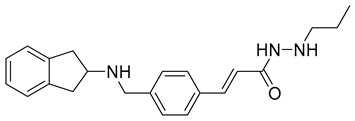	78.7 ± 0.7	10.9 ± 1.2	95.6 ± 0.2	94.8 ± 0.5
**30**	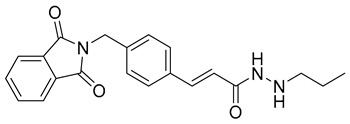	28.4 ± 1.1	5.1 ± 1.3	85.1 ± 0.1	53.9 ± 0.4
**19a**	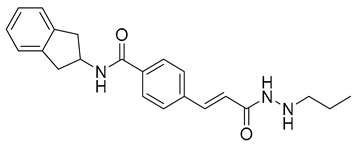	39.9 ± 0.6	8.1 ± 0.3	78.2 ± 0.5	37.4 ± 0.1
**19b**	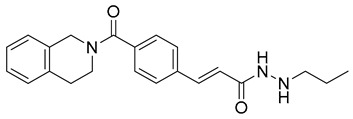	−0.4 ± 1.7	−4.6 ± 2.1	18.3 ± 0.5	−1.1 ± 2.3
**19c**	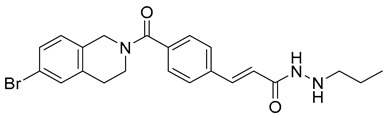	27.2 ± 2.8	1.5 ± 1.5	38.1 ± 0.8	4.1 ± 9.9
**19d**	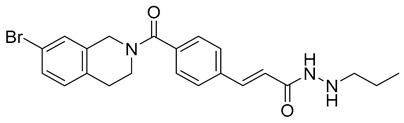	38.2 ± 2.3	−1.4 ± 0.5	34.8 ± 1.2	−0.4 ± 3.4
**SCA**	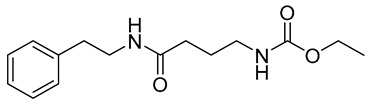	/	/	/	/
**Entinostat**	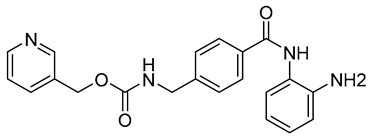	37.6 ± 2.6	3.8 ± 1.1	77.9 ± 1.6	12.5 ± 1.0

^α^ Inhibition rate values are the mean of three independent experiments ± SEM.

**Table 4:** Structure of compounds (**9j**–**9l**, **25a**–**25g** and **30**) and their IC_50_ values against HDAC1/3.

Compound No.	Structure	IC_50_ (nM) ^α^		
		HDAC1	HDAC2	HDAC3
**9j**	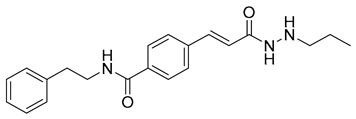	35.8 ± 3.6	69.4 ± 3.4	3.9 ± 0.3
**9k**	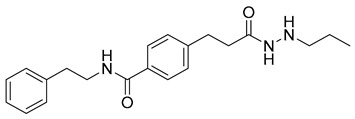	121.6 ± 18.6	373.7 ± 21.7	10.1 ± 1.4
**9l**	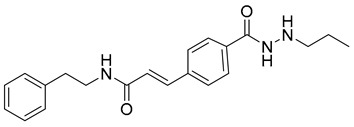	37.4 ± 5.8	116.3 ± 11.4	6.0 ± 1.7
**25a**	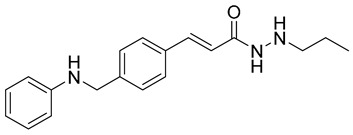	96.5 ± 1.1	224.1 ± 19.4	8.2 ± 0.9
**25b**	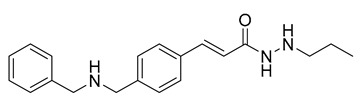	27.0 ± 2.0	111.2 ± 16.9	2.7 ± 0.6
**25c**	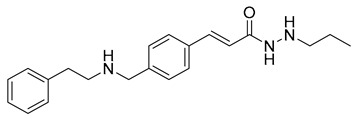	28.1 ± 8.1	134.9 ± 13.3	2.39 ± 0.9
**25d**	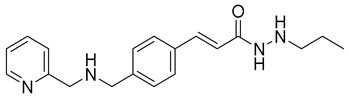	100.6 ± 27.0	314.3 ± 36.7	13.0 ± 6.4
**25e**	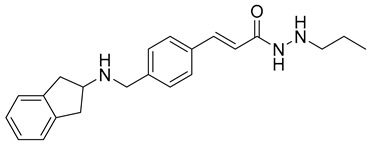	36.5 ± 5.0	160.7 ± 29.0	3.1 ± 1.5
**25g**	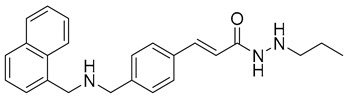	16.0 ± 1.0	46.3 ± 4.1	/
**30**	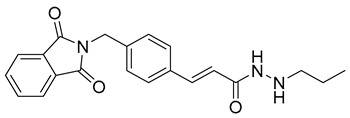	135.5 ± 20.6	651.9 ± 3.4	27.8 ± 1.8
**SCA**	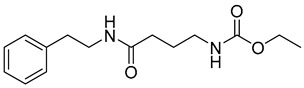	/	/	/
**Entinostat**	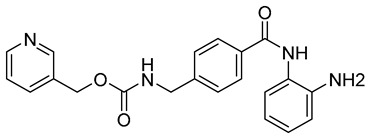	37.6 ± 2.6	3.8 ± 1.1	77.9 ± 1.6

^α^ IC_50_ values are the mean of three independent experiments ± SEM.

**Table 5:** In vitro anti-proliferative activity of representative compounds (**25b**, **25c**, **25e**, **25g**).

Compound No.	IC_50_ (nM) ^α^			
	MV4-11	THP-1	HL-60	Kasumi-1
**25b**	361.9 ± 76.8	516.2 ± 145.1	735.6 ± 24.8	448.4 ± 121.7
**25c**	340.1 ± 116.6	391.8 ± 60.5	480.3 ± 177.3	337.6 ± 125.4
**25e**	353.2 ± 118.9	450.6 ± 73.8	502.0 ± 189.4	380.5 ± 147.0
**25g**	581.5 ± 240.2	1017.0 ± 498.1	649.8 ± 246.9	1010.4 ± 642.7
**SCA**	>10,000	>10,000	>10,000	>10,000
**Entinostat**	651.2 ± 37.4	1516.3 ± 218.0	1102.3 ± 51.5	480.3 ± 160.9

^α^ IC_50_ values are the mean of three independent experiments ± SEM.

**Table 6:** The HDAC inhibitory activity of **25c** in vitro.

HDAC Classes	Subtype	IC_50_ (nM) ^α^
Class I	HDAC1	28.0 ± 11.5
	HDAC2	134.9 ± 18.5
	HDAC3	2.3 ± 1.3
	HDCA8	4109 ± 630
Class II a	HDAC4	>10,000
	HDAC5	>10,000
	HDAC7	>10,000
	HDAC9	>10,000
Class II b	HDAC6	>10,000
Class IV	HDAC11	>10,000

^α^ IC_50_ values are the mean of three independent experiments ± SEM.

## Data Availability

Data are contained within the article or Supplementary Materials.

## Supplementary Materials

The following supporting information can be downloaded at https://www.mdpi.com/article/10.3390/md22060250/s1: Figures S1–S3: IC_50_ curves representing compounds’ in vitro inhibitory activity or anti-proliferative activity. Figures S4–S7: Results of repeated tests on the biological aspects of **25c**, including the Western blot of **25c** single-drug selectivity, combination index (CI) for **25c** and Venetoclax after treatment 72h, and induction of apoptosis and cell cycle by **25c** and Venetoclax in MV4-11 cells. ^1^H NMR and ^13^C NMR spectrum and HRMS (AP-ESI) of target compounds. HPLC traces and purity of the target compounds.
