# Supplementary material for: Marine Cytotoxin Santacruzamate A Derivatives as Potent HDAC1-3 Inhibitors and Their Synergistic Anti-Leukemia Effects with Venetoclax
Source: Mar Drugs. 2024 May 28;22(6):250. doi: 10.3390/md22060250 (PMC11204923; doi:10.3390/md22060250)
Supplement: Supplementary file 1 [file marinedrugs-22-00250-s001.zip › marinedrugs-3011920-supplementary.pdf]

Marine Cytotoxin Santacruzamate A Derivatives as potent HDAC1-3 inhibitors and their synergistic anti-leukemia effects with Venetoclax

Wanting Hao[1,#], Leyan Wang[1,#], Tongqiang Xu[1], Yuqi Jiang[1], Chong Qin[1], and Xiaoyang Li[1,*]
[1]*Key Laboratory of Marine Drugs, Chinese Ministry of Education, School of Medicine and Pharmacy, Ocean University of China, Qingdao 266003, China;*
[#]*Wanting Hao and Leyan Wang contributed equally to this work.*
Corresponding author
lixiaoyang@ouc.edu.cn (X. L.)

Contents

Figure S1. IC_{50} curves of target compounds toward HDAC1, 2 and 3....................................2
Figure S2. IC_{50} curves of compound **25c** toward HDAC4, 5, 6, 7, 8, 9, and 11. 2
Figure S3. IC_{50} curves of target compounds against MV4-11 cell line....................................3
Figure S4. Western blot analysis of **25c**-inhibited enzyme substrates 3
Figure S5. Induction of apoptosis at 24 h by compound **25c** and Venetoclax single or in combination with different concentrations in MV4-11 cells 4
Figure S6. Induction of cell cycle at 24 h by compound **25c** and Venetoclax single or in combination with different concentrations in MV4-11 cells 5
Figure S7. Changes in Bcl-2 family apoptotic regulatory proteins and apoptotic pathway proteins detected by western blot..5
^{1}H NMR / ^{13}C NMR Spectrums for all the target compounds...6
HPLC traces and purity of the target compounds....53

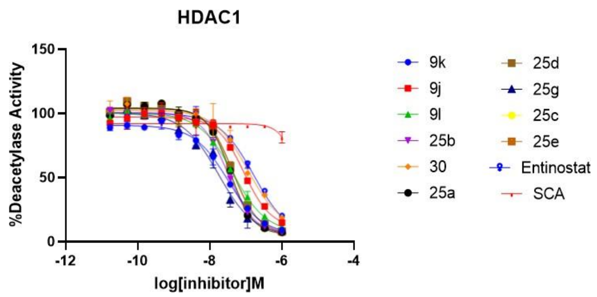

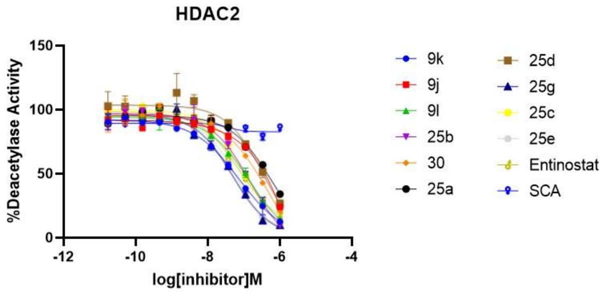

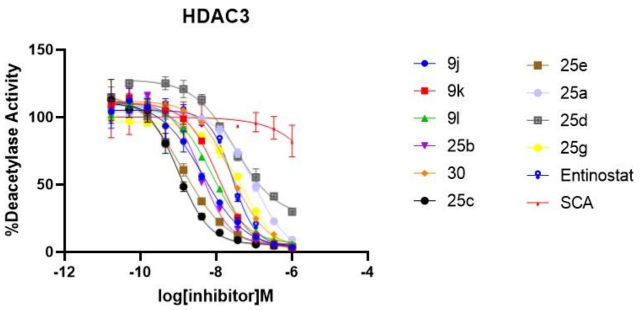

Figure S1. IC_{50} curves of target compounds toward HDAC1, 2 and 3.

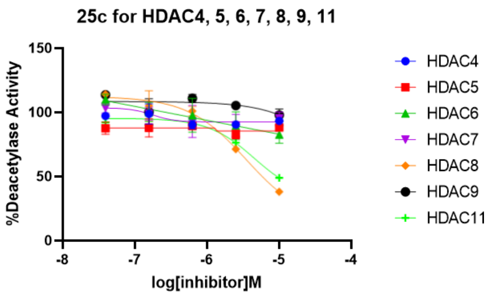

Figure S2. IC_{50} curves of compound **25c** toward HDAC4, 5, 6, 7, 8, 9, and 11.

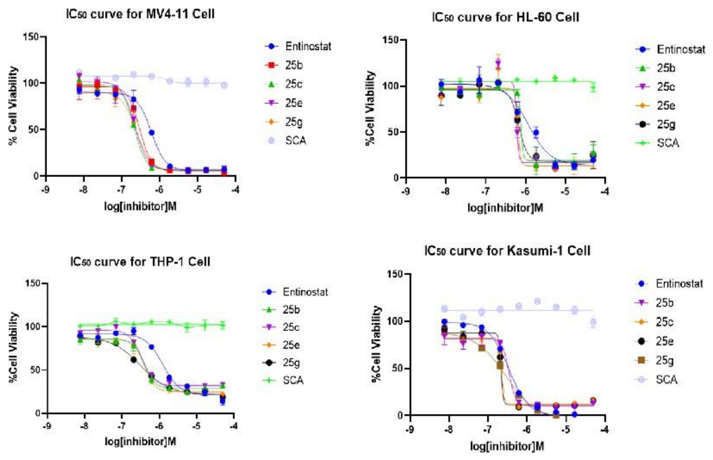

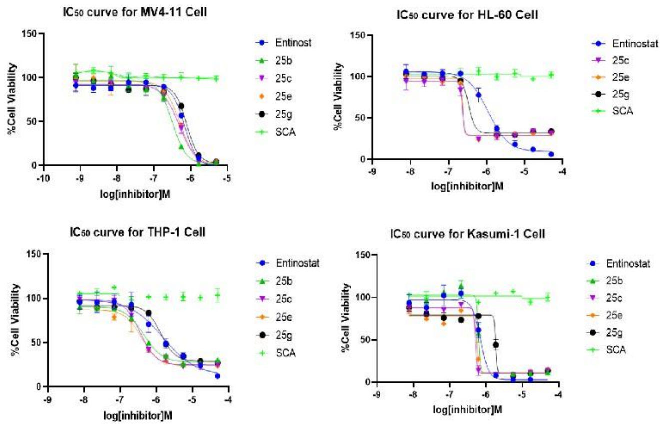

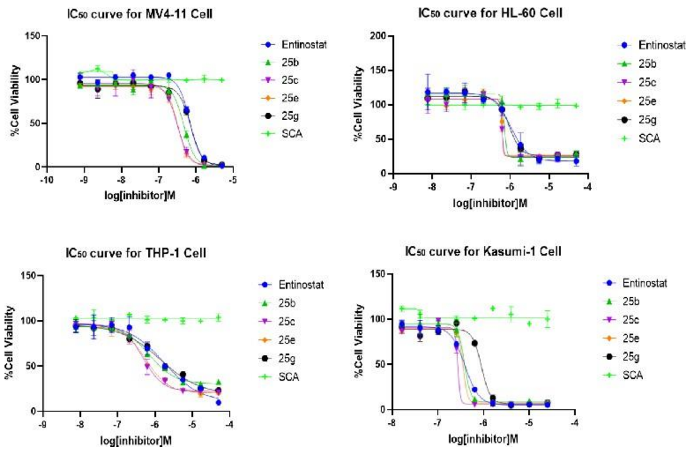

Figure S3. IC_{50} curves of target compounds against MV4-11 cell line.

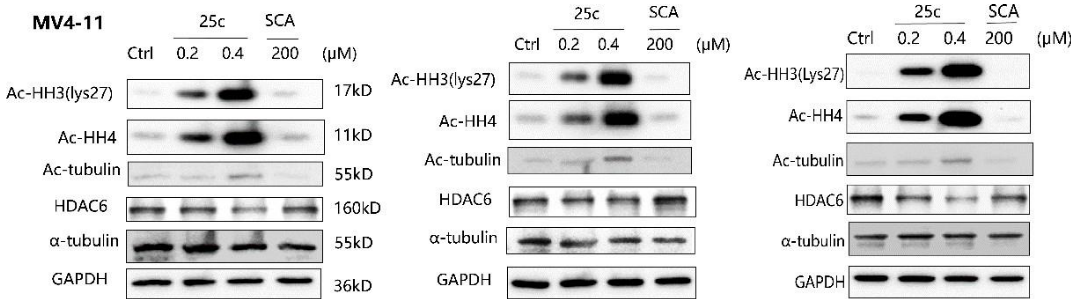

Figure S4. Western blot analysis of **25c**-inhibited enzyme substrates.

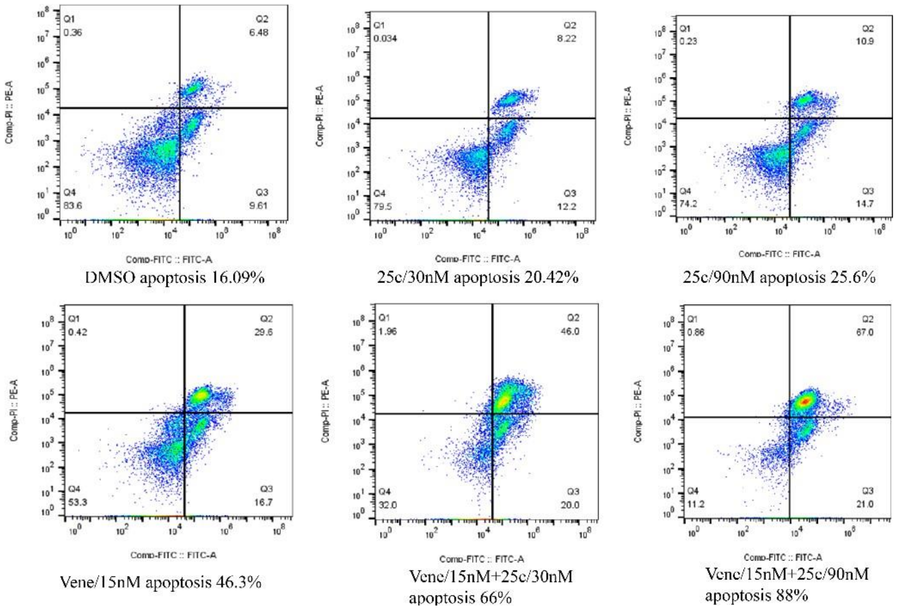

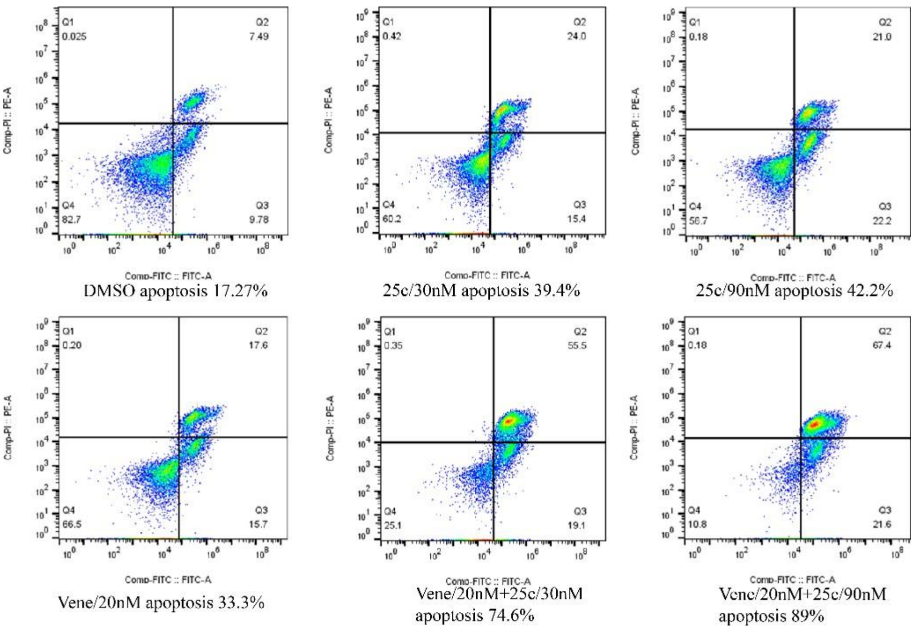

Figure S5. Induction of apoptosis at 24 h by compound **25c** and Venetoclax single or in combination with different concentrations in MV4-11 cells.

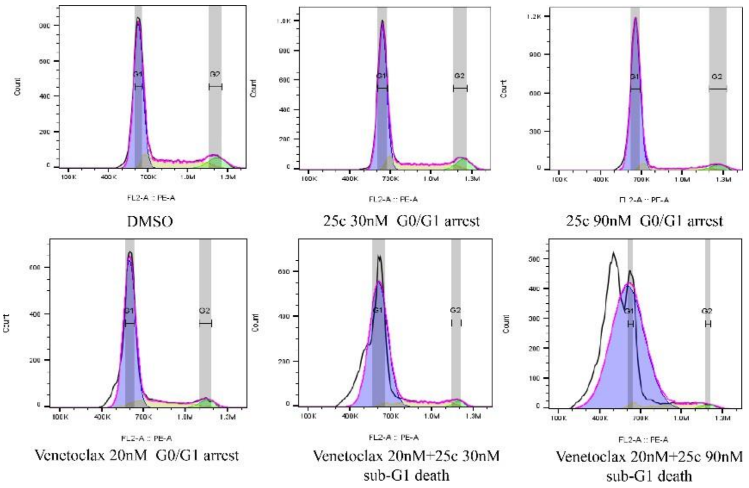

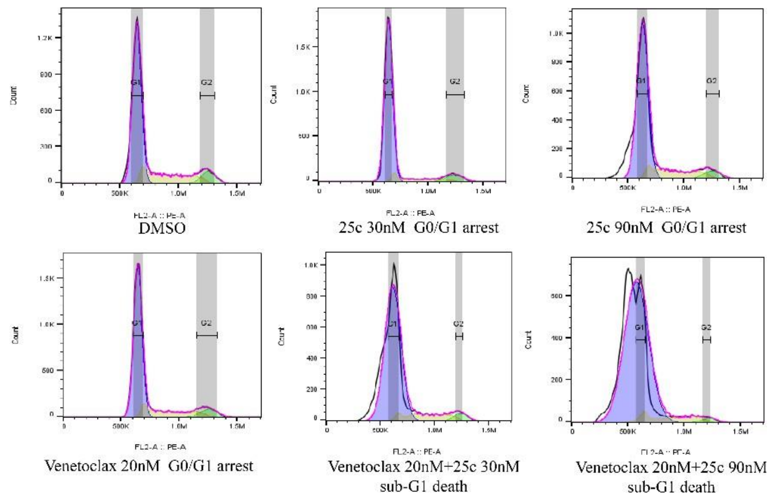

Figure S6. Induction of cell cycle at 24 h by compound **25c** and Venetoclax single or in combination with different concentrations in MV4-11 cells.

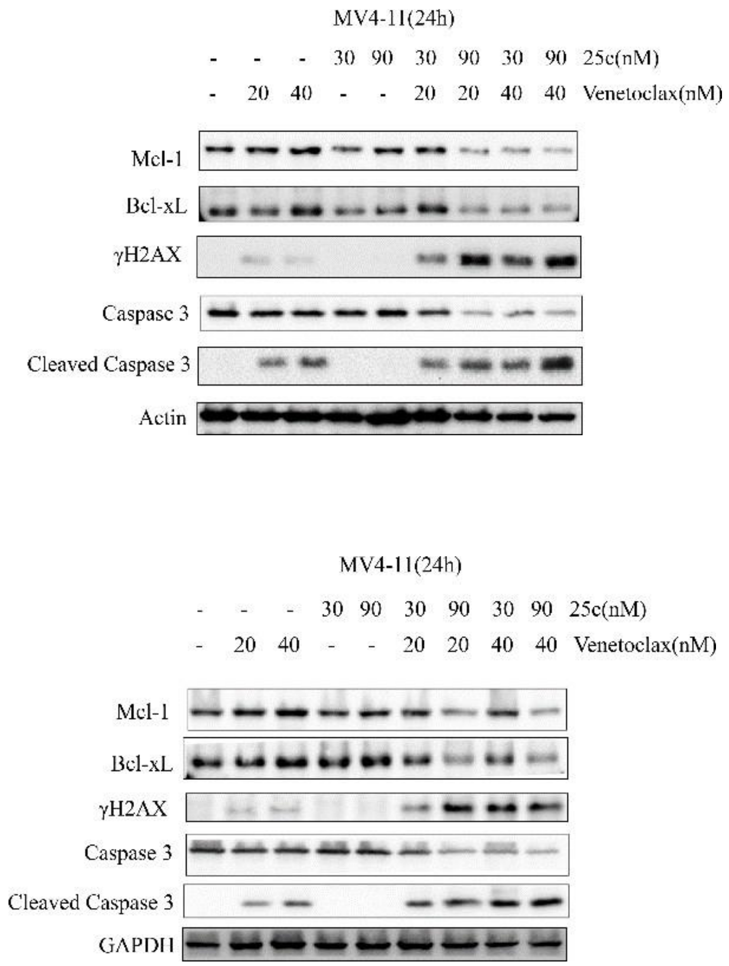

Figure S7. Changes in Bcl-2 family apoptotic regulatory proteins and apoptotic pathway proteins detected by western blot.

^{1}H NMR / ^{13}C NMR Spectrums for all the target compounds.

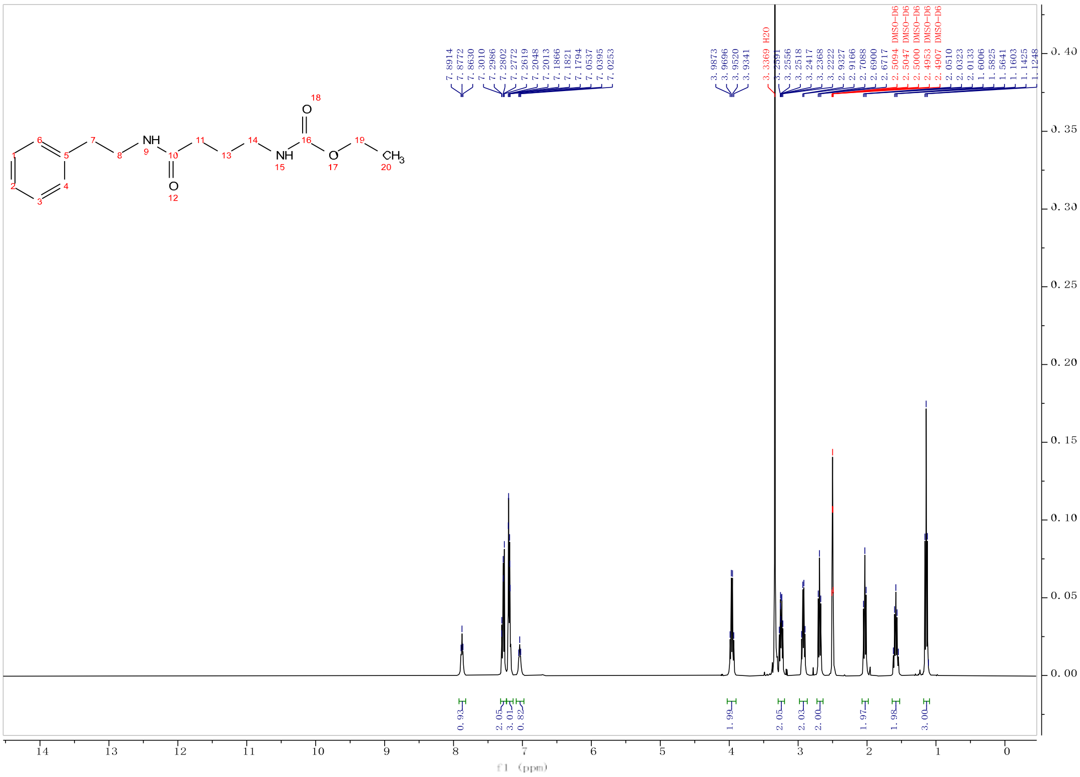

^{1}H NMR spectra for compound **SCA**

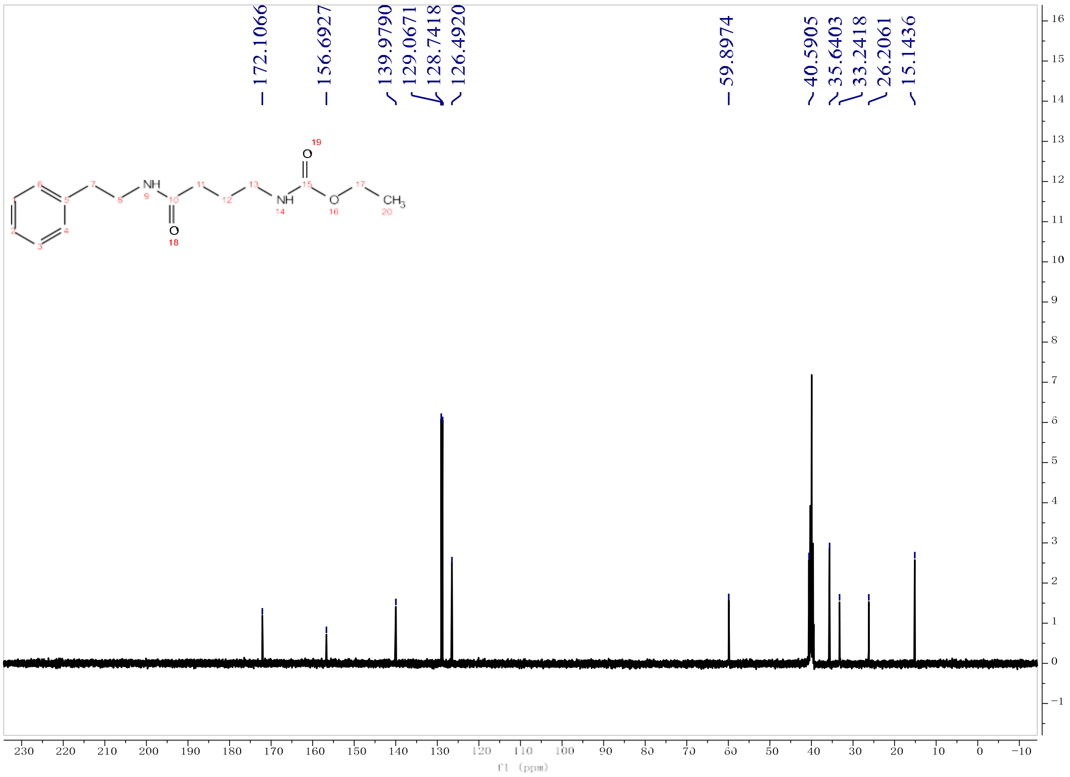

^{13}C NMR spectra for compound **SCA**

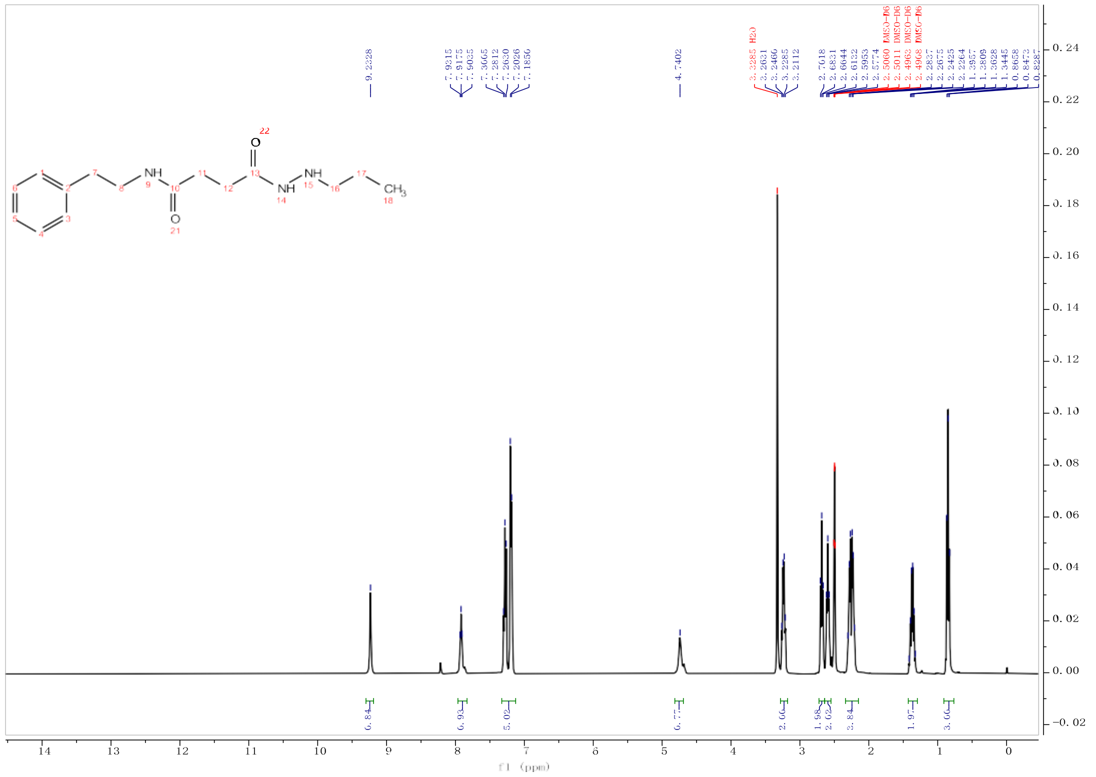

^{1}H NMR spectra for compound **9a**

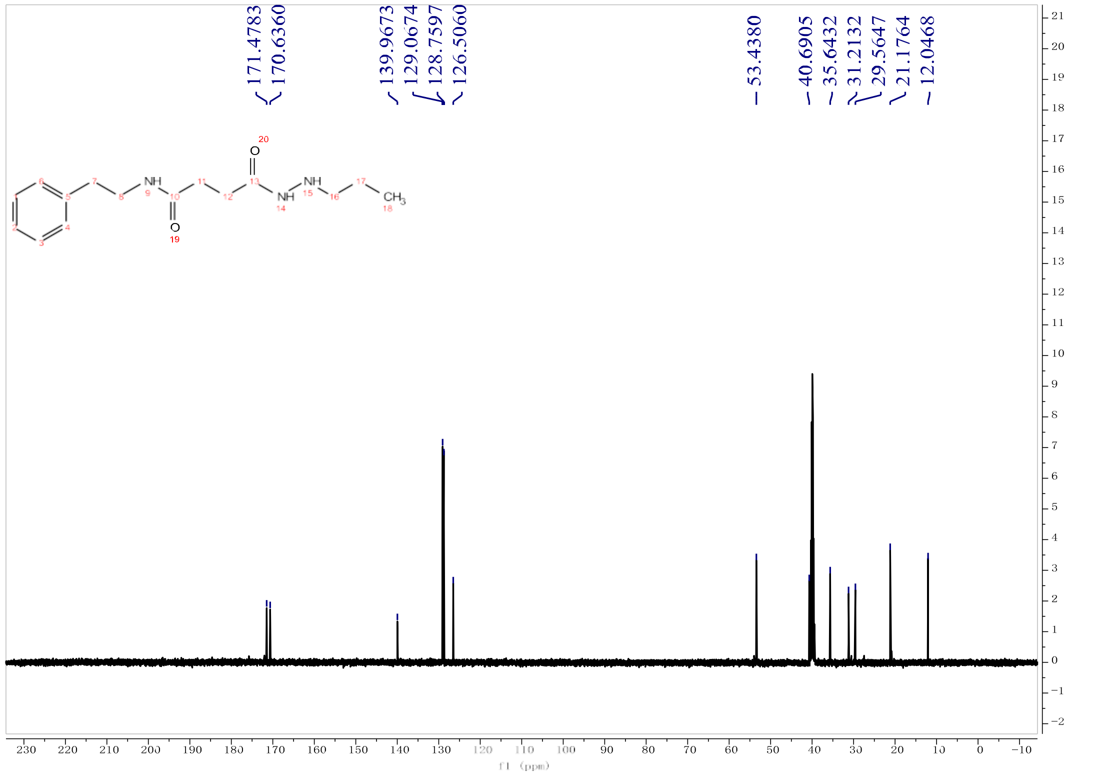

^{13}C NMR spectra for compound **9a**

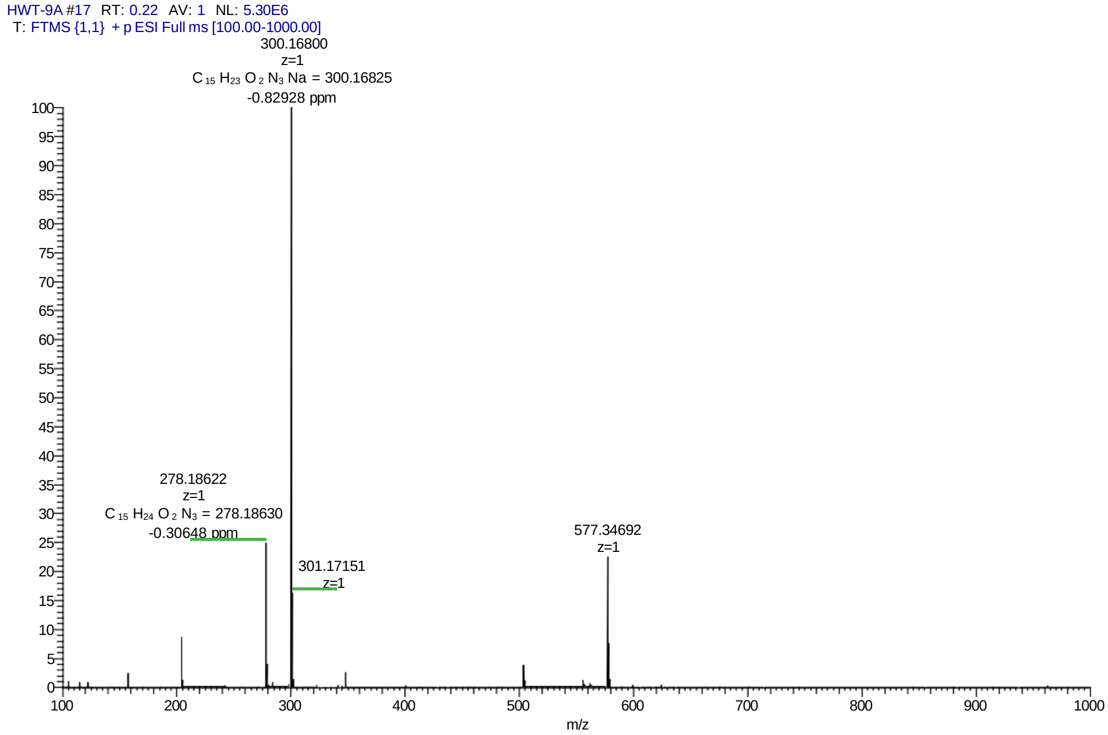

HRMS (AP-ESI) spectrum of compound **9a**

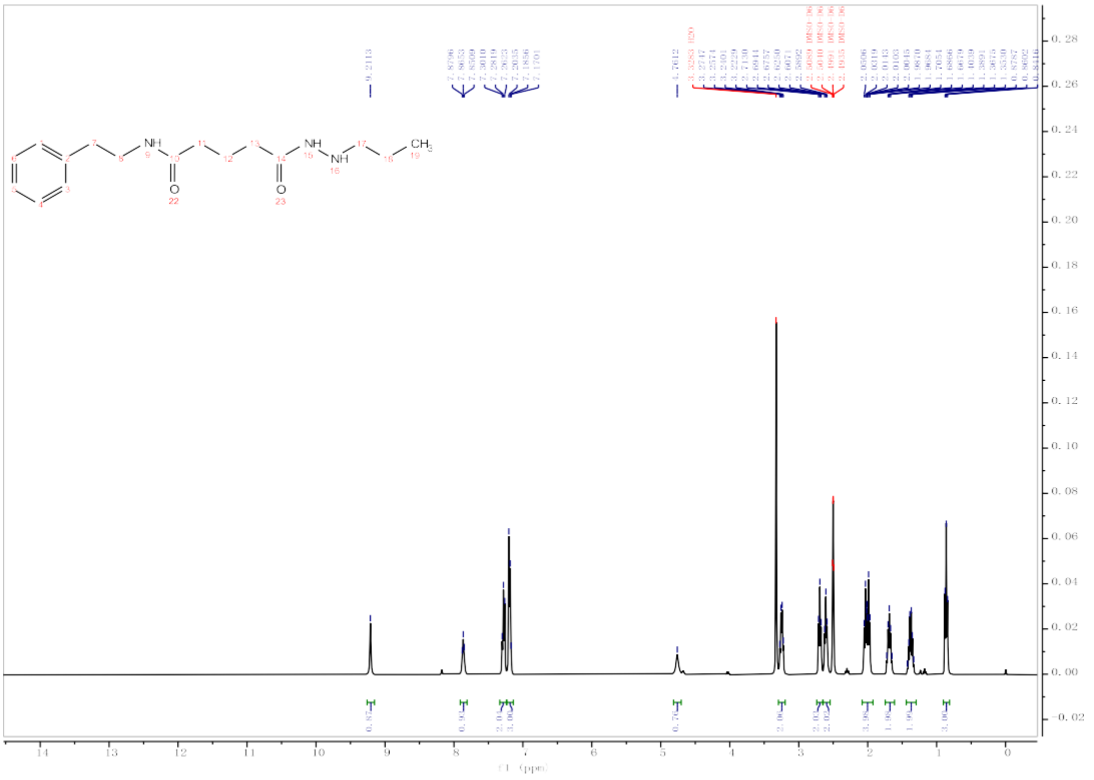

^{1}H NMR spectra for compound **9b**

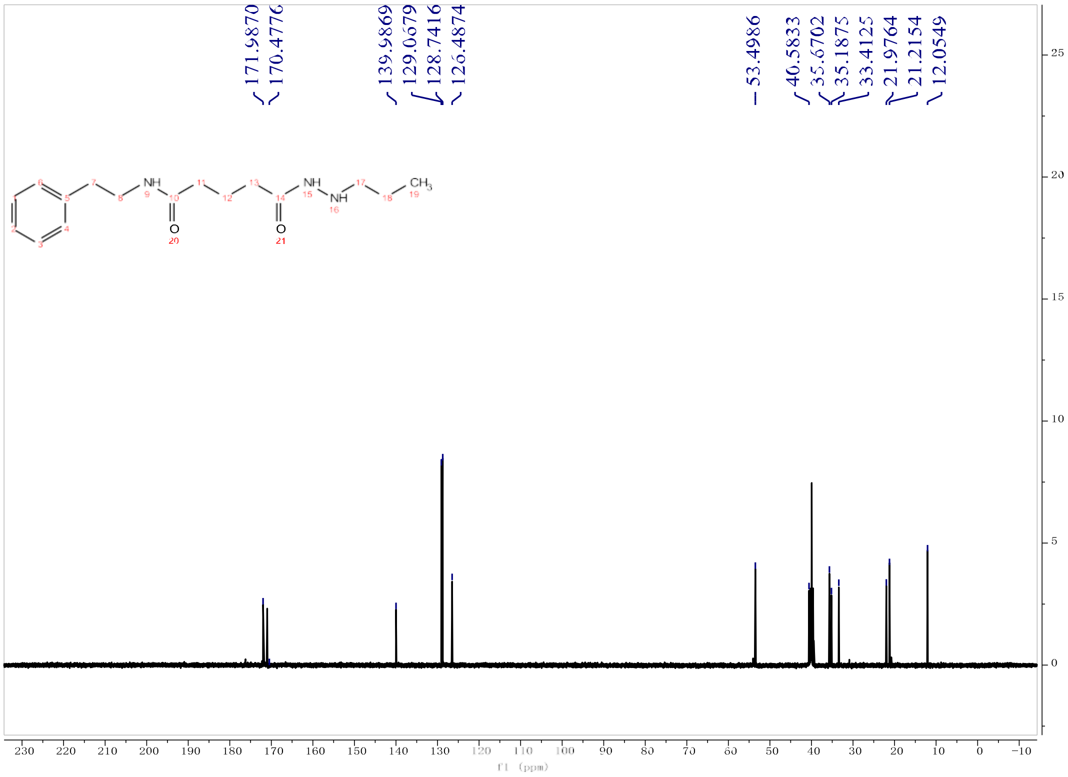

^{13}C NMR spectra for compound **9b**

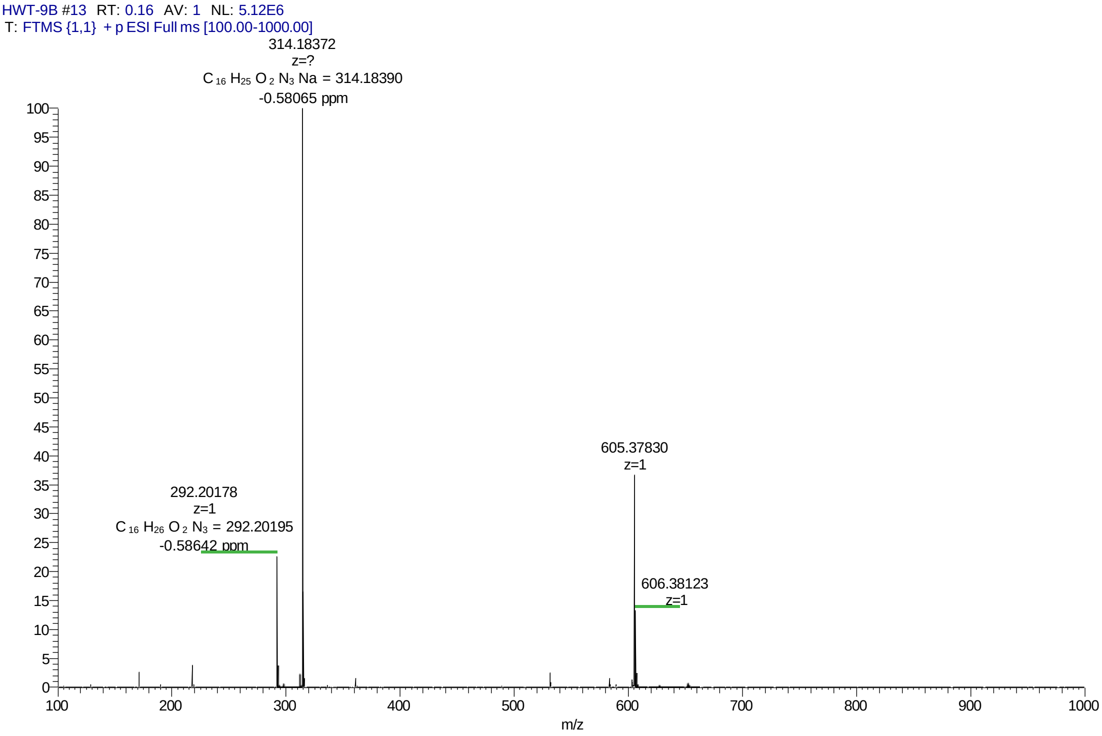

HRMS (AP-ESI) spectrum of compound **9b**

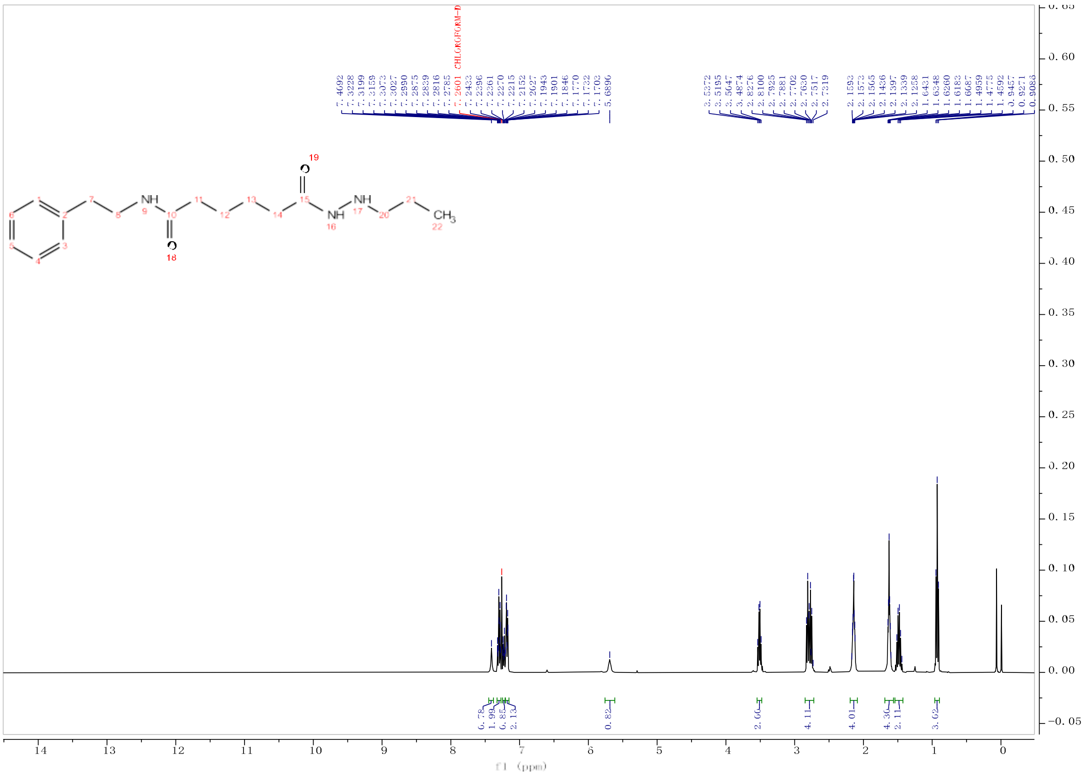

^{1}H NMR spectra for compound **9c**

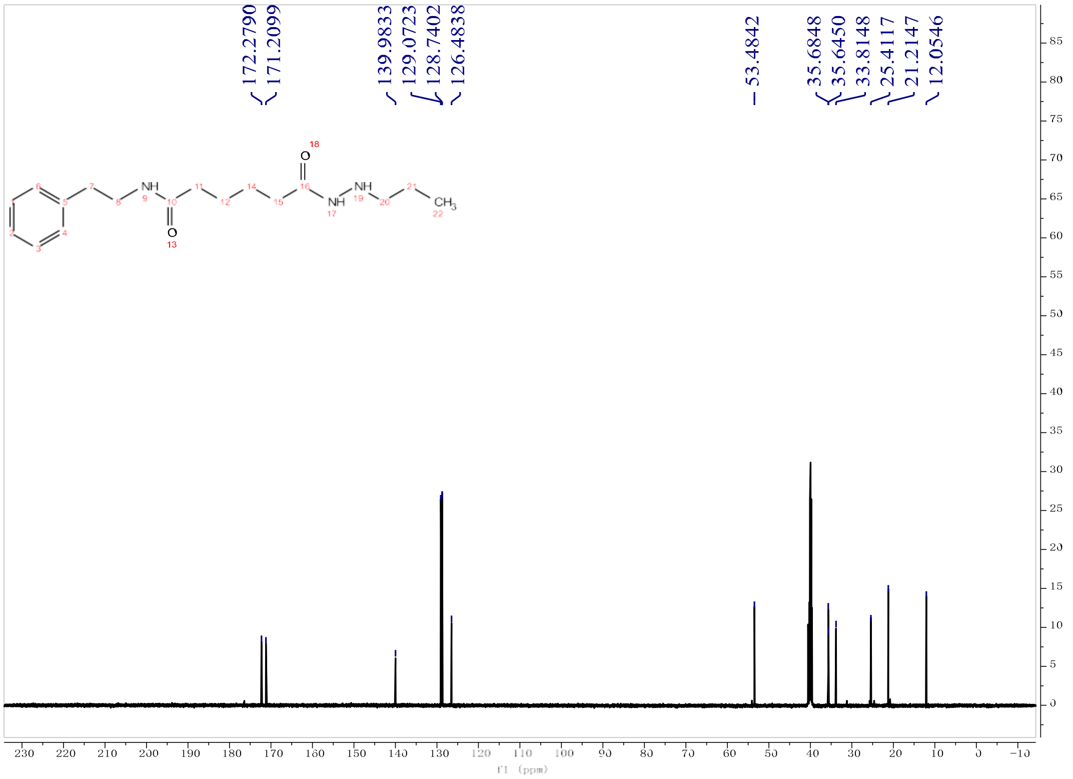

^{13}C NMR spectra for compound **9c**

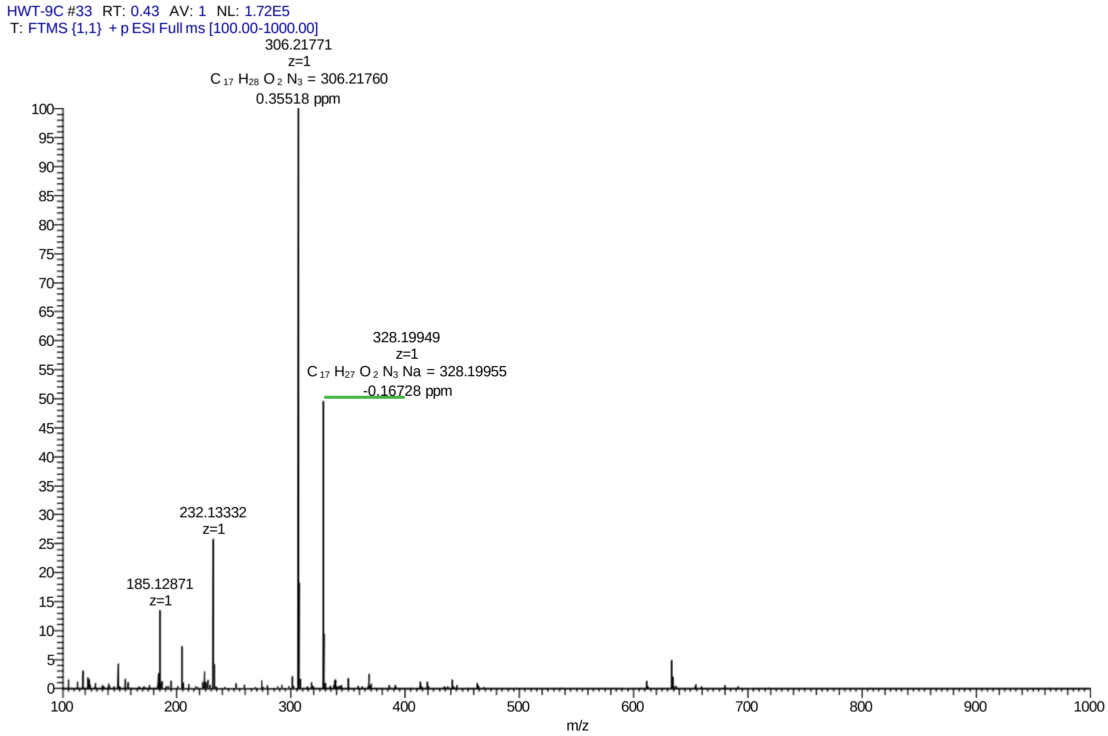

HRMS (AP-ESI) spectrum of compound **9c**

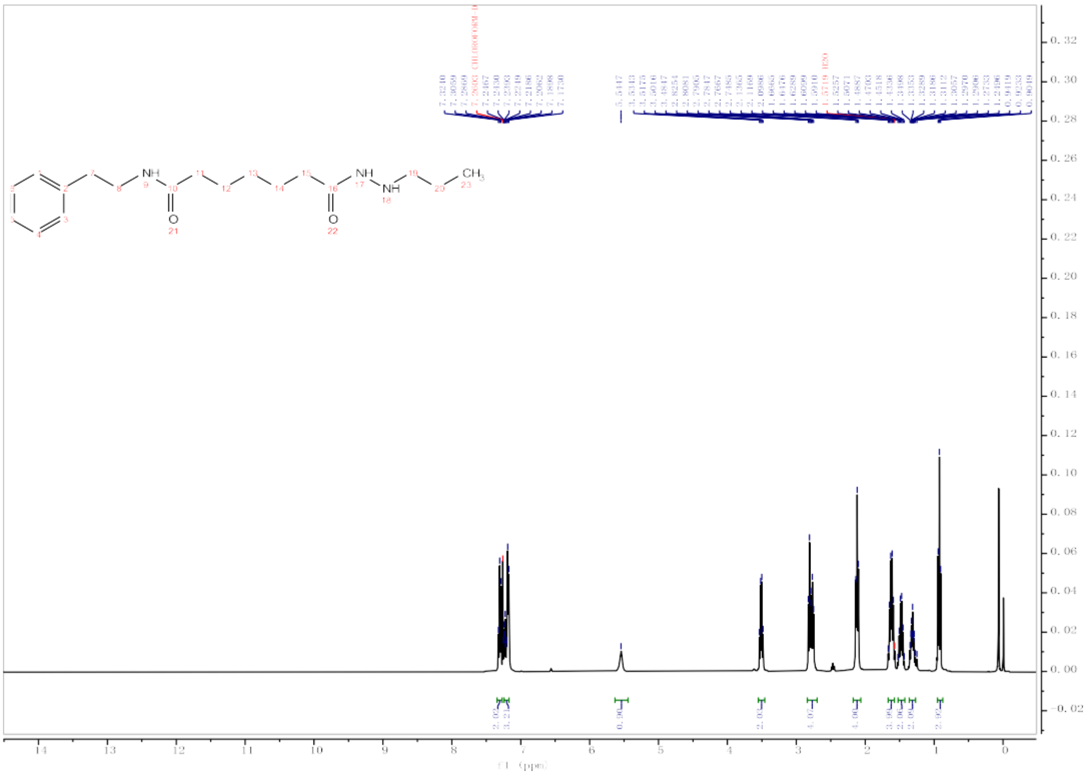

^{1}H NMR spectra for compound **9d**

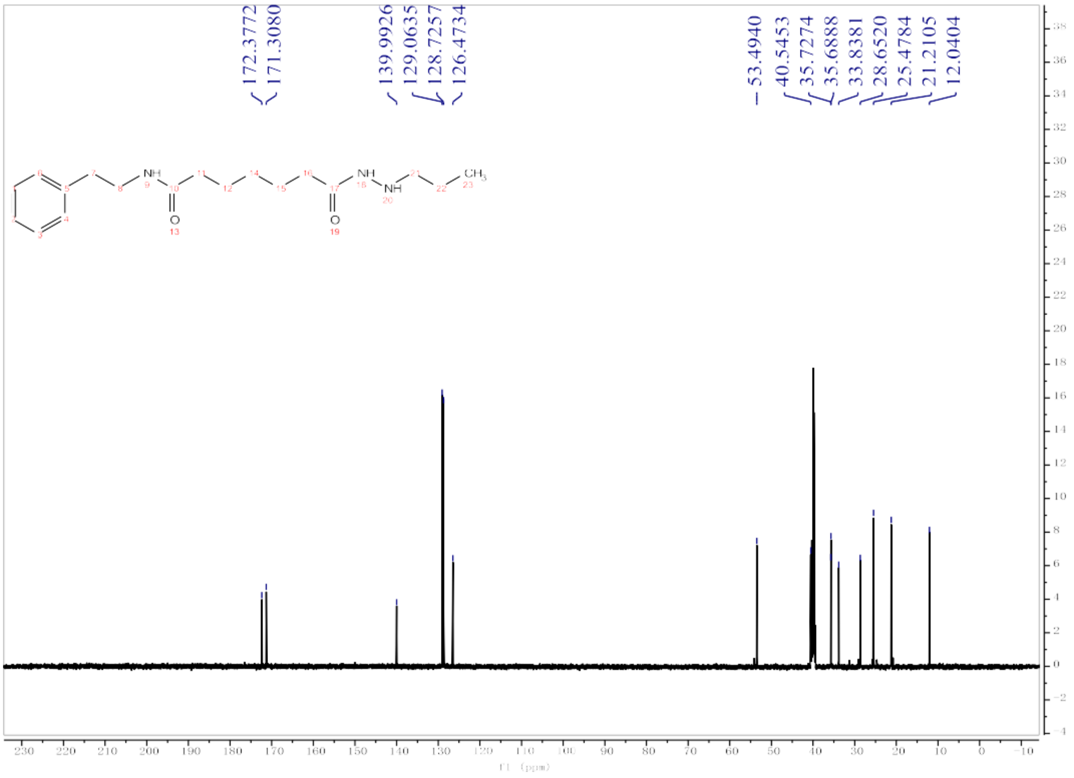

^{13}C NMR spectra for compound **9d**

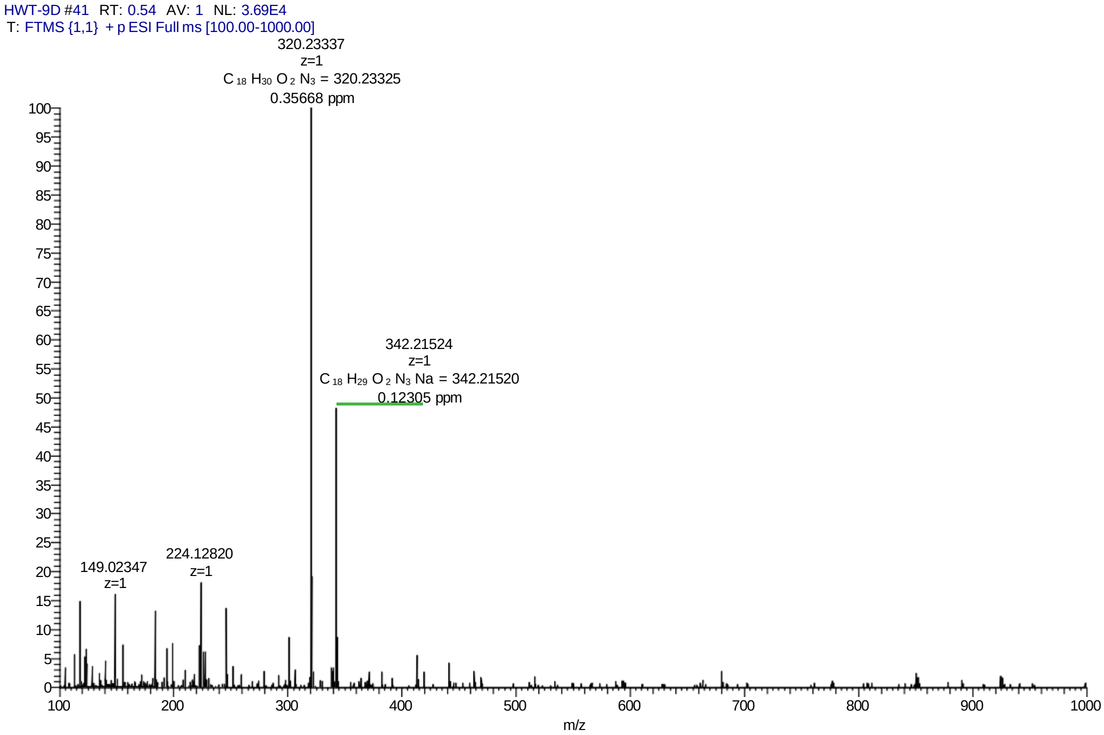

HRMS (AP-ESI) spectrum of compound **9d**

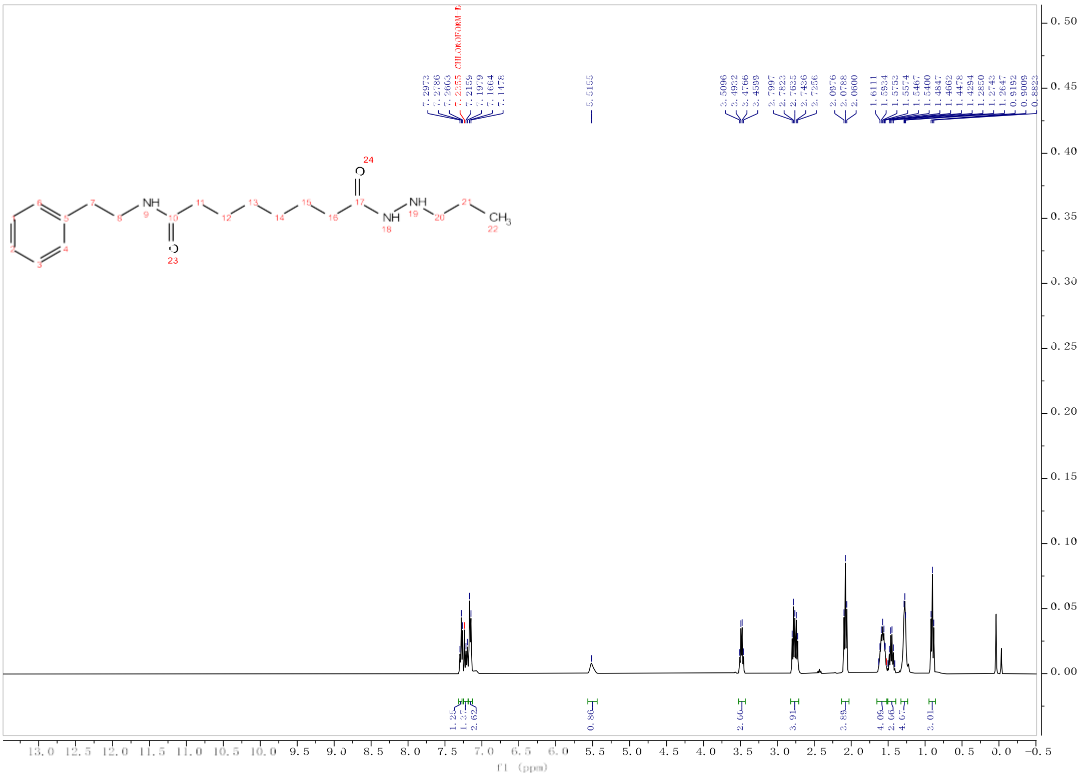

^{1}H NMR spectra for compound **9e**

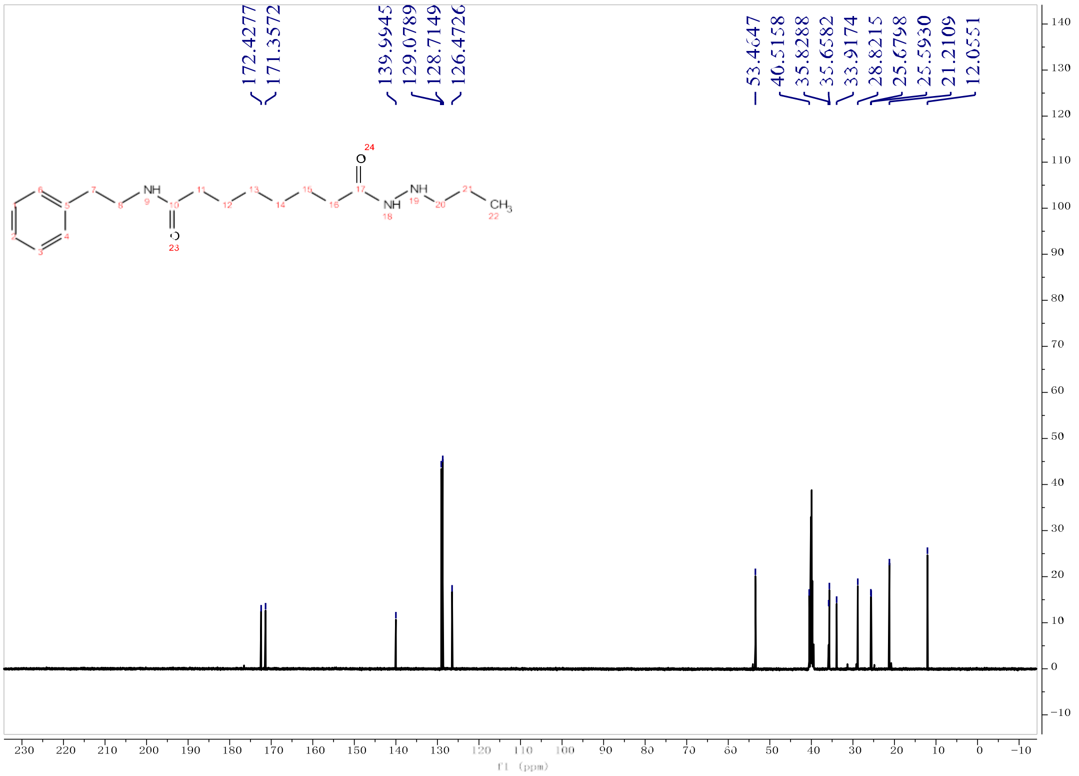

^{13}C NMR spectra for compound **9e**

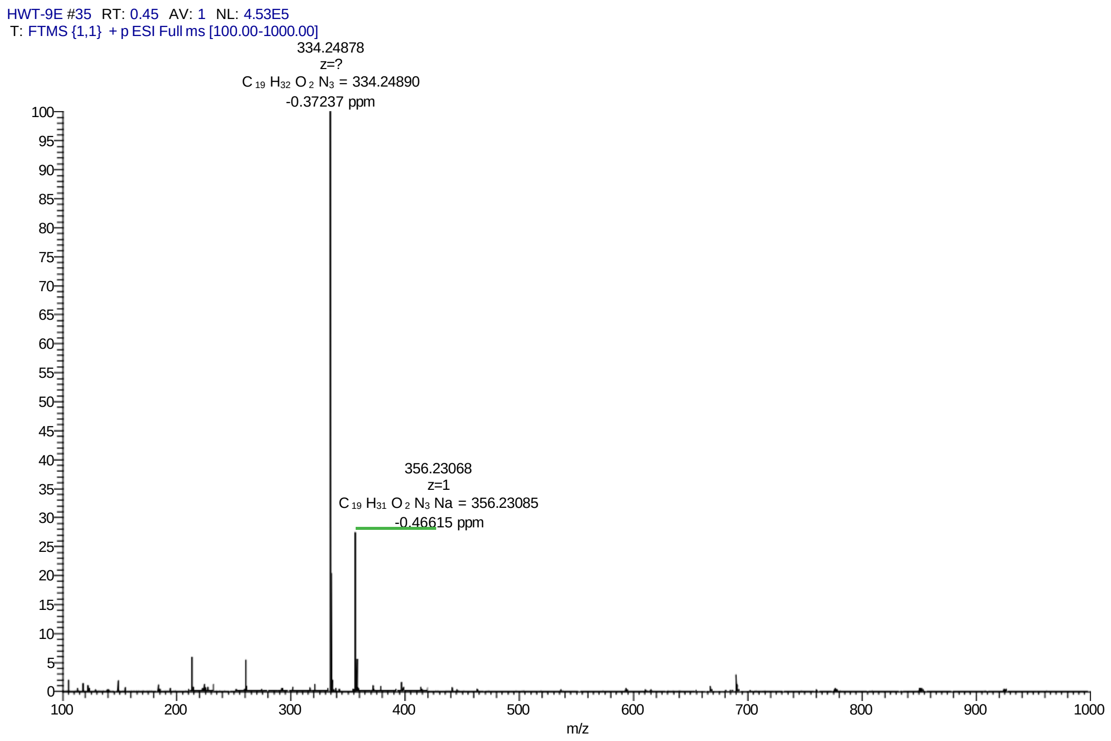

HRMS (AP-ESI) spectrum of compound **9e**

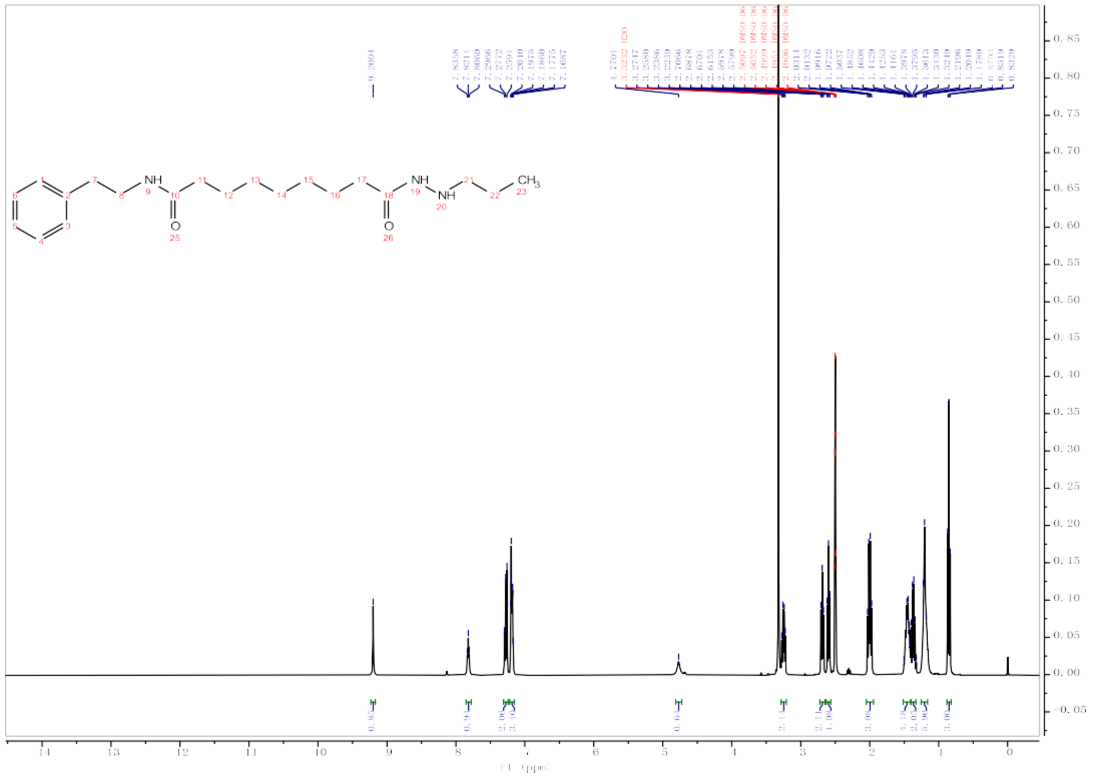

^{1}H NMR spectra for compound **9f**

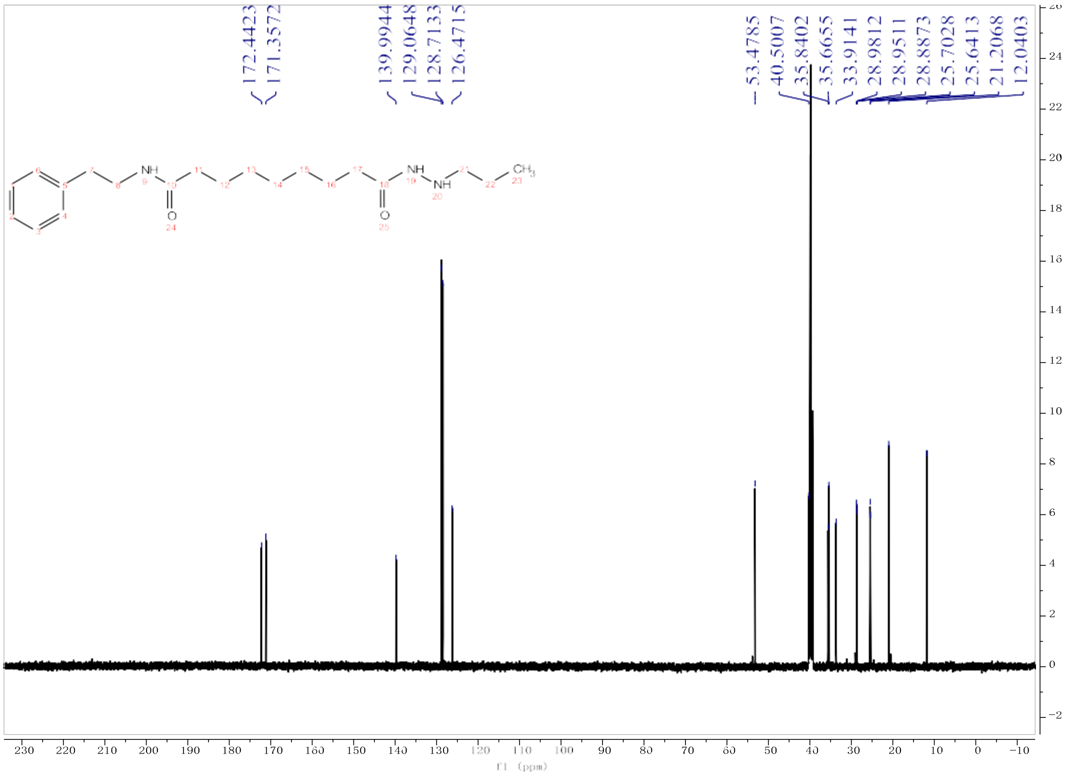

^{13}C NMR spectra for compound **9f**

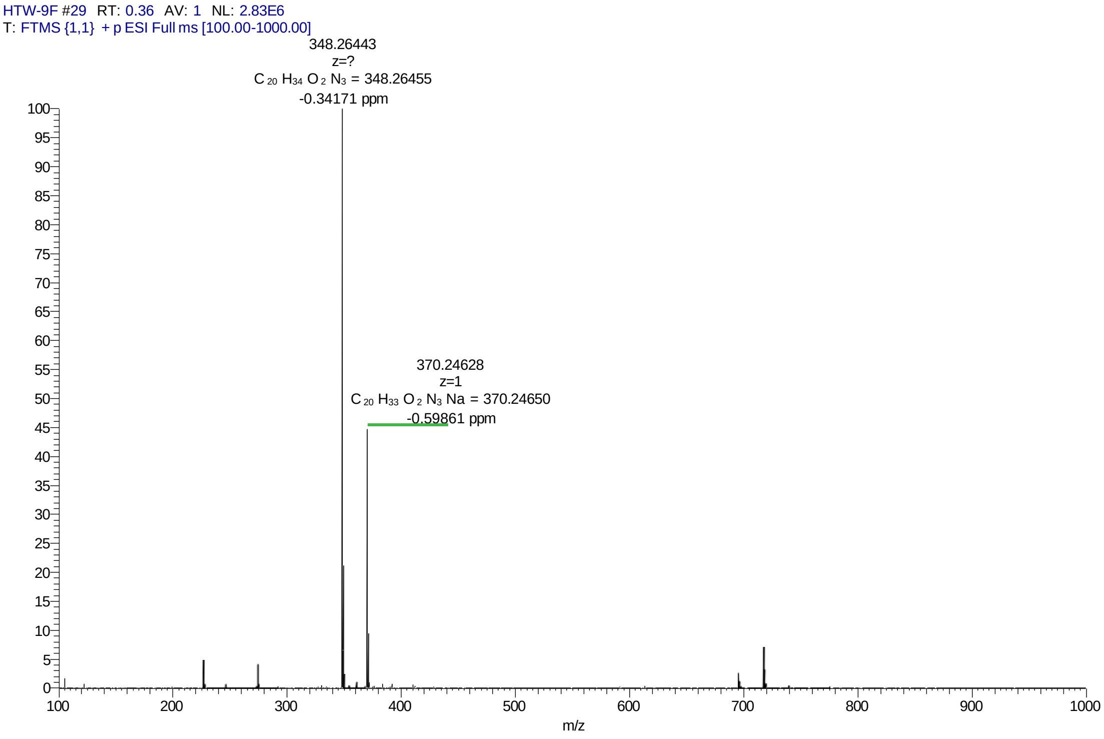

HRMS (AP-ESI) spectrum of compound **9f**

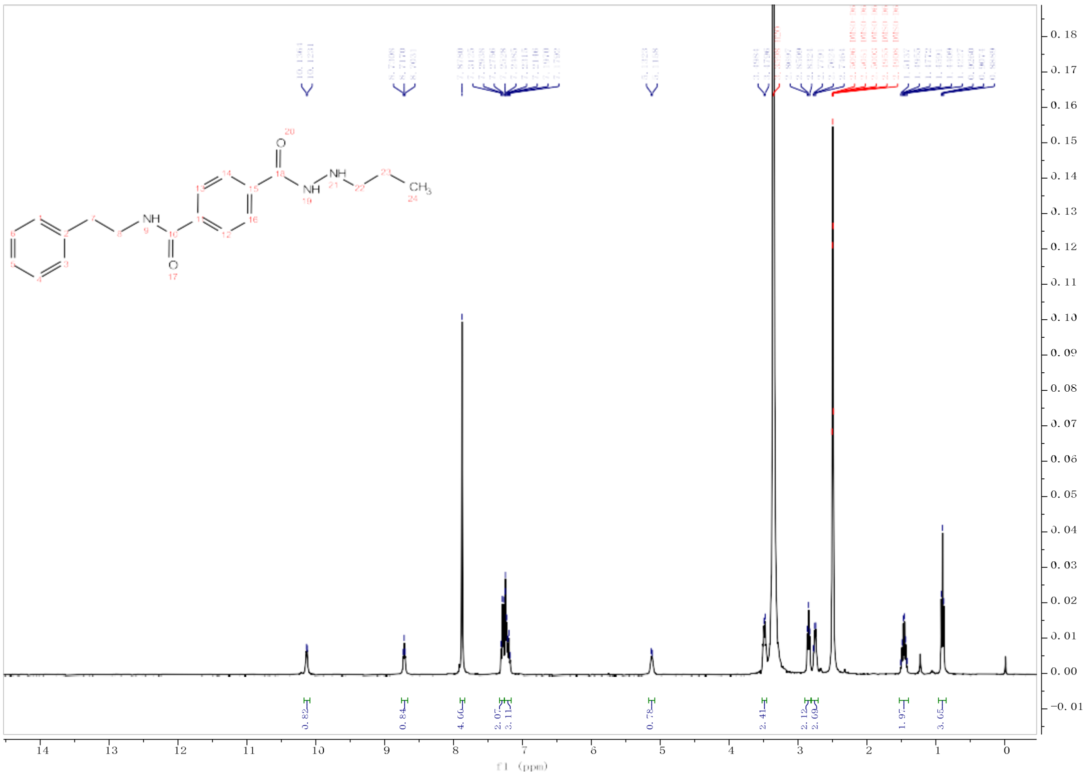

^{1}H NMR spectra for compound **9g**

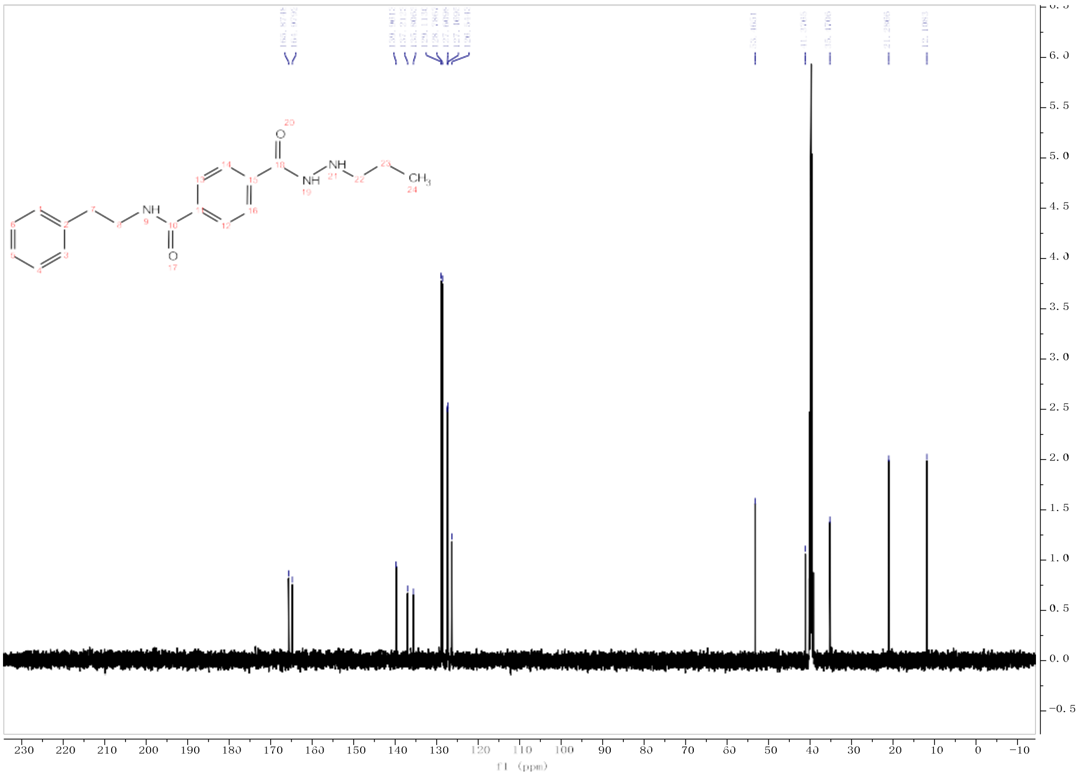

^{13}C NMR spectra for compound **9g**

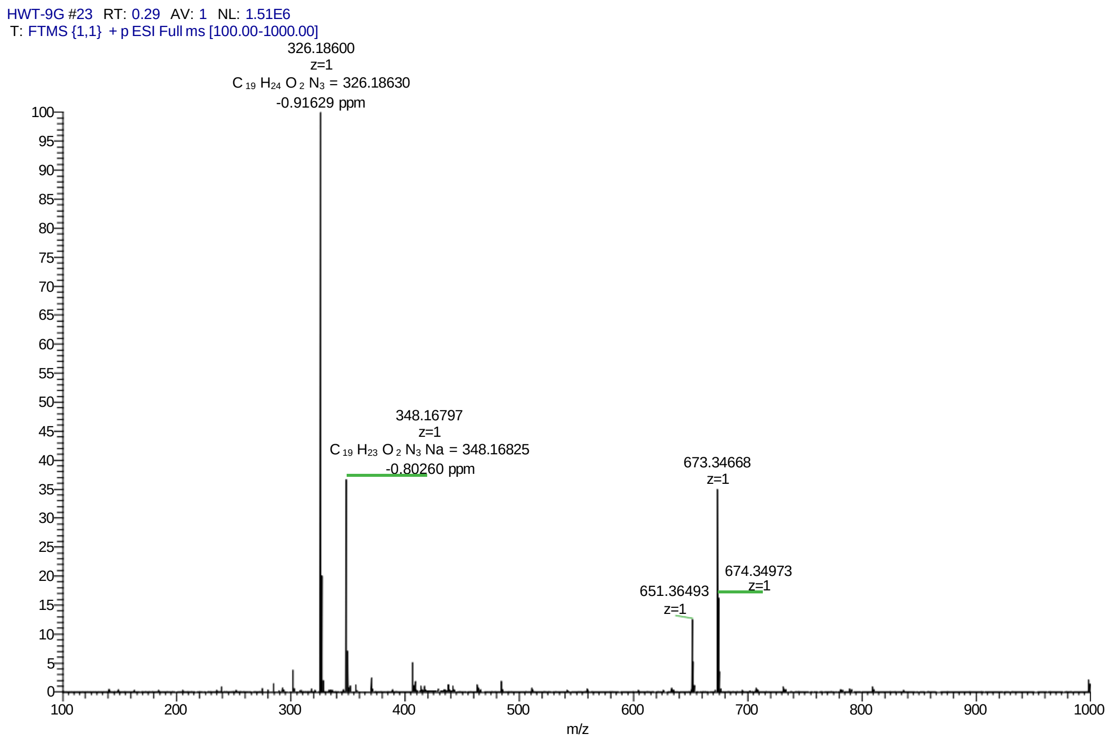

HRMS (AP-ESI) spectrum of compound **9g**

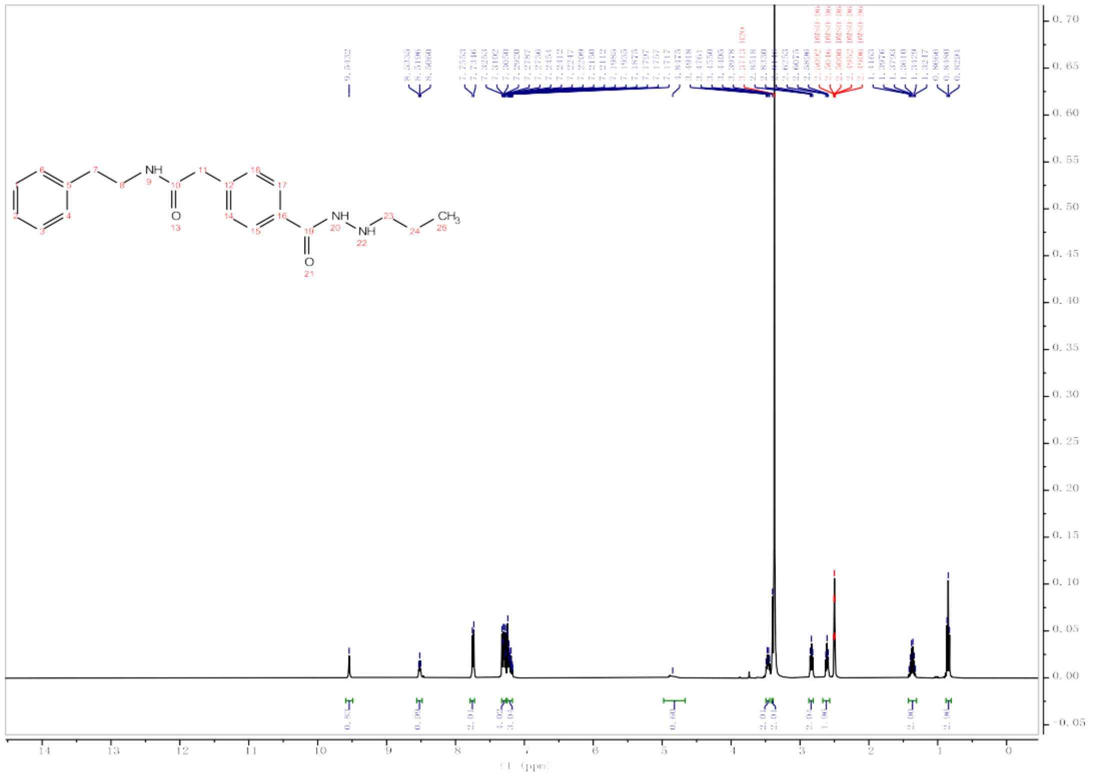

^{1}H NMR spectra for compound **9h**

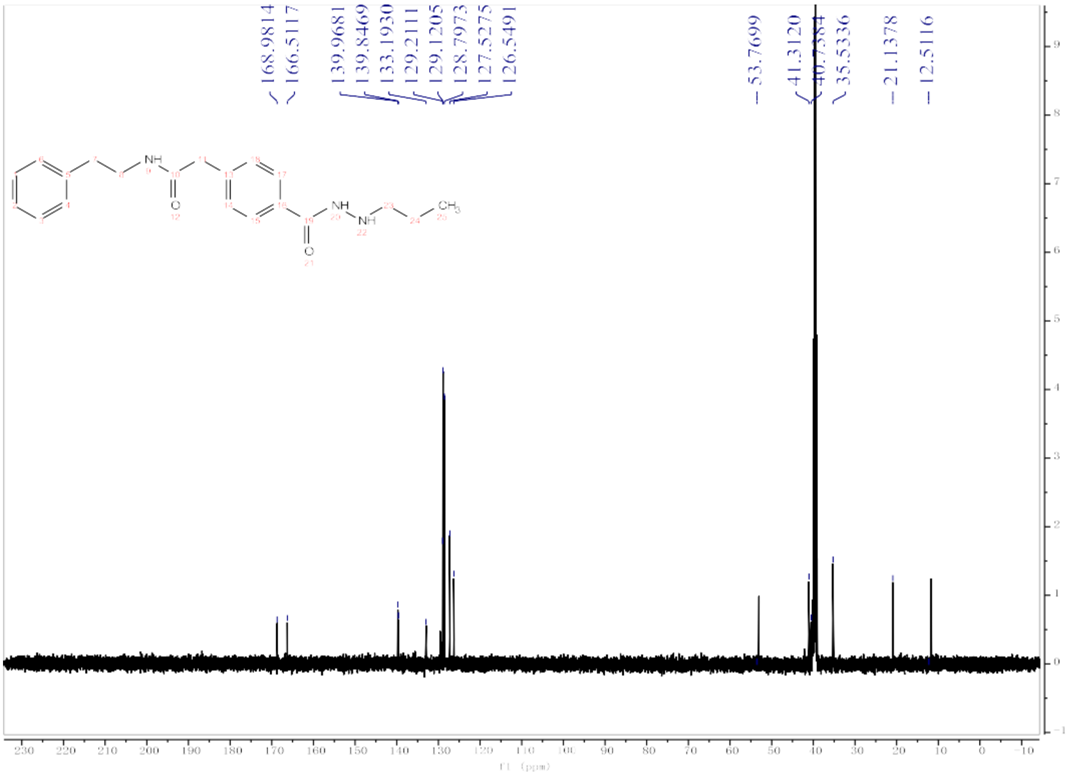

^{13}C NMR spectra for compound **9h**

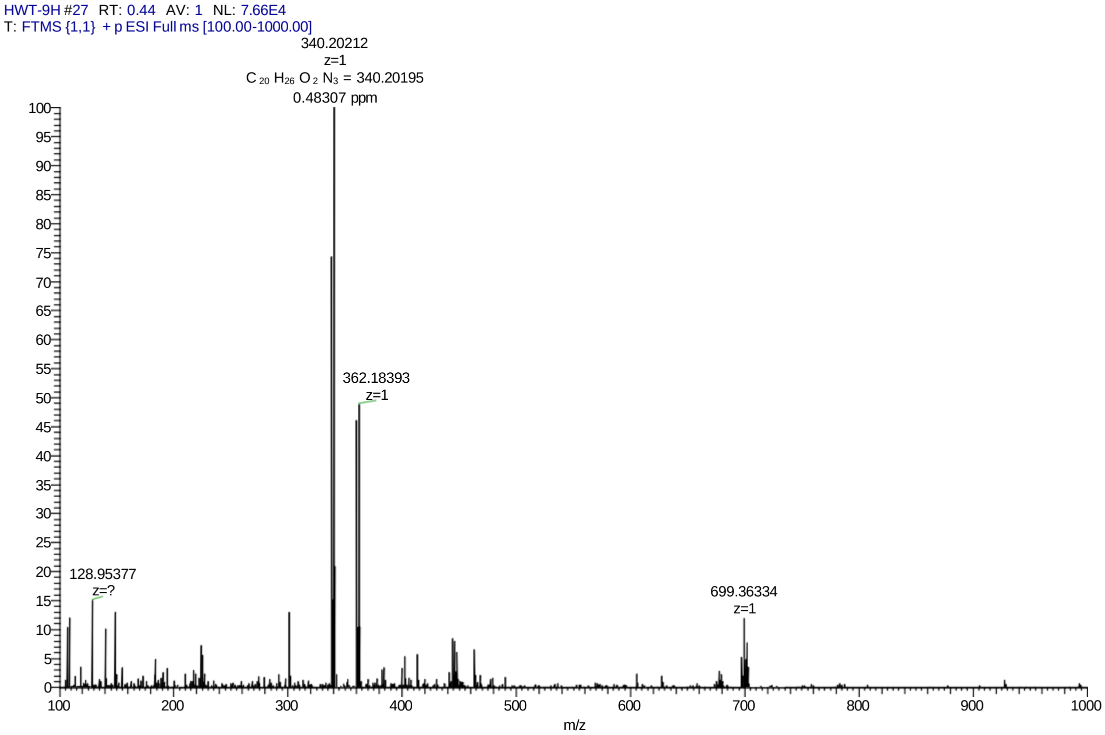

HRMS (AP-ESI) spectrum of compound **9h**

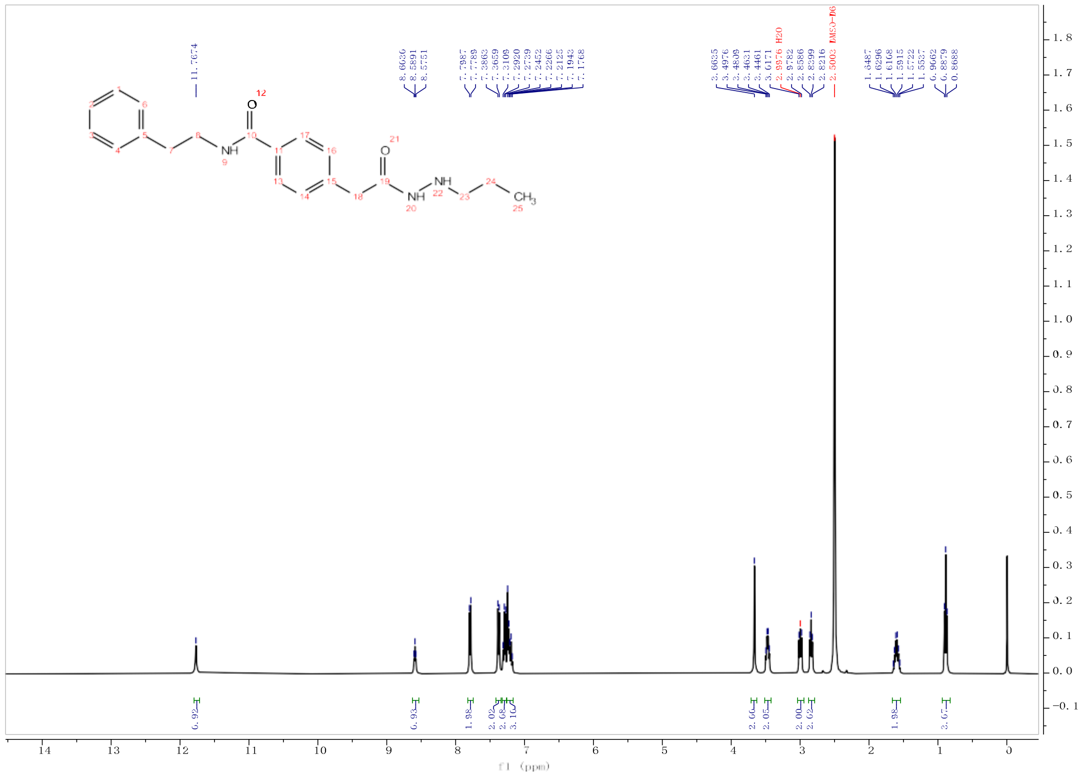

^{1}H NMR spectra for compound **9i**

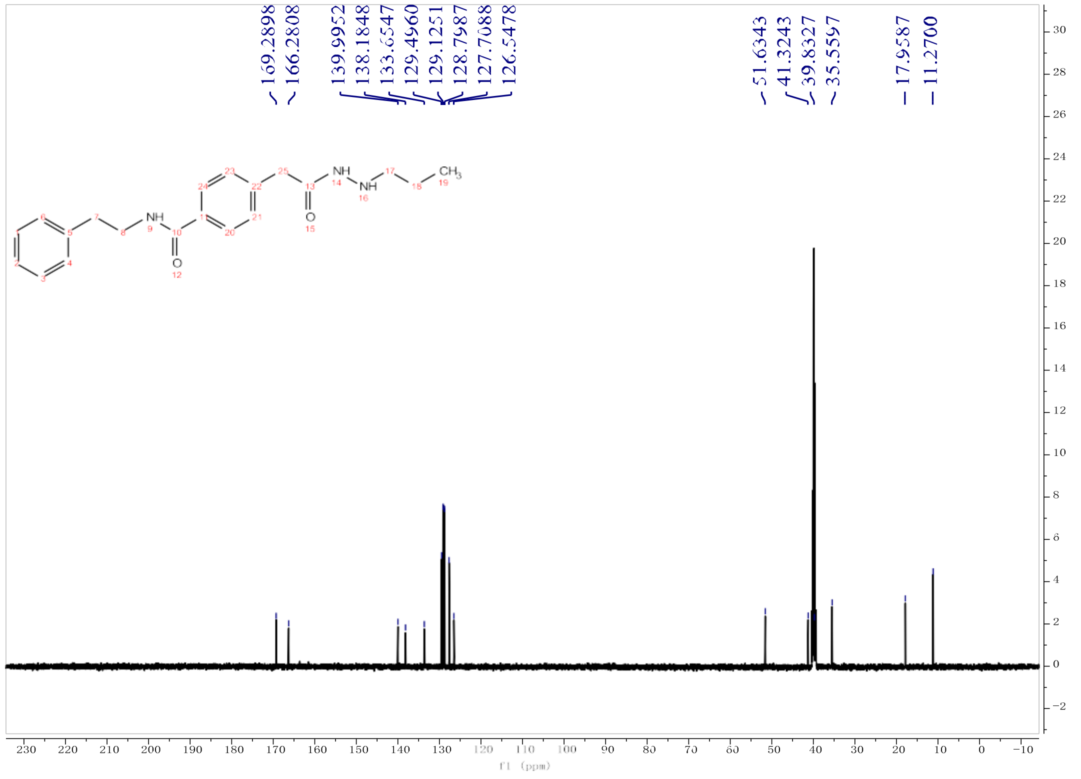

^{13}C NMR spectra for compound **9i**

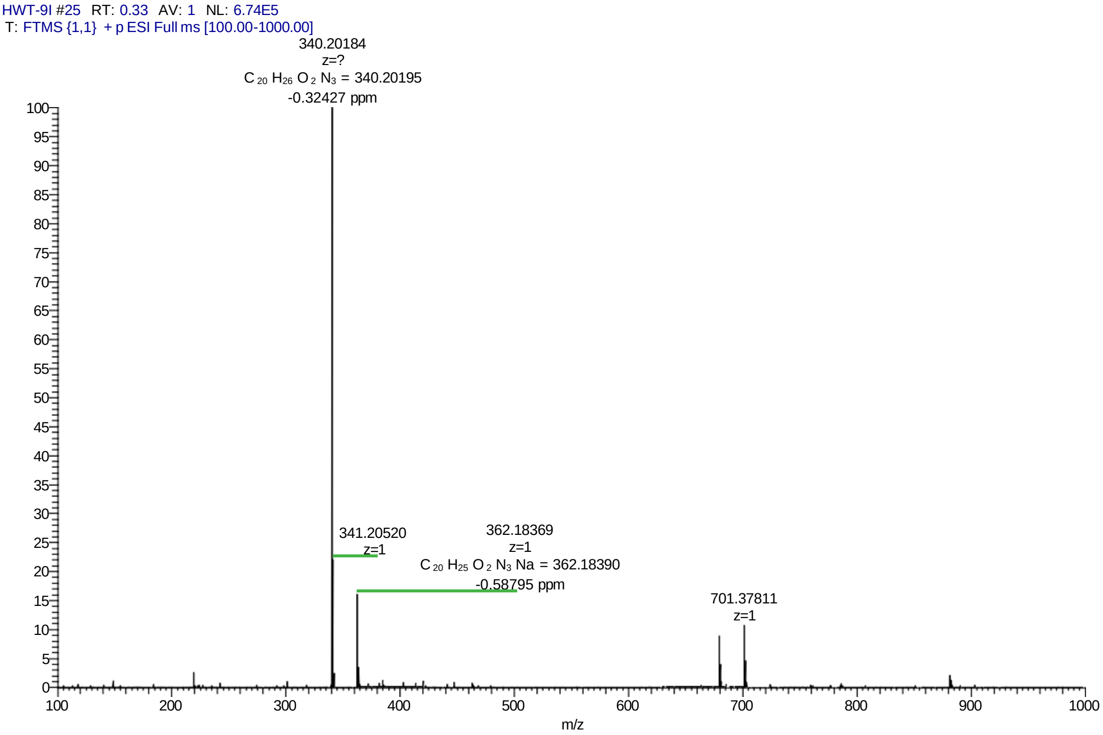

HRMS (AP-ESI) spectrum of compound **9i**

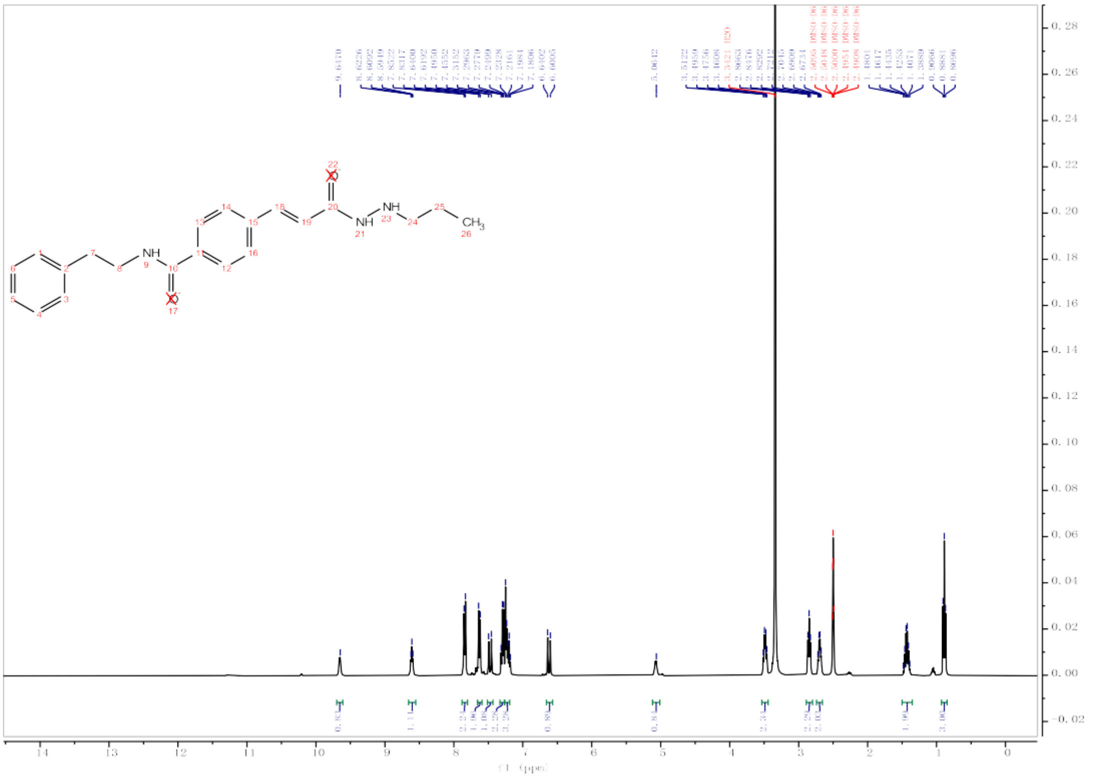

^{1}H NMR spectra for compound **9j**

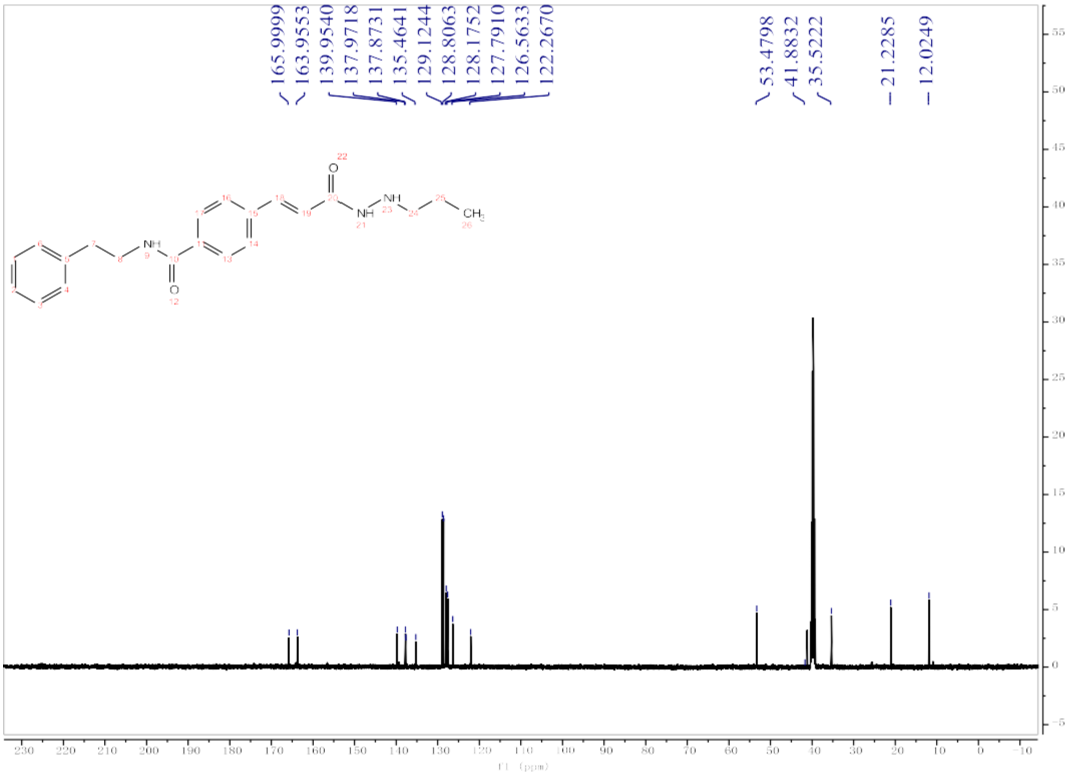

^{13}C NMR spectra for compound **9j**

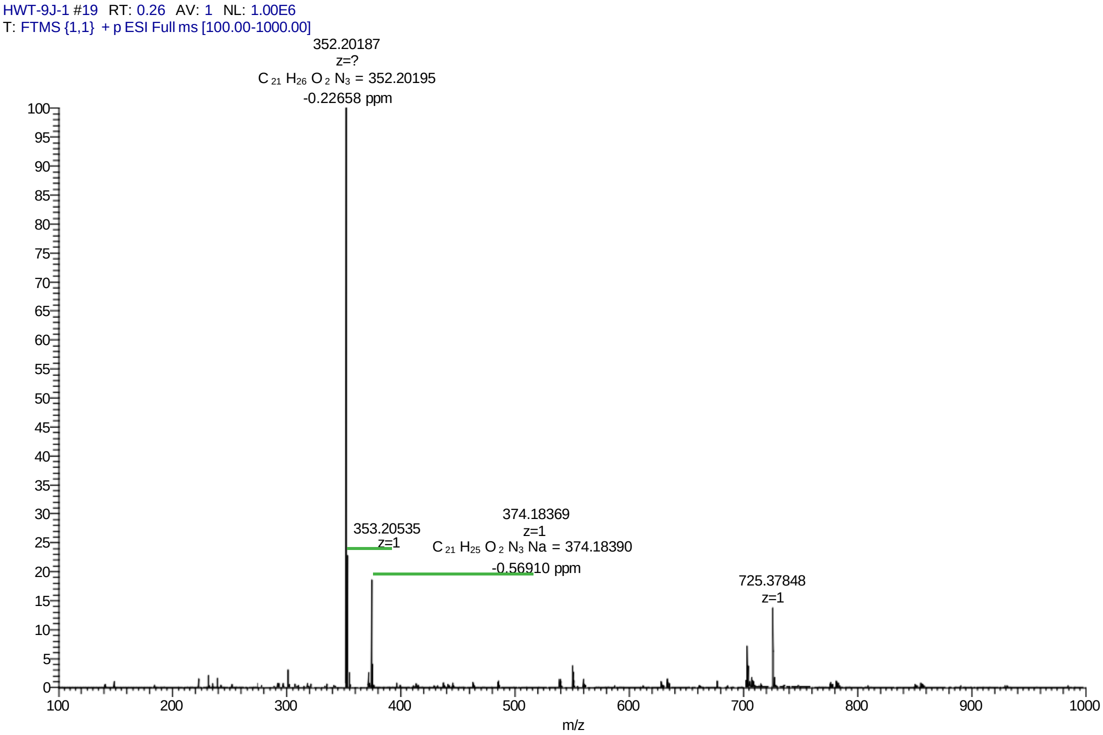

HRMS (AP-ESI) spectrum of compound **9j**

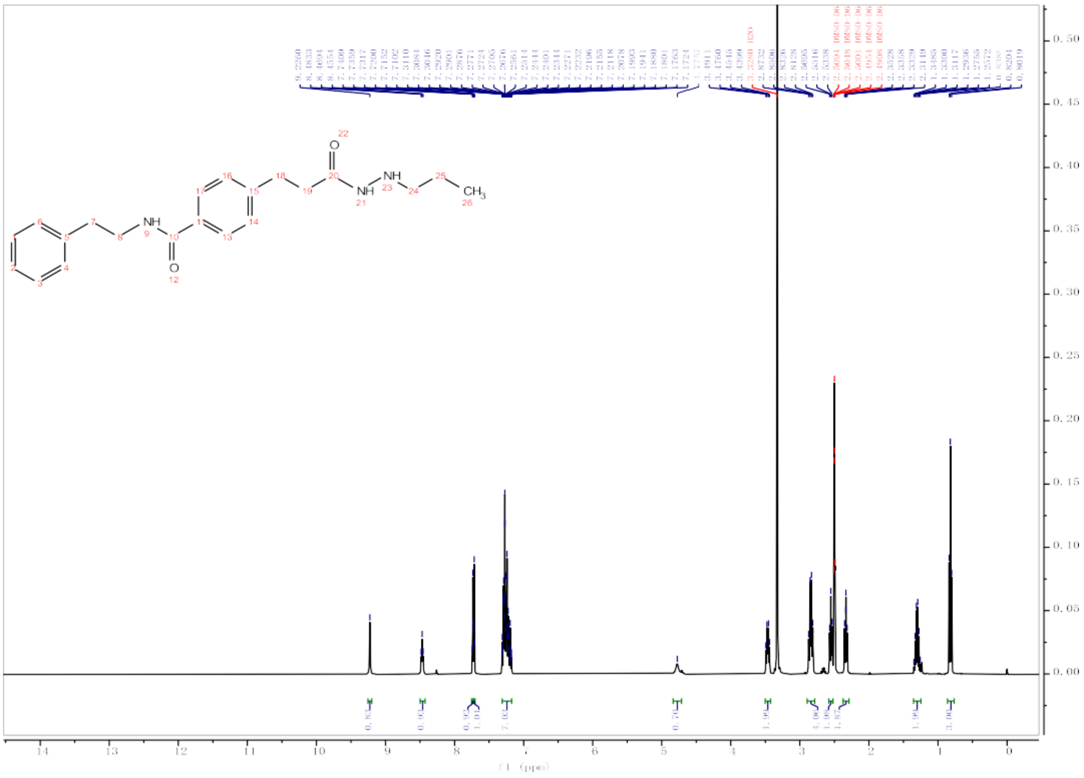

^{1}H NMR spectra for compound **9k**

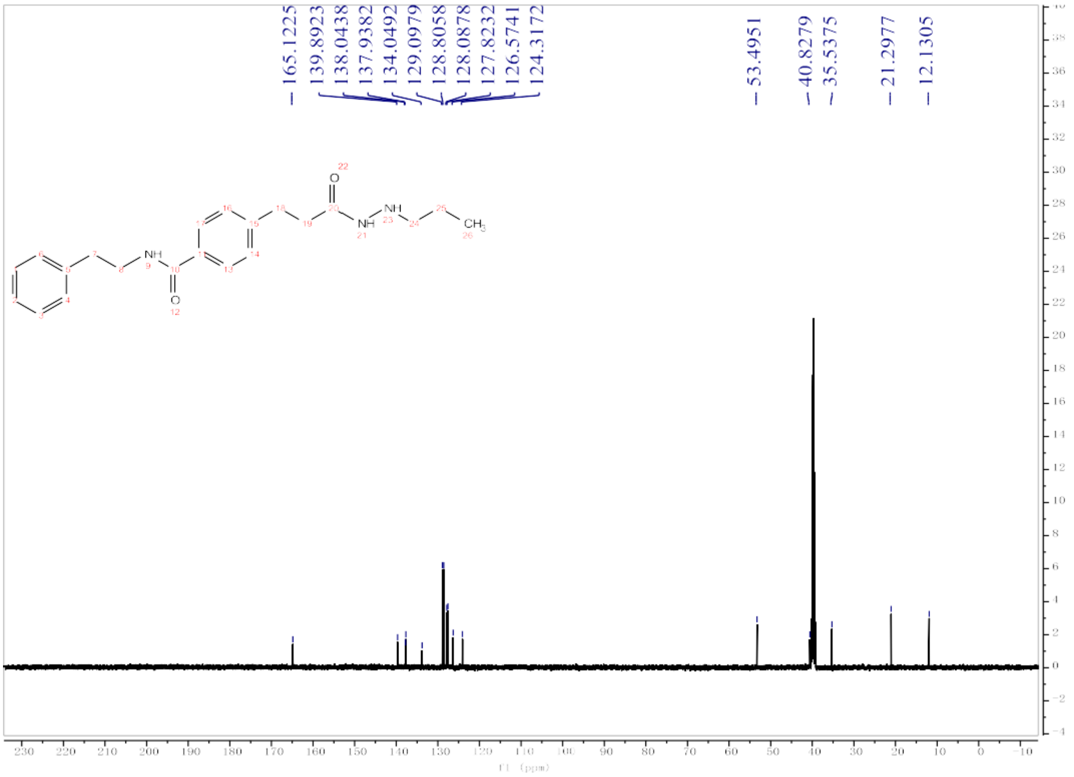

^{13}C NMR spectra for compound **9k**

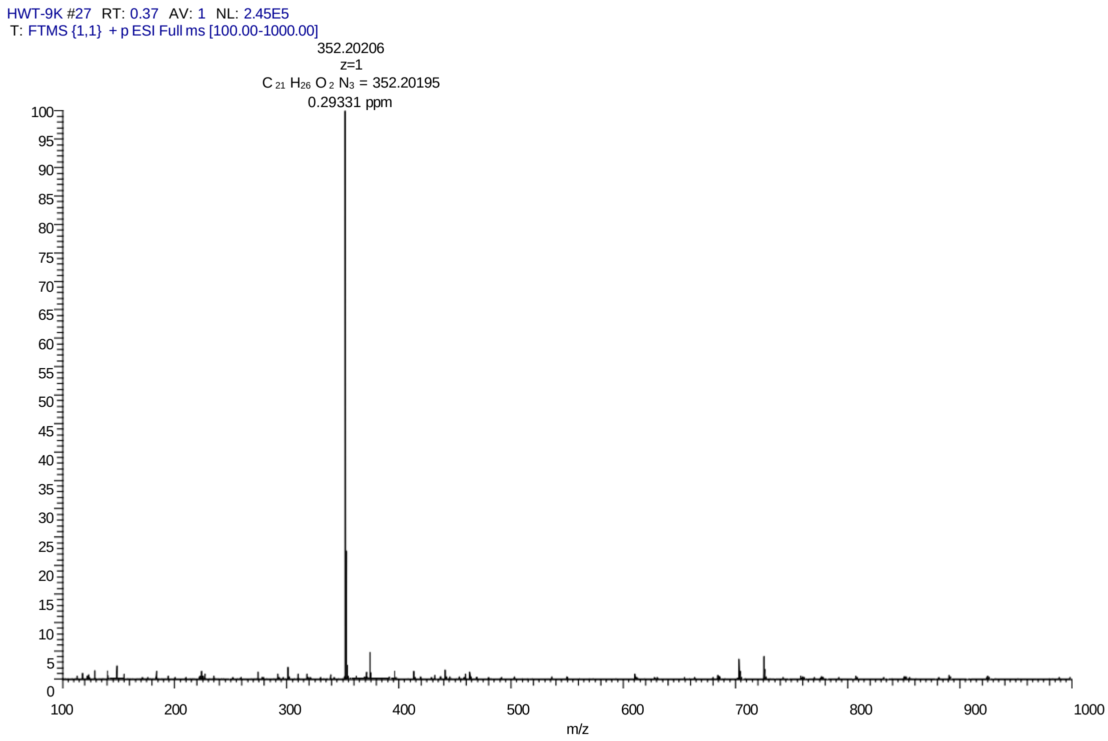

HRMS (AP-ESI) spectrum of compound **9k**

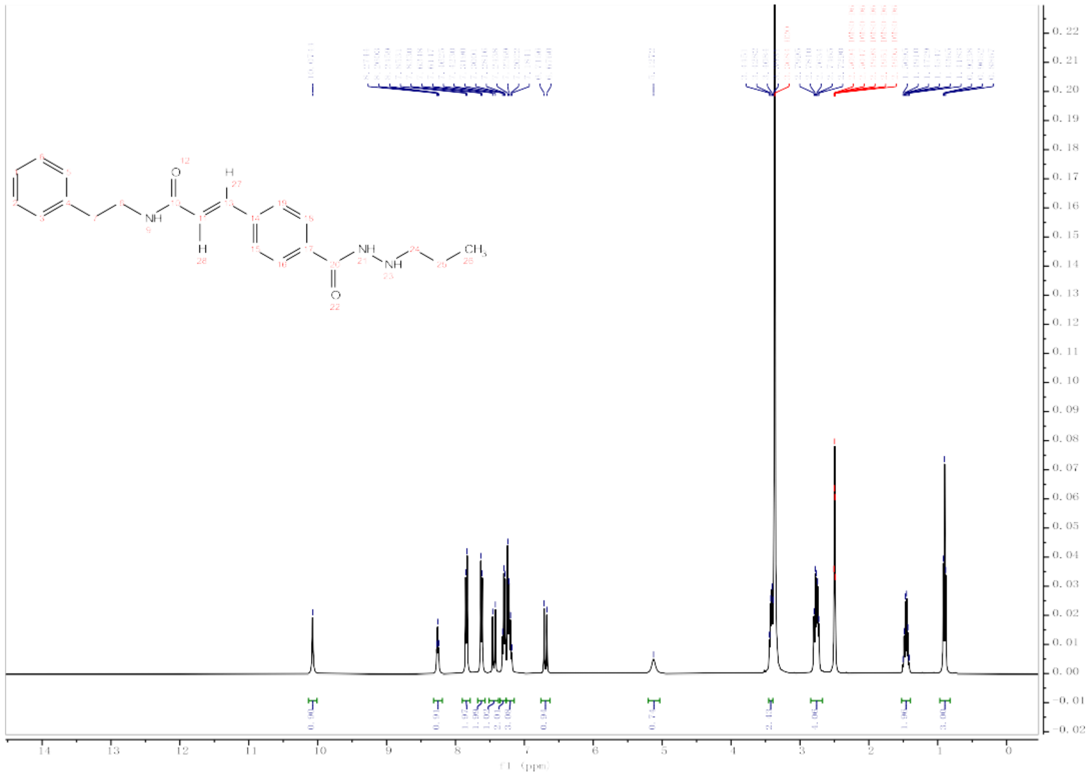

^{1}H NMR spectra for compound **9l**

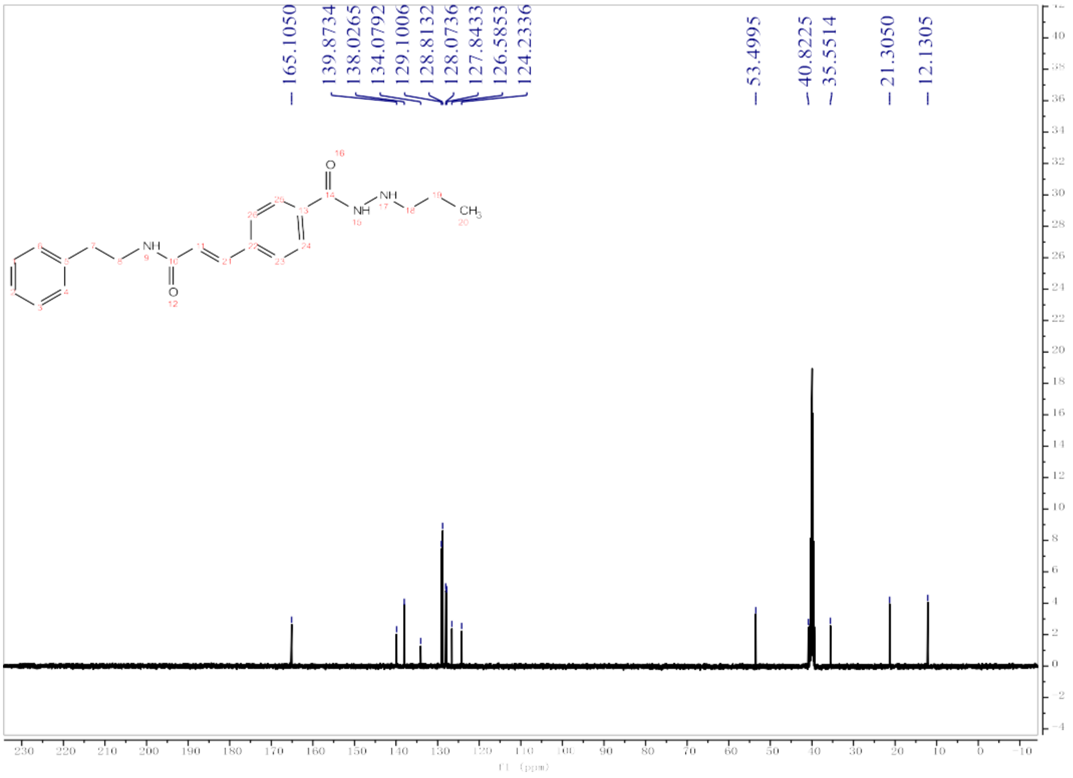

^{13}C NMR spectra for compound **9l**

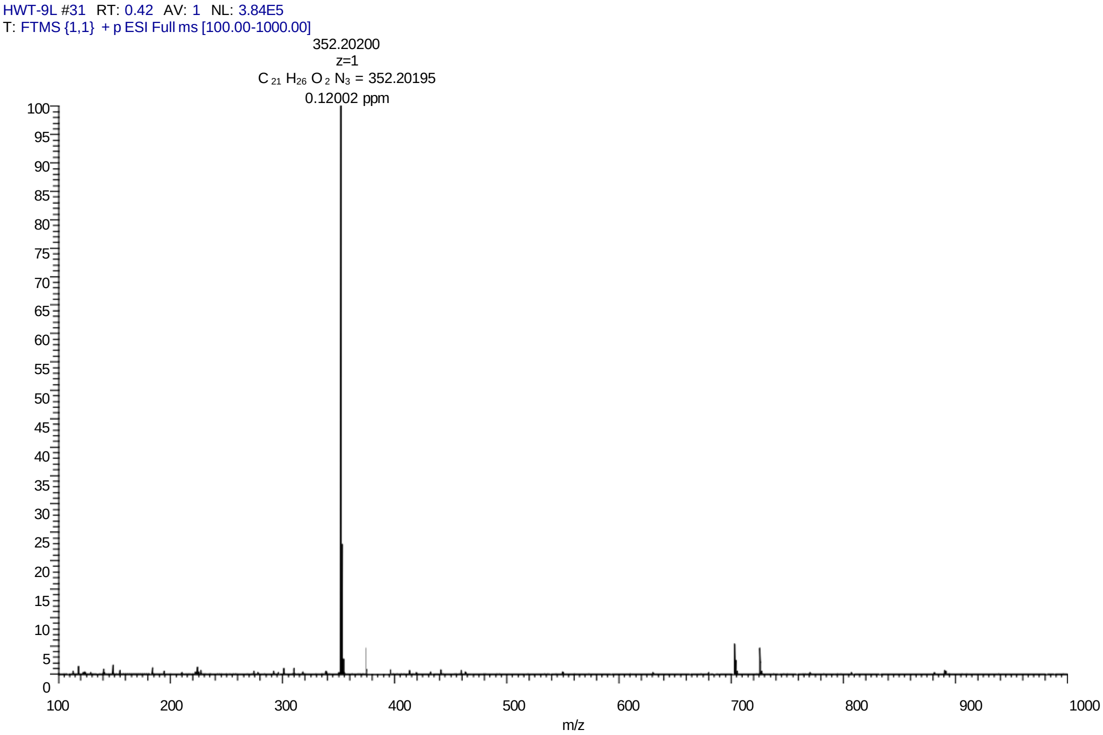

HRMS (AP-ESI) spectrum of compound **9l**

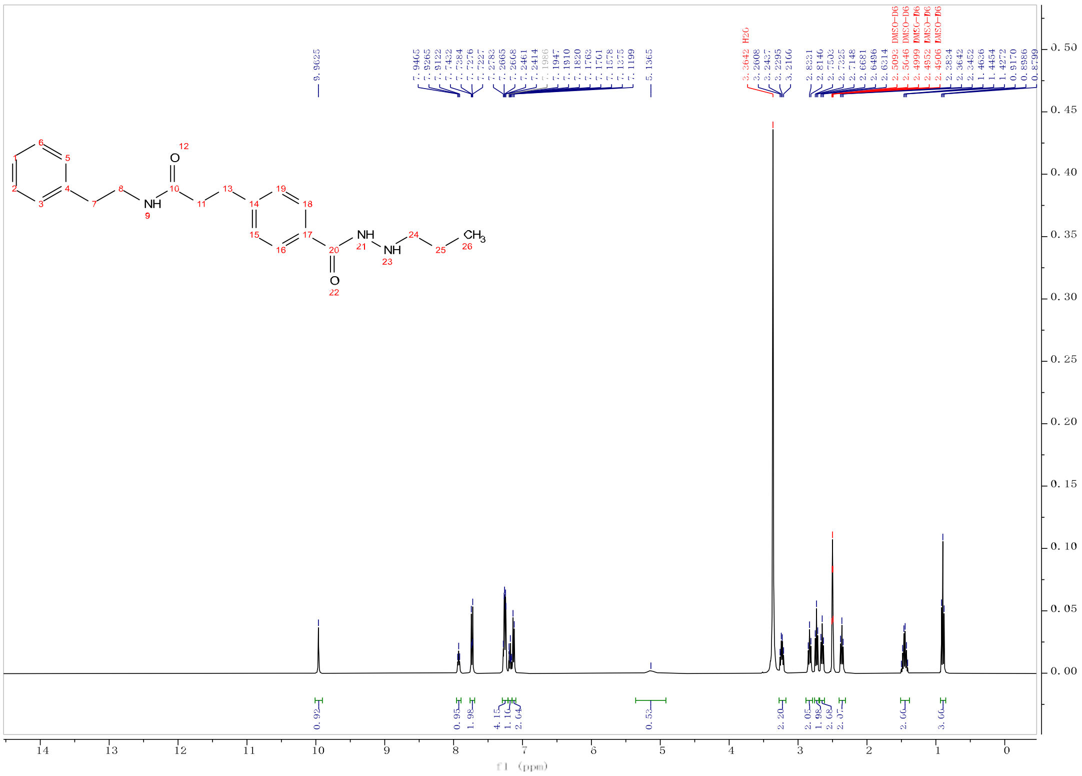

^{1}H NMR spectra for compound **9m**

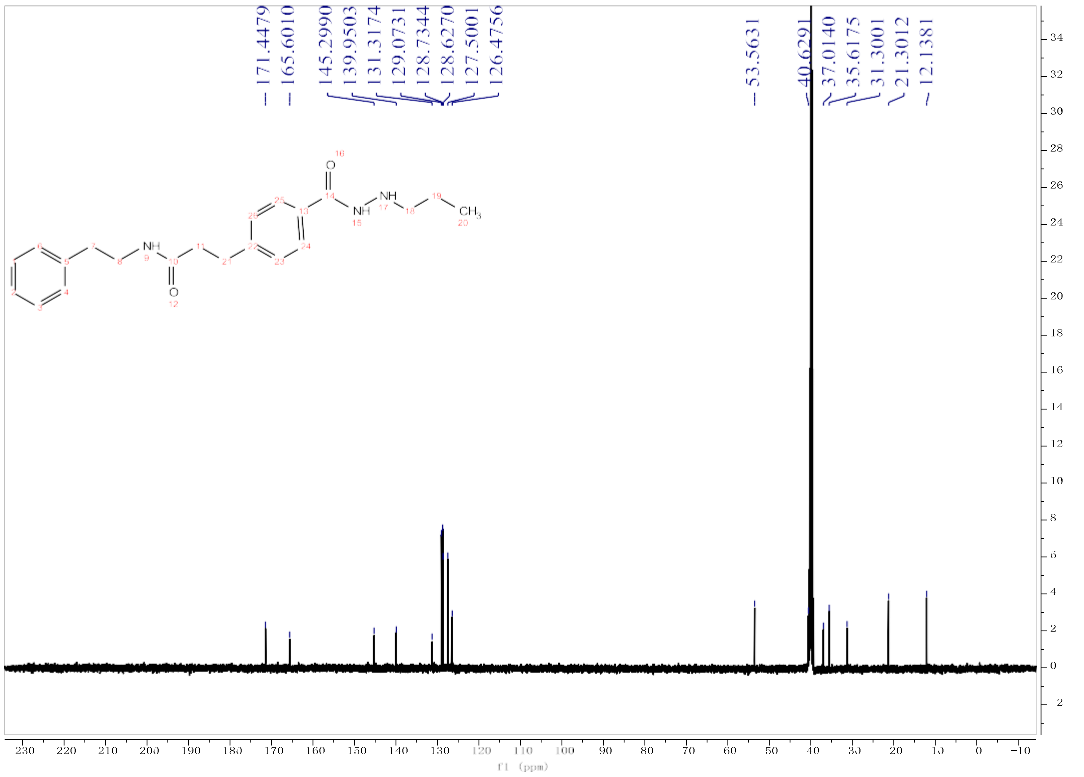

^{13}C NMR spectra for compound **9m**

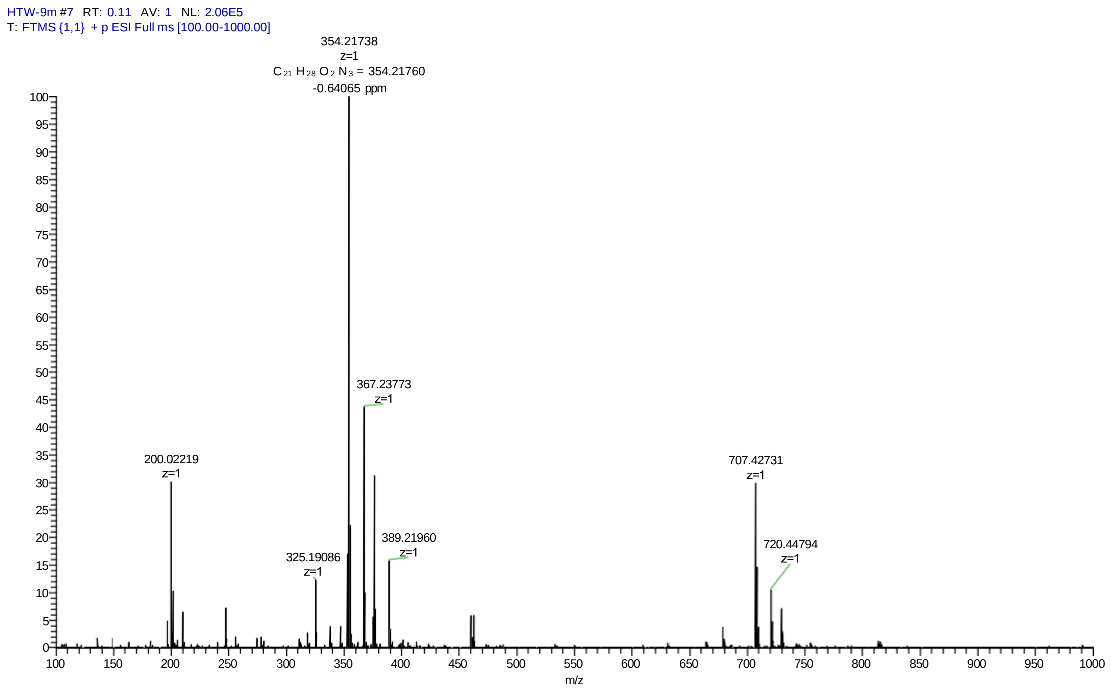

HRMS (AP-ESI) spectrum of compound **9m**

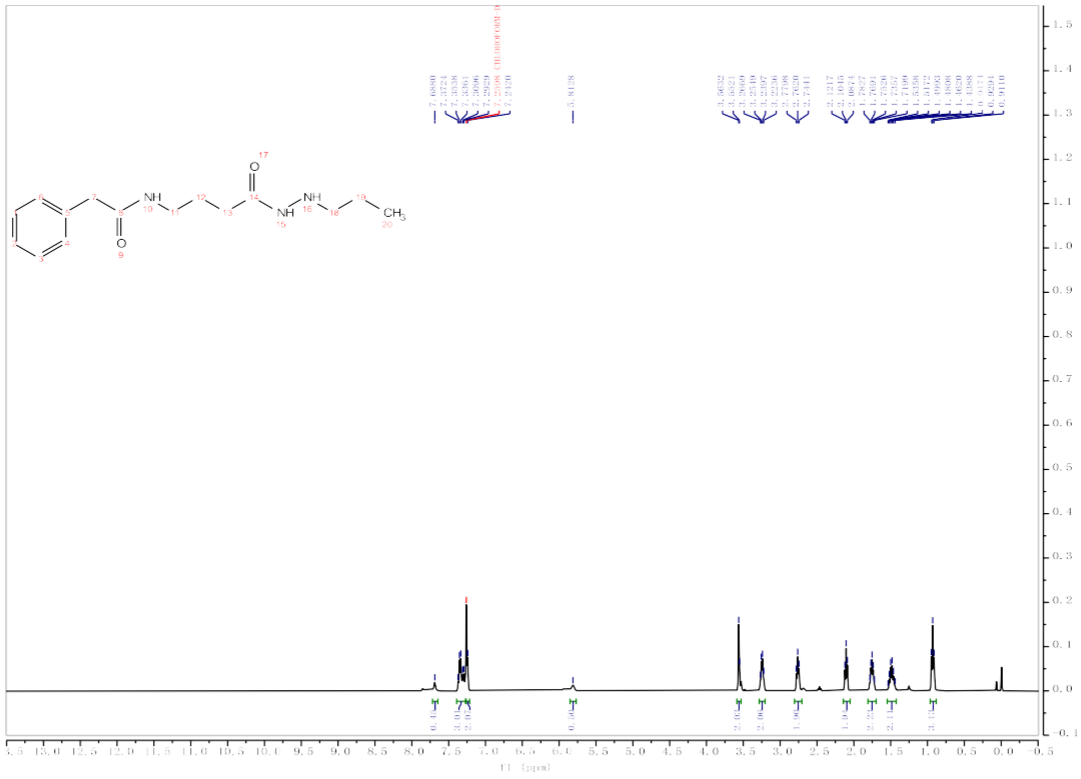

^{1}H NMR spectra for compound **14a**

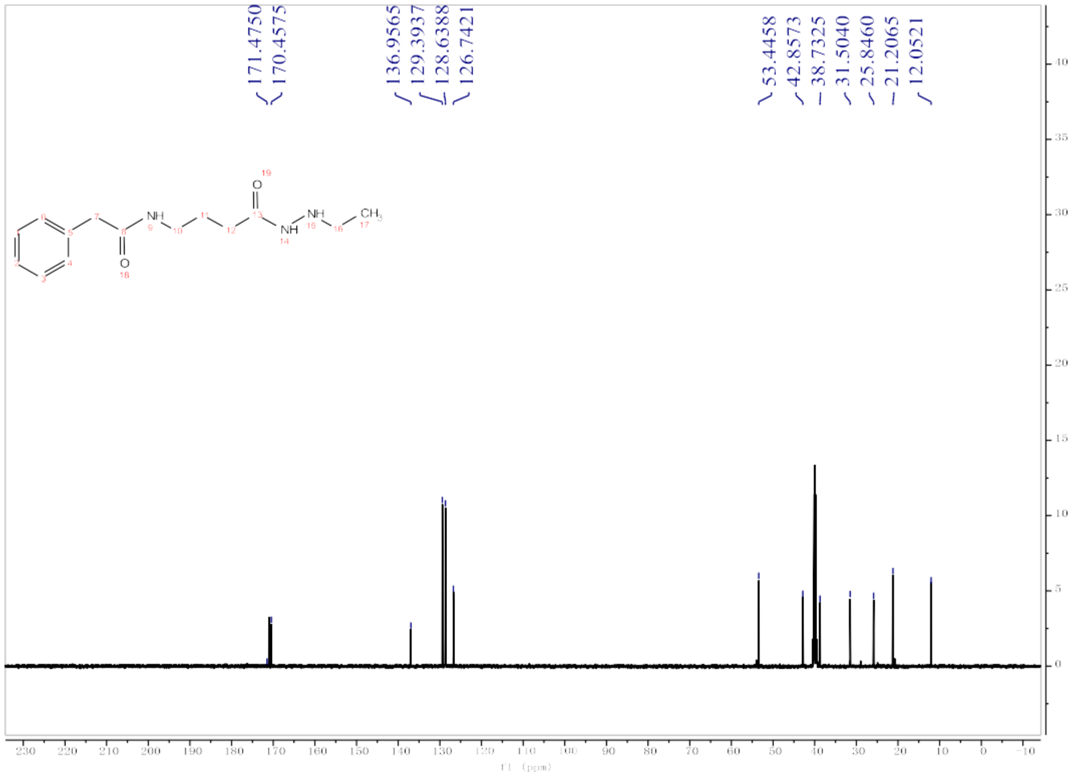

^{13}C NMR spectra for compound **14a**

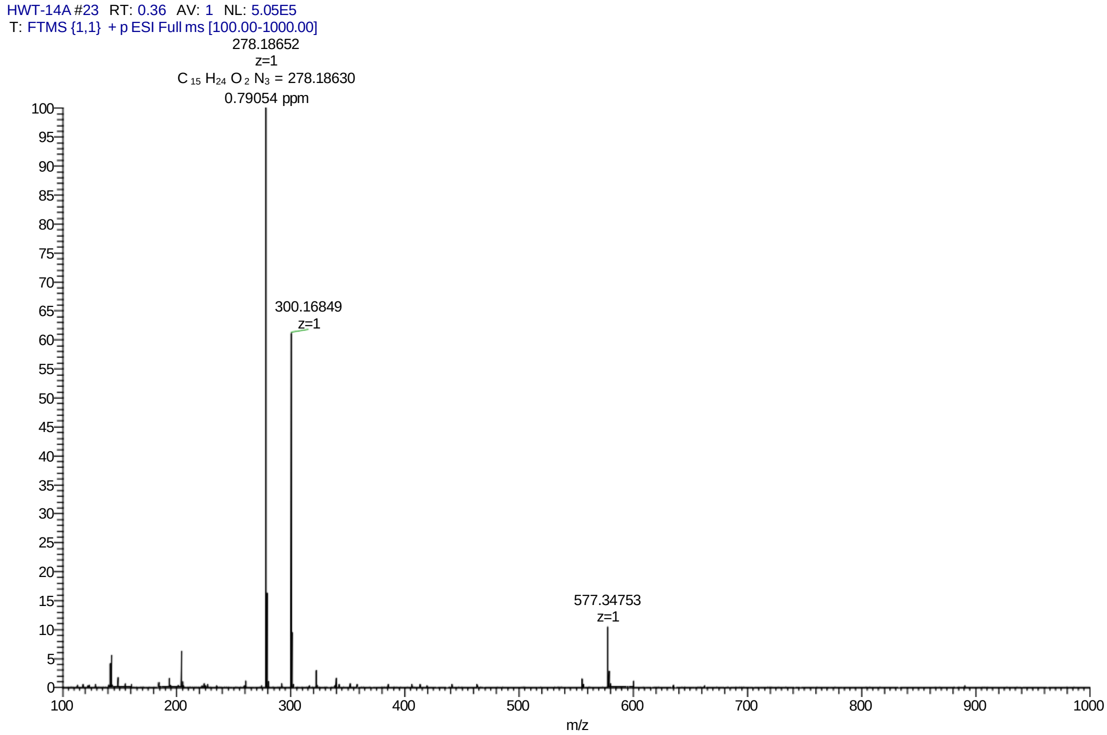

HRMS (AP-ESI) spectrum of compound **14a**

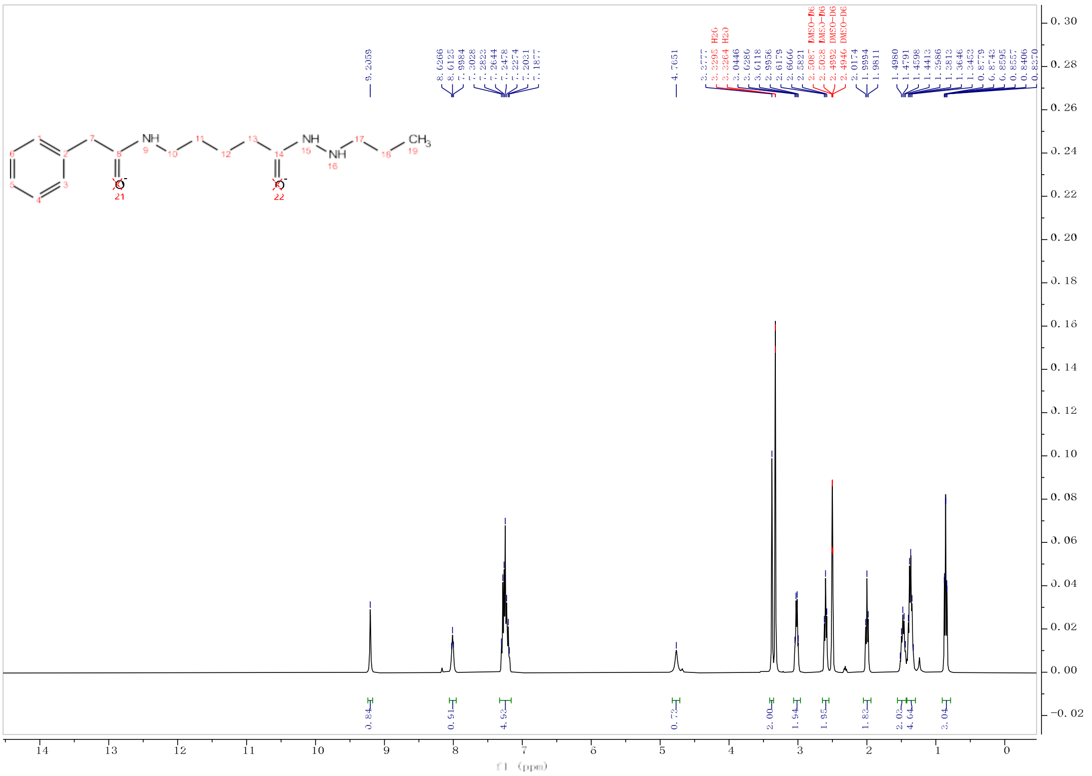

¹H NMR spectra for compound **14b**

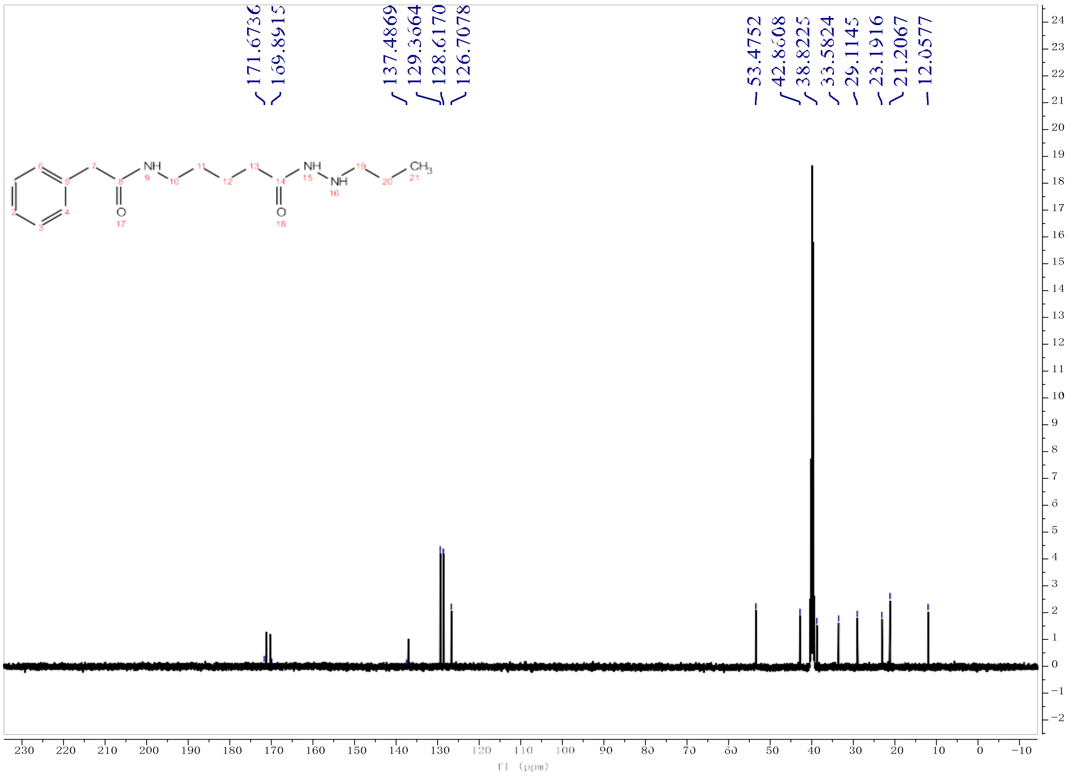

¹³C NMR spectra for compound **14b**

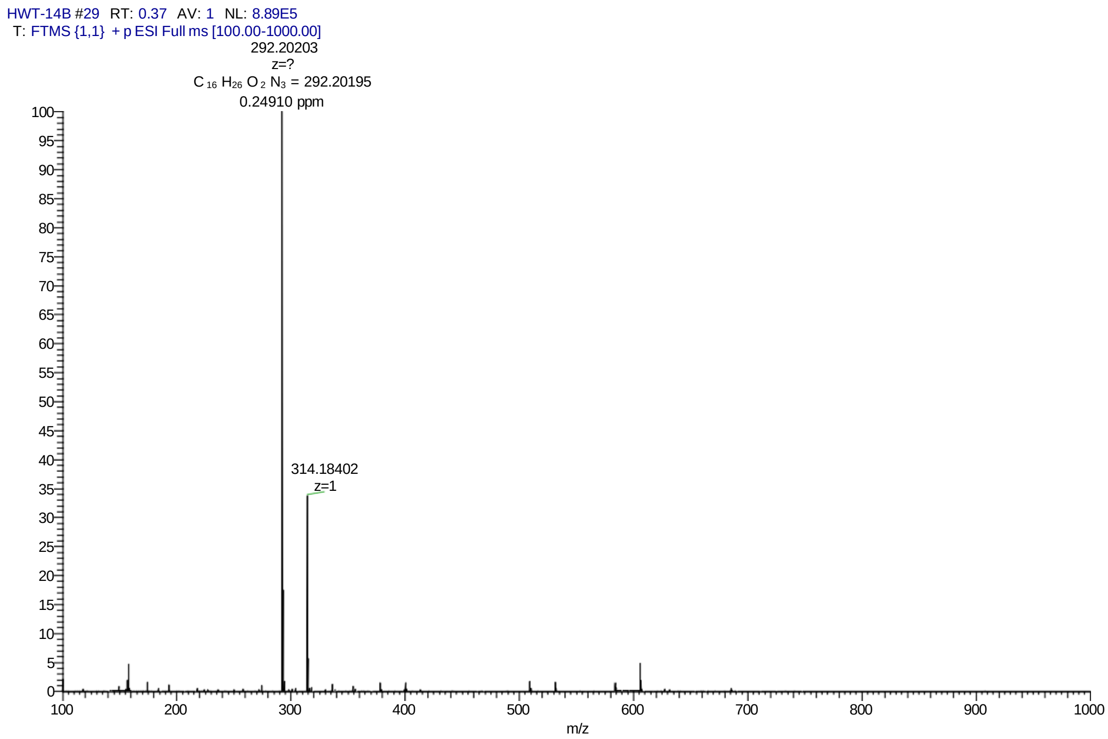

HRMS (AP-ESI) spectrum of compound **14b**

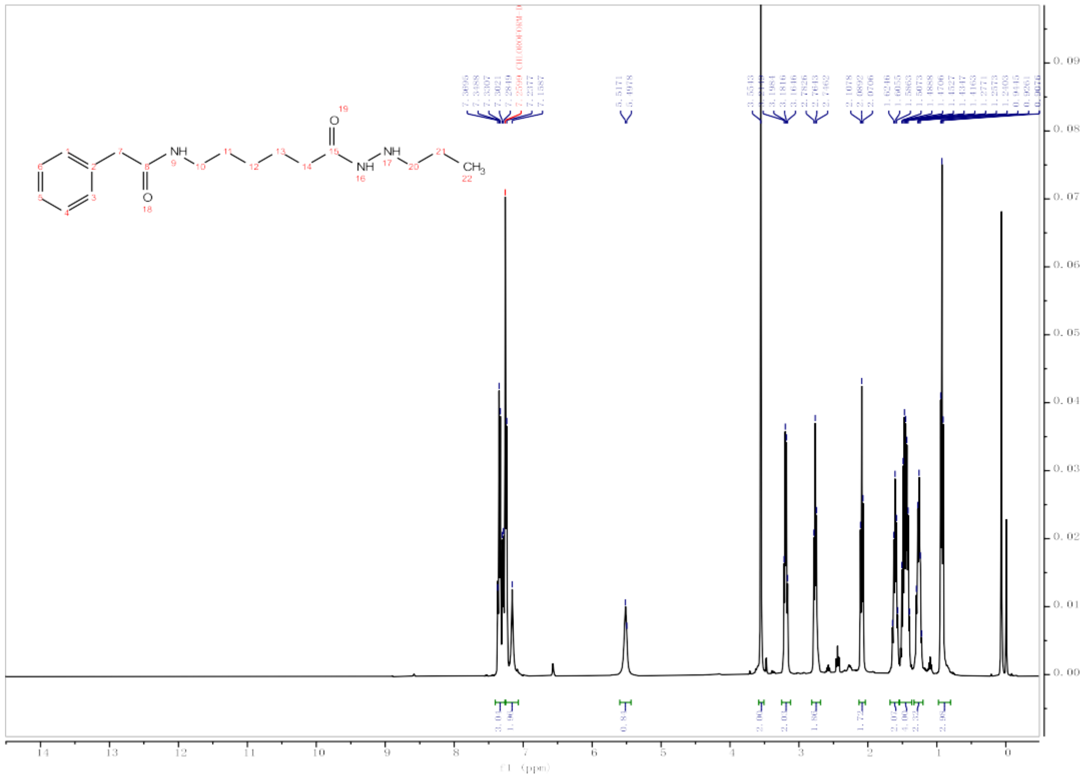

^{1}H NMR spectra for compound **14c**

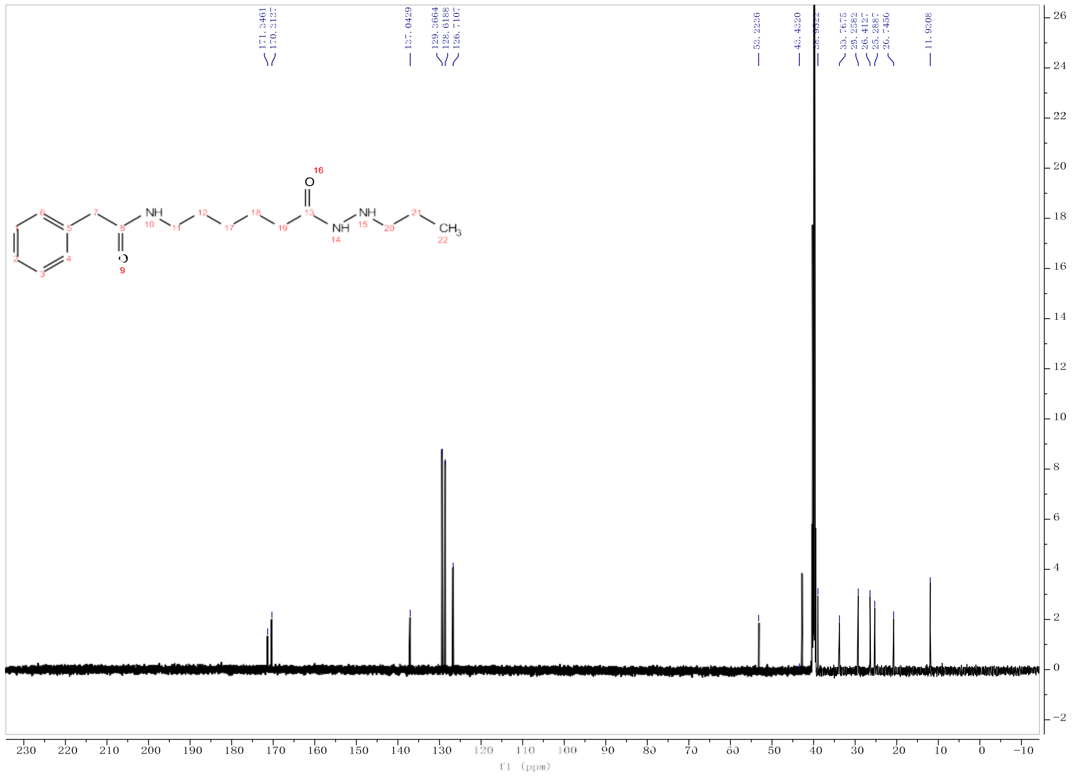

^{13}C NMR spectra for compound **14c**

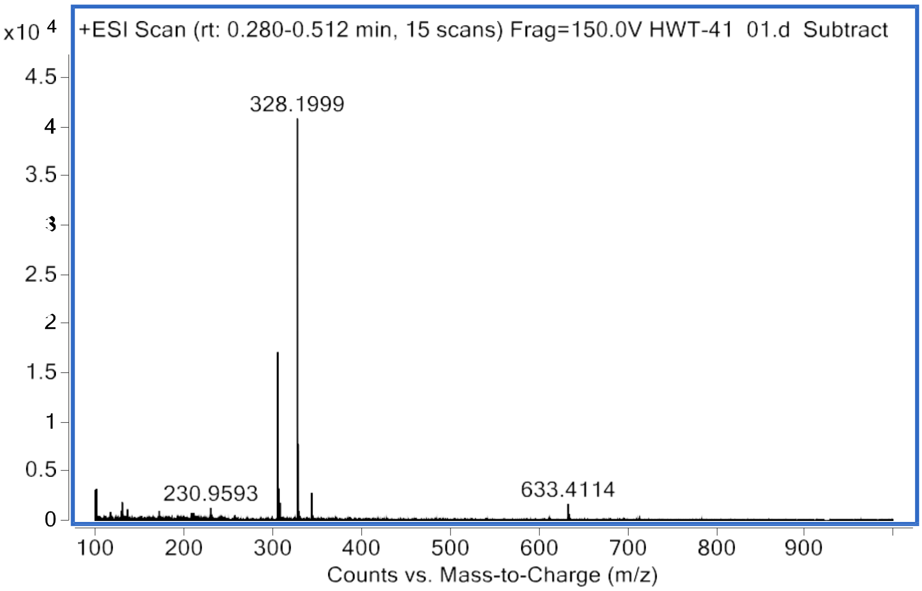

HRMS (AP-ESI) spectrum of compound **14c**

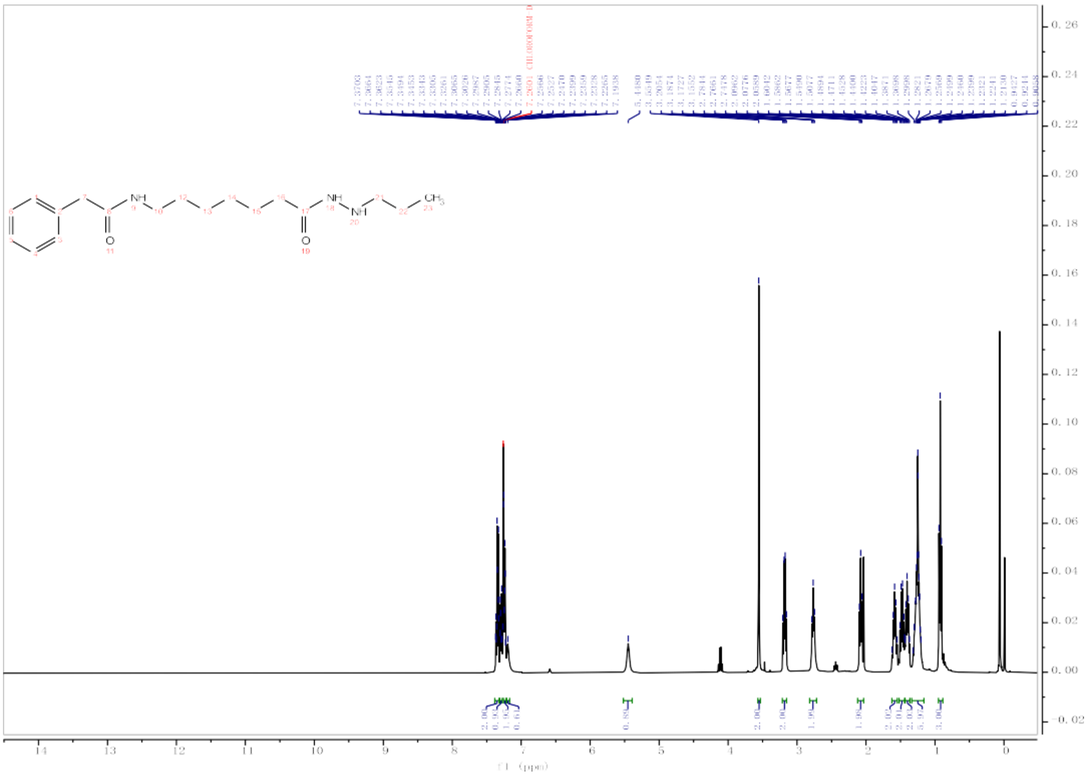

^{1}H NMR spectra for compound **14d**

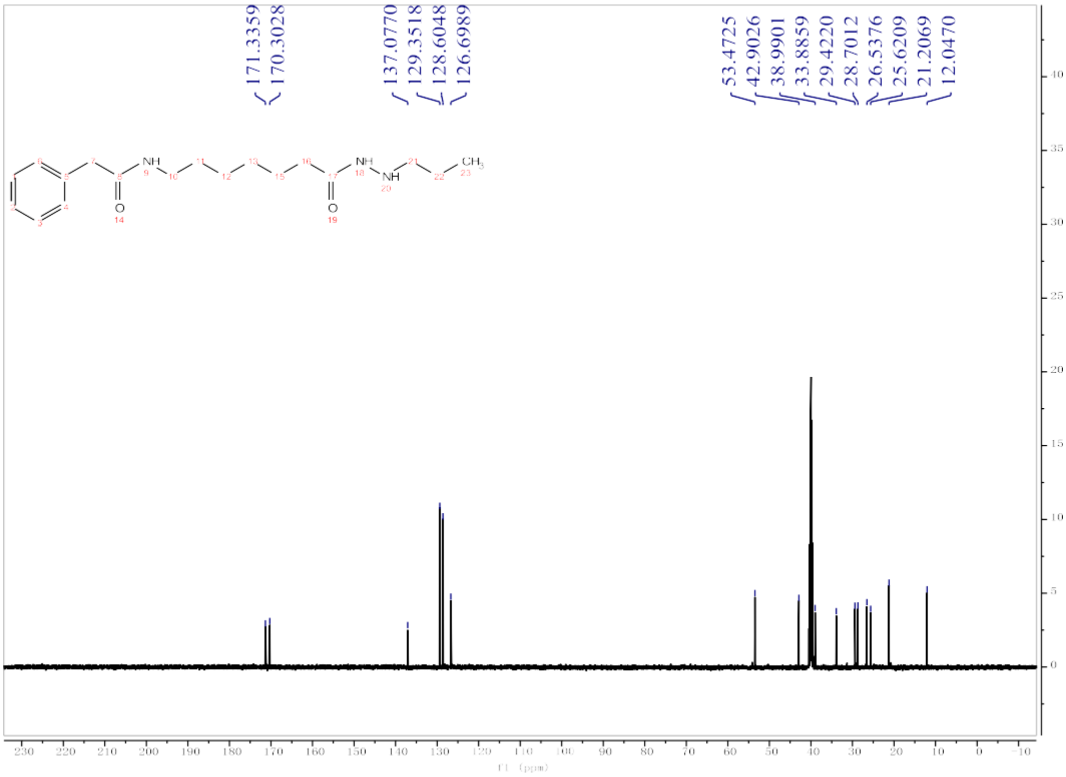

^{13}C NMR spectra for compound **14d**

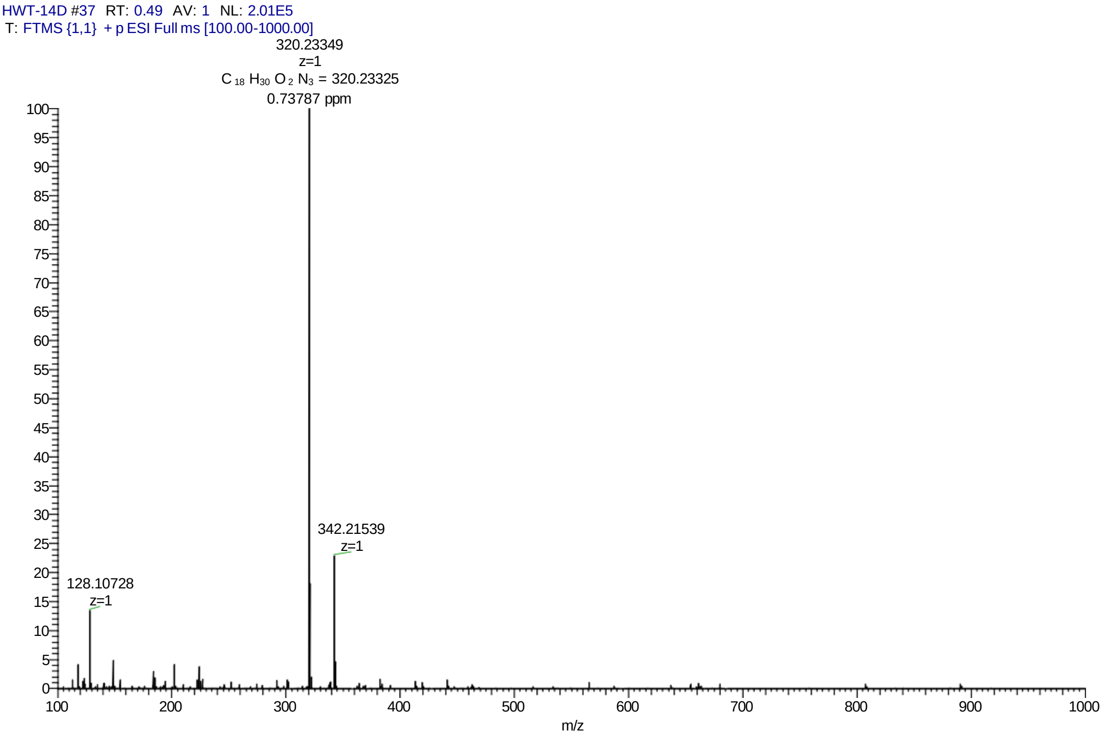

HRMS (AP-ESI) spectrum of compound **14d**

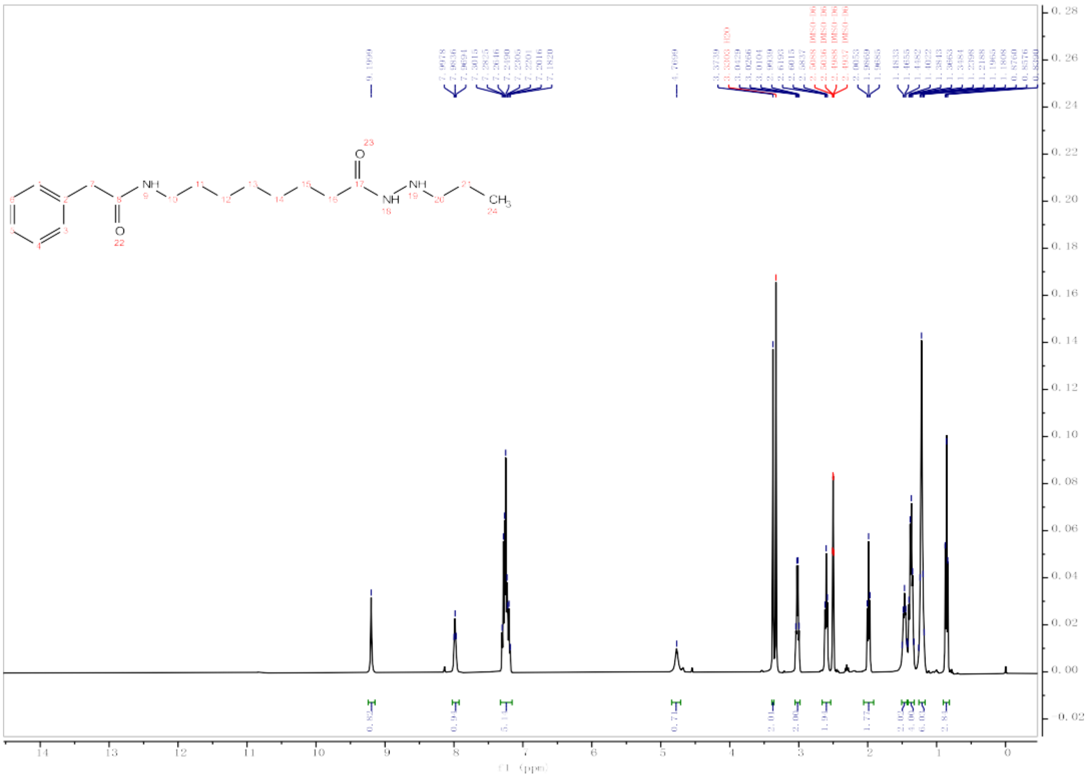

^{1}H NMR spectra for compound **14e**

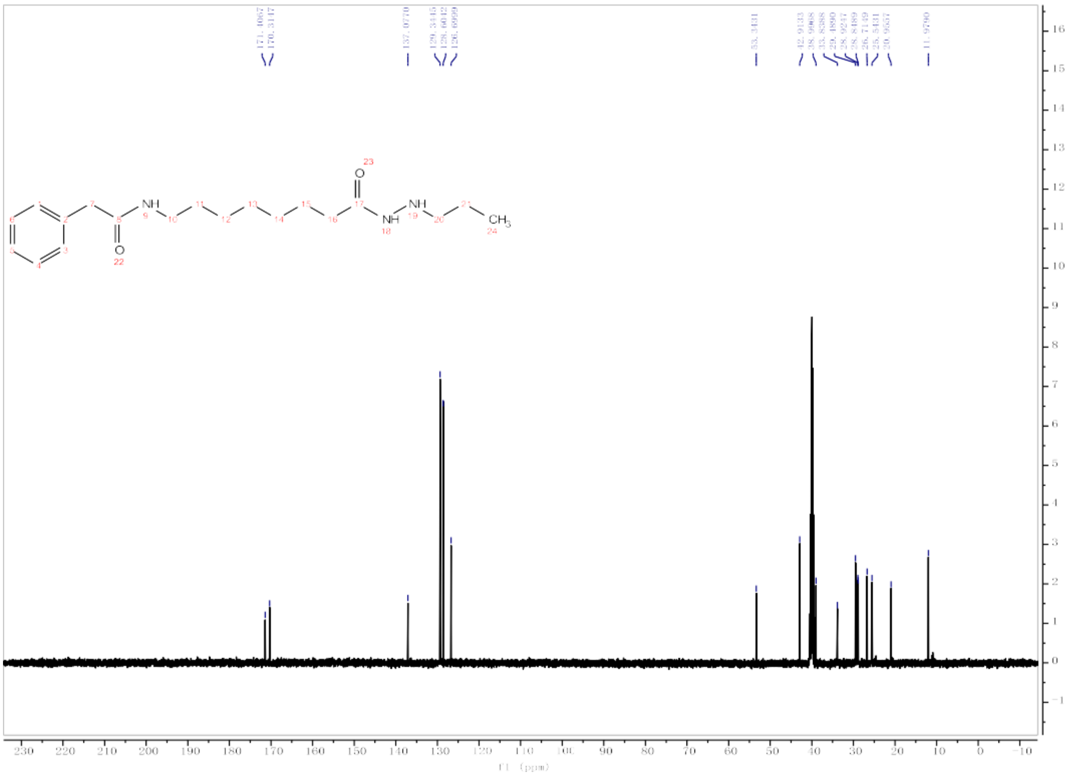

^{13}C NMR spectra for compound **14e**

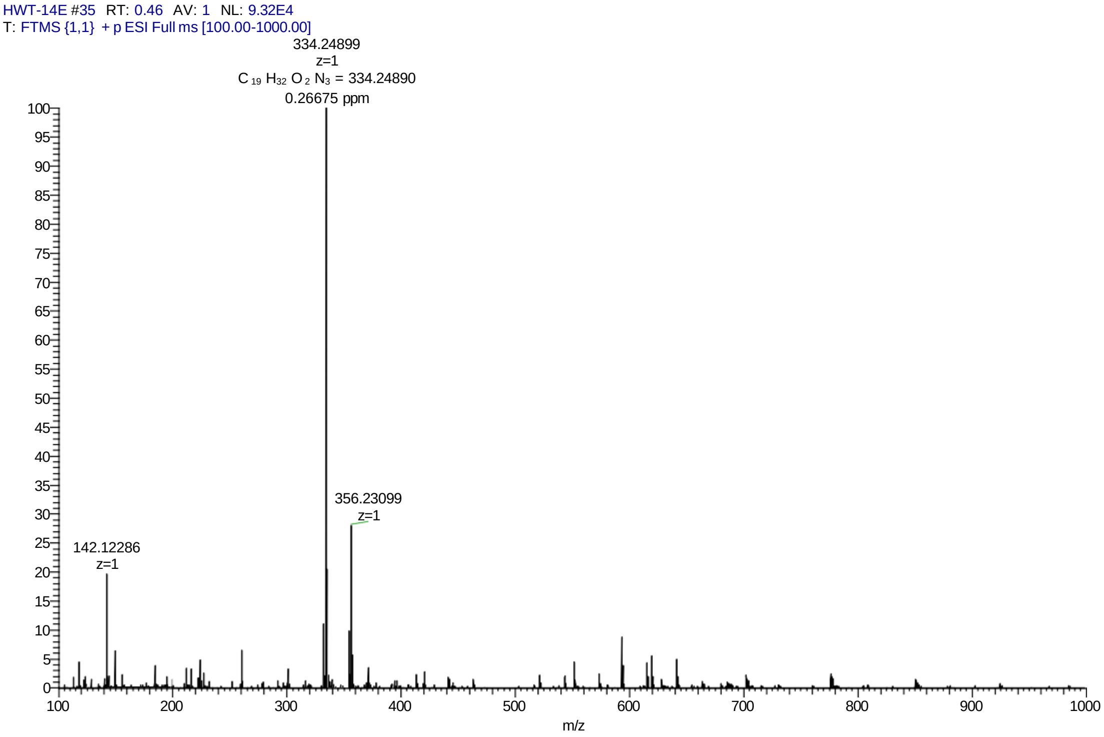

HRMS (AP-ESI) spectrum of compound **14e**

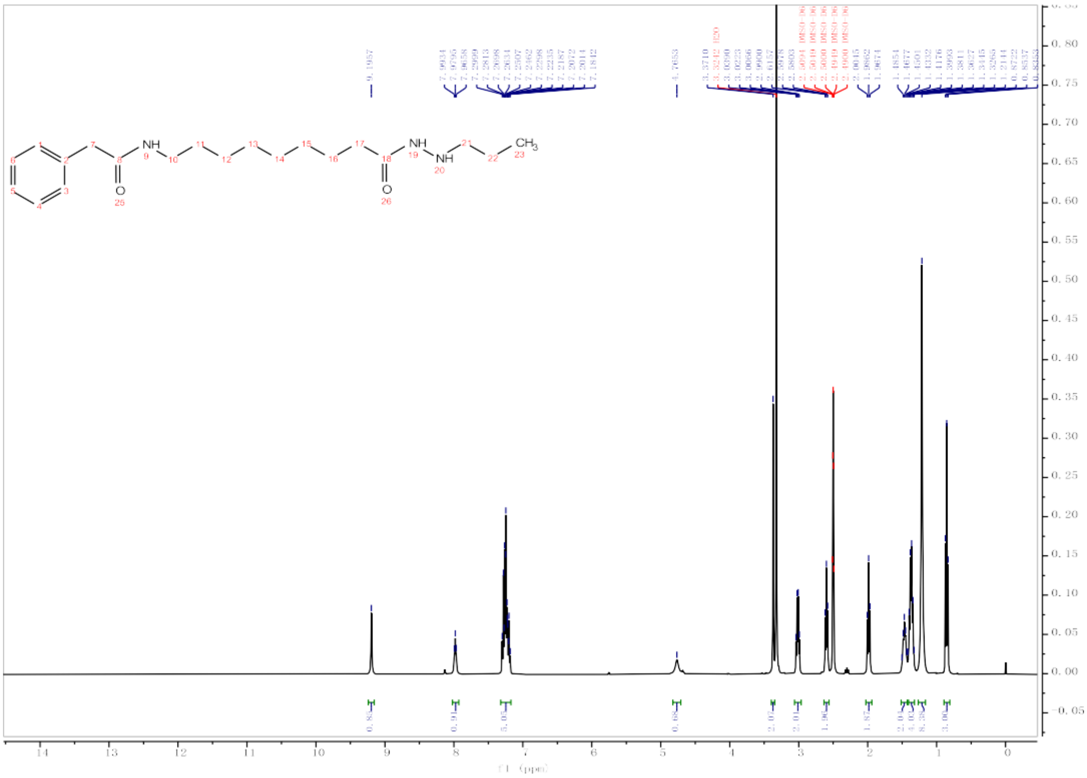

^{1}H NMR spectra for compound **14f**

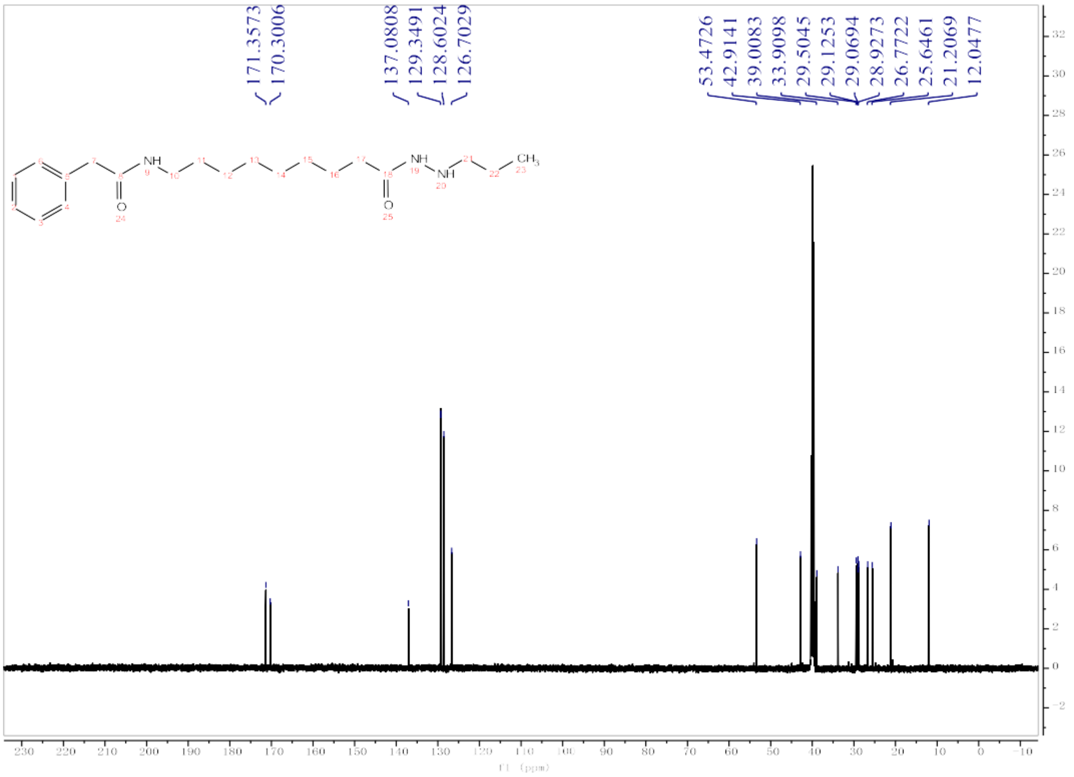

^{13}C NMR spectra for compound **14f**

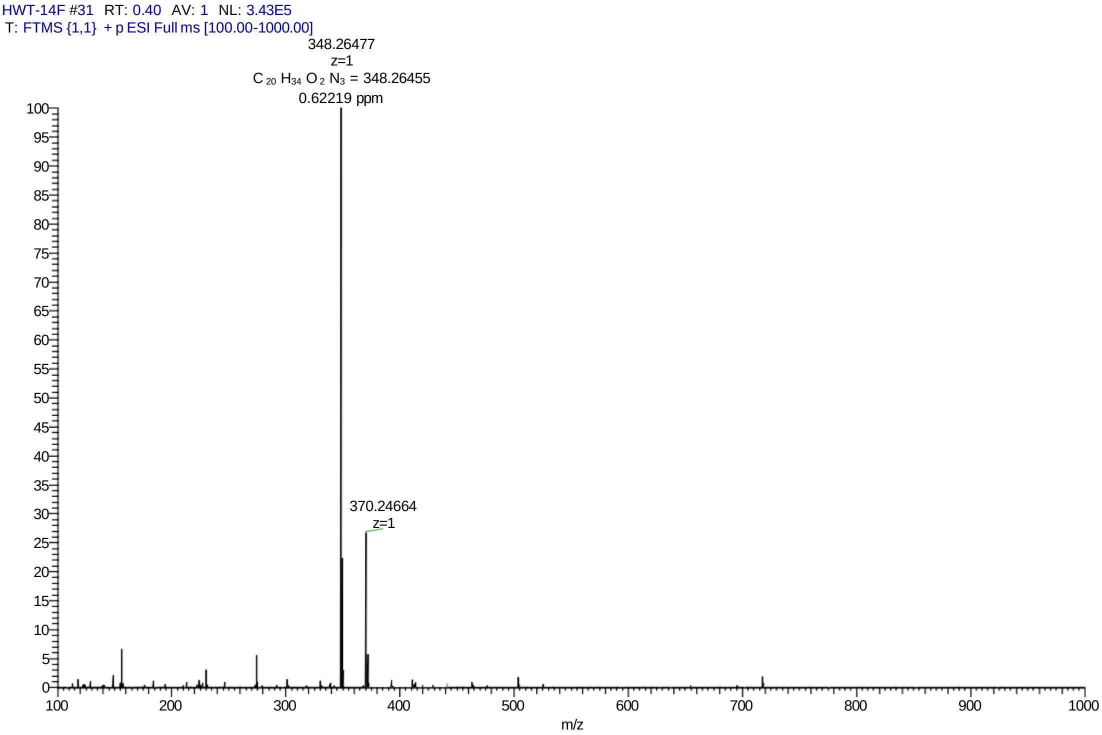

HRMS (AP-ESI) spectrum of compound **14f**

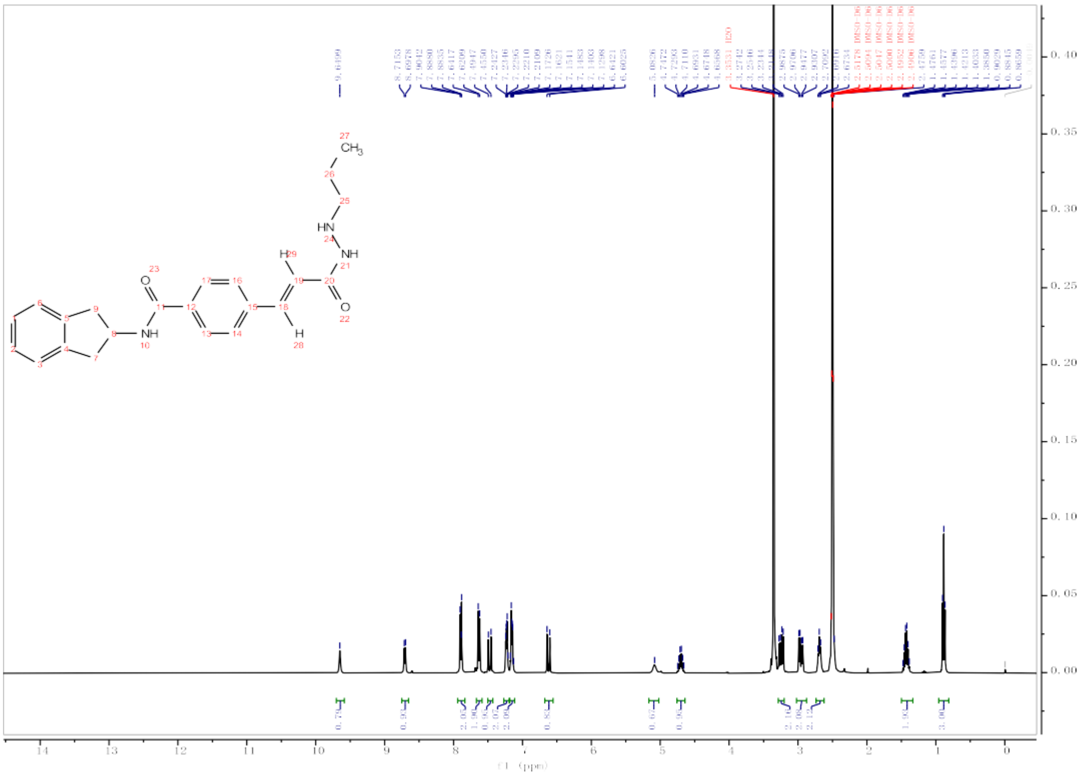

^{1}H NMR spectra for compound **19a**

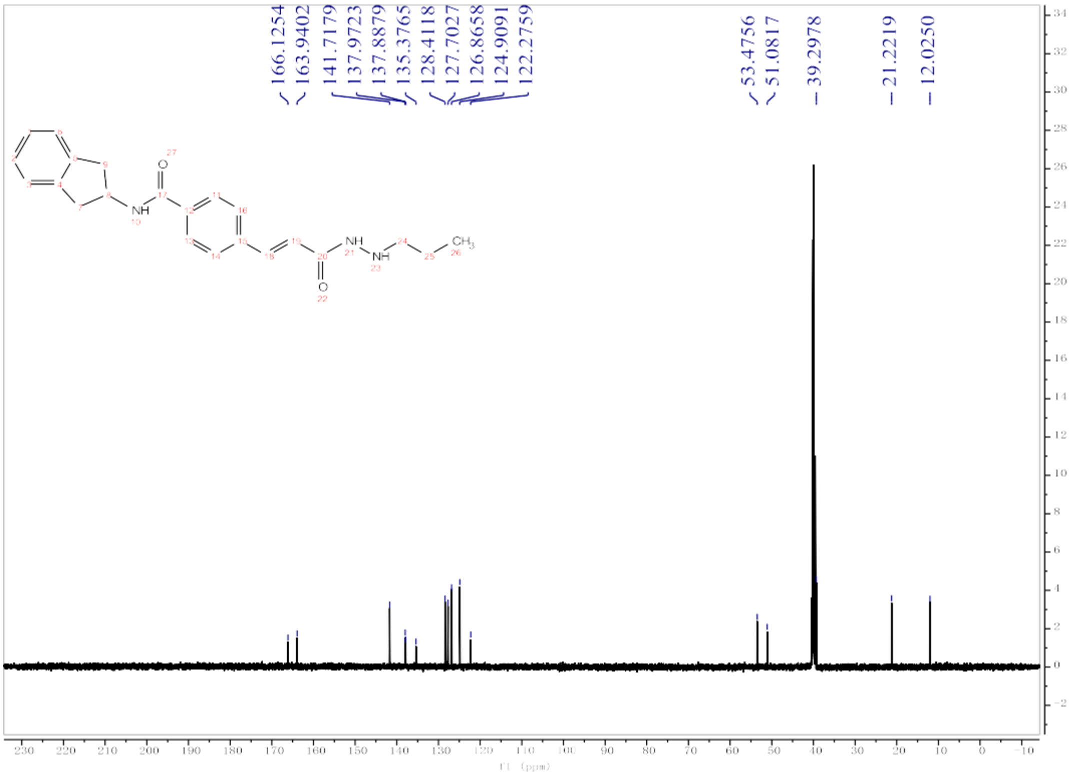

^{13}C NMR spectra for compound **19a**

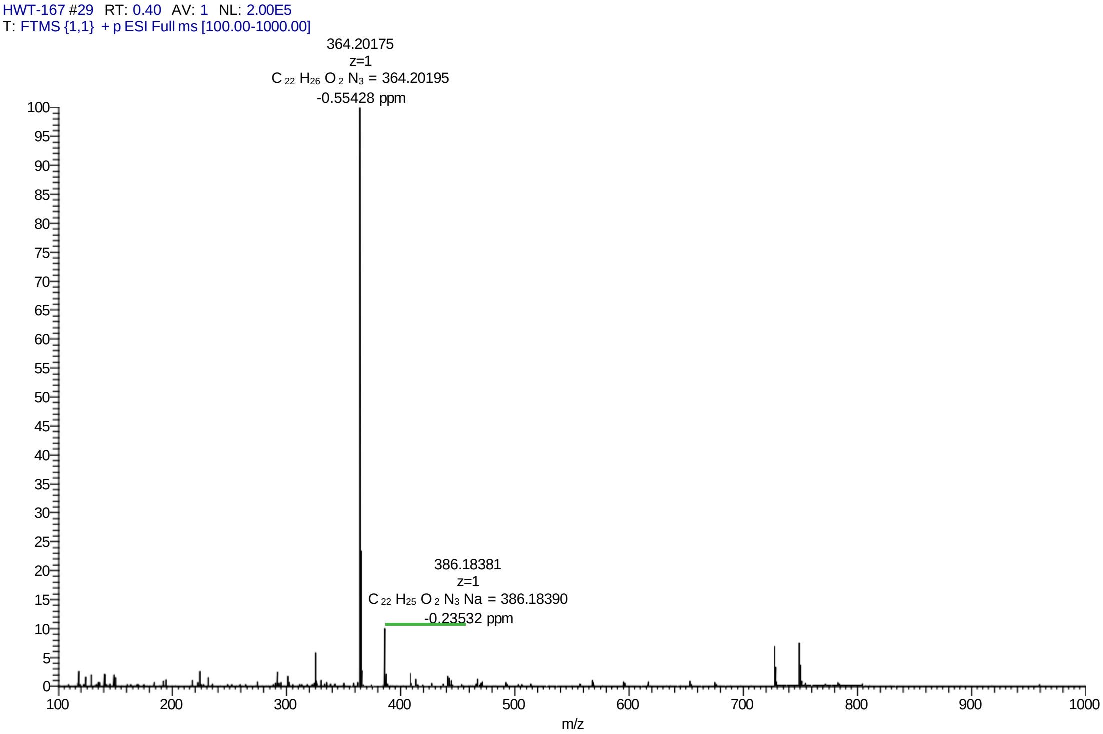

HRMS (AP-ESI) spectra for compound **19a**

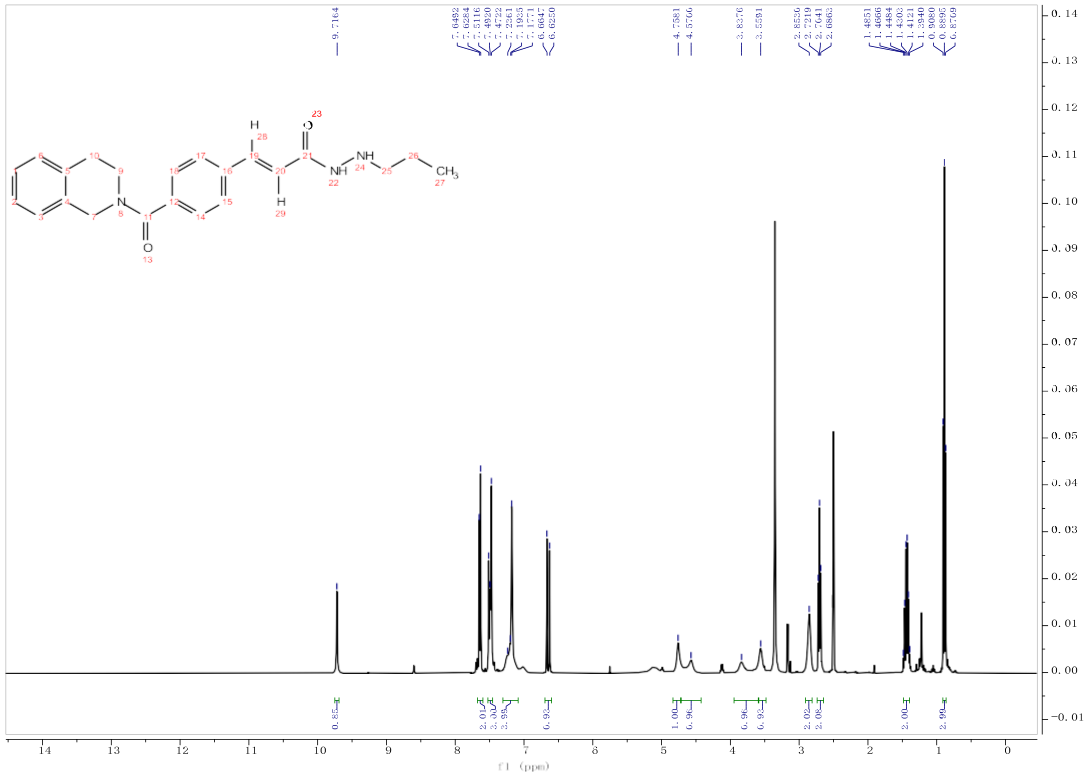

^{1}H NMR spectra for compound **19b**

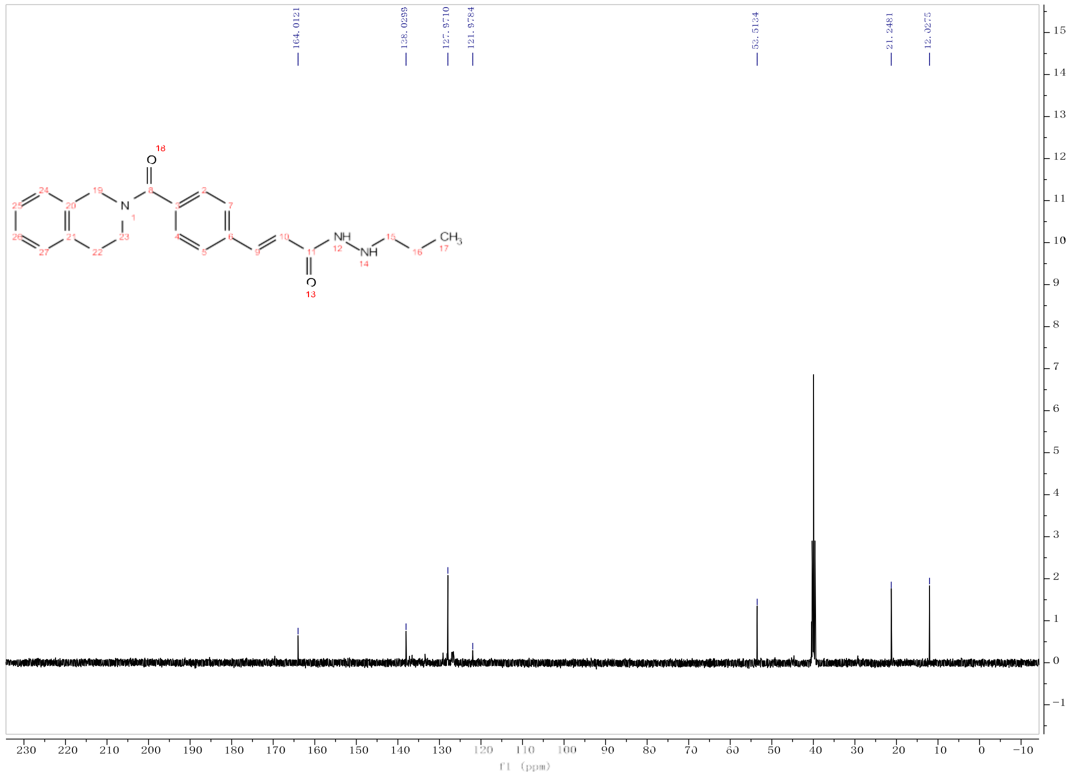

^{13}C NMR spectra for compound **19b**

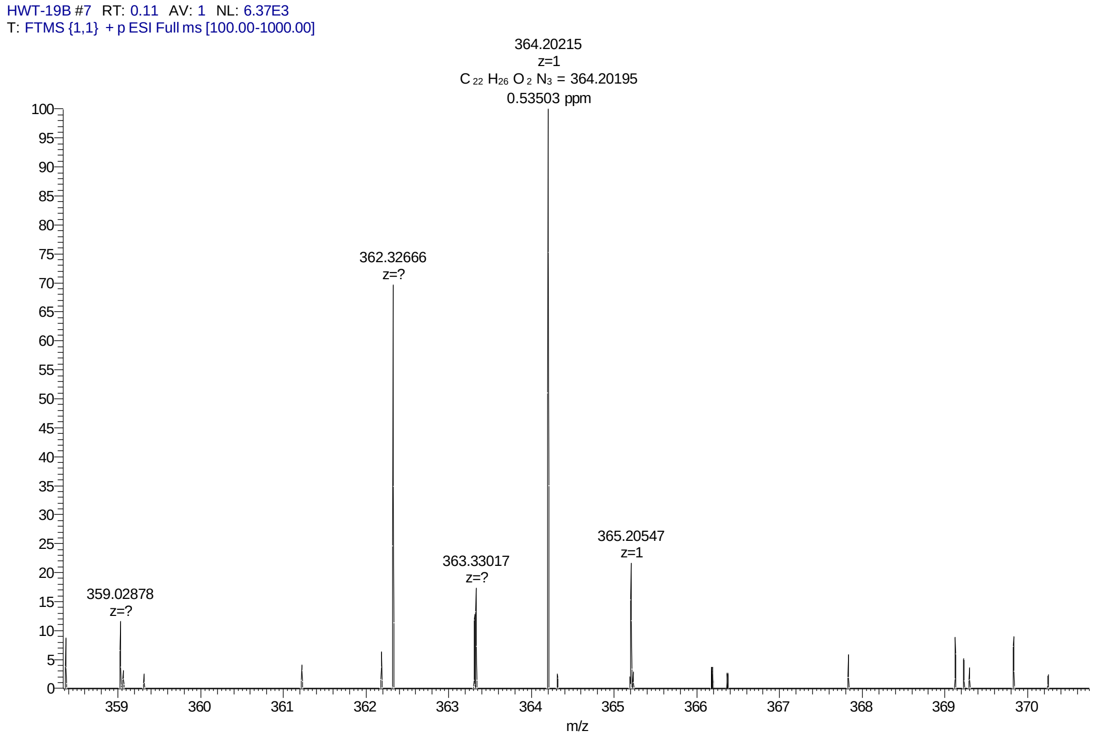

HRMS (AP-ESI) spectrum of compound **19b**

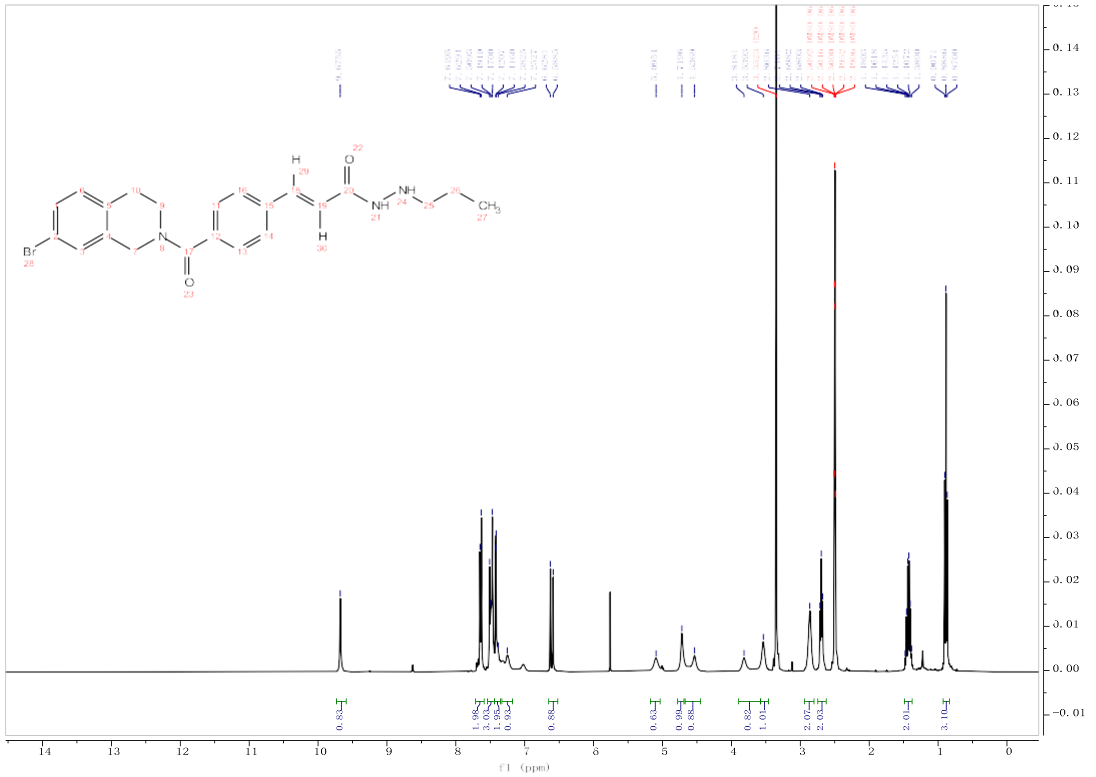

^{1}H NMR spectra for compound **19c**

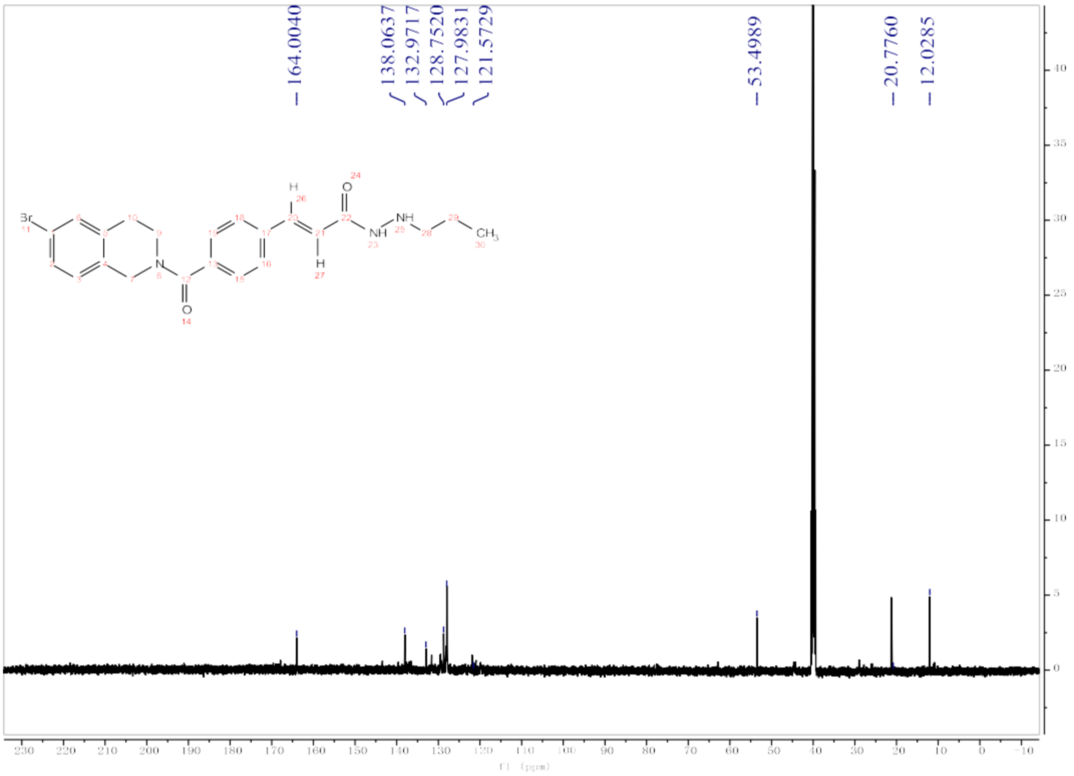

^{13}C NMR spectra for compound **19c**

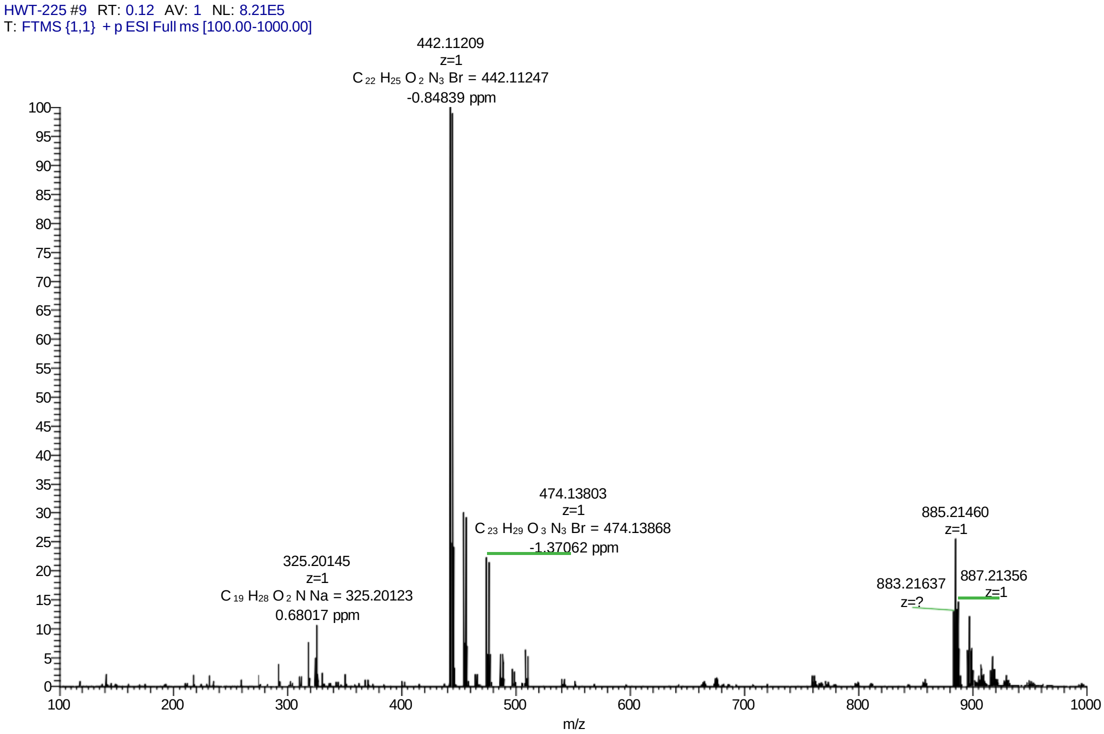

HRMS (AP-ESI) spectra for compound **19c**

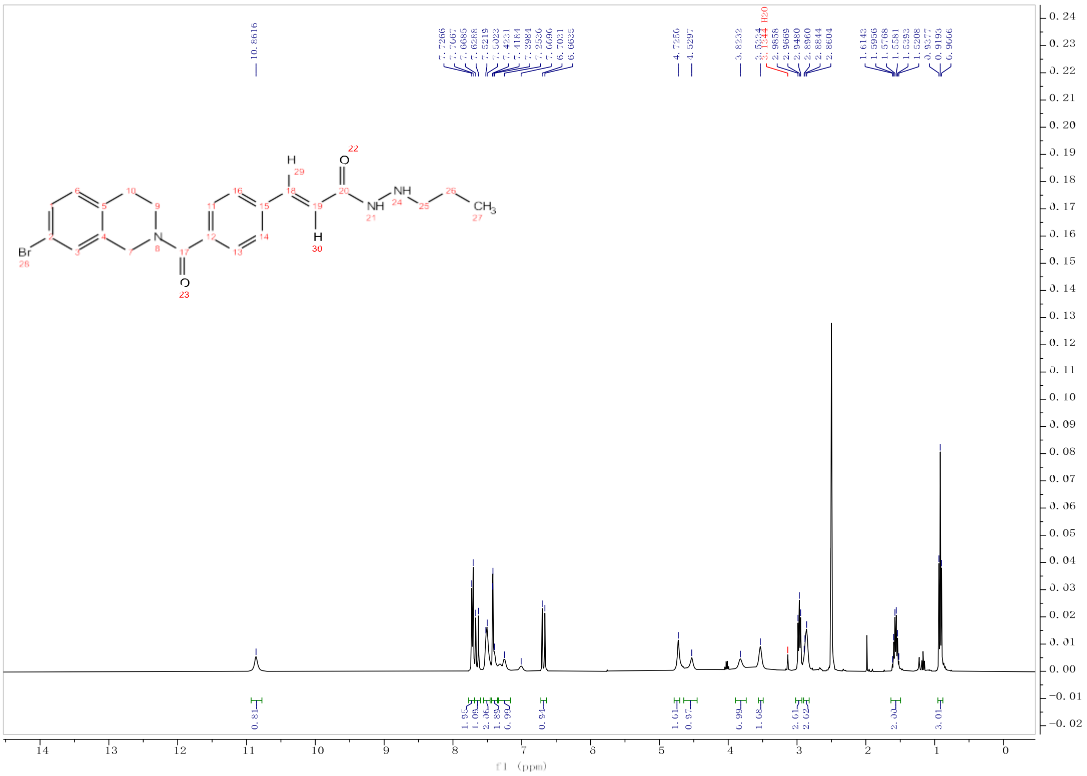

^{1}H NMR spectra for compound **19d**

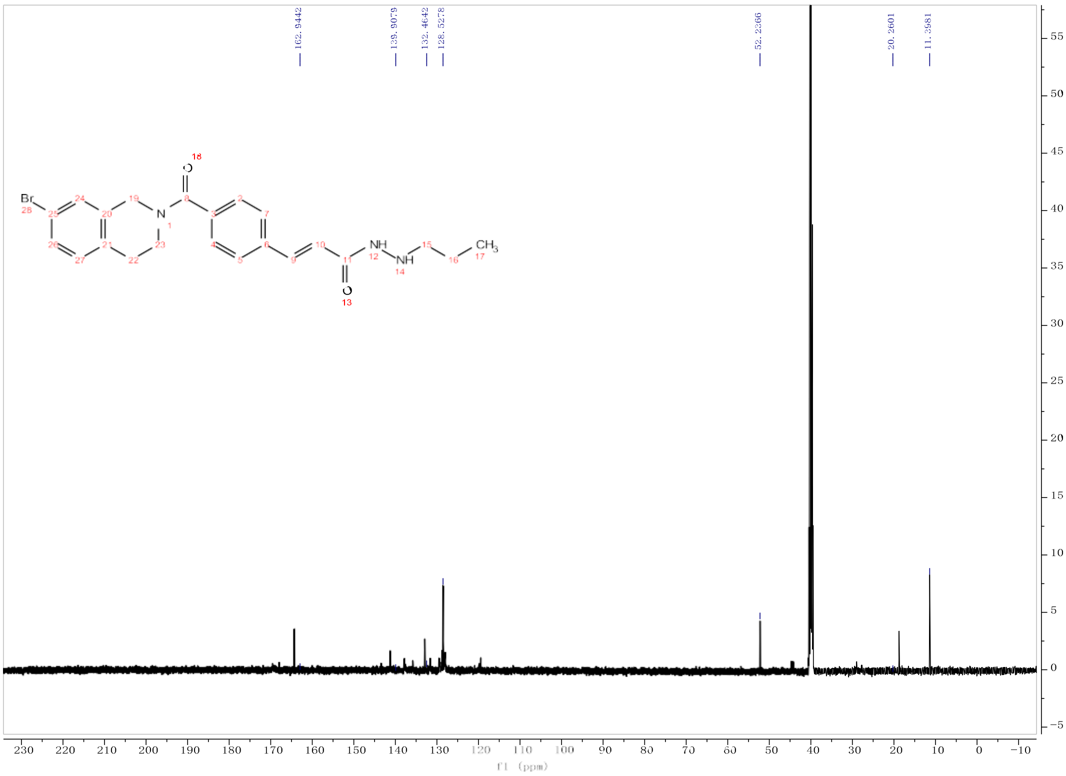

^{13}C NMR spectra for compound **19d**

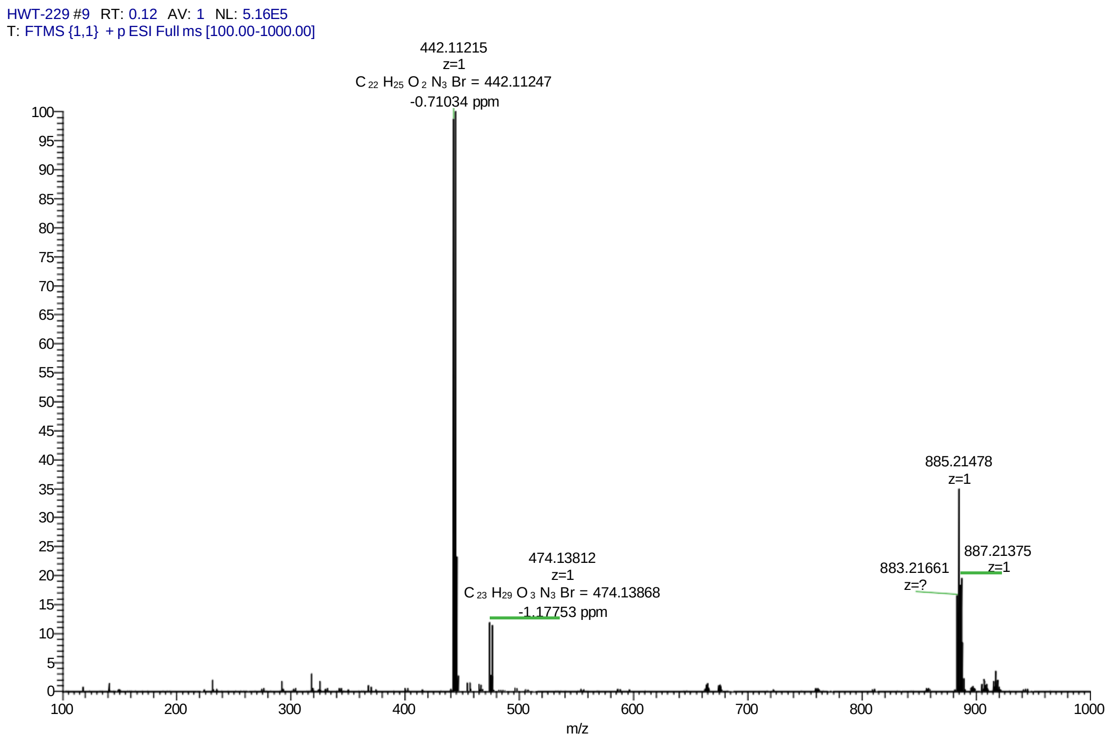

HRMS (AP-ESI) spectra for compound **19d**

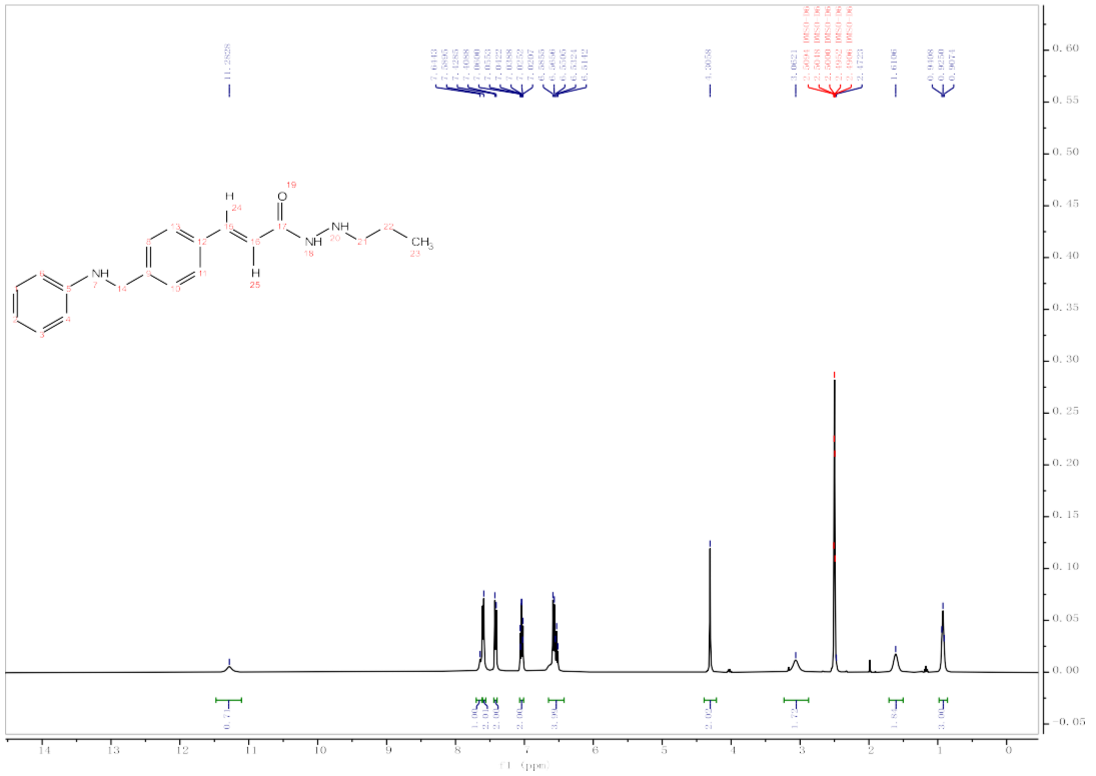

^{1}H NMR spectra for compound **25a**

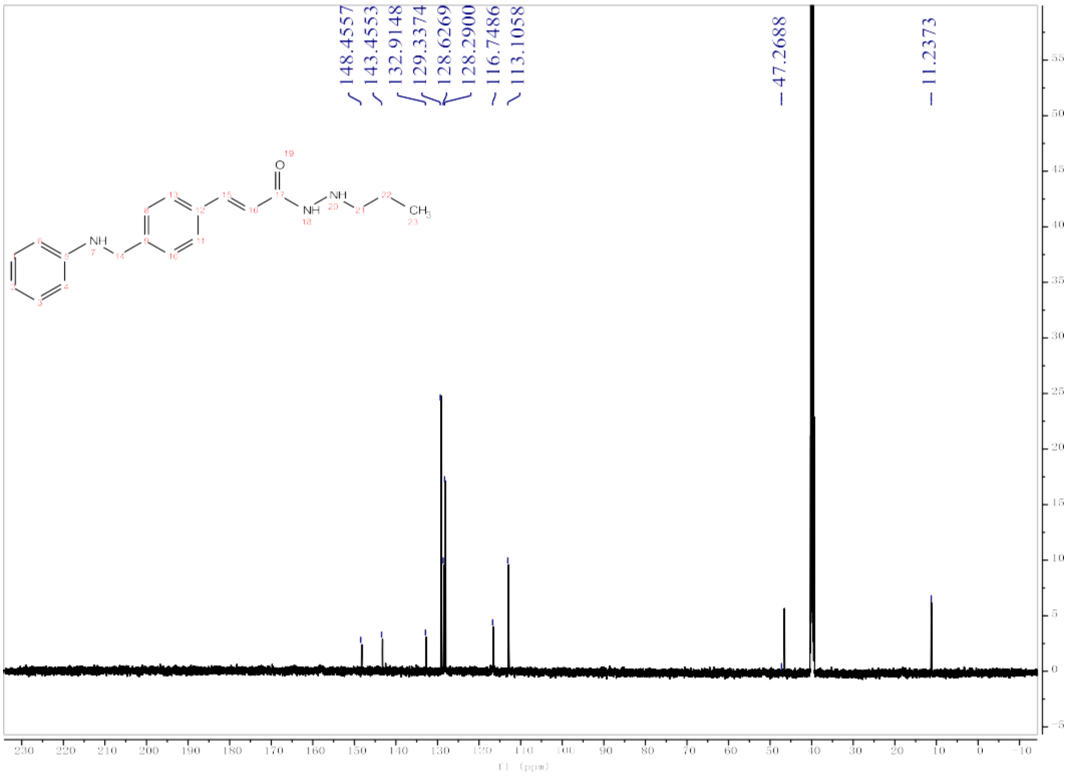

^{13}C NMR spectra for compound **25a**

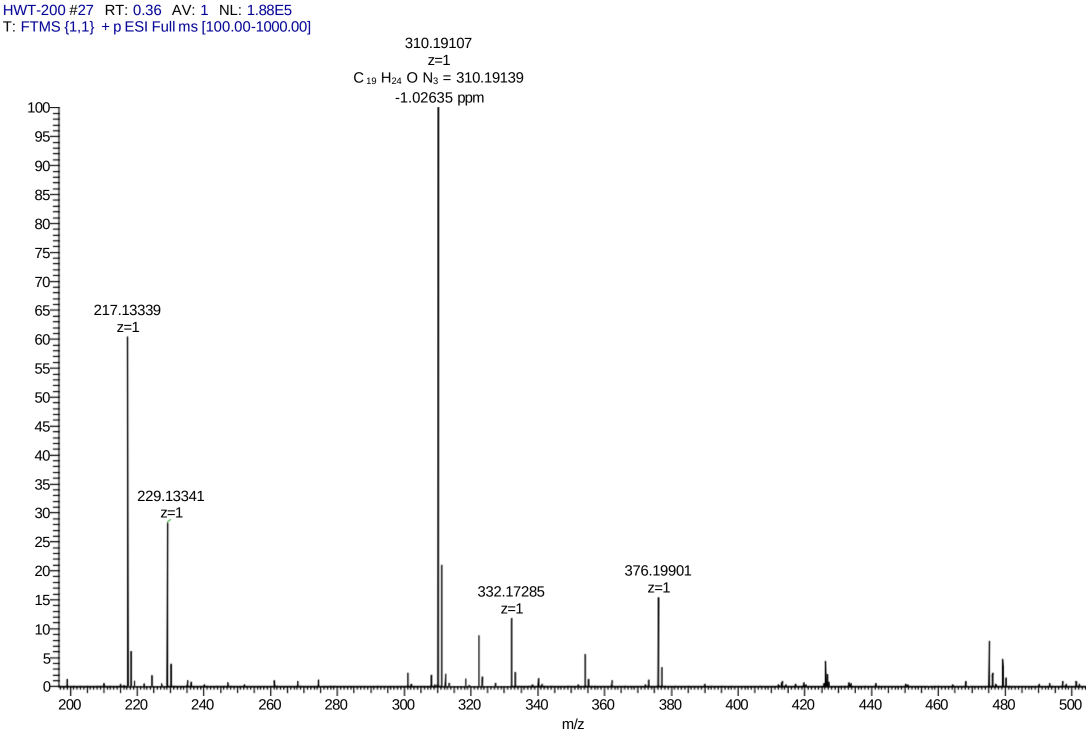

HRMS (AP-ESI) spectra for compound **25a**

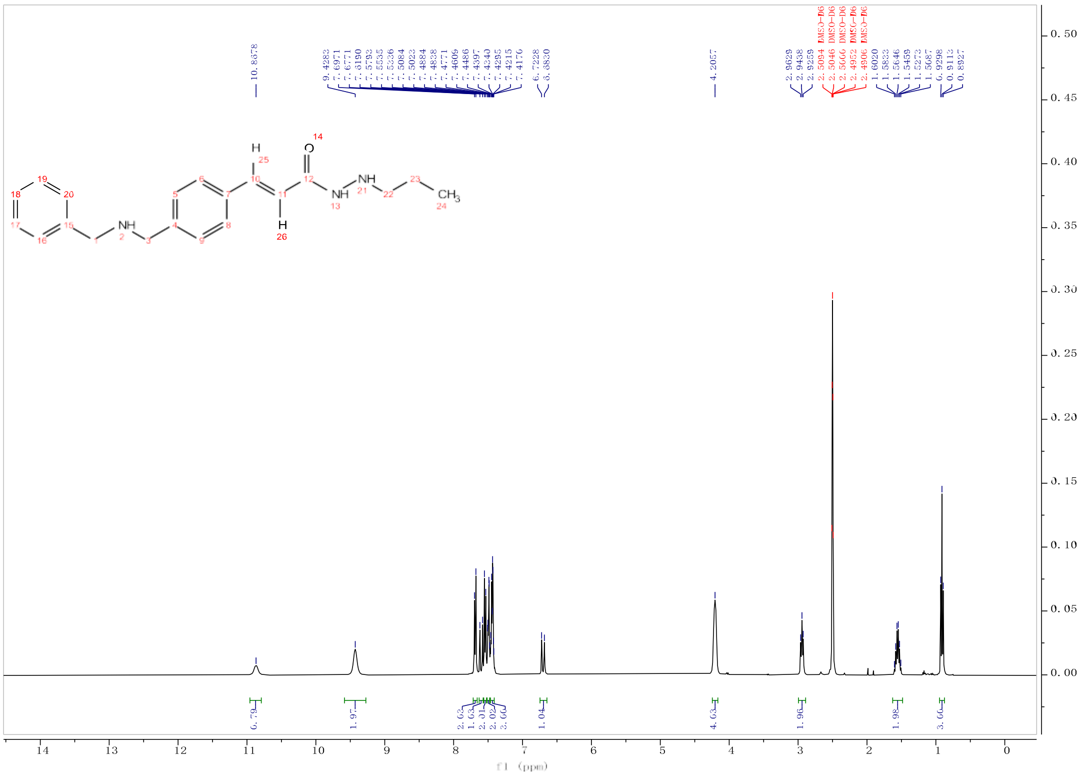

^{1}H NMR spectra for compound **25b**

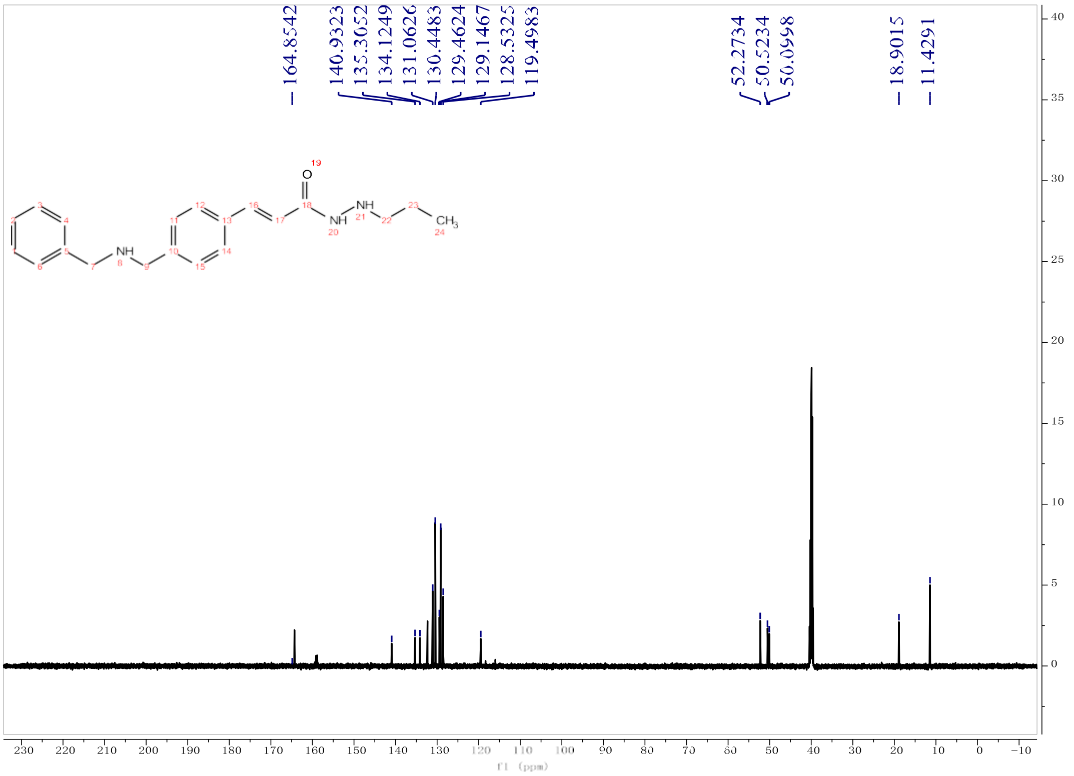

^{13}C NMR spectra for compound **25b**

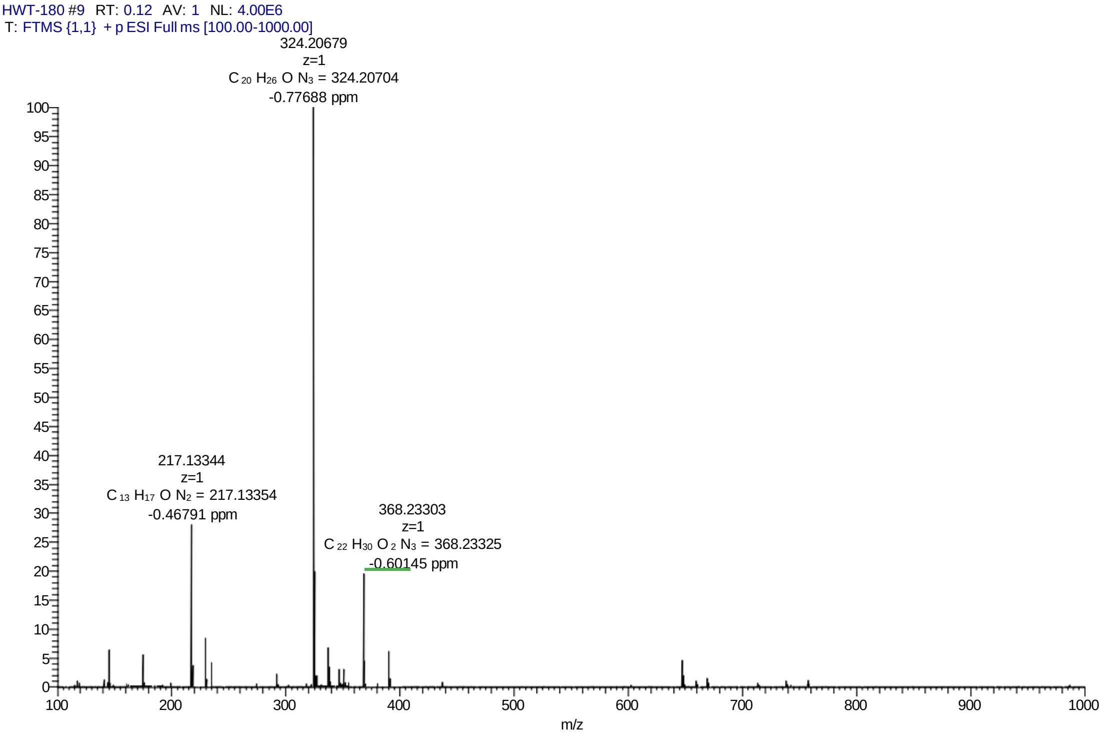

HRMS (AP-ESI) spectra for compound **25b**

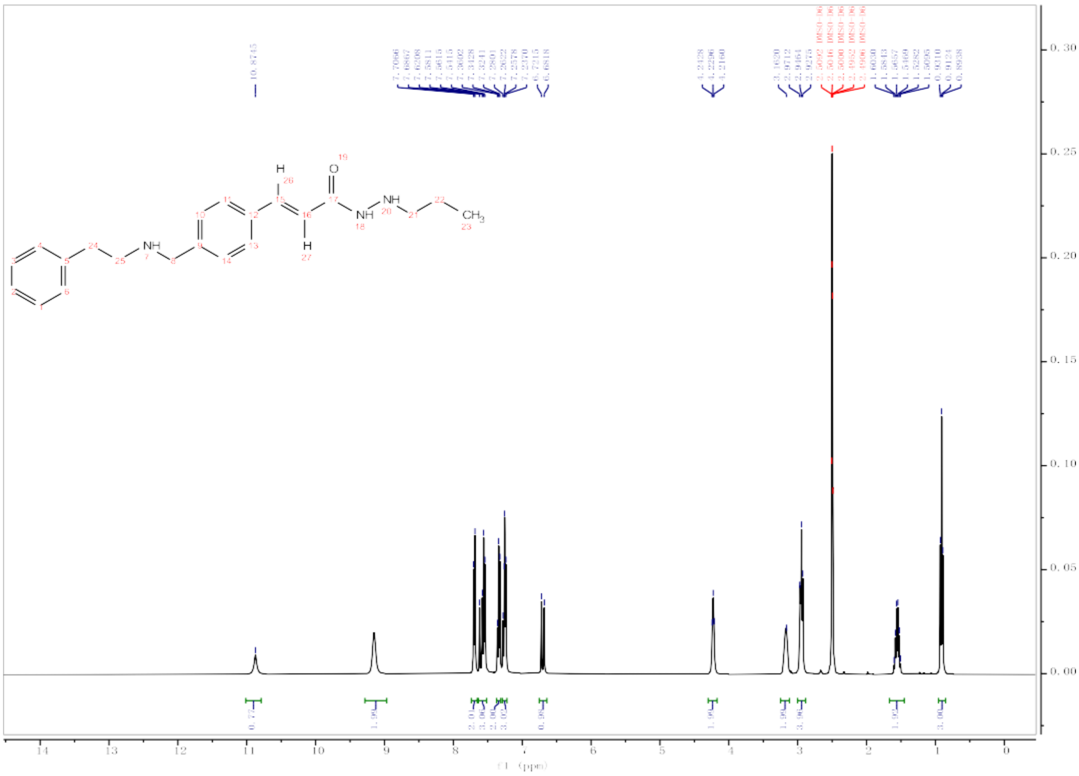

^{1}H NMR spectra for compound **25c**

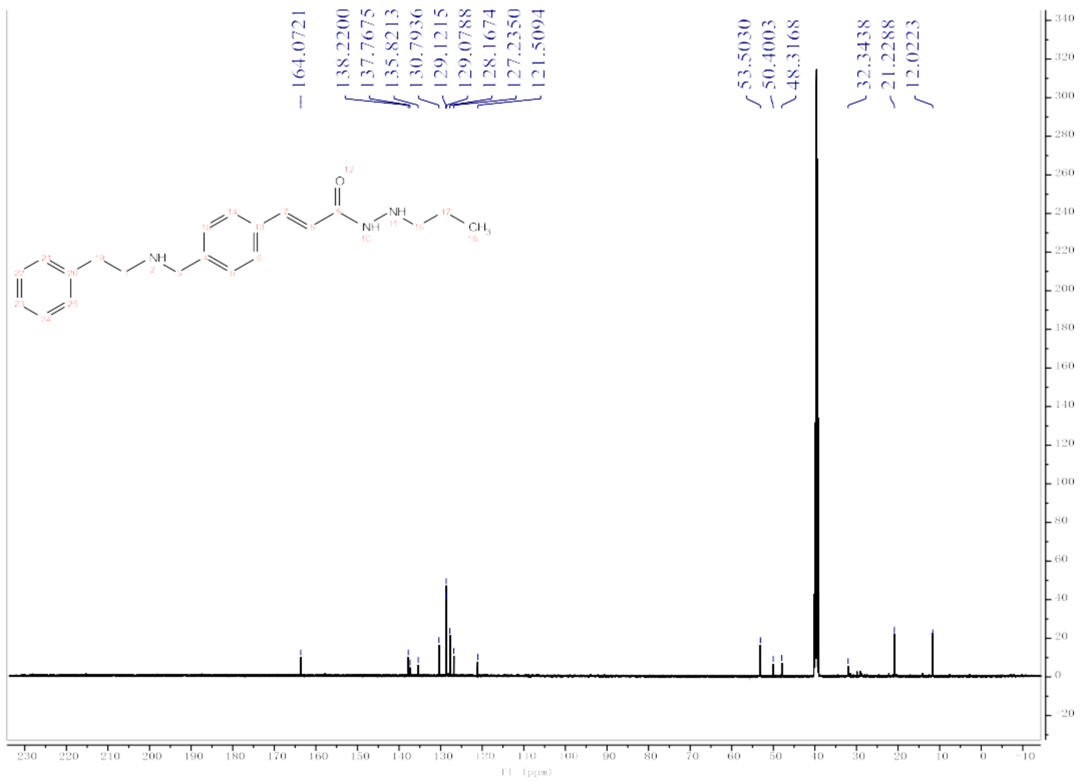

^{13}C NMR spectra for compound **25c**

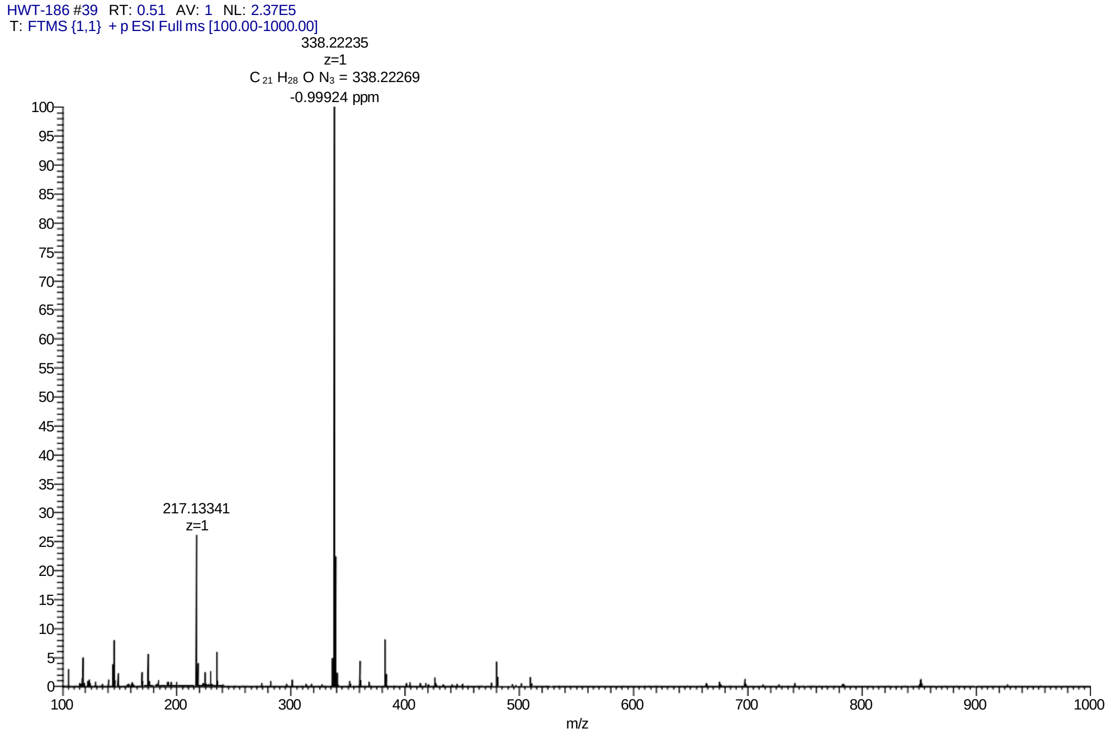

HRMS (AP-ESI) spectra for compound **25c**

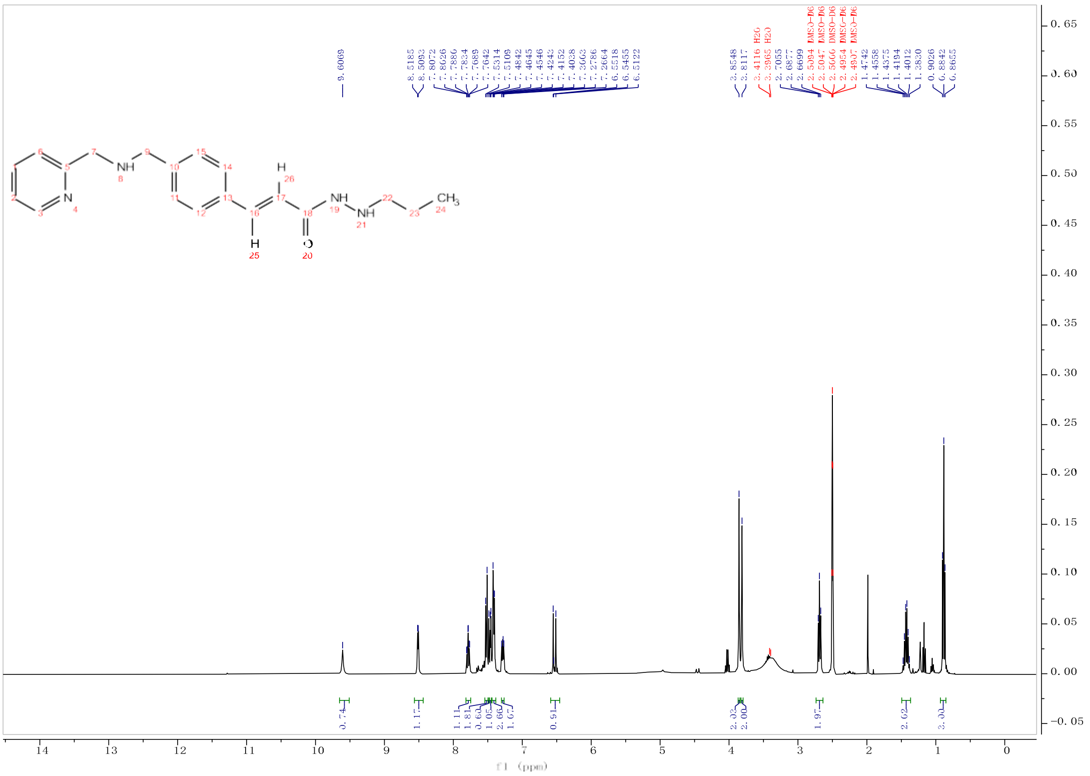

^{1}H NMR spectra for compound **25d**

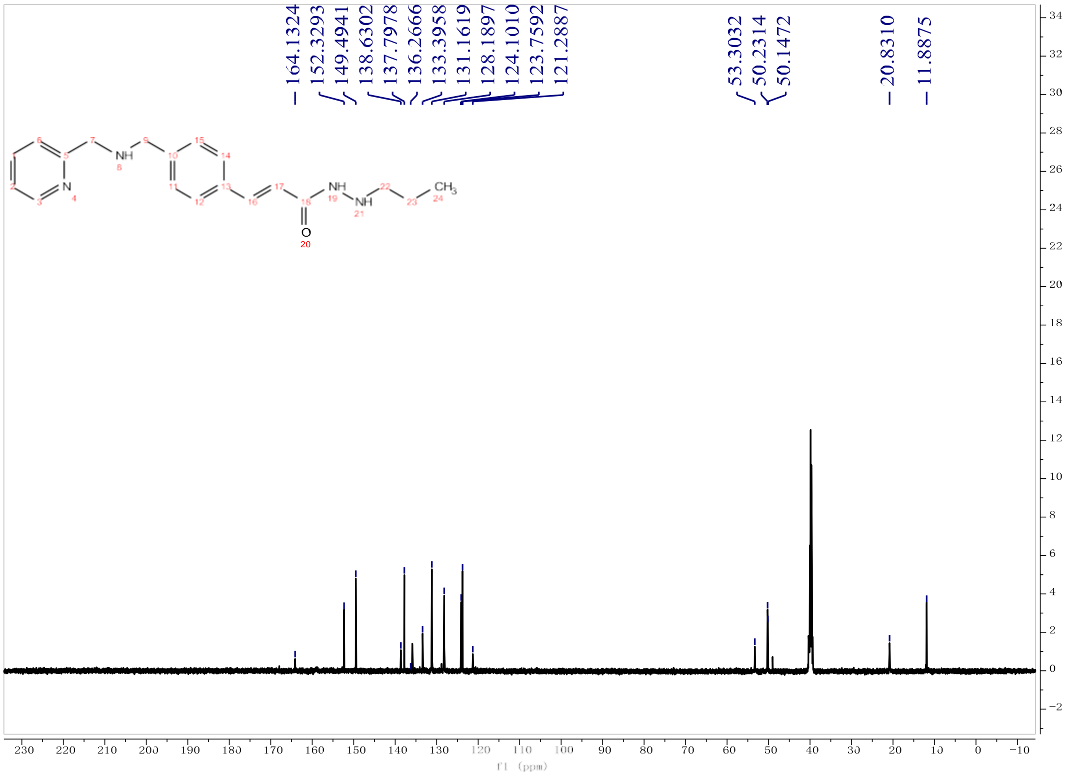

^{13}C NMR spectra for compound **25d**

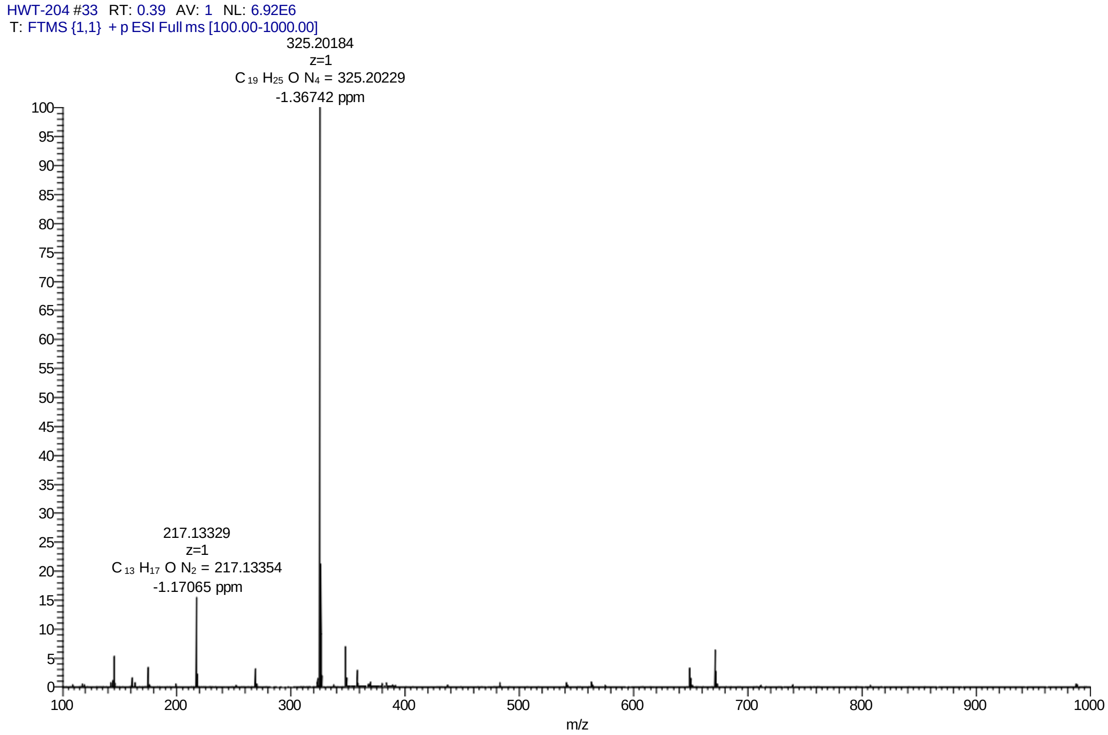

HRMS (AP-ESI) spectra for compound **25d**

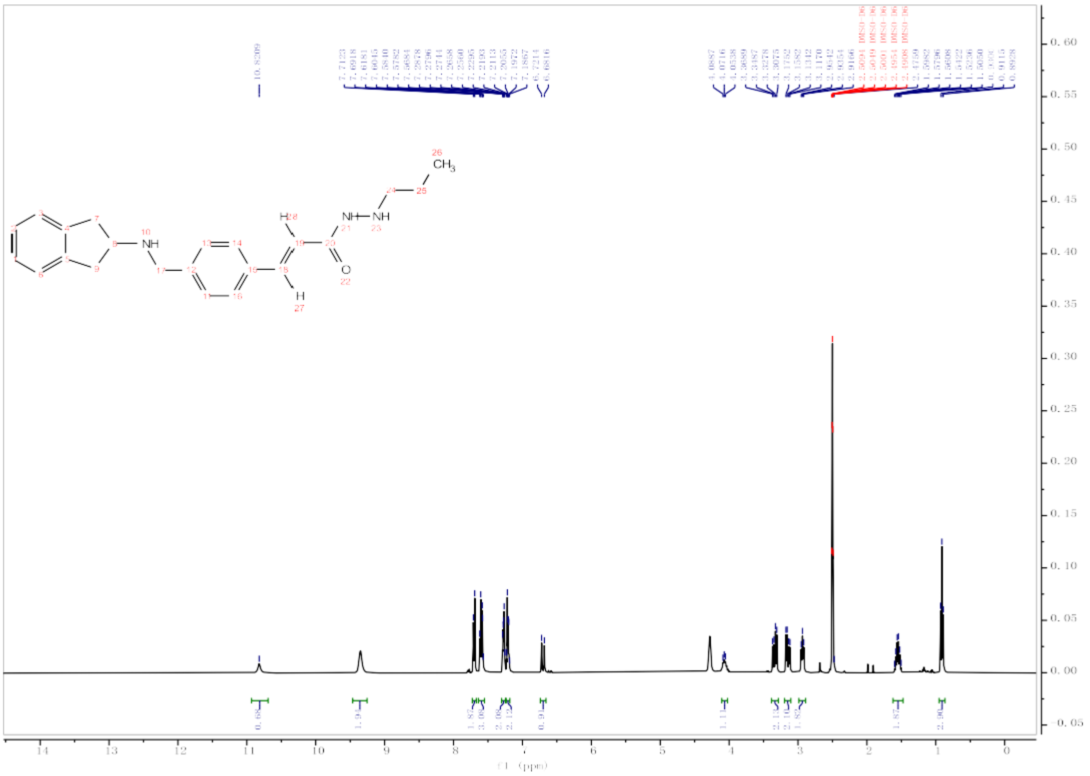

^{1}H NMR spectra for compound **25e**

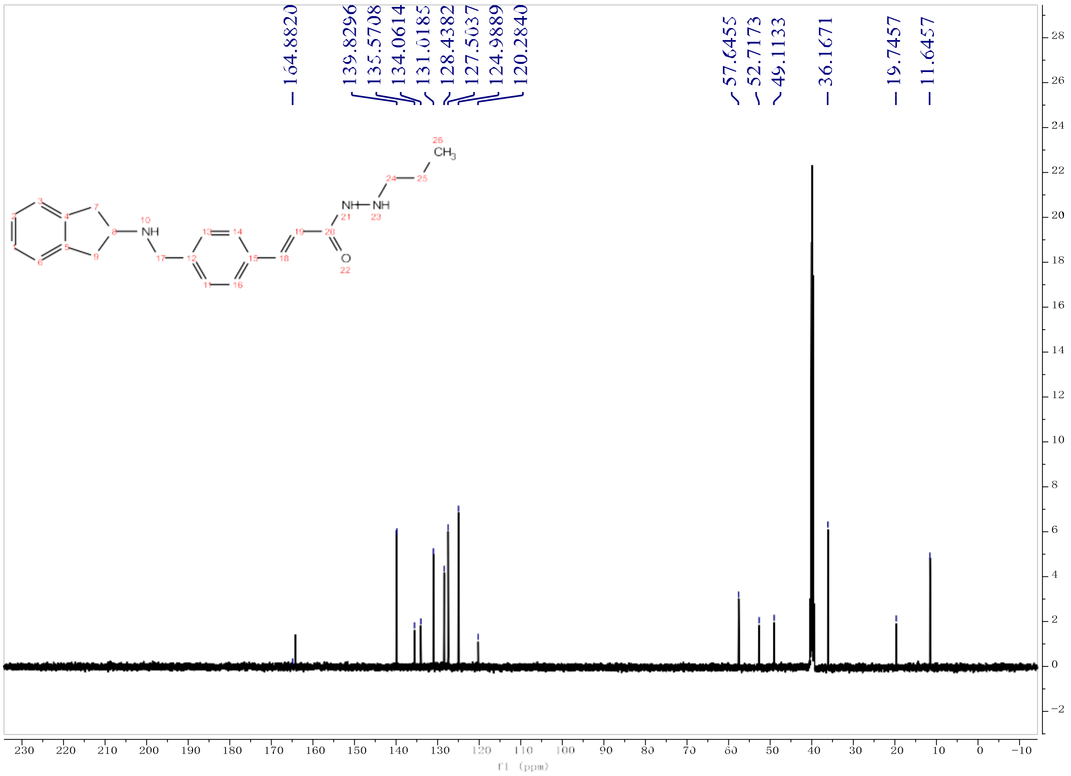

^{13}C NMR spectra for compound **25e**

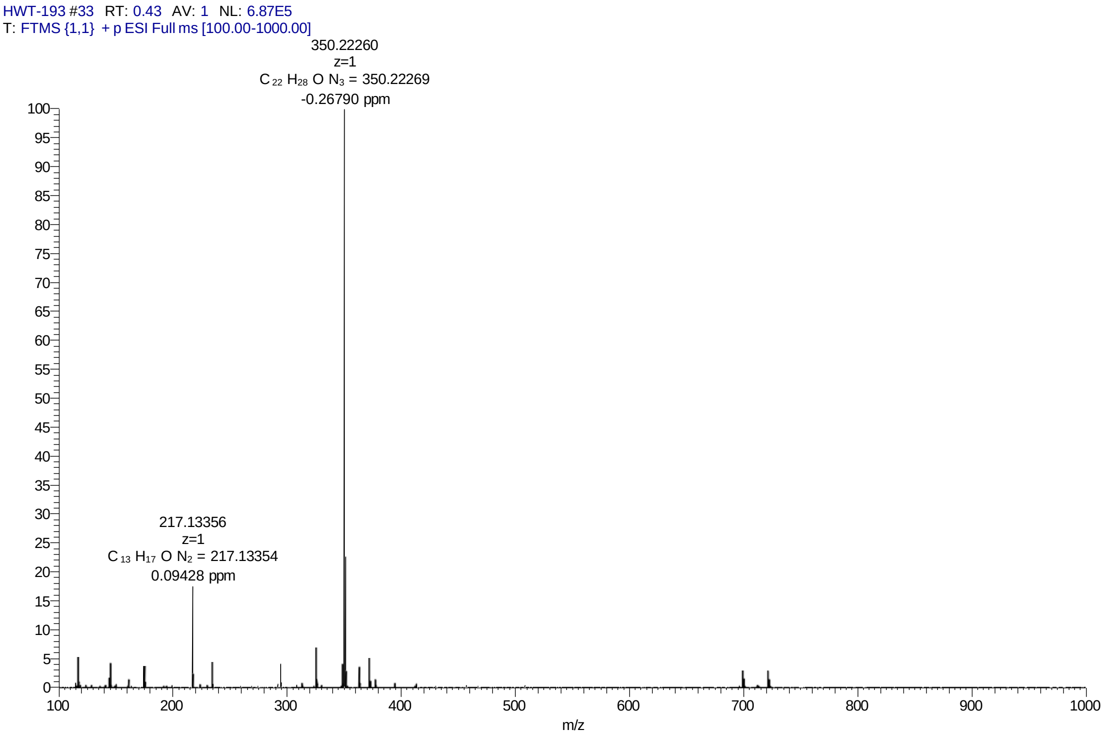

HRMS (AP-ESI) spectra for compound **25e**

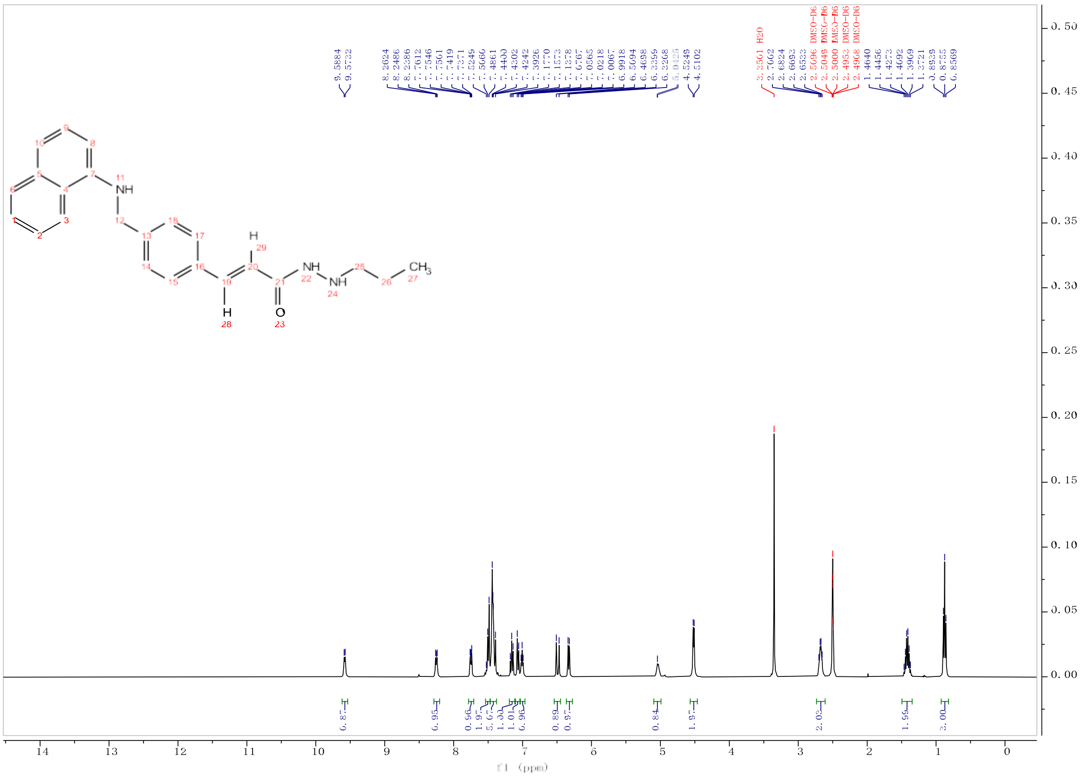

^{1}H NMR spectra for compound **25f**

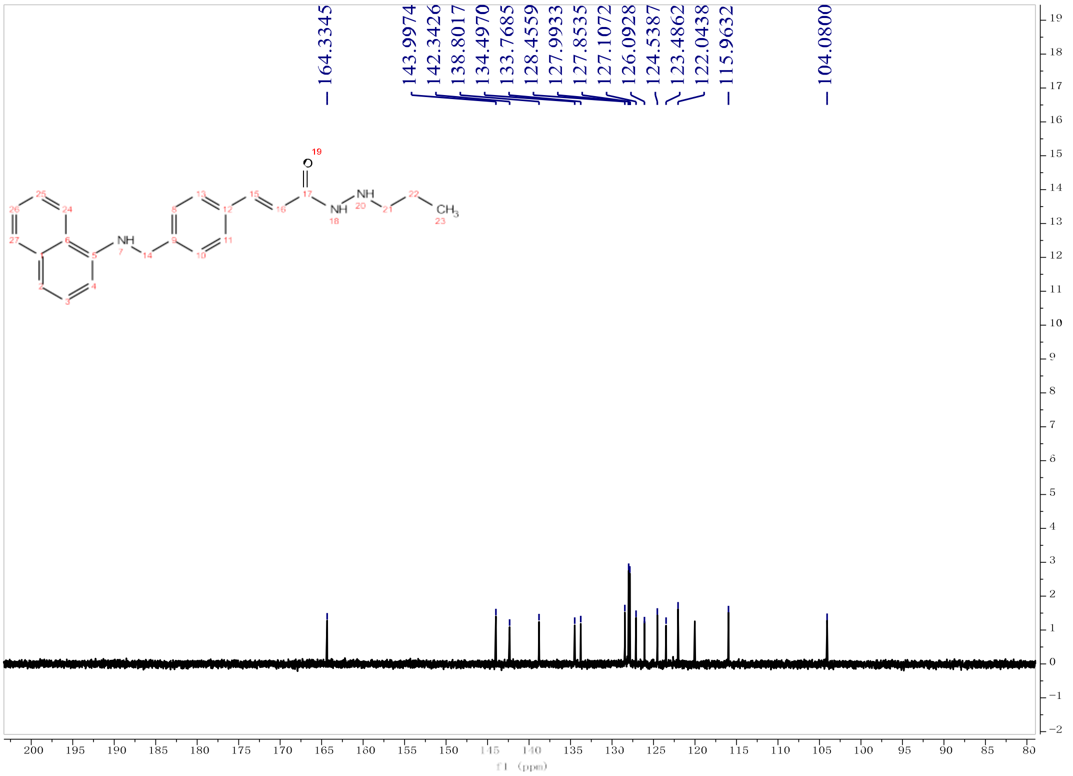

^{13}C NMR spectra for compound **25f**

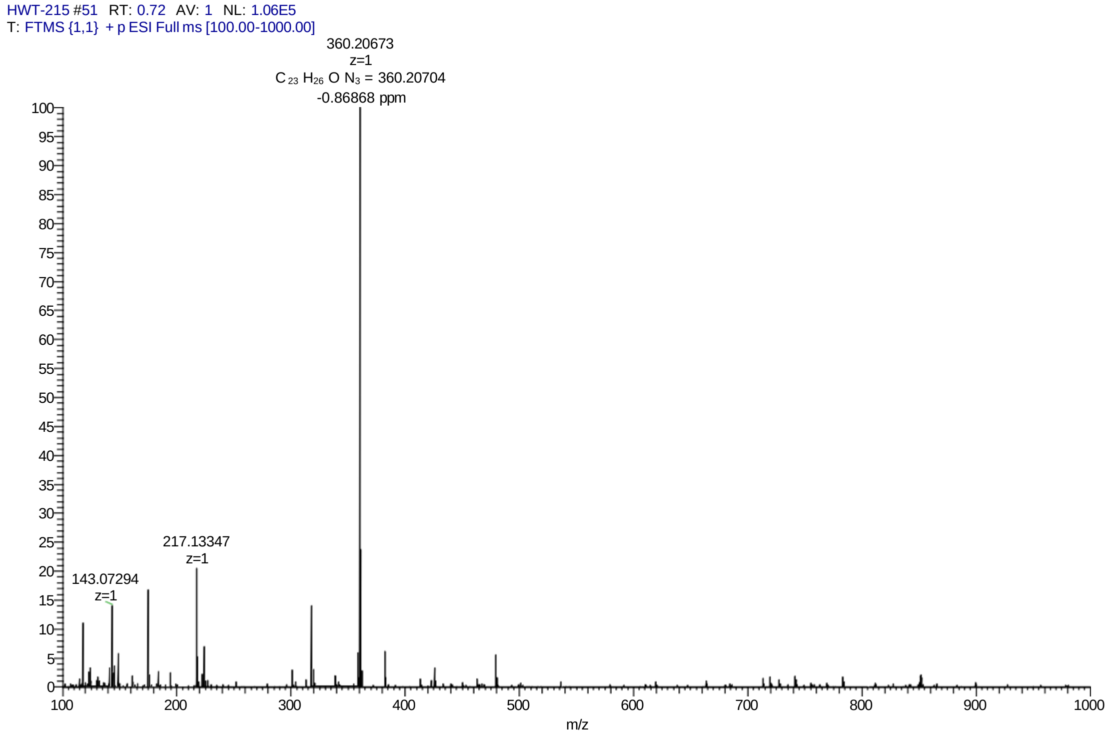

HRMS (AP-ESI) spectra for compound **25f**

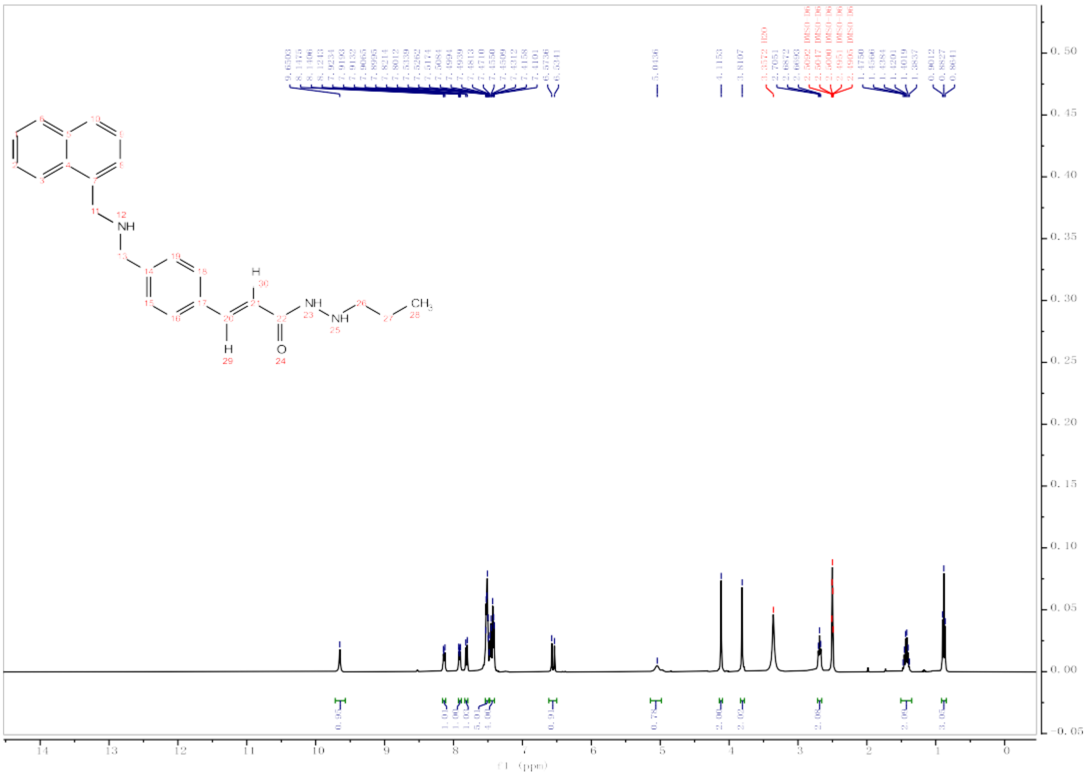

^{1}H NMR spectra for compound **25g**

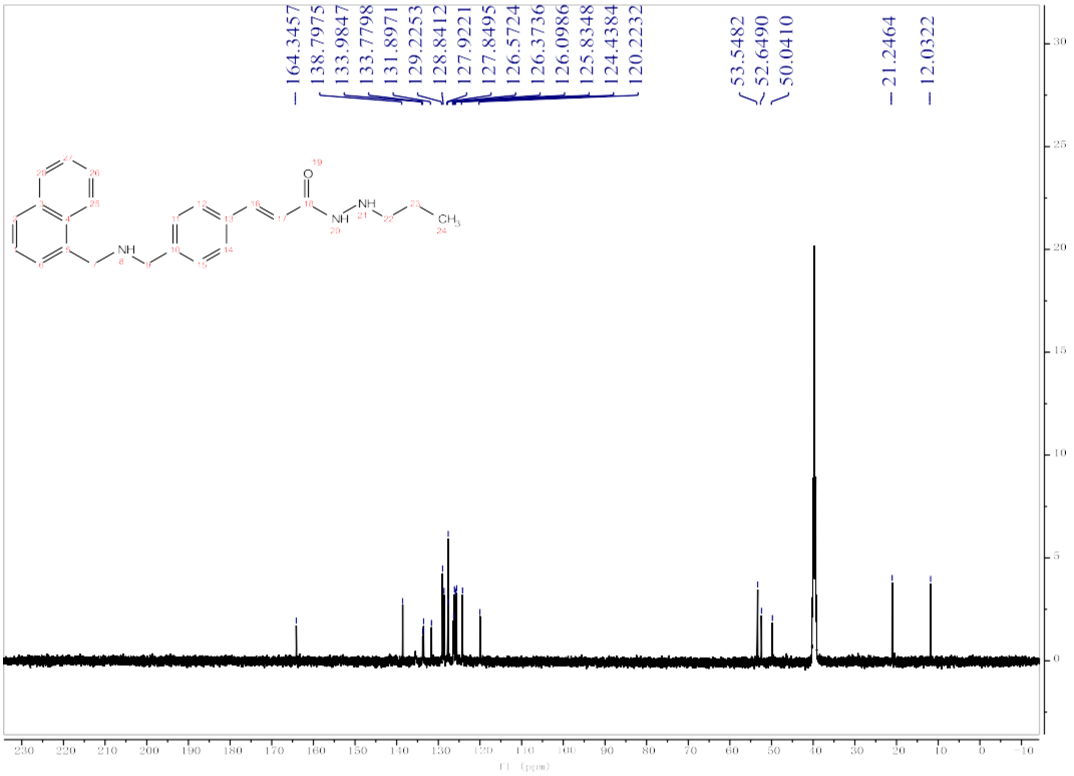

^{13}C NMR spectra for compound **25g**

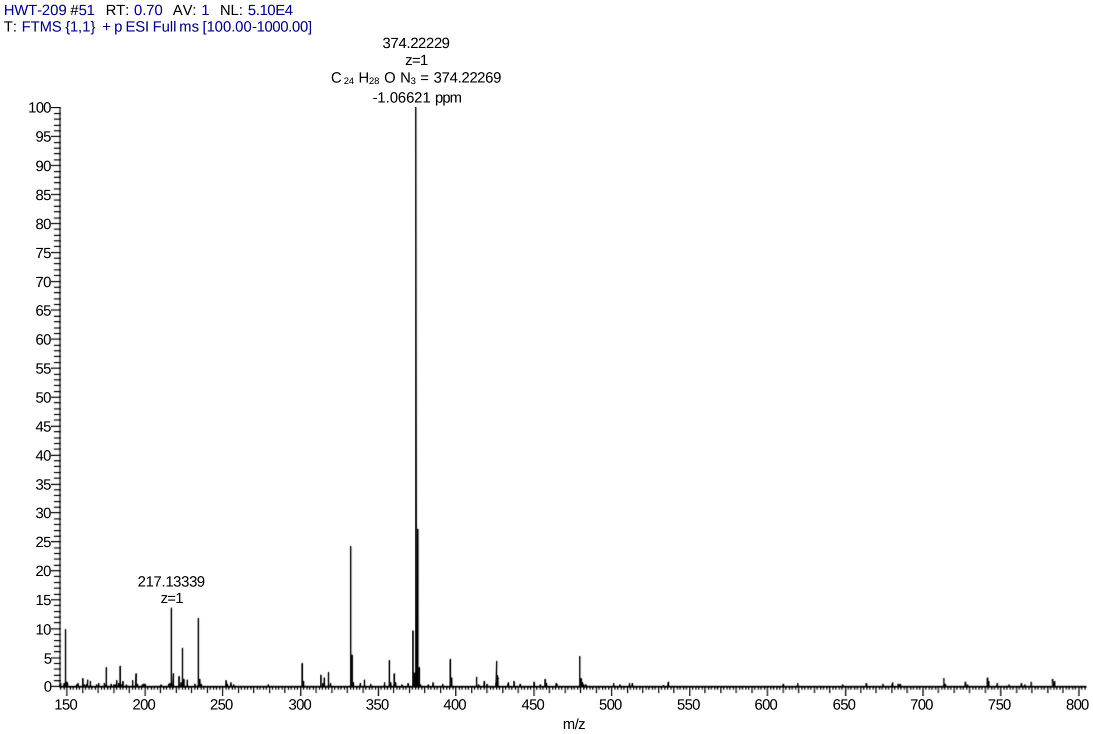

HRMS (AP-ESI) spectrum of compound **25g**

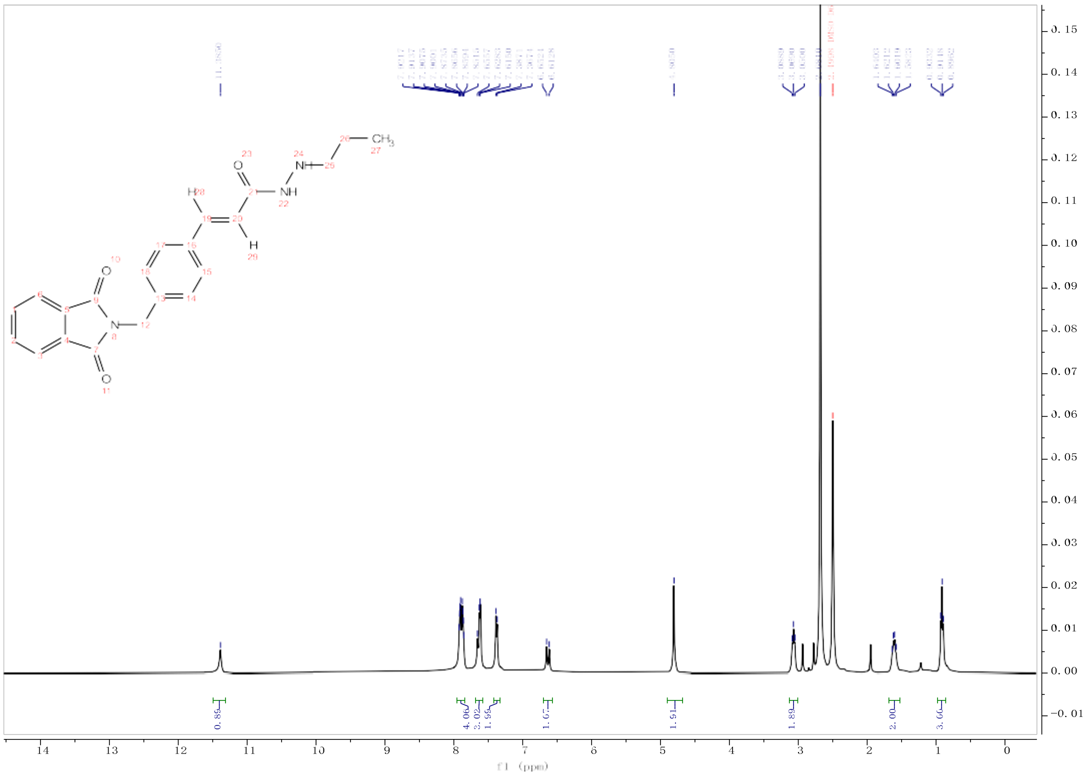

^{1}H NMR spectra for compound **30**

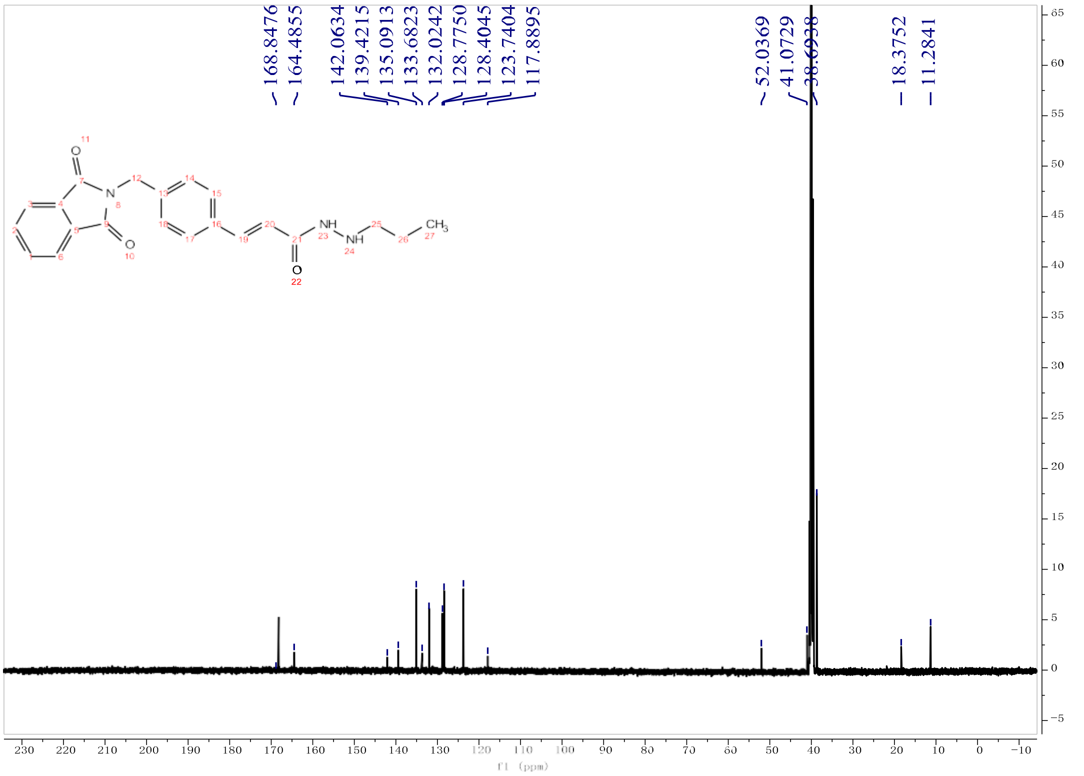

^{13}C NMR spectra for compound **30**

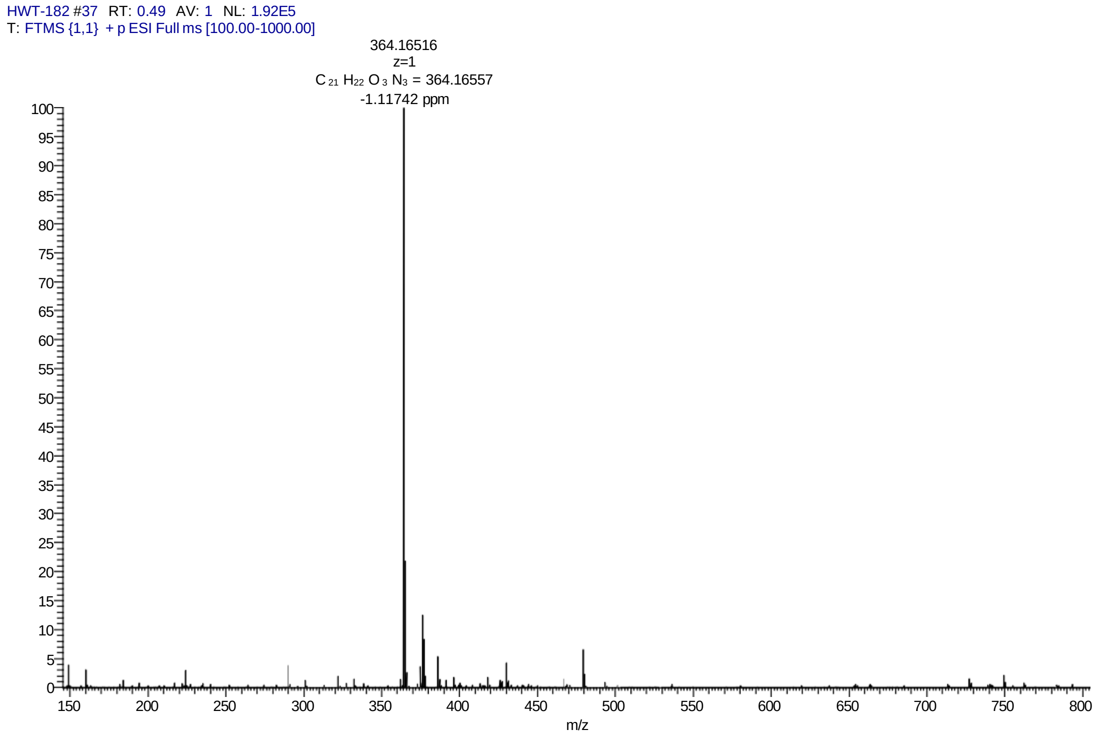

HRMS (AP-ESI) spectrum of compound **30**

HPLC traces and purity of the target compounds.

All target compounds were > 95% pure by HPLC analysis, performed on an Agilent 1260 Infinity II HPLC instrument using an Agilent 5 TC-C18(2) C18 column (5 μm, 4.6 mm × 250 mm) using a equivalent of potassium phosphate buffer/acetonitrile (60 : 40 to 30 : 70).

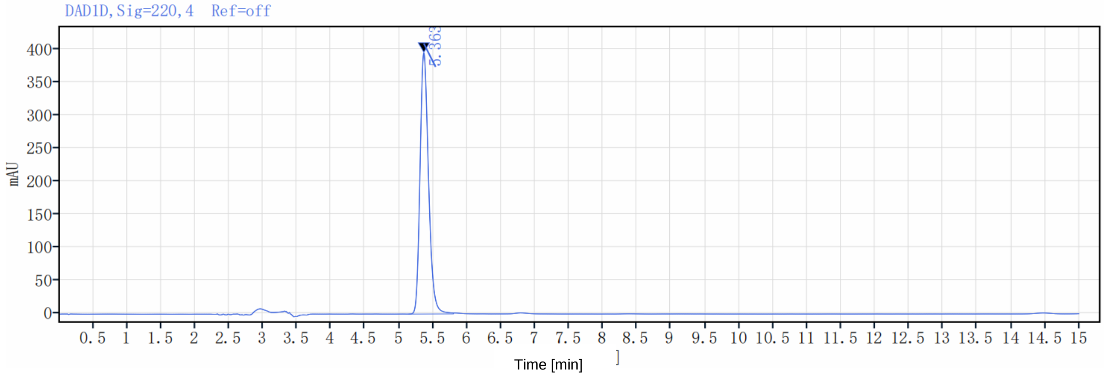

HPLC analysis of compound **9a** (purity: 100%)

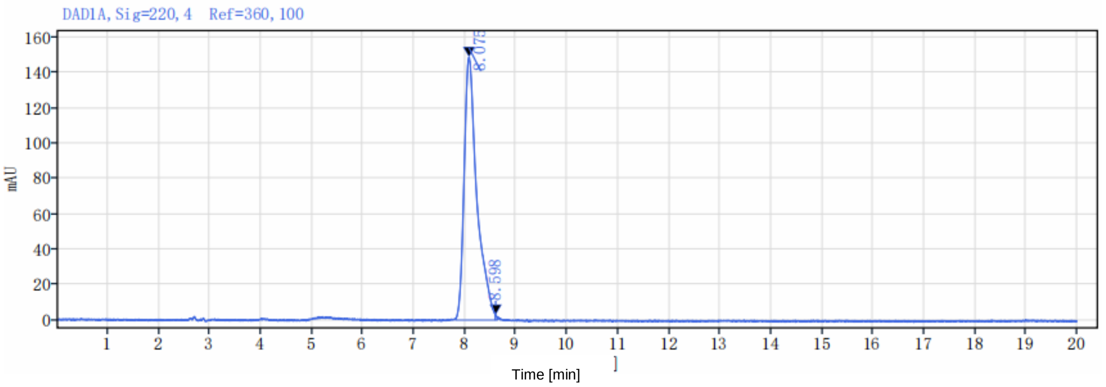

HPLC analysis of compound **9b** (purity: 99.80%)

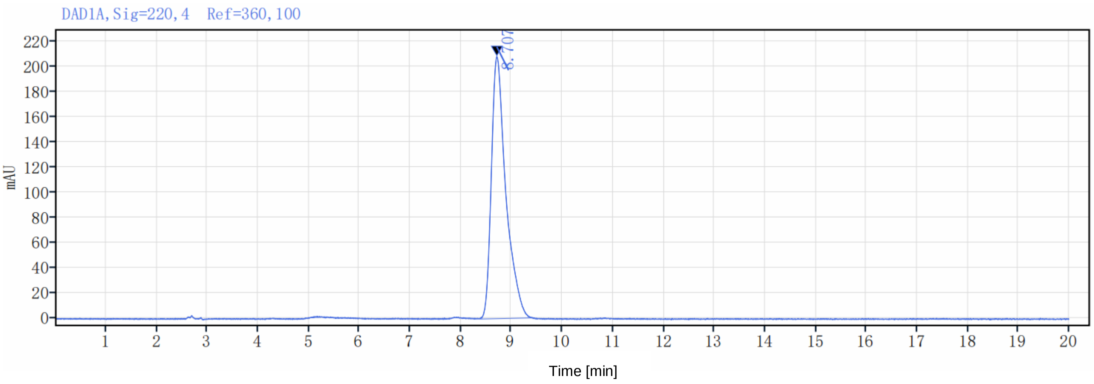

HPLC analysis of compound **9c** (purity: 100%)

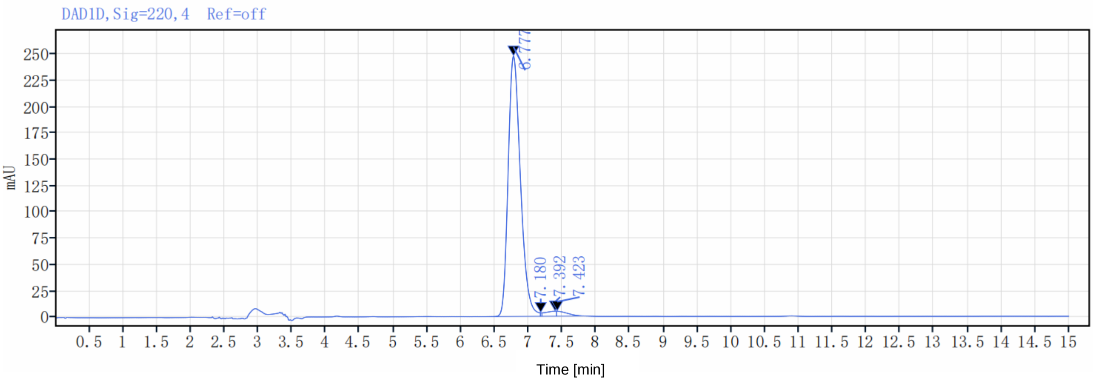

HPLC analysis of compound **9d** (purity: 96.65%)

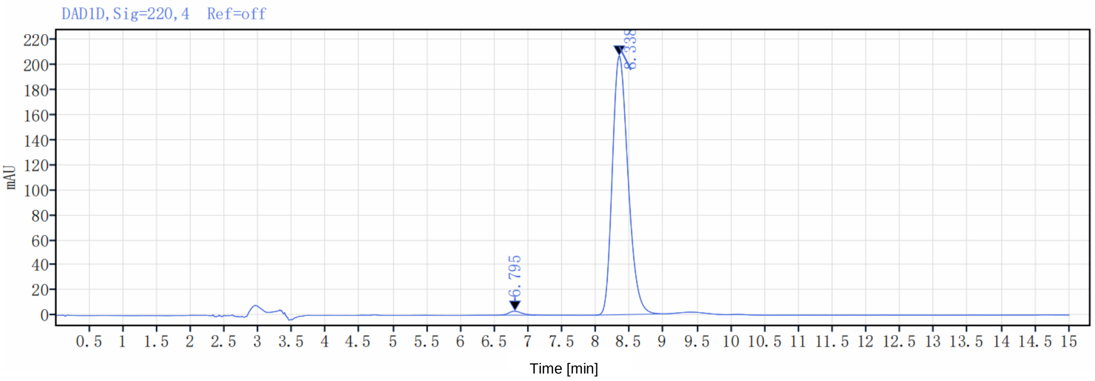

HPLC analysis of compound **9e** (purity: 98.71%)

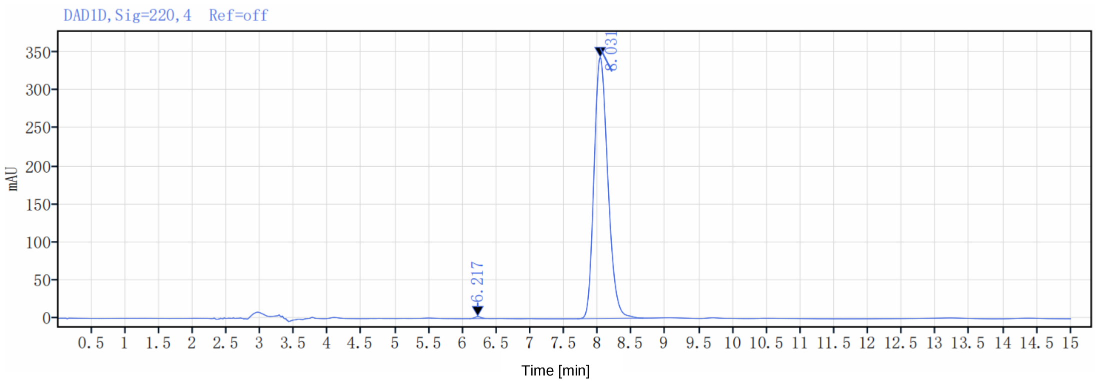

HPLC analysis of compound **9f** (purity: 99.64%)

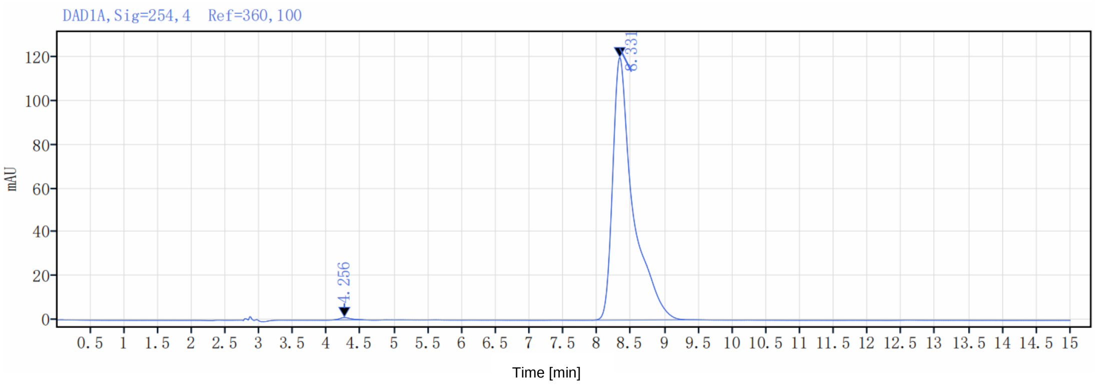

HPLC analysis of compound **9g** (purity: 99.52%)

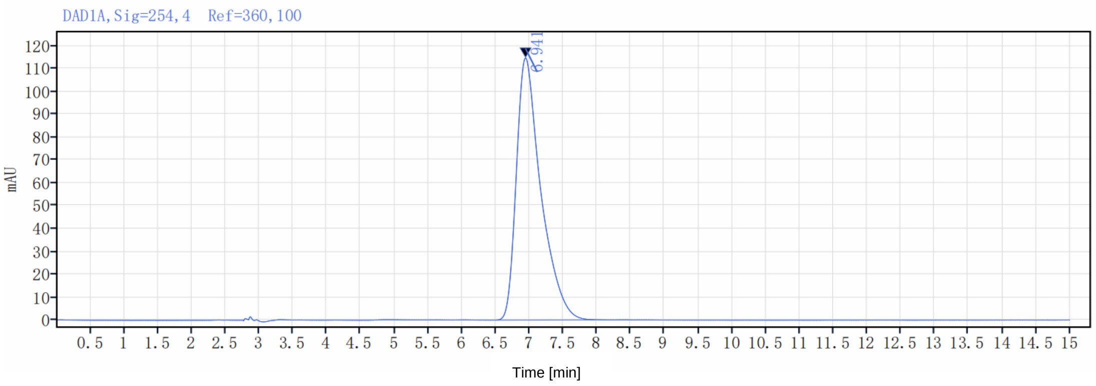

HPLC analysis of compound **9h** (purity: 100%)

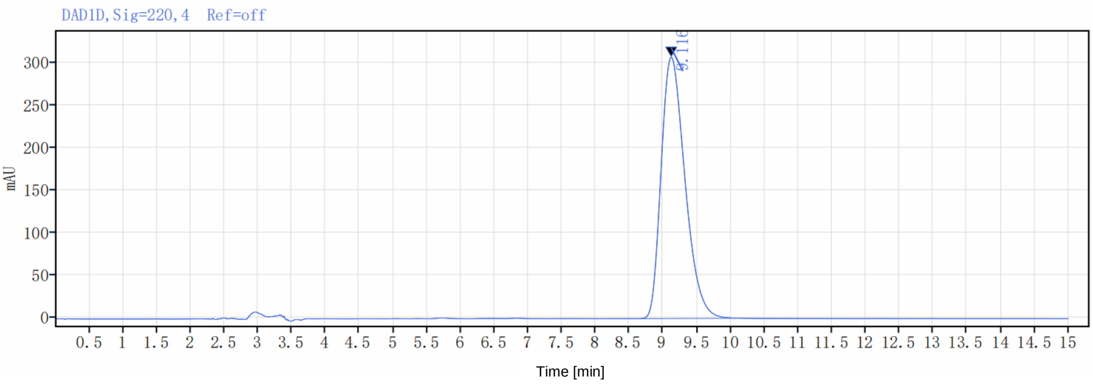

HPLC analysis of compound **9i** (purity: 100%)

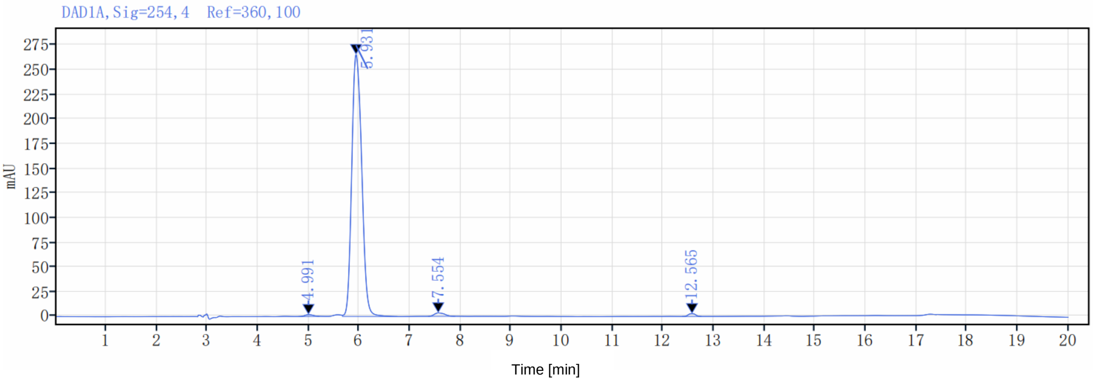

HPLC analysis of compound **9j** (purity: 97.40%)

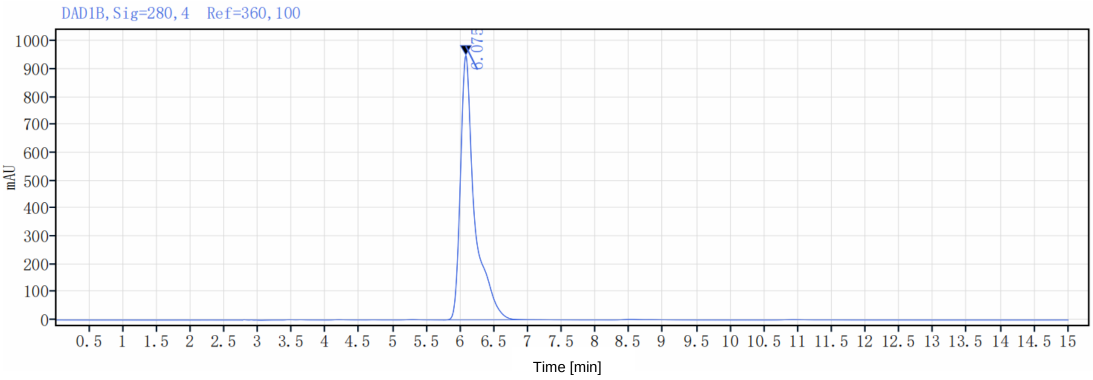

HPLC analysis of compound **9k** (purity: 100%)

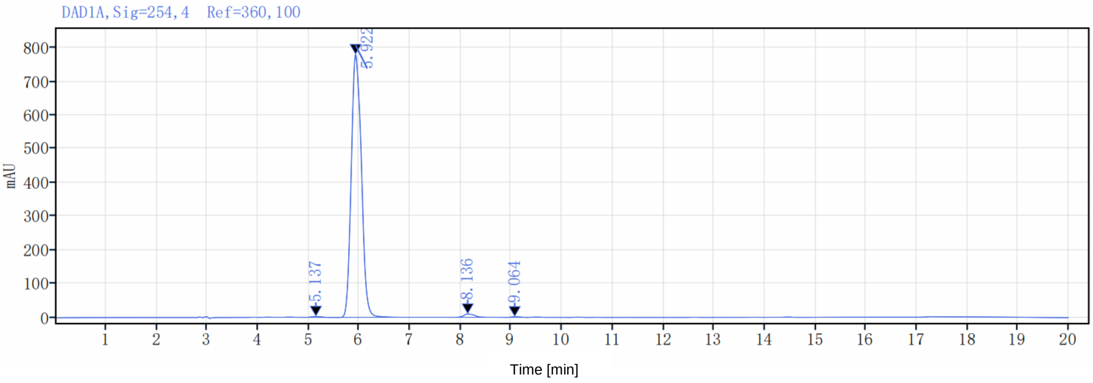

HPLC analysis of compound **9l** (purity: 98.12%)

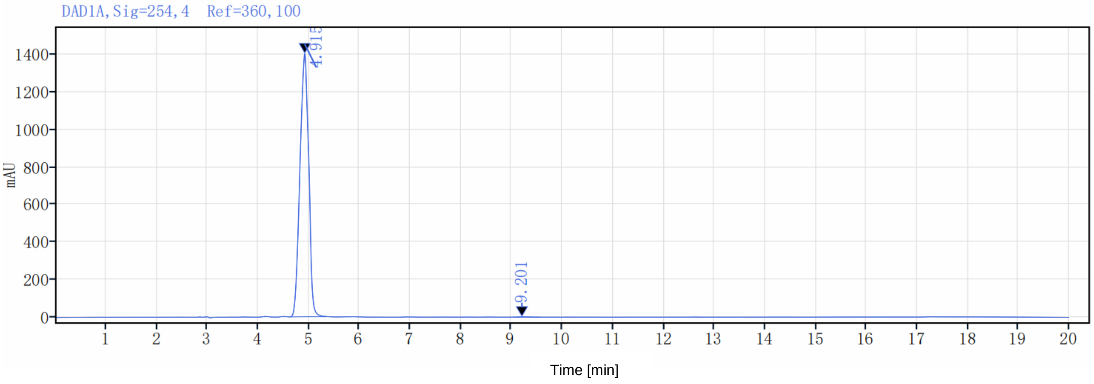

HPLC analysis of compound **9m** (purity: 99.81%)

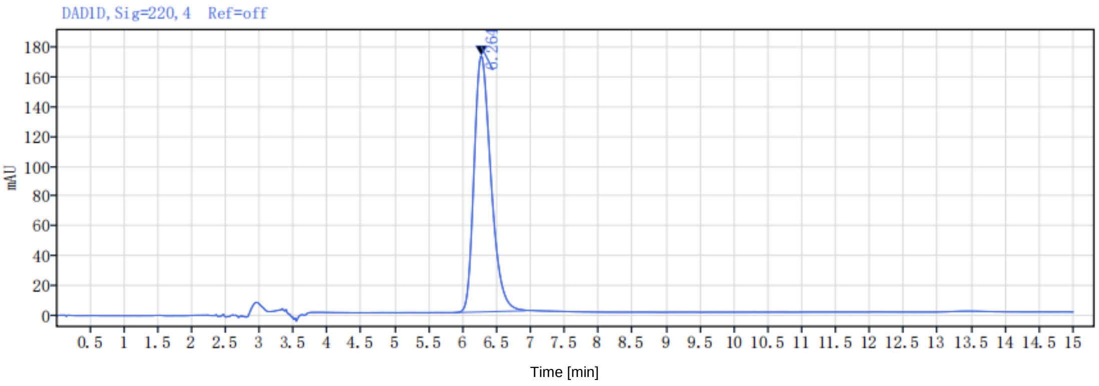

HPLC analysis of compound **14a** (purity: 100%)

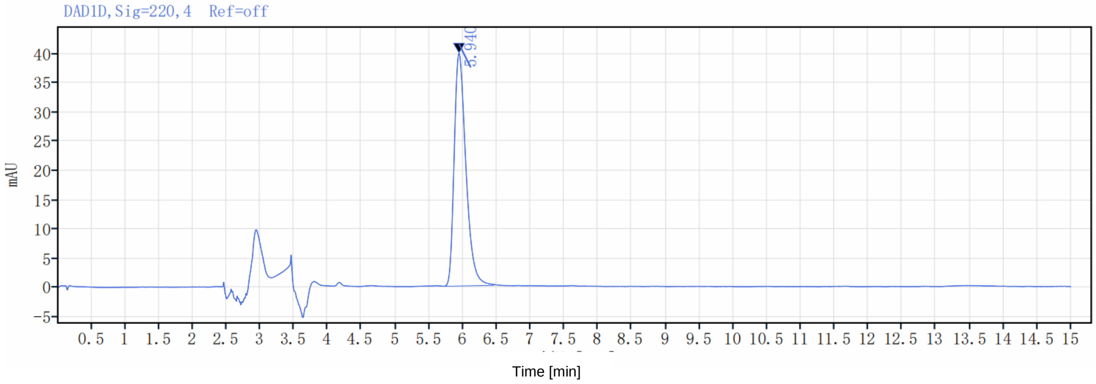

HPLC analysis of compound **14b** (purity: 100%)

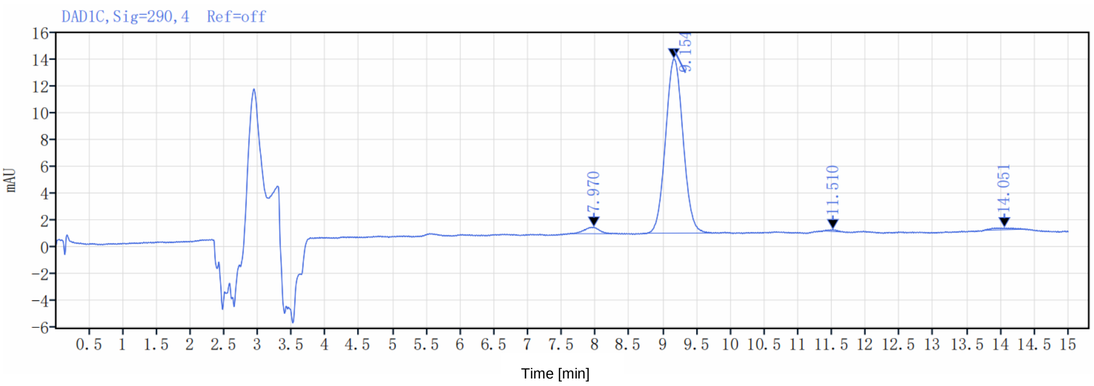

HPLC analysis of compound **14c** (purity: 95.40%)

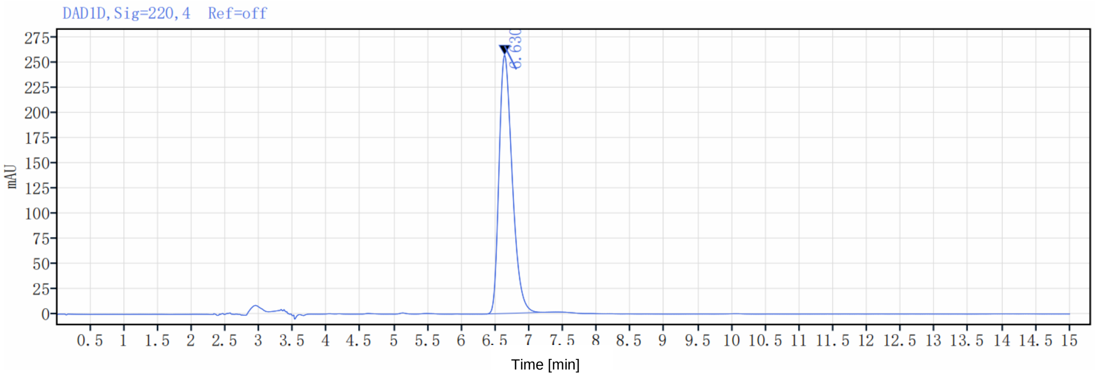

HPLC analysis of compound **14d** (purity: 100%)

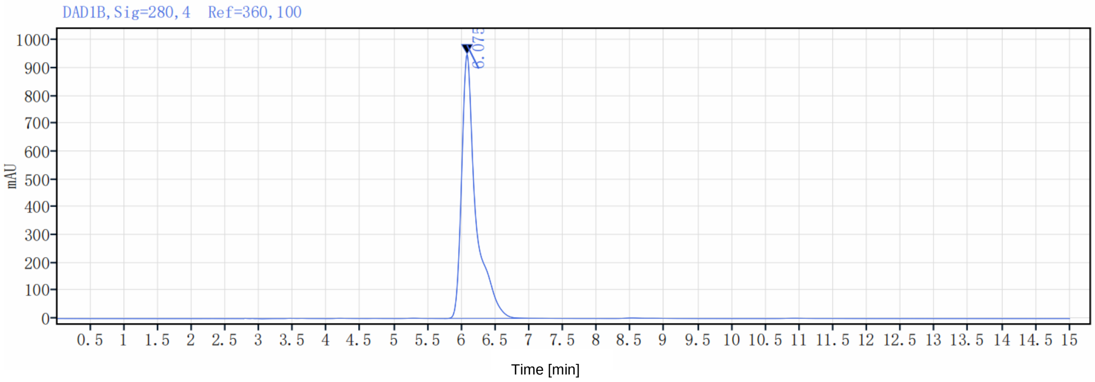

HPLC analysis of compound **14e** (purity: 100%)

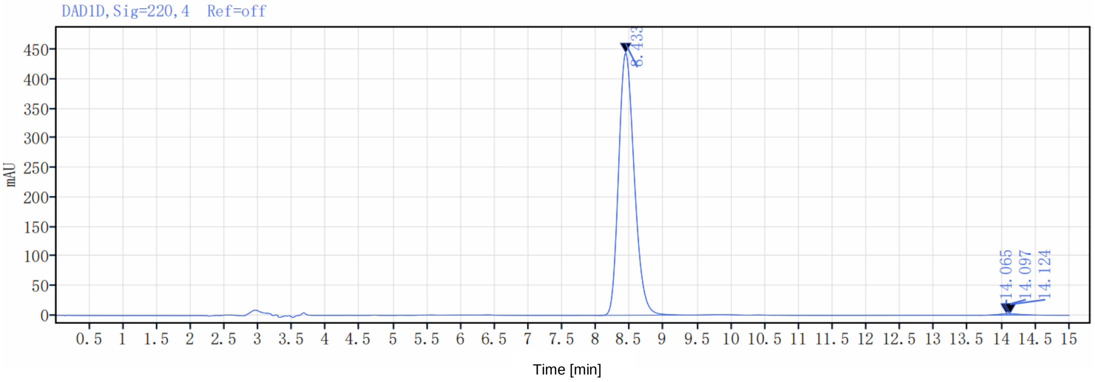

HPLC analysis of compound **14f** (purity: 100%)

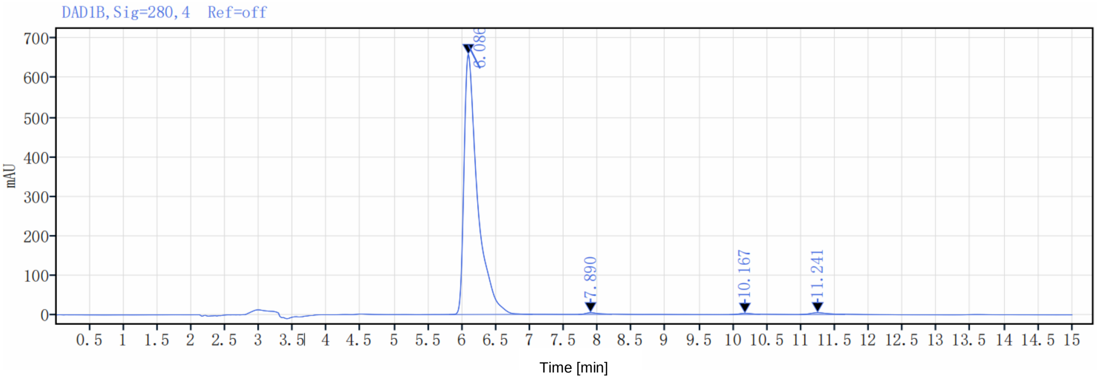

HPLC analysis of compound **19a** (purity: 95.7%)

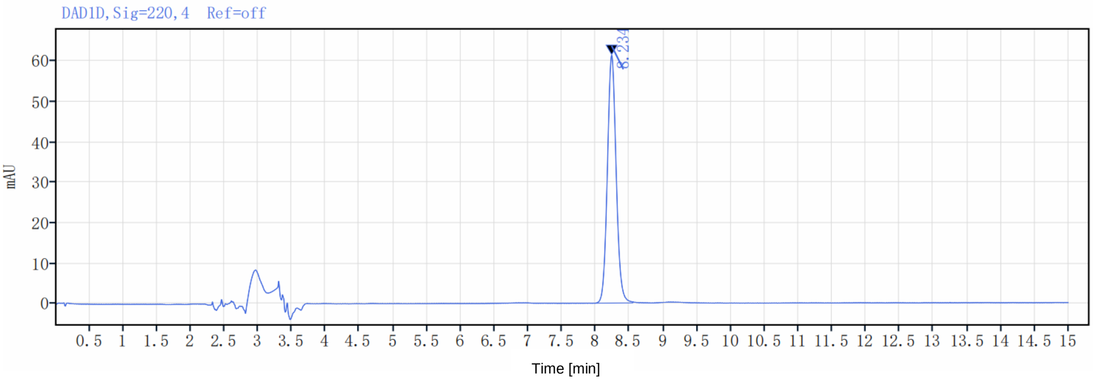

HPLC analysis of compound **19b** (purity: 100%)

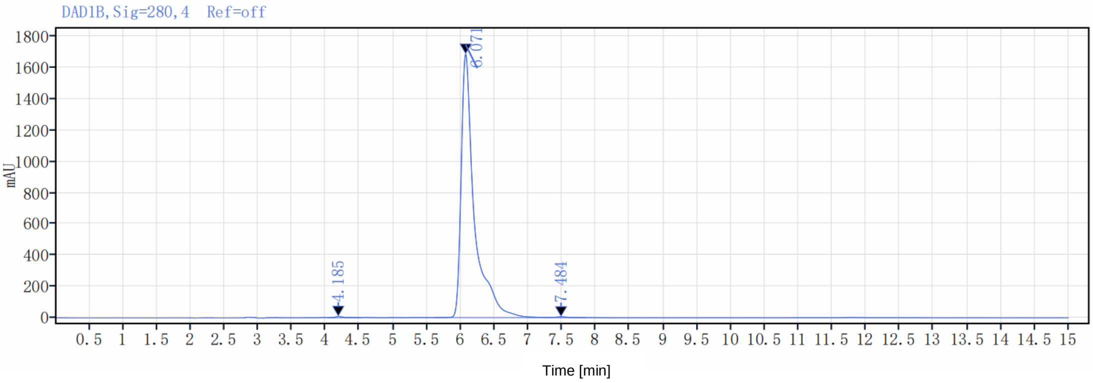

HPLC analysis of compound **19c** (purity: 99.27%)

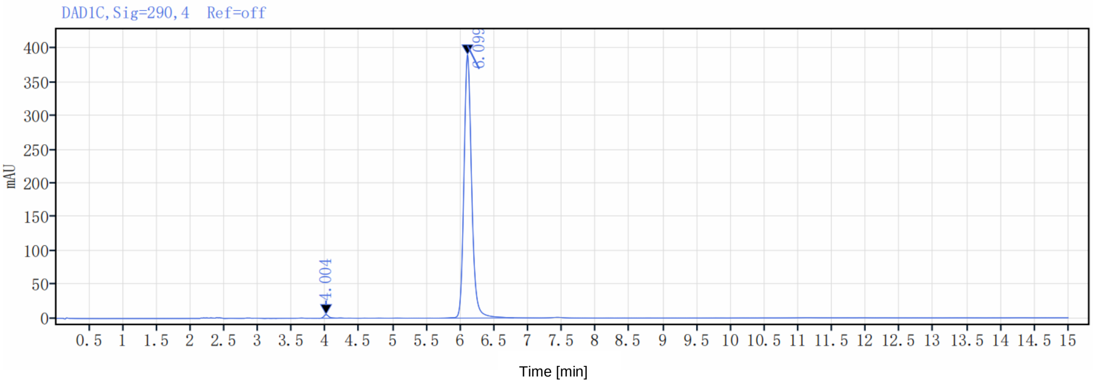

HPLC analysis of compound **19d** (purity: 99.12%)

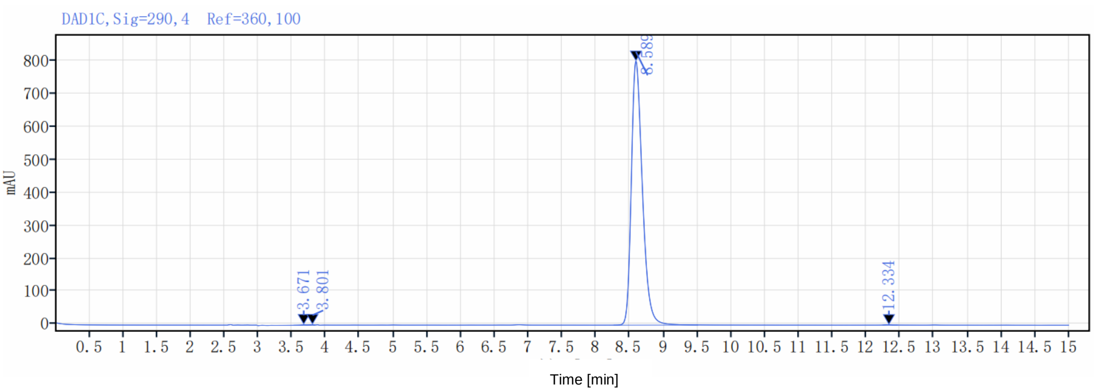

HPLC analysis of compound **25a** (purity: 99.77%)

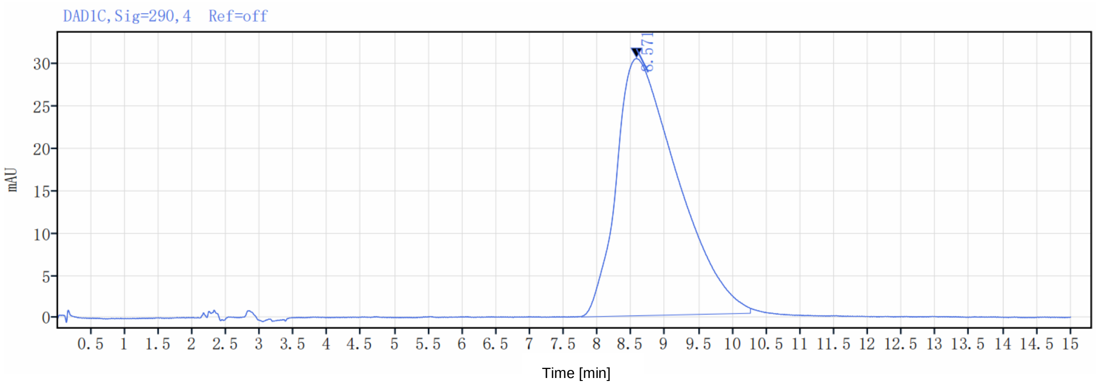

HPLC analysis of compound **25b** (purity: 100%)

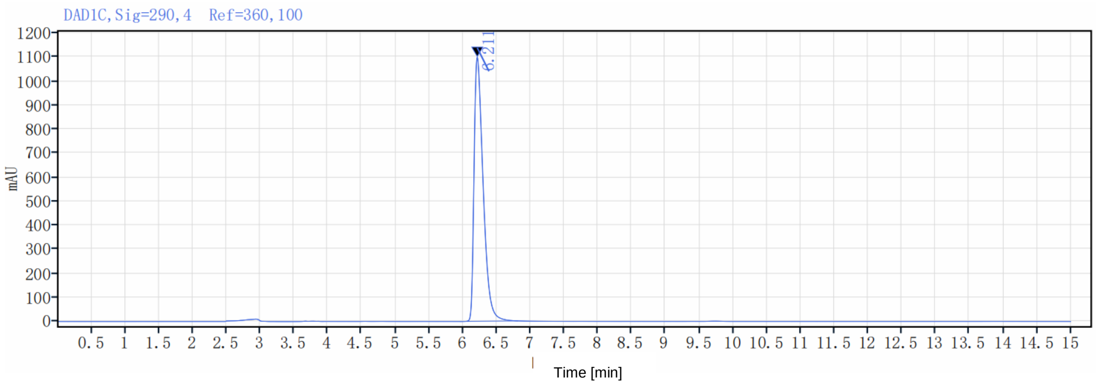

HPLC analysis of compound **25c** (purity: 100%)

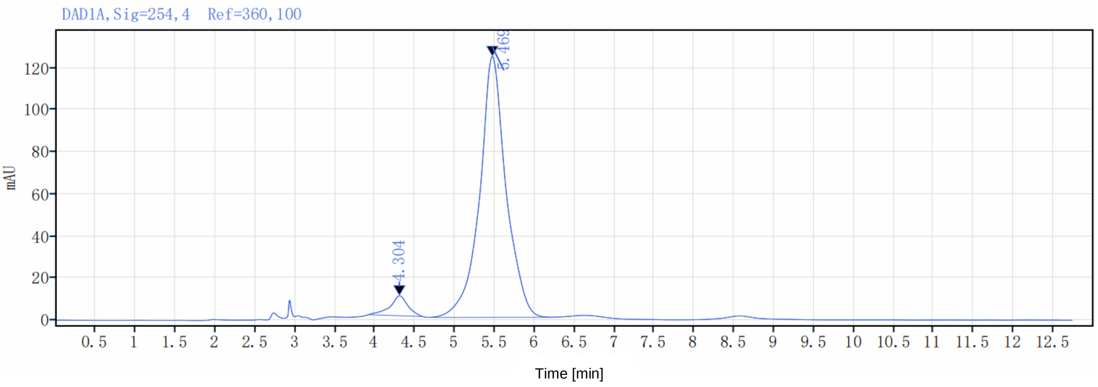

HPLC analysis of compound **25d** (purity: 95.11%)

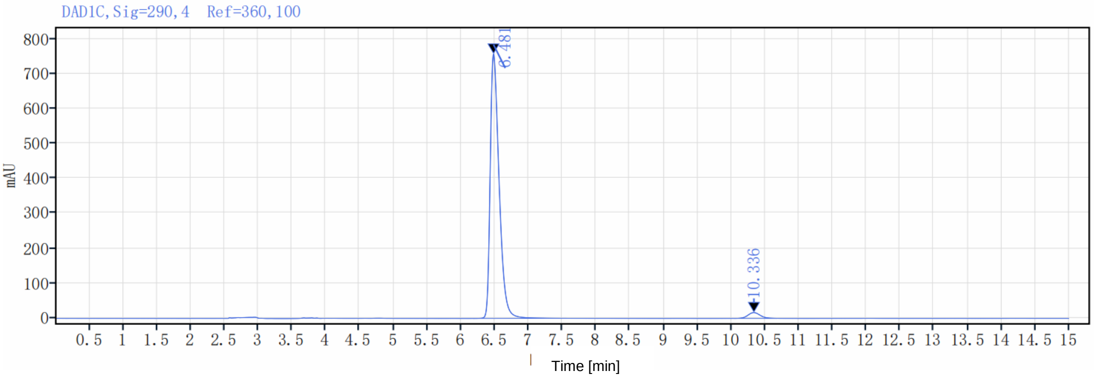

HPLC analysis of compound **25e** (purity: 97.02%)

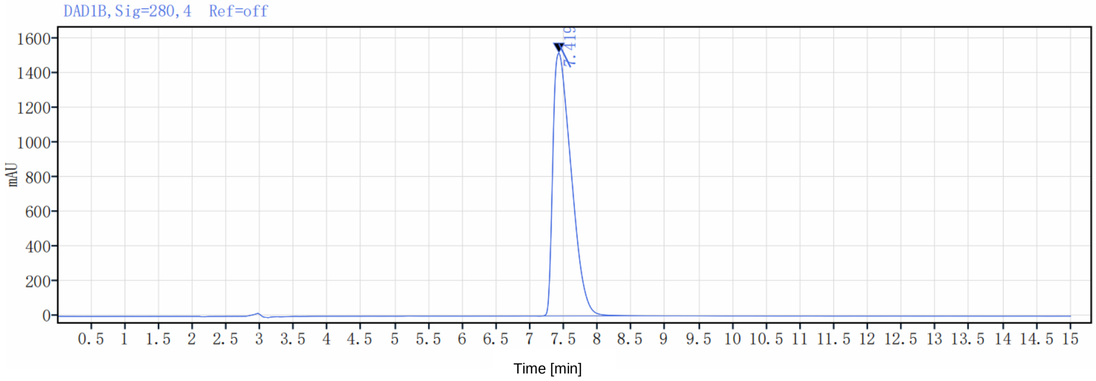

HPLC analysis of compound **25f** (purity: 100%)

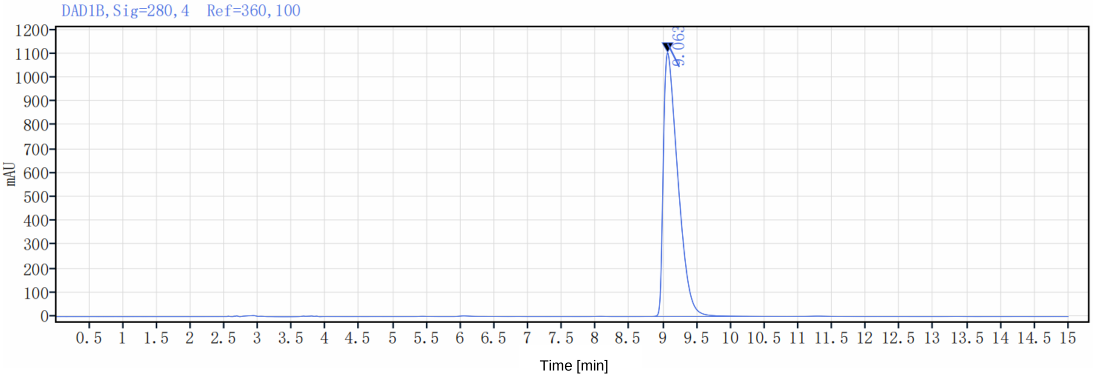

HPLC analysis of compound **25g** (purity: 100%)

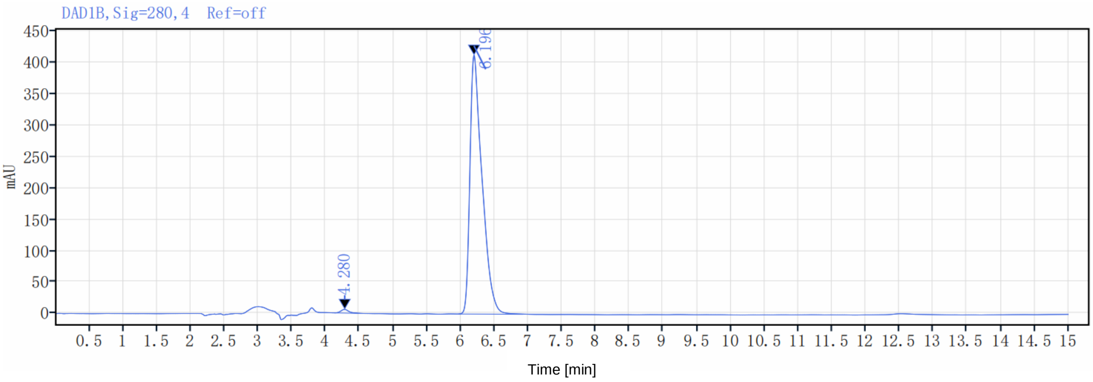

HPLC analysis of compound **30**(purity: 99.08%)
